# A Cooperative Cobalt-Driven System for One-Carbon Extension in the Synthesis of (*Z*)-Silyl Enol Ethers from Aldehydes: Unlocking Regio- and Stereoselectivity

**DOI:** 10.1021/jacs.3c10491

**Published:** 2023-12-12

**Authors:** Soumyashree Jena, Lars Frenzen, Vishal Chugh, Jiajun Wu, Thomas Weyhermüller, Alexander A. Auer, Christophe Werlé

**Affiliations:** † Max Planck Institute for Chemical Energy Conversion, Stiftstr. 34−36, 45470 Mülheim an der Ruhr, Germany; ‡ Ruhr University Bochum, Universitätsstr. 150, 44801 Bochum, Germany; § Max-Planck-Institut für Kohlenforschung, Kaiser-Wilhelm-Platz 1, 45470 Mülheim an der Ruhr, Germany

## Abstract

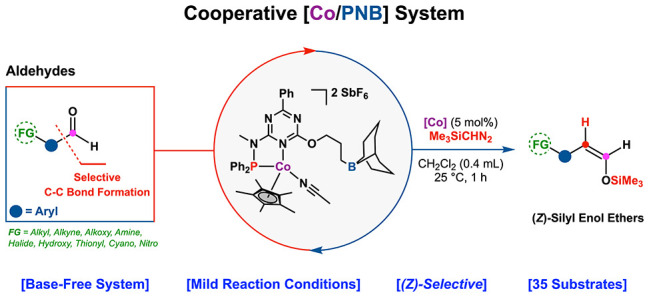

The research presented herein explores a cobalt-based catalytic system, distinctively featuring a cooperative boron-centric element within its intricate ligand architecture. This system is strategically engineered to enable the integration of a singular carbon atom into aldehydes, a process culminating in the production of (*Z*)-silyl enol ethers. Beyond offering an efficient one-pot synthesis route, this method adeptly overcomes challenges inherent to conventional techniques, such as the need for large amounts of additives, restrictive functional group tolerance, and extreme reaction temperatures. Initial mechanistic studies suggest the potential role of a cobalt–carbene complex as a catalytically significant species and underscore the importance of the borane segment. Collectively, these observations highlight the potential of this system in advancing complex bond activation pursuits.

Naturally occurring catalysts exhibit remarkable precision in controlling chemical bond dynamics, using synergistic interactions within their structures to selectively activate and transform substrates.^1^ Scientists have replicated these phenomena in laboratory settings, enabling the activation and transformation of traditionally unreactive small molecules.^2,3^

The focal point of our research program encompasses the advancement of dynamic and adaptive molecular systems capable of facilitating complex bond activations.^4^ Such systems are particularly salient in the domain of chemical energy conversion, where obstacles arise due to variable energy supplies and inconsistent feedstock quality. Through modulation of the reactivity, these dynamic catalyst systems target efficient resource allocation and optimization.

Leveraging our previous advancements, we have now concentrated our efforts on the formation of carbon–carbon bonds,^5^ specifically the incorporation of a single carbon atom into aldehydes to produce silyl enol ethers, a standing challenge in *bond activation*.

Silyl enol ethers are pivotal intermediates in many synthetic transformations.^6^ While traditional synthesis methods have proven insightful and in certain instances exceptionally elegant, they come with limitations.^7,8^ These include the need for excessive amounts of base, functional group sensitivity, potential over-reductions, and strict reaction conditions (Scheme 1A).^9^

**Scheme 1:**
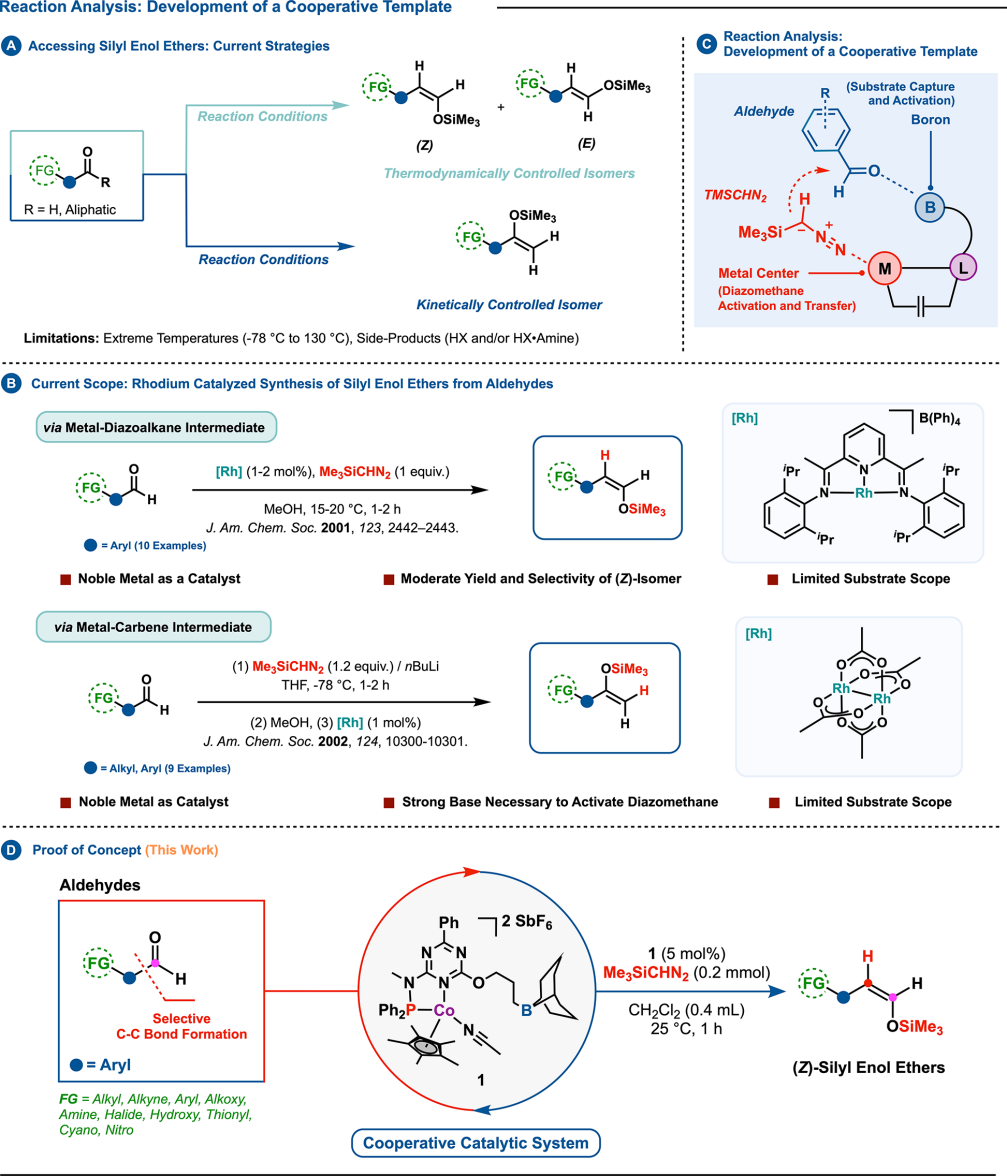
Development of a Cooperative Catalytic System for Selective (*Z*)-Silyl Enol Ether Synthesis from Aldehydes (A) Current methods.^8^ (B) Syntheses using rhodium complexes.^13b,13c^ (C) Guiding principle for our approach. (D) Concept validation.

In the pursuit of aligning the synthesis processes with environmentally conscious methodologies advancing the principles of *Green Chemistry*,^10^ researchers have been directed toward employing transition metals.^11^

However, in the prevailing research landscape, a substantial number of studies preferentially focus on noble metals^12,8q^ and significantly emphasize the usage of ketones as starting materials (Scheme 1B).^13,12b^ In contrast, protocols that explore the addition of a single carbon atom to aldehydes, culminating in the synthesis of silyl enol ethers, especially when utilizing base metal catalysts, have been exceedingly limited.

In our quest to navigate the complexities specific to this area of research, we devised a strategy tailored to the unique demands of the target substrate. Central to our approach is a catalytic system, integrating a cobalt center synergistically paired with a boron site in its secondary coordination sphere.^14,15^ This configuration is engineered to proficiently engage in simultaneous activation: targeting the carbonyl unit at the boron site and (trimethylsilyl)diazomethane (Me_3_SiCHN_2_) at the cobalt site. We propose that this cooperative dual-activation mode not only enables the pivotal carbon atom transfer necessary for C–C bond genesis but also orchestrates the delivery of the −SiMe_3_ group to the nascent alcohol functionality, all while expelling nitrogen as the sole byproduct (Scheme 1C).^16^

Considering the specified parameters, a procedure is proposed for one-carbon extension in the synthesis of (*Z*)-silyl enol ethers derived from aldehydes, utilizing a Co-based catalytic system (Scheme 1D). This catalytic framework demonstrates exceptional versatility, accommodating a broad spectrum of substrates without the necessity of specific functional groups. Importantly, this system operates efficiently under comparatively mild conditions, thereby obviating the need for stringent temperature controls and ensuring the preservation of other potentially susceptible functional groups.

## Results and Discussion

**Scheme 2:**
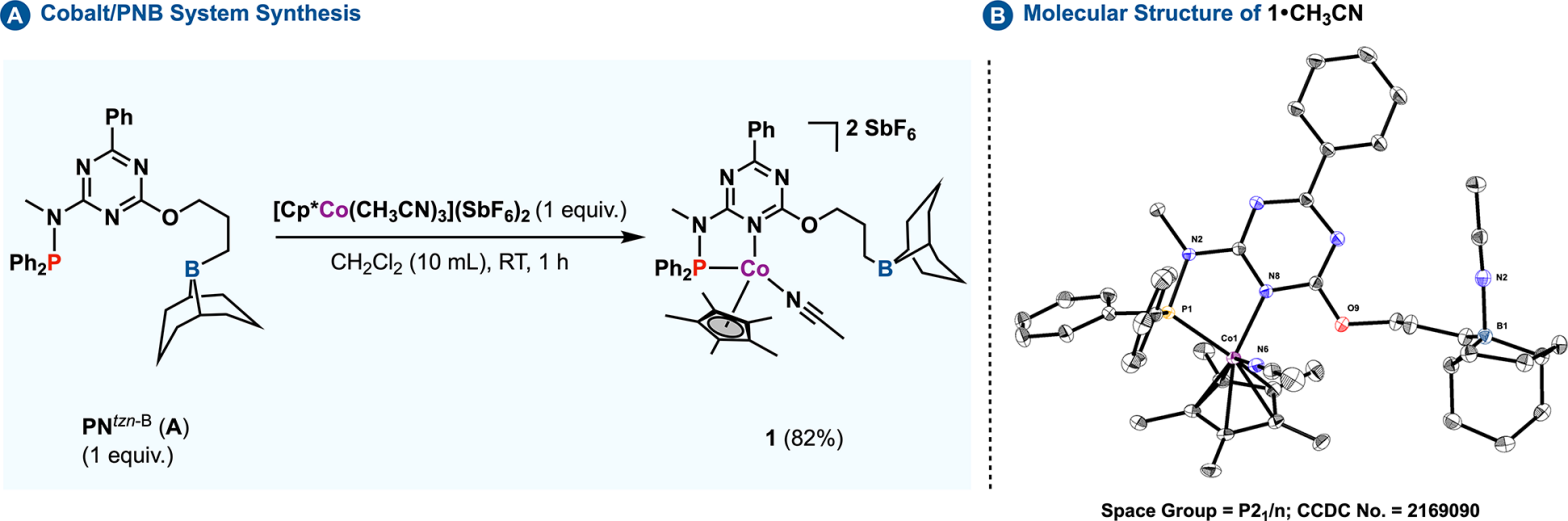
(A) Synthesis of Complex **1**; (B) 40% Probability Thermal Ellipsoid of **1**•**CH**_**3**_**CN** with Partial Atom Numbering SbF_6_ anions, protons, and extra solvents were omitted for clarity.

To assess the viability of the proposed strategy, the initial step involved the synthesis of cobalt complex **1** (Scheme 2). This complex encompasses a triazine-based **PN**^*tzn*-B^ ligand (**A**).^4d^ The ligand structure was designed to merge an electron-withdrawing triazine core with an electron-donating phosphine. A boron component was incorporated into the secondary coordination sphere, specifically to mediate substrate capture and activation during the reaction sequence. The targeted Co(III) complex was synthesized from the precursor [Cp*Co(CH_3_CN)_3_](SbF_6_)_2_ (**7**).^17^ A detailed protocol for synthesis and complex characterization, which supports the illustrated binding scenario in Scheme 2, is further elaborated in the Supporting Information (SI).^18^

To evaluate the protocol’s broad applicability, standard reaction conditions were established using 4-methyl benzaldehyde. The optimized method, detailed in Table 1, emerged from a comprehensive screening of the various conditions. Initial trials utilized catalyst **1** in acetonitrile at 40 °C with 1 equiv of Me_3_SiCHN_2_, yielding (*Z*)-silyl enol ether **3b** (76%) and (*E*)-silyl enol ether **4b** (14%).^19^ Further optimization explored various solvents. Nonpolar options like toluene and mesitylene lowered selectivity (Table 1, entry 2; Table S1, entries 4–6), while dichloromethane yielded the best performance (Table 1, entry 4). Temperature studies indicated that variations from 25 °C had minimal effect on selectivity (Table 1, entries 5–6). Modifying catalyst loading and shortening reaction time to 1 h fine-tuned the protocol, leading to an 87% yield of (*Z*)-silyl enol ether **3b** (Table 1, entry 9). A control experiment without a catalyst showed no aldehyde conversion (Table 1, entry 10).

**Table 1:**
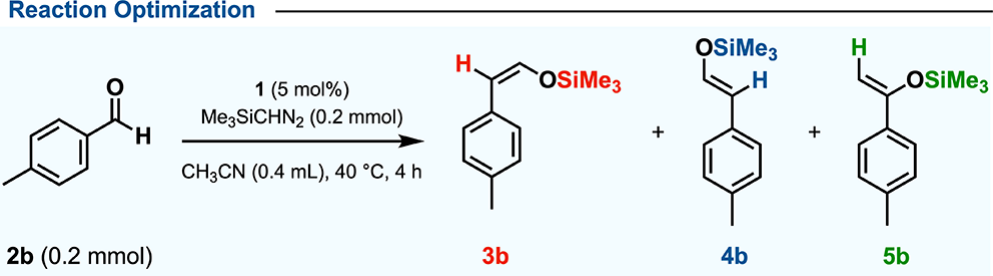

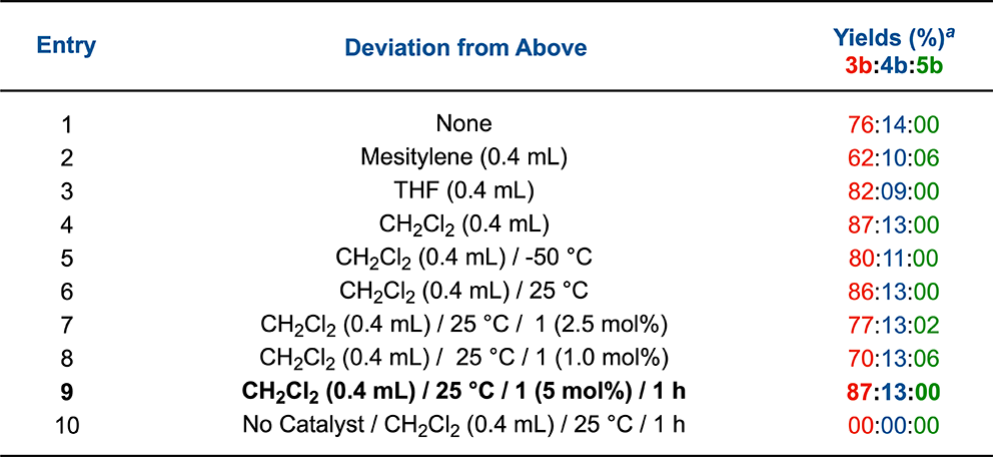
Reaction Optimization

a Yield (%) is based on ^1^H NMR relative to mesitylene (0.2 mmol) as an internal standard.

**Scheme 3:**
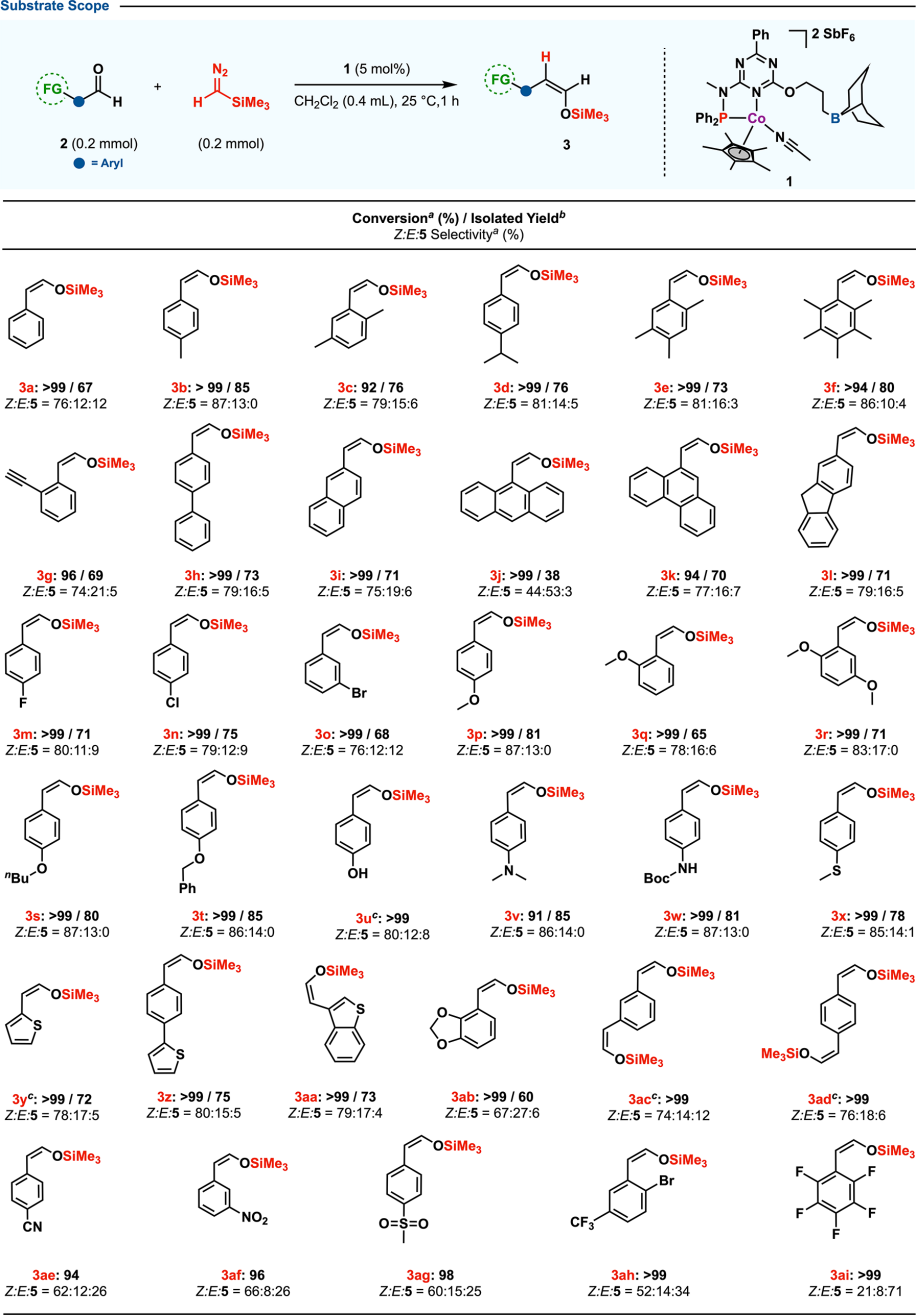
Conversion and selectivity based on ^1^H NMR with mesitylene (0.2 mmol) standard. Isolated yield of (*Z*)-silyl enol ethers. Used 2 equiv of Me_3_SiCHN_2_.

The protocol’s versatility was assessed through a broad substrate scope (Scheme 3). Many substrates, despite varied functionalities, reacted efficiently, highlighting the protocol’s robustness. Alkyl-substituted aryl aldehydes showed strong selectivity for (*Z*)-silyl enol ether synthesis (**3b**–**f**). Biphenyl (**2h**) and polyaromatic aldehydes (**2i**–**l**) also converted well, with moderate to satisfactory yields. Halogenated (**2m**–**o**) and electron-rich (**2p**–**x**) substrates showed similar selectivity, the latter reducing the level of unwanted compound **5** formation. The protocol effectively handled heterocyclic (**2y**, **2z**, **2aa**–**ab**) and base-sensitive substrates like hydroxyl (**2u**), alkyne (**2g**), and Boc-protected amines (**2w**). Dual aldehyde substrates (**2ac**–**ad**) mostly yielded (*Z*)-silyl enol ethers. However, substrates featuring electron-withdrawing groups such as cyano (**2ae**), nitro (**2af**), and trifluoromethyl (**2ah**) also led to the formation of internal silyl enol ether (**5ae**, **5af**, and **5ah**). This behavior could potentially be attributed to the limited migration capabilities of these aryl groups, which seem to favor hydride migration over that of their aryl counterparts.^20^

**Scheme 4:**
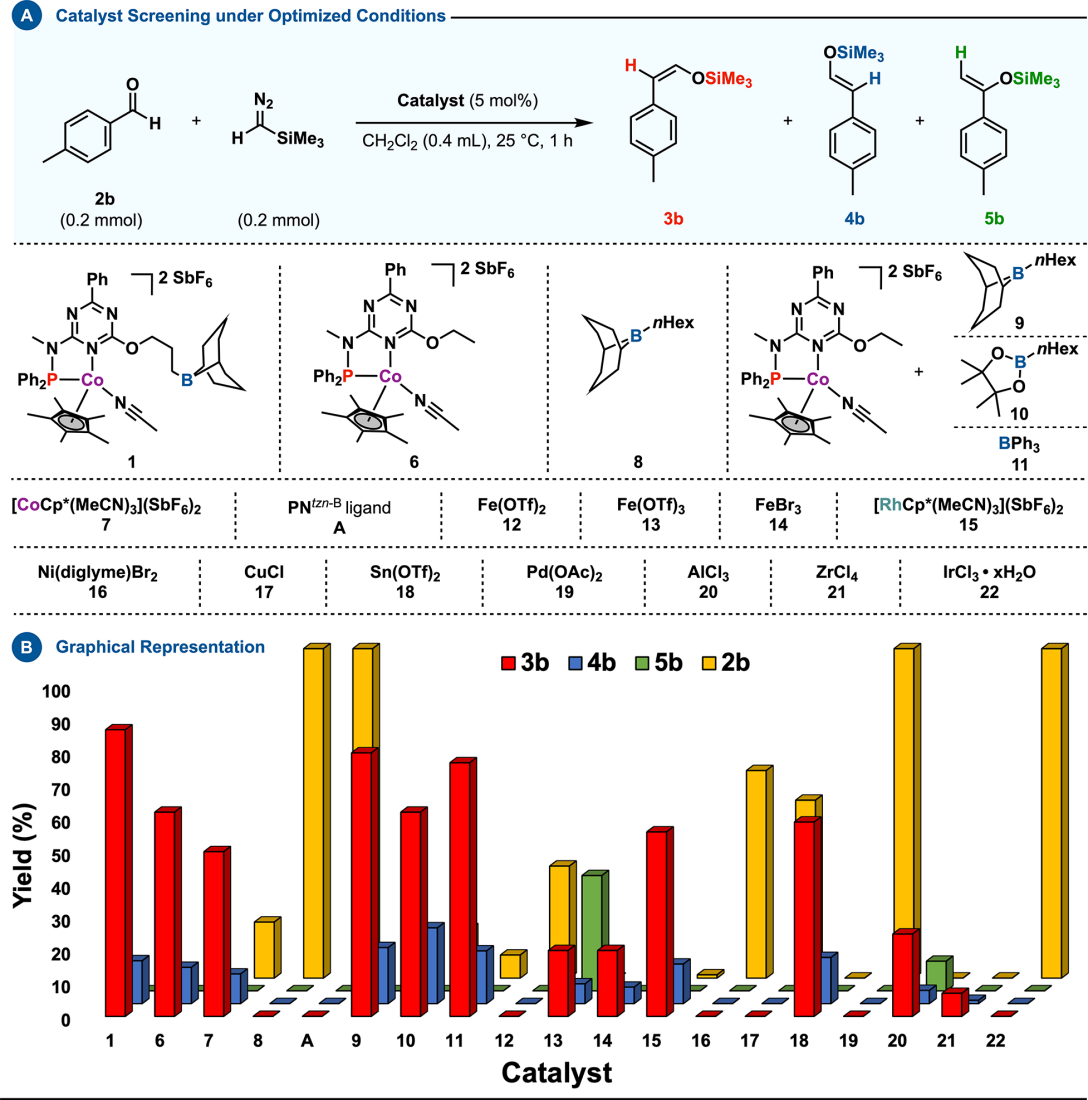
(A) Catalyst Systems Compared under Optimized Conditions; (B) Graph Illustrating Differences in Catalytic Performance

To understand the catalyst’s mechanics, action mode, and comparative performance, we conducted control experiments. These focused on the impact of the structural elements on the reaction efficiency. Initial tests compared its efficacy with analogous systems and traditional Lewis acid catalysts (Scheme 4). Results showed that, under optimized conditions, none outperformed our principal catalyst.^21^

To isolate the impact of the peripheral boron center, we created complex **6** without a boron arm and analyzed its catalytic behavior. Under standard conditions, catalyst **6** showed reduced yield and selectivity, **3b** (62%) and **4b** (11%), compared to **1**.

To expand our understanding, several intermolecular variants of compound **1** were synthesized by reacting complex **6** with exogenous boron-based additives, culminating in systems **9**–**11**. While these modifications resulted in enhanced reactivity compared to **6**, they did not reach the yield and selectivity demonstrated by **1**.

**Scheme 5:**
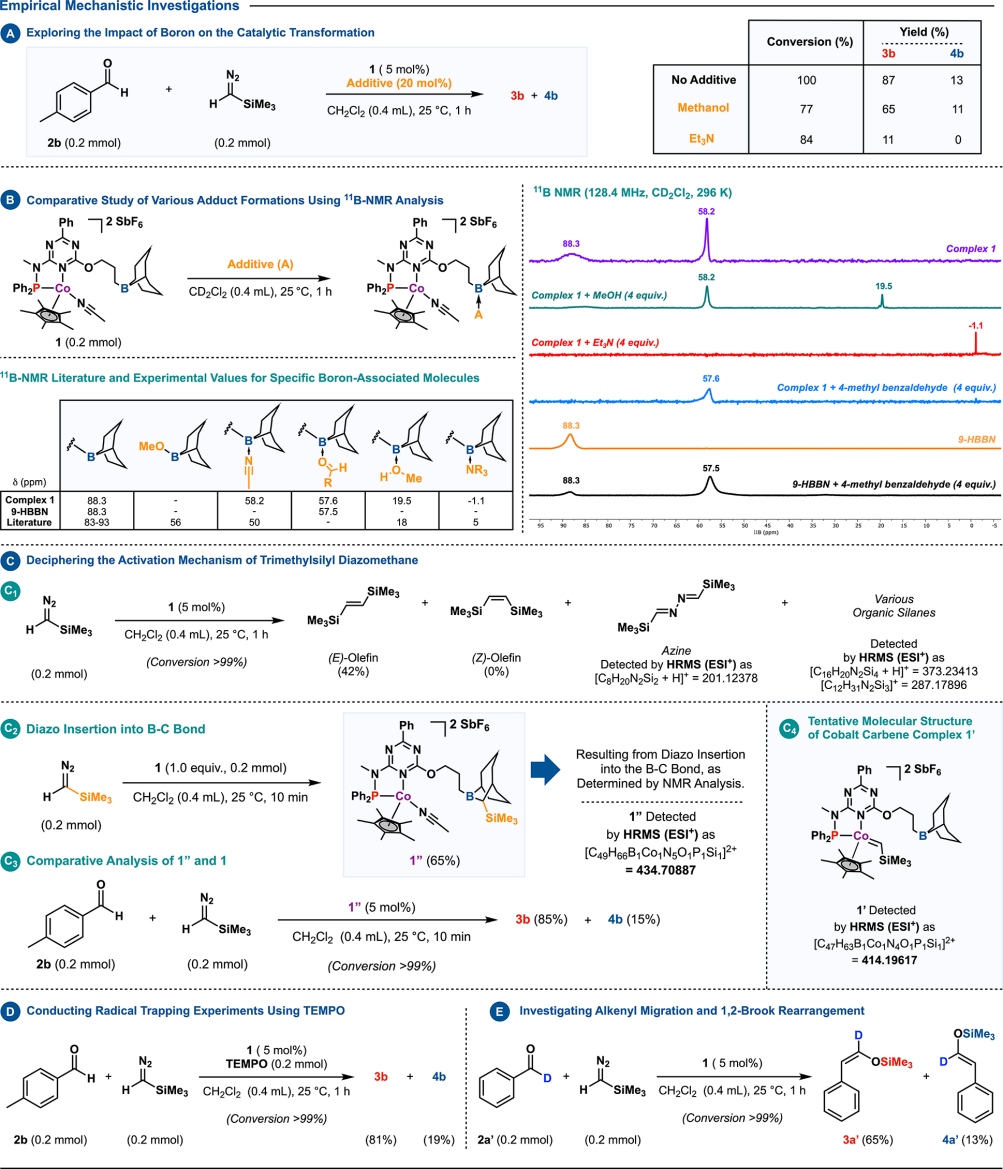
Empirical Mechanism Studies (A) Boron’s impact on catalysis. (B) Presents a comparison of adducts as characterized by ^11^B NMR spectroscopy alongside referenced literature values.^22^ (C) Activation mechanism of Me_3_SiCHN_2_. (D) Probing radicals with TEMPO. (E) Examination of alkenyl migration and 1,2-Brook rearrangement.

Boron’s impact on catalysis was studied using boron-binding additives like NEt_3_ and MeOH. These additives lowered the conversion and yield of **3b** compared to **1** (Scheme 5A).^15a^^11^B NMR studies supported boron’s ability to interact with, bind with, and potentially activate substrates. This was evidenced by the observed new chemical shifts in the presence of MeOH or NEt_3_ (Scheme 5B).^23,22e,4d^ Importantly, in a CD_2_Cl_2_ solution with compound **1** and 4-methyl benzaldehyde, a unique ^11^B resonance at δ 57.6 ppm appeared, similar to the signal observed at δ 57.5 ppm when the aldehyde was combined with 9-HBBN, suggesting carbonyl activation at the boron site. Incorporating Me_3_SiCHN_2_ into the mixture containing **1** eliminated the original resonance and unveiled (*Z*)-silyl enol ether signals in the ^1^H NMR spectrum.^24^

Subsequent investigations were directed toward unraveling the fundamental mechanism at play. Historically, studies into one-carbon homologation processes have hinted at the formation of a metal–carbene complex post-N_2_ extrusion^25^ or, as an alternative, the generation of metal-bound diazo complexes.^26^ An observation of particular note was the instantaneous release of nitrogen gas upon the stepwise addition of Me_3_SiCHN_2_ to complex **1**. Remarkably, this phenomenon was consistent even at profoundly low temperatures, such as −80 °C. Motivated by this finding, an array of studies was undertaken to delve into the solution’s internal dynamics and to pinpoint possible intermediates of the metal catalyst. The methodology involved executing several reactions between catalyst **1** and the diazoalkane under both ambient and reduced temperatures.^27^ Subsequent ^1^H NMR analysis evidenced the complete depletion of Me_3_SiCHN_2_, unmasking organic derivatives such as the homocoupled alkene, azine, and various compounds enriched with nitrogen and silicon (Scheme 5C_1_). While these derivatives do not conclusively point toward metal carbene synthesis, their properties are suggestive of carbene-mimetic reactivity.^28^

Attempts to fully characterize any carbene-related intermediate via NMR spectroscopy largely proved futile given the highly reactive nature of the metal-based species generated. Nevertheless, the nearest structural approximation at this stage was derived from the stoichiometric reaction between compounds **1** and Me_3_SiCHN_2_, culminating in the identification of a novel species, denoted as **1″** (Scheme 5C_2_–C_3_; see the SI for details).^29^ These observations were in accordance with existing literature, indicating the potential for the −CHSiMe_3_ fragment from diazomethane to integrate into the B–C bonds of the 9-BBN ring.^30a,16a,30b^

The most compelling evidence of metal–carbene formation was gleaned when a −60 °C sample, a mixture of complex **1** and diazoalkane incorporated shortly prior to the procedure, was swiftly introduced into a mass spectrometer. This analysis revealed a high-resolution molecular mass consistent with the anticipated cobalt carbene (Scheme 5C_4_). Further, when juxtaposing the molecular structure of complex **1** with other cobalt complexes from the literature, it becomes clear that complex **1**, characterized by its higher oxidation state (+III), lacks the charge transfer capability observed in previously described low-coordinate, low-valent systems.^26^ This discrepancy makes the system more prone to generating carbene intermediates after nitrogen release.^31^ As a result, an end-on binding scenario, characterized by a considerable overlap between high-lying, filled metal d-orbitals and diazoalkane π*-orbitals, which would theoretically lead to charge accumulation on the diazoalkane and strengthening of metal–nitrogen and carbon–nitrogen bonds to prevent nitrogen loss, appears less probable.

Pivoting to another facet, we investigated the potential involvement of free or carbenoid radicals in the reaction pathway (Scheme 5D).^32,25d^ The method chosen was a radical trapping experiment employing the radical scavenger 2,2,6,6-tetramethylpiperidinyloxyl (TEMPO). Notably, product formation was not affected by the presence of TEMPO, which discarded this hypothesis.

In an effort to delve deeper into the reaction pathway, a deuterium labeling experiment was executed (Scheme 5E).

The experiment utilized deuterated benzaldehyde and Me_3_SiCHN_2_ under optimized conditions. The results revealed a predominant formation of the (*Z*)-[D] **3a′** isomer, accounting for 65% of the product, alongside a less prevalent (*E*)-[D] **4a′** isomer at 13%. This outcome indicates the reaction pathway likely involves aryl migration rather than hydride migration^13b^ or epoxide formation.^33,13c^

Preliminary density functional theory (DFT) computations, conducted at the RI-B3LYP/def2-svp D3BJ level^34^ with the CPCM solvent model for dichloromethane (using the ORCA 5.0 software),^35^ align with our experimental results (see the SI for details). Geometry optimization confirmed carbene **1′** as a distinct minimum on the potential energy surface. Although several potential ether and epoxy intermediates were identified, their formation was either energetically unfavorable or necessitated high activation energies, suggesting a negligible or nonexistent role in the mechanistic pathway.

Subsequently, our attention shifted to delineating the aryl migration pathway by examining the variations in potential energy associated with the modulation of pivotal carbon–carbon bond lengths; specifically, the bond between the migrating aryl carbon and the recipient CH carbon during the genesis of (*Z*)-isomer (**3b**) and (*E*)-isomer (**4b**). These investigations unveiled exothermic reaction pathways characterized by small activation barriers, thus substantiating the proposed mechanism that assigns a central role to the aryl group rearrangement.

Notably, our computational analysis indicated that the (*Z*)-isomer benefits from reduced steric hindrance between the trimethylsilyl segment and the large borane group, relative to its (*E*)-isomer counterpart, which likely affects the reaction’s stereoselectivity. Additionally, we estimated a marginal energetic preference for the (*Z*)-isomer, around 5 kJ/mol, corroborating the *cis*-product predominance observed in our experimental pursuits and offering a theoretical basis for the reaction’s observed stereoselectivity.

**Scheme 6:**
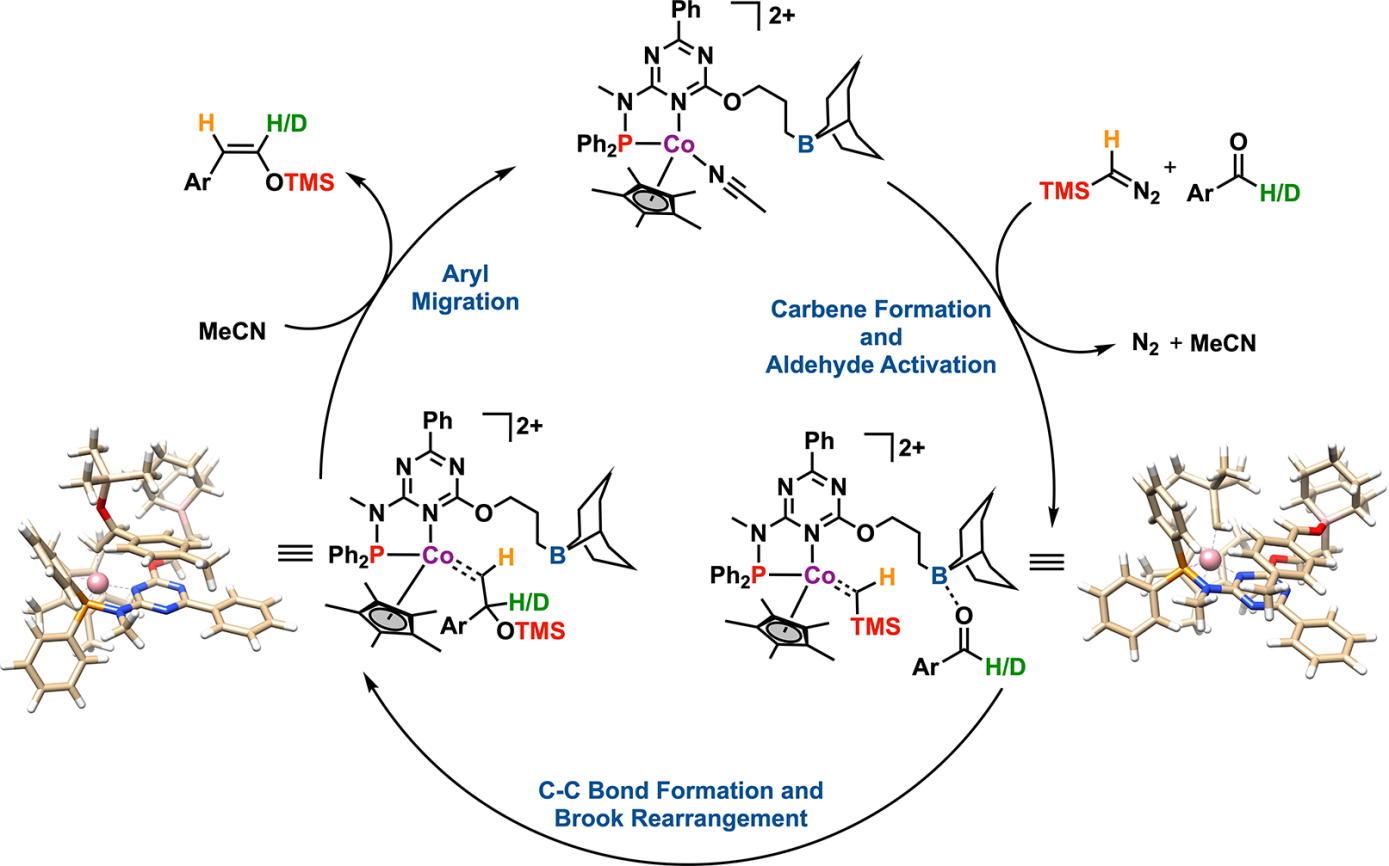
Tentative Reaction Mechanism

Integrating the information gathered thus far, we propose a mechanism for the synthesis of (*Z*)-silyl enol ethers from aromatic aldehydes, tentatively commencing with complex **1**, as illustrated in Scheme 6. This mechanistic pathway suggests that the boron atom, situated in the secondary coordination sphere, plays a pivotal role in capturing and activating the aldehyde functional group. Following the addition of Me_3_SiCHN_2_, the evidence leans toward the emergence of a reactive cobalt–carbene rather than a cobalt-diazoalkane species, a conclusion supported by the consistent observation of vigorous gas evolution and the high-valent nature of the cobalt complex. This cobalt–carbene intermediate is theorized to facilitate the incorporation of the Me_3_SiCHN_2_ carbon into the aldehyde. The reaction sequence is then thought to continue with a Brook rearrangement and culminate in aryl migration, ultimately yielding the targeted (*Z*)-silyl enol ether product.

## Conclusion

Inspired by the inherent efficacy of natural catalytic systems, we have developed a cobalt-based catalyst specifically engineered for the stereoselective incorporation of a single carbon atom into aldehydes, facilitating the synthesis of (*Z*)-silyl enol ethers. Central to the catalyst’s design is a Lewis acidic boron center, strategically integrated within a triazine ligand, which plays a pivotal role in augmented bond activation. This advancement not only positions cobalt as a viable, cost-effective substitute for the traditionally favored noble metals but also adeptly addresses multiple prevailing challenges in the synthesis of silyl enol ethers. These challenges include obstacles like regioselectivity, the necessity for large quantities of additives, limited functional group tolerance, and the requirement for extreme reaction temperatures. Our methodology distinguishes itself by operating under comparatively milder conditions and demonstrating remarkable versatility, as evidenced by its ability to process a diverse array of aromatic aldehyde substrates, each possessing various functionalities. This achievement marks a substantial leap forward in the realm of controlled bond activations within synthetic chemistry. Future work will focus on similar systems, aiming to deepen our understanding, refine our methodology, and broaden the range of achievable chemical transformations.

## Data Availability

The data that support the findings of this study (e.g., general considerations, experimental methods, synthetic details, computed structures, copies of NMR spectra, and crystallographic data) are available in the Supporting Information of this article.

## Supporting Information Available

General considerations, experimental methods, synthetic details, computed structures, copies of NMR spectra, and crystallographic data (PDF)
