# Supplementary material for: A Cooperative Cobalt-Driven System for One-Carbon Extension in the Synthesis of (Z)-Silyl Enol Ethers from Aldehydes: Unlocking Regio- and Stereoselectivity
Source: J Am Chem Soc. 2023 Dec 12;145(51):27922–32. doi: 10.1021/jacs.3c10491 (PMC10755702; doi:10.1021/jacs.3c10491)
Supplement: Supplementary file 1 — ja3c10491_si_001.pdf [file ja3c10491_si_001.pdf]

Supporting Information

A Cooperative Cobalt-Driven System for One-Carbon Extension in the Synthesis of (*Z*)-Silyl Enol Ethers from Aldehydes: Unlocking Regio- and Stereoselectivity

Soumyashree Jena,[1, 2, #] Lars Frenzen,[1, #] Vishal Chugh,[1, 2, #] Jiajun Wu,[1] Thomas Weyhermüller,[1] Alexander A. Auer,[3] and Christophe Werlé[1,2,*]

[1] Max Planck Institute for Chemical Energy Conversion, Stiftstr. 34 – 36, 45470 Mülheim an der Ruhr, Germany.

[2] Ruhr University Bochum, Universitätsstr. 150, 44801 Bochum, Germany.

[3] Max-Planck-Institut für Kohlenforschung, Kaiser-Wilhelm-Platz 1, 45470 Mülheim an der Ruhr, Germany

[#] S.J., L.F., and V.C. contributed equally to this work.

[*] Email: christophe.werle@cec.mpg.de

Table of Contents

1. Methods and Procedures S4

2. Synthesis and Characterization of Complexes S6

2.1. Synthesis of [Co(Cp*)(CH_3CN)$_3$](SbF_6)$_2$ (**7**)[1] S6

2.2. Synthesis of [CoCp*(**PN**$^{tzn\text{-}B}$)(MeCN)](SbF_6) (**1**) S8

2.3. Synthesis of [Co(Cp*)(CH_3CN)(**PN**$^{tzn\text{-}OEt}$)](SbF_6)$_2$ (**6**) S13

3. Parameter Optimization for (*Z*)-Silyl Enol Ether Synthesis S17

3.1. Solvent Selection S17

3.2. Temperature Regulation S17

3.3. Catalyst Loading S18

3.4. Reaction Duration S18

4. Protocol for (*Z*)-Silyl Enol Ether Synthesis S19

5. Empirical Mechanistic Studies S39

5.1. Catalyst Screening under Optimized Conditions S39

5.2. Investigating the Properties of the Lewis Acidic Boron Center in **1** S58

5.2.1. Catalytic Reaction in the Presence of MeOH S58

5.2.2. Catalytic Reaction in the Presence of Et_3N S59

5.3. Additive Interaction with Boron Atom in Secondary Coordination Sphere S60

5.4. Exploring the Formation of Hypothetical Metal Carbenes S61

5.5. Investigation of Alkenyl Migration and 1,2-Brook Rearrangement S63

5.6. Tempo Experiment S65

5.7. Exploring Product Formation and Intermediate Generation from Mixtures of Complex **1** and Me_3SiCHN_2 .... S66

5.7.1. Reaction of **1** with $TMSCHN_2$ S75

5.7.2. Analyzing Product Formation in the Reaction Between **1** (5 mol%) and $Me_3SiCHN_2$78

5.7.3. Probing the Impact of Reagent Addition Sequence: Commencing with Me_3SiCHN_2 and Subsequently Introducing Aldehyde S81

5.8. Control Experiments S82

5.8.1. Reaction of Aldehyde with $TMSCHN_2$ S82

5.8.2. Reaction of Aldehyde with $TMSCHN_2$ in presence of 9-HBBN S83

6. ^{1}H and ^{13}C NMR Spectral Analysis of (*Z*)-Silyl Enol Ethers S84

7. Computational study S116

7.1. Computational Details S116

7.1.1. Electronic Structure Calculations S116

7.1.2. Tentative Mechanistic Model for *Z*-Isomer Selectivity S120

7.2. xyz Coordinates of Computed Structures S121

7.2.1. Structure **ThSI1** S121

7.2.2. Structure **ThSI2** S126
7.2.3. Structure **ThSI3** S131
7.2.4. Structure **ThSI4** S136
7.2.5. Structure **ThSI5** S141
7.2.6. Structure **ThSI8** S146
8. X-ray Structural Analysis and Refinement S151
8.1. Molecular Structure of **1** (CCDC 2169090) S153
8.2. Molecular Structure of **6** (CCDC 2169092) S154
8.3. Molecular Structure of **7** (CCDC 2169091) S155
9. References S156

1. Methods and Procedures

All reactions were prepared within an MBraun glovebox in an argon atmosphere. Solvents employed in air- and moisture-sensitive experiments underwent purification through a two-column solvent purification system (MBraun-SPS-7) and were transferred directly to the glovebox for storage over molecular sieves (3 Å). Technical grade solvents were used for workup and purification procedures. All reagents, sourced from abcr, Alfa Aesar, or Sigma Aldrich at the highest commercial quality, required no further purification. Conversions and yields were determined spectroscopically *via* ^{1}H NMR with mesitylene serving as an internal standard. Reaction completion was monitored using thin-layer chromatography (TLC) with 0.25 mm Polygram SIL G/UV254 Silica Plates and short-wavelength UV light as visualizing agents, or alternatively, $KMnO_4$ and iodine vapors as developing reagents. Products were isolated through a small silica gel chromatography column (1 cm thickness) filled with high purity grade silica gel (60 Å, particle size 0.043–0.063 mm) from Sigma Aldrich. Both the silica gel and sea sand used for the column were dried at 120 ºC overnight. Column chromatography employed dried solvents, with a dry environment maintained during separation. All solution-state NMR were recorded on a Bruker Ascend 400 spectrometer in the specified solvents at the given temperatures. The coupling constants (J) are given in Hertz (Hz). The chemical shifts (δ) expressed in ppm are calibrated using residual proton peaks in the solvent ($CDCl_3$, C_6D_6, Toluene-d_8, CD_3CN at 7.26, 7.16, 2.09, 1.94 ppm for ^{1}H NMR, respectively, and 77.16, 128.06, 20.43, 1.32 ppm for ^{13}C{^{1}H} NMR, respectively). Abbreviations were used to denote multiplicities: s = singlet, d = doublet, dd = doublet of doublet, t = triplet, q = quartet, tt = triplet of triplet, m = multiplet, bs = broad singlet. Gas chromatography (GC) was performed on a Shimadzu GC2030 equipped with an FID-detector (CP-WAX-52CB column from Agilent), and gas chromatography-mass spectrometry (GC-MS) was conducted on a Shimadzu QP2020. HR-MS spectra were recorded using a Bruker ESQ3000 spectrometer. Infrared spectra were recorded on a Thermo Scientific Nicolet™ iS5 Spectrometer with an ID7 ATR accessory. The IR signals were categorized as strong (st), medium (m), or weak (w). Elemental analyses (carbon, hydrogen, and nitrogen) were carried out using an Elementar UNICUBE elemental analyzer.

Disclaimers:

Caution: When conducting experiments involving potentially explosive diazo compounds, appropriate safety procedures must be followed, and suitable equipment must be used. Protective measures, including the use of gloves and a shield within a fume hood, are necessary.

Please note that slight variations in catalytic outcomes may emerge based on the source, supplier, preparation, and storage conditions of Me_3SiCHN_2*. Such variations arise due to potential disparities in the molar concentration of* Me_3SiCHN_2 *in solution. These disparities might be represented as suppliers may provide an approximate concentration or a concentration range rather than a precise value. Furthermore, to achieve consistent and optimal results, it is advisable to employ diazo reagents that have been recently procured. This is because aged samples are prone to thermal decomposition. Strict adherence to the outlined protocol will minimize these variations, ensuring the reproducibility and robustness of experimental results.*

2. Synthesis and Characterization of Complexes

2.1. Synthesis of [Co(Cp*)(CH_3CN)$_3$](SbF_6)$_2$ (7)[1]

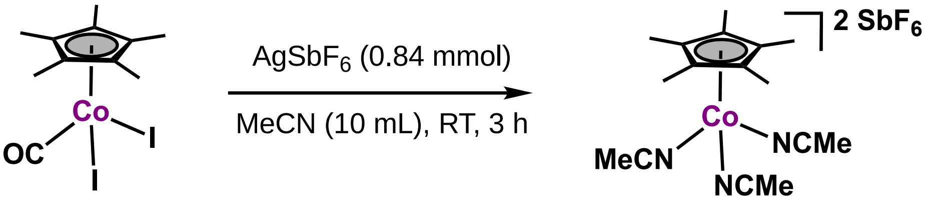

(0.42 mmol) **7** (90%)

Procedure: An oven-dried Schlenk tube was charged with Co(Cp*)(CO)I_2 (200 mg, 0.42 mmol) and $AgSbF_6$ (288.64 mg, 0.84 mmol) in acetonitrile (10 ml). This mixture was stirred in the dark for 3 hours, leading to the immediate formation of AgI precipitate. Stirring continued for an additional hour at room temperature. Subsequently, the reaction was halted, and all the AgI was filtered off with a syringe filter. Solvents were then removed in vacuo, resulting in the precipitation of a dark purple solid. This precipitate was washed with pentane (3 × 5 mL) and dried *in vacuo* to yield compound **7** as a dark purple solid (298 mg, 0.38 mmol, 90%). Crystals suitable for X-ray diffraction were obtained by layering a concentrated CH_2Cl_2 solution (0.4 mL) of compound **7** with pentane (4 mL).

^{1}H NMR (400 MHz, CD_3CN, 296 K): δ 1.95 (s, 9H), 1.39 (s, 15H)

^{13}C NMR (101 MHz, CD_3CN, 296 K): δ 117.93, 101.43, 9.91

Anal Calcd (%): ($C_{16}H_{24}CoF_{12}N_3Sb_2$ + 0.2 $AgSbF_6$): C 21.97, H 2.77, N 4.80; Found: C 21.75, H 2.67, N 4.85.

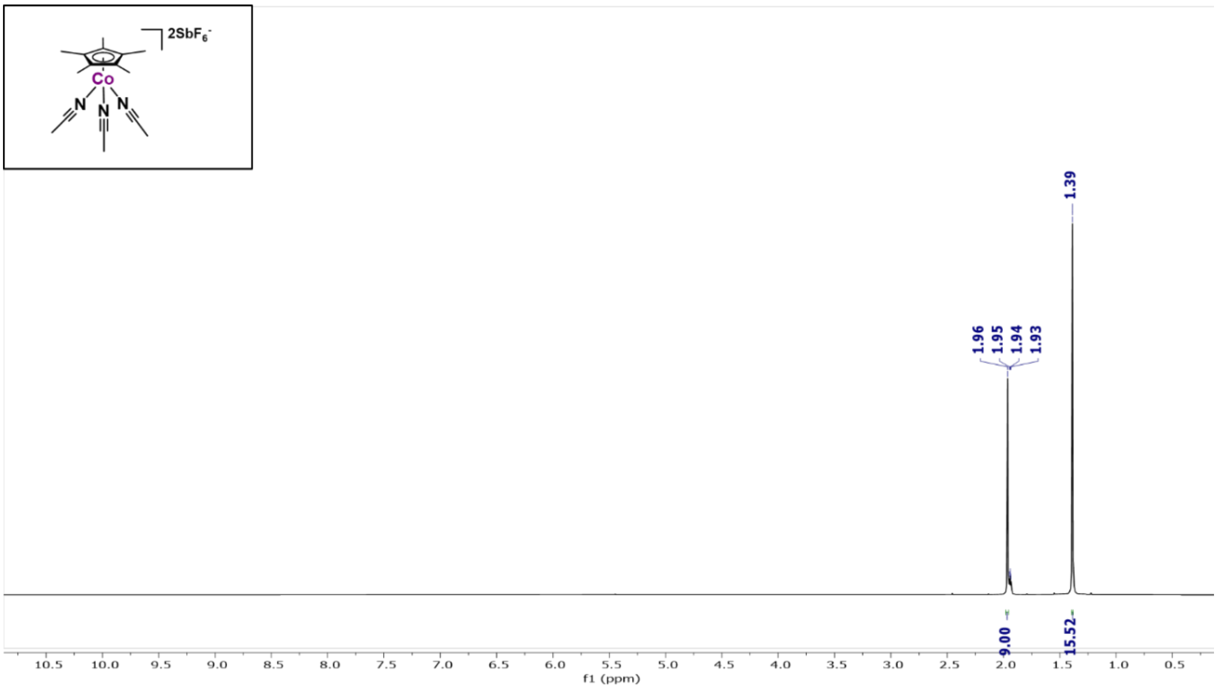

Figure S1 – ^{1}H NMR (400 MHz, CD_3CN, 293 K) spectrum of **7**.

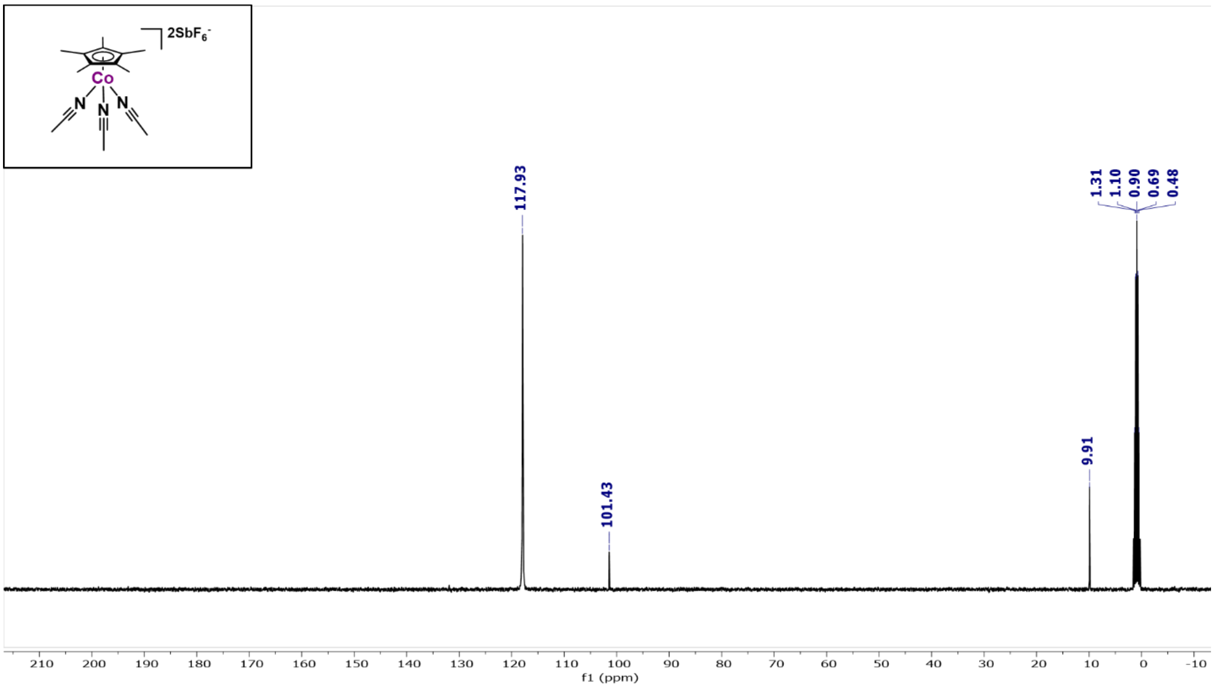

Figure S2 – ^{13}C{^{1}H} NMR (101 MHz, CD_3CN, 293 K) spectrum of **7**.

2.2. Synthesis of [CoCp*(PN$^{tzn\text{-}B}$)(MeCN)](SbF_6) (1)

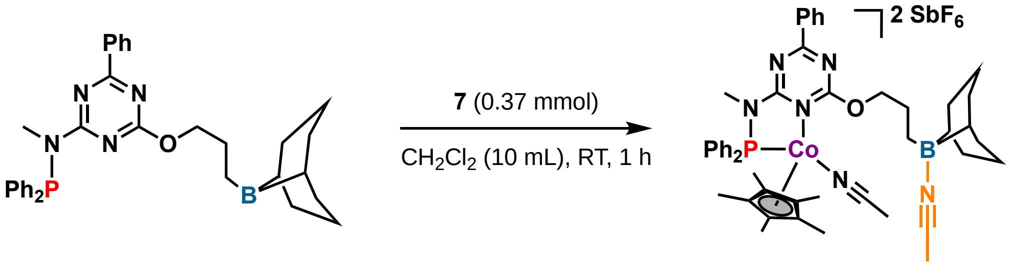

A (0.37 mmol) **1** • CH_3CN (82%)

Procedure: Under an argon atmosphere, an oven-dried Schlenk tube was loaded with a mixture of ligand **A**[2] (202.90 mg, 0.37 mmol) and [Cp*Co(MeCN)$_3$](SbF_6)$_2$ (300 mg, 0.37 mmol) in CH_2Cl_2 (10 mL). This mixture was stirred at room temperature for 1 hour, during which the solution's color changed from pink to dark red, indicating the end of the reaction. The resulting dark red solution was then concentrated *in vacuo*. The precipitated solid was washed with pentane (3 × 5 mL) and dried under vacuum to yield compound **1** as a dark red solid (402.7 mg, 0.30 mmol, 82%). Crystals suitable for X-ray diffraction were obtained by layering a concentrated CH_3CN solution (0.4 mL) of complex **1** with Et_2O (4 mL).

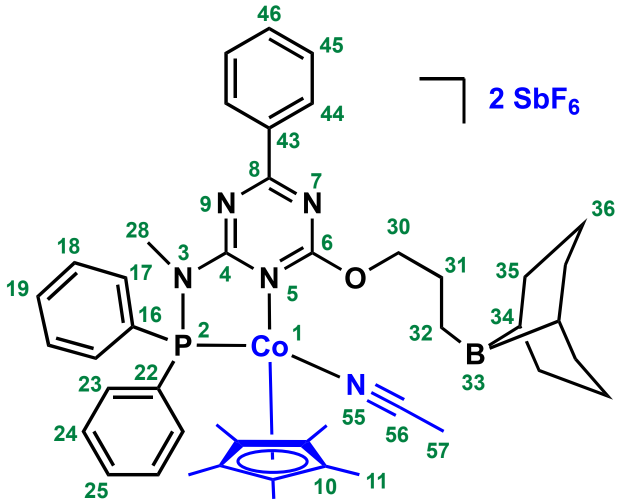

^{1}H NMR (400 MHz, CD_2Cl_2, 296 K): δ 8.60 – 8.45 (m, 2H, 44), 7.82 – 7.68 (m, 4H, 17, 23), 7.67 – 7.58 (m, 4H, 18, 24), 7.50 (t, *J* = 7.8 Hz, 3H, 45, 46), 7.43 – 7.37 (m, 2H, 19, 25), 5.26 – 5.14 (m, 1H, 30), 4.55 (dt, *J* = 10.6, 7.9 Hz, 1H, 30), 3.34 (d, *J* = 5.1 Hz, 3H, 28), 2.08 (p, *J* = 7.9 Hz, 2H, 31), 1.85 – 1.64 (m, 12H, 35, 36,), 1.63 (d, *J* = 1.1 Hz, 3H, 57), 1.48 – 1.41 (m, 2H, 32), 1.23 (d, *J* = 2.5 Hz, 15H, 11), 1.19 – 1.13 (m, 2H, 34).

$^{13}C\{^1H\}$ NMR (101 MHz, CD_2Cl_2, 296 K): δ 174.6 , 8, 171.9 (d, J = 22.1 Hz, 4), 170.1 (d, J = 3.2 Hz, 6), 134.9, 134.7 , 46, 133.7, 133.2 , 43, 132.9 , 16, 22, 132.5 (d, J = 7.9 Hz, 17), 131.8, 130.5 (t, J = 11.0 Hz, 18, 24), 130.1 (44), 129.8 (d, J = 11.6 Hz), 129.4, 128.8 (45), 126.9 (d, J = 45.4 Hz, 56), 121.5, 102.6 (d, J = 1.9 Hz, 10), 74.0 (30), 37.3 (d, J = 4.9 Hz, 28), 33.1 (35), 31.1 (34), 23.8 (31), 23.1 (36), 22.3 (32), 10.0 (11), 2.7 (57).

$^{31}P\{^1H\}$ NMR (162 MHz, CD_2Cl_2, 296 K): δ 114.25 ppm.

^{11}B NMR (126 MHz, CD_2Cl_2, 296 K): δ 88.08 ppm, 58.17 ppm (**1**•CH_3CN).

HRMS (ESI$^+$): Calcd. for $C_{45}H_{56}BCoN_5OP$ [M – CH_3CN]$^{2+}$: 391.6818; Found 391.6817.

Anal Calcd (%): ($C_{47}H_{59}BCoF_{12}N_6OPSb_2$ + 0.5 C_5H_{12}): C 44.62, H 4.92, N 6.31; Found: C 44.37, H 4.91, N 6.45.

IR (Diamond – ATR, neat), υ (cm^{-1}): 2881.21 (w), 1566.51 (st), 1473.80 (m), 1434.49 (m), 1376.65 (m), 1351.02 (m), 1158.06 (m), 1097.43 (m), 1012.71 (w), 942.26 (w), 821.56 (w), 783.18 (m), 749.88 (m), 696.91 (m), 652.90 (st), 556.92 (m).

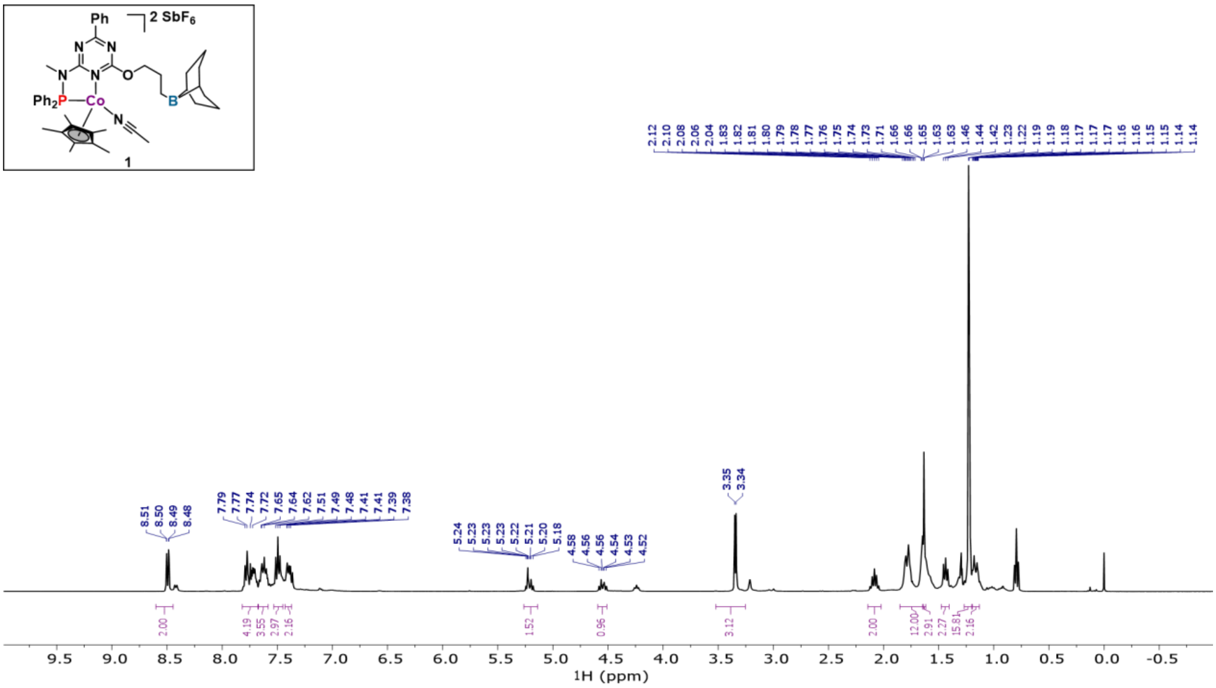

Figure S3 – **^1H NMR (400 MHz, CD_2Cl_2, 293 K)** spectrum of complex **1**.

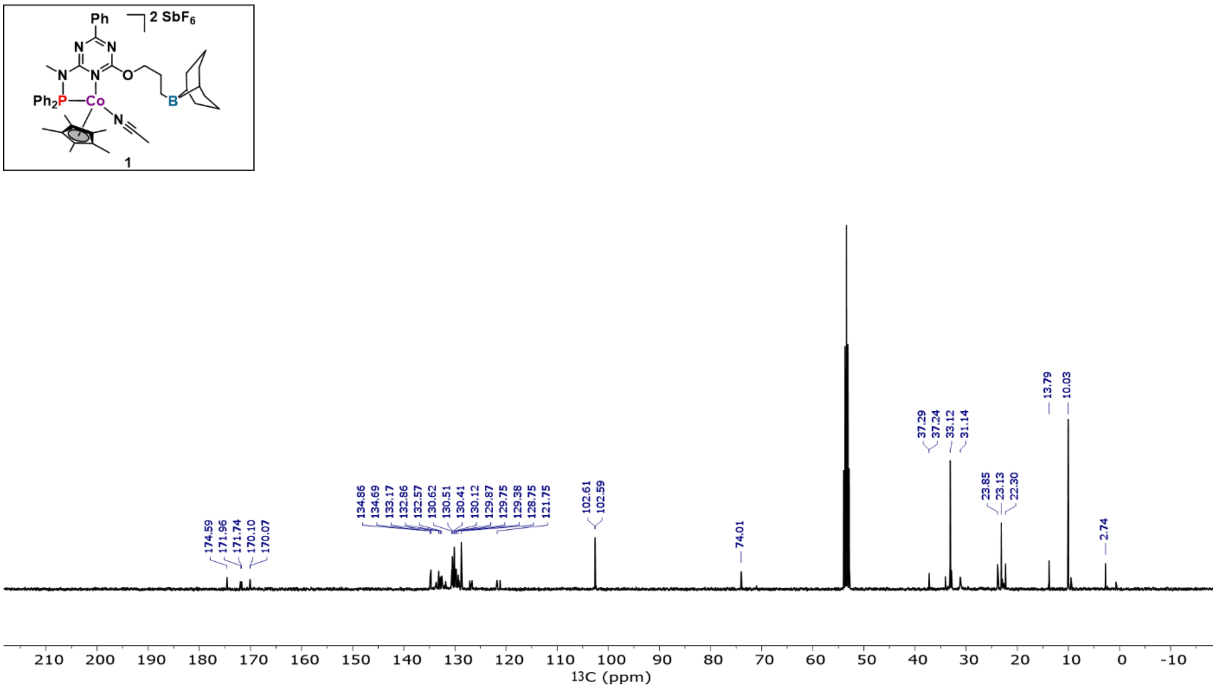

Figure S4 – **$^{13}C\{^{1}H\}$ NMR (101 MHz, CD_2Cl_2, 293 K)** spectrum of complex **1**.

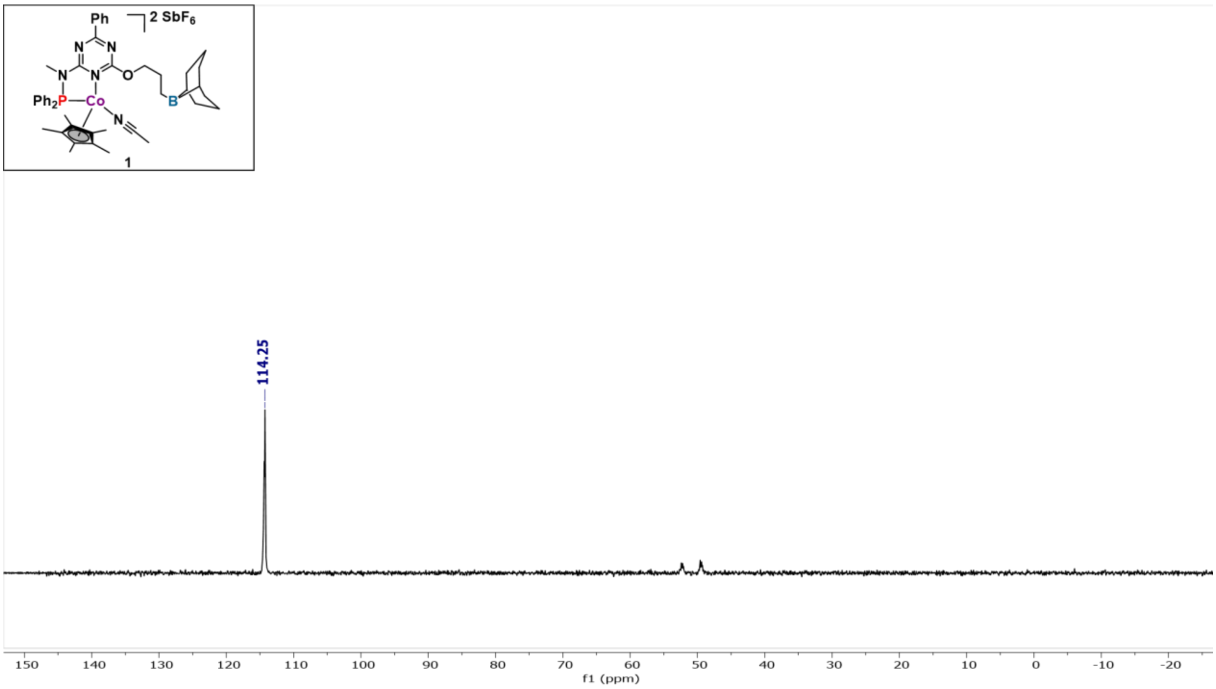

Figure S5 – **$^{31}P\{^{1}H\}$ NMR (162 MHz, CD_2Cl_2, 293 K)** spectrum of complex **1**.

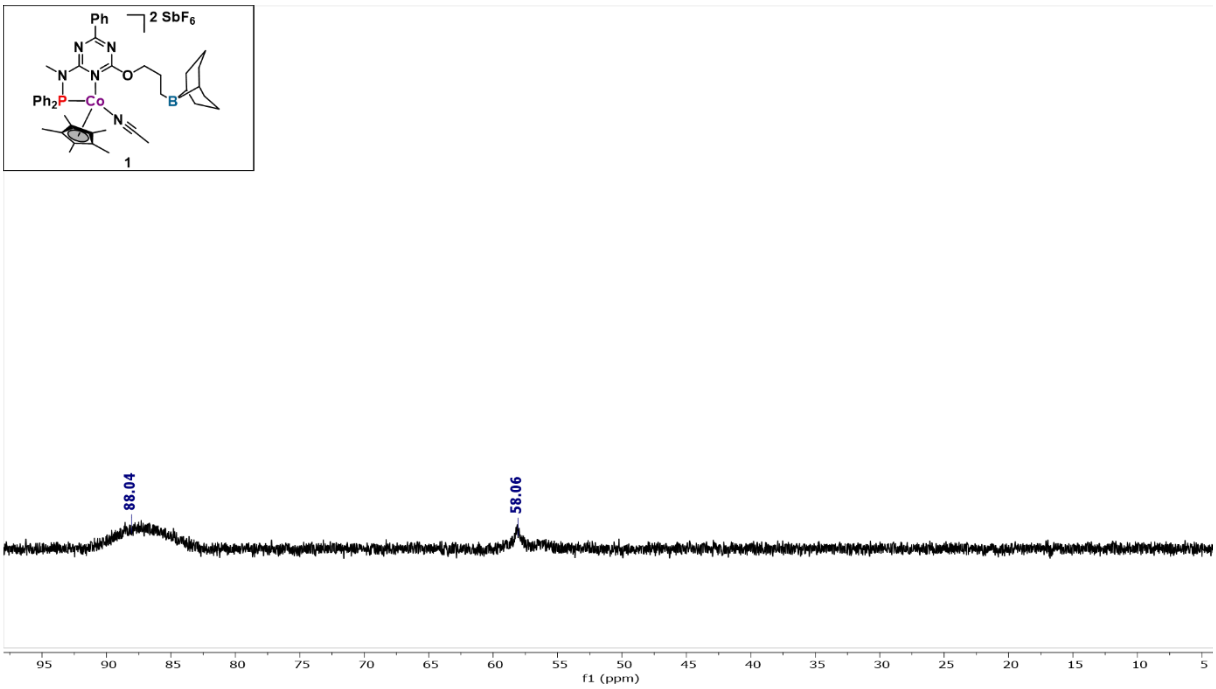

Figure S6 – **^{11}B NMR (126 MHz, CD_2Cl_2, 293 K)** spectrum of complex **1**.

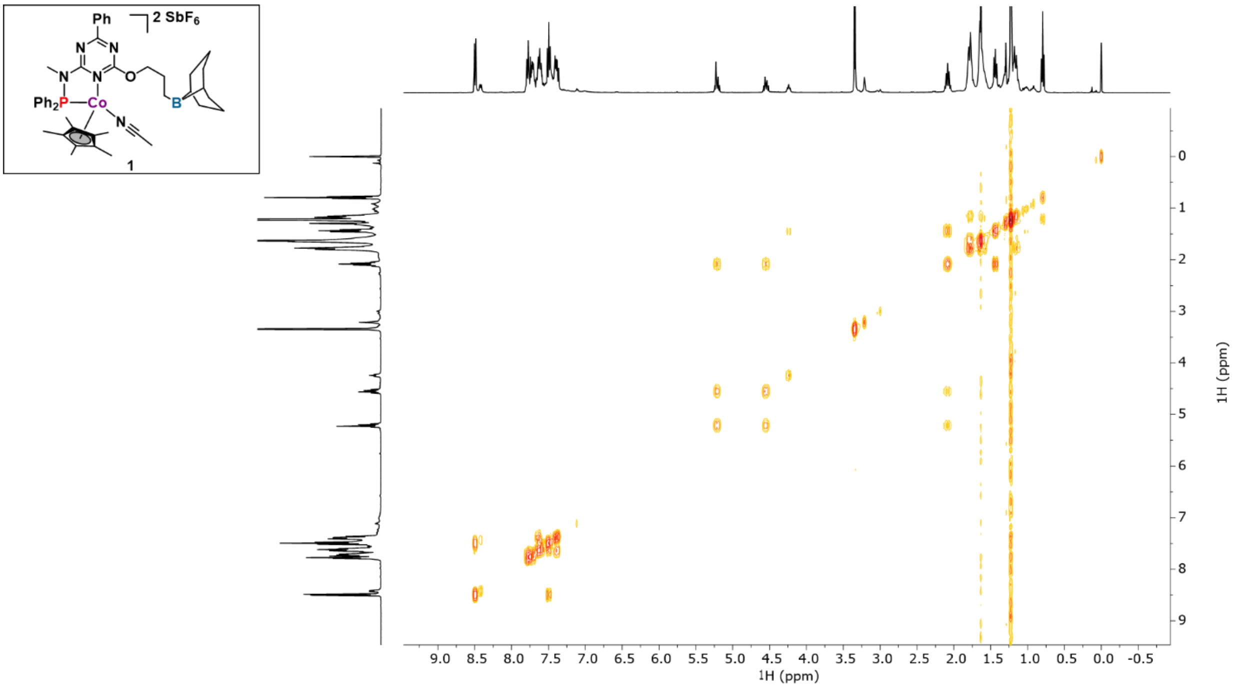

Figure S7 – **^{1}H-^{1}H COSY NMR (400 MHz, CD_2Cl_2, 293 K)** spectrum of complex **1**.

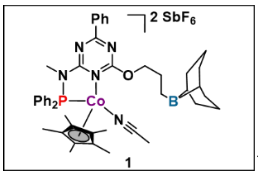

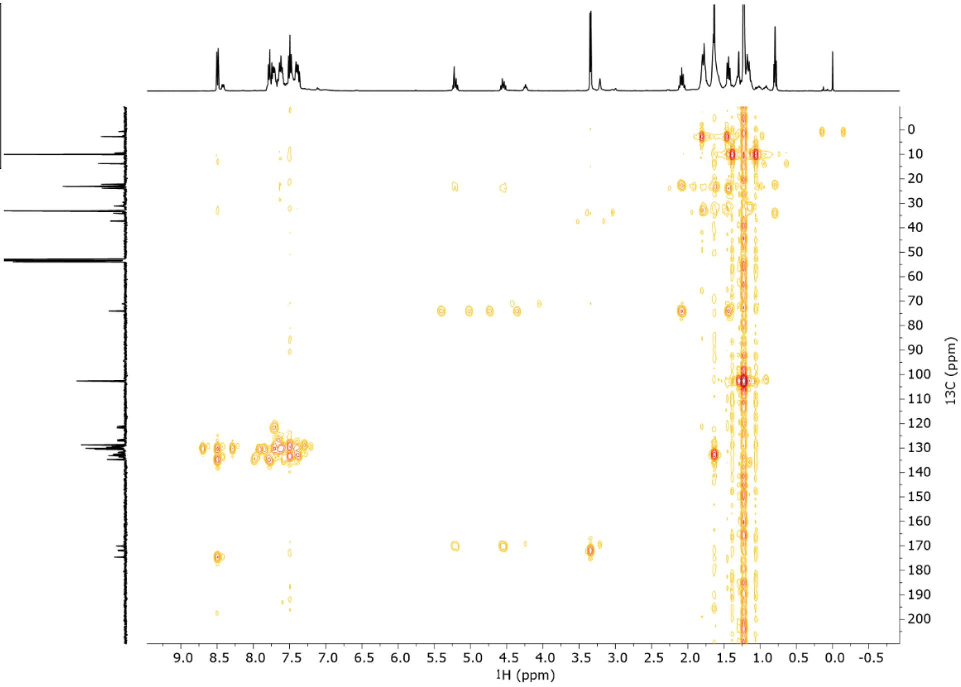

Figure S8 – **^{1}H-^{13}C HMBC NMR (400, 101 MHz, CD_2Cl_2, 293 K)** spectrum of complex **1**.

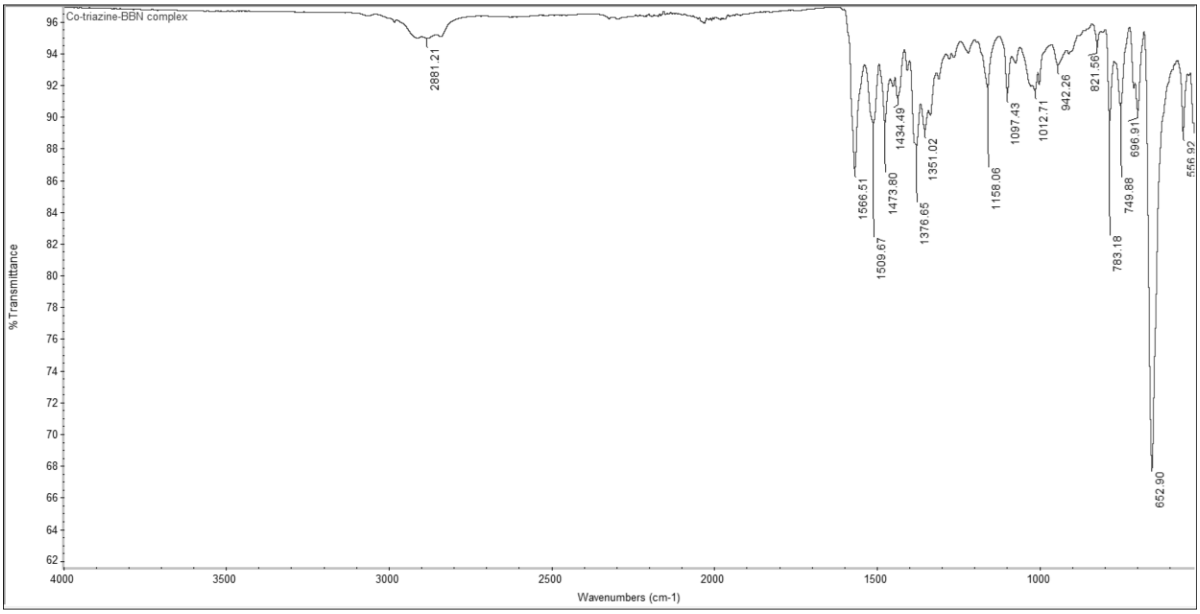

Figure S9 – **IR (Diamond – ATR, neat)** spectrum of complex **1**.

2.3. Synthesis of $[Co(Cp^*)(CH_3CN)(PN^{tzn\text{-}OEt})](SbF_6)_2$ (6)

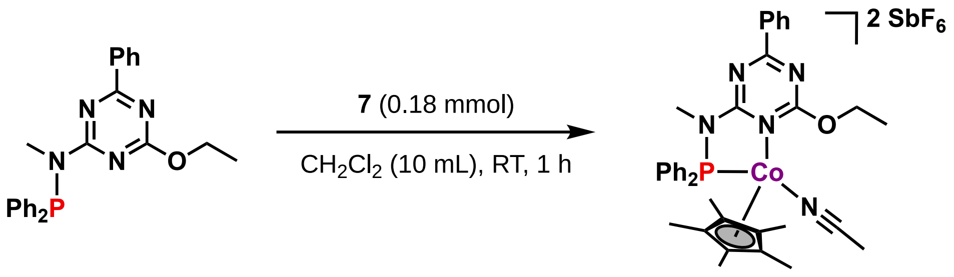

B (0.18 mmol) **6** (78%)

Procedure: An oven-dried Schlenk tube was charged with ligand **B**[2] (83.25 mg, 0.18 mmol) and $[Cp^*Co(MeCN)_3](SbF_6)_2$ (150 mg, 0.18 mmol) in CH_2Cl_2 (10 mL). The resulting mixture was stirred at room temperature for 1 hour. The color of the solution changed from pink to dark red as the reaction proceeded. Following this period, the solution was concentrated *in vacuo* to remove all solvents. Pentane (5 mL) was added to induce the formation of a dark red precipitate. The precipitated solid was subsequently washed with pentane (2 × 5 mL) and dried under vacuum to yield compound **6** as a dark red solid (159.5 mg, 0.14 mmol, 78%). Crystals suitable for X-ray diffraction were obtained by layering a concentrated CH_2Cl_2 solution (0.4 mL) of compound **6** with pentane (4 mL), or simply by leaving the concentrated mixture in CH_2Cl_2-d_2 at room temperature overnight.

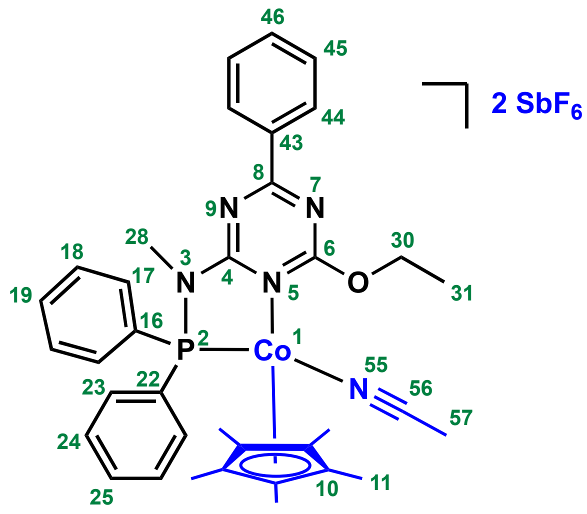

^{1}H NMR (400 MHz, CD_2Cl_2, 296 K): δ 8.55 – 8.44 (m, 2H, 44), 7.82 – 7.67 (m, 5H, 17, 23), 7.66 – 7.54 (m, 4H, 18, 24), 7.50 (dd, J = 8.4, 7.1 Hz, 2H, 45), 7.36 (ddt, J = 12.1, 5.8, 1.6 Hz, 2H, 19, 25), 5.29 – 5.16 (m, 1H, 30), 4.70 (dq, J = 10.9, 7.1 Hz, 1H), 3.33 (d, J = 5.2 Hz, 3H, 28), 1.49 (t, J = 7.0 Hz, 3H, 31), 1.21 (d, J = 2.5 Hz, 15H, 11).

^{13}C{^{1}H} NMR (101 MHz, CD_2Cl_2, 296 K): δ 175.2 (8), 172.5 (d, J = 22.1 Hz, 4), 170.6 (d, J = 3.2 Hz, 6), 135.4 (d, J = 3.1 Hz, 16), 135.3 (46), 134.4 (19, 25), 133.8 (43), 133.4 (d, J = 3.0 Hz, 22), 131.1 (dd, J = 13.6, 11.6 Hz, 17, 23), 130.7 (44), 130.4 (d, J = 11.6 Hz, 18, 24), 129.4 (45), 127.5 (d, J = 45.1 Hz, 56), 103.2 (d, J = 2.0 Hz, 10), 68.8 (30), 37.8 (d, J = 4.9 Hz, 28), 14.8 (31), 10.6 (11), 1.3 (57)

$^{31}P\{^{1}H\}$ NMR (162 MHz, CD_2Cl_2, 296 K): δ 114.45 ppm.

IR (Diamond – ATR, neat), υ (cm^{-1}): 558.09 (s), 654.81 (w), 698.63 (s), 735.60 (s), 784.(s), 822.12 (s), 944.31 (s), 999.43 (s), 1015.51 (s), 1073.02 (s),1098.24 (s), 1159.42 (s), 1218.14 (s), 1264.83 (s), 1336.85 (m), 1358.68 (m), 1384.39 (w), 1435.64 (m), 1474.52 (m), 1511.21 (m), 1566.77 (w), 2919.28 (s)

HRMS (ESI^+): Calcd. for $C_{34}H_{38}CoN_4OP$ [M – CH_3CN]$^{2+}$: 304.10521; Found 304.10483.

Anal Calcd (%): ($C_{36}H_{42}CoF_{12}N_5OPSb_2$): C 38.53, H 3.77, N 6.24; Found C 39.10, H 3.74, N 6.24

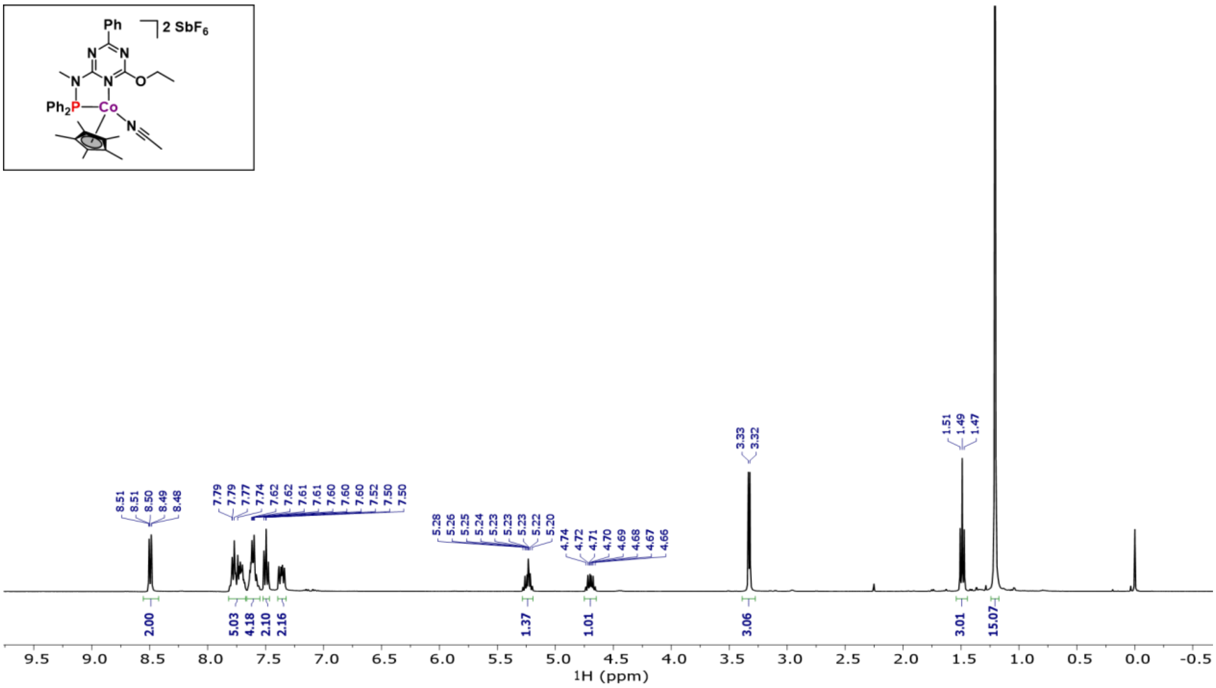

Figure S10 – **^{1}H NMR (400 MHz, CD_2Cl_2, 293 K)** spectrum of complex **6**.

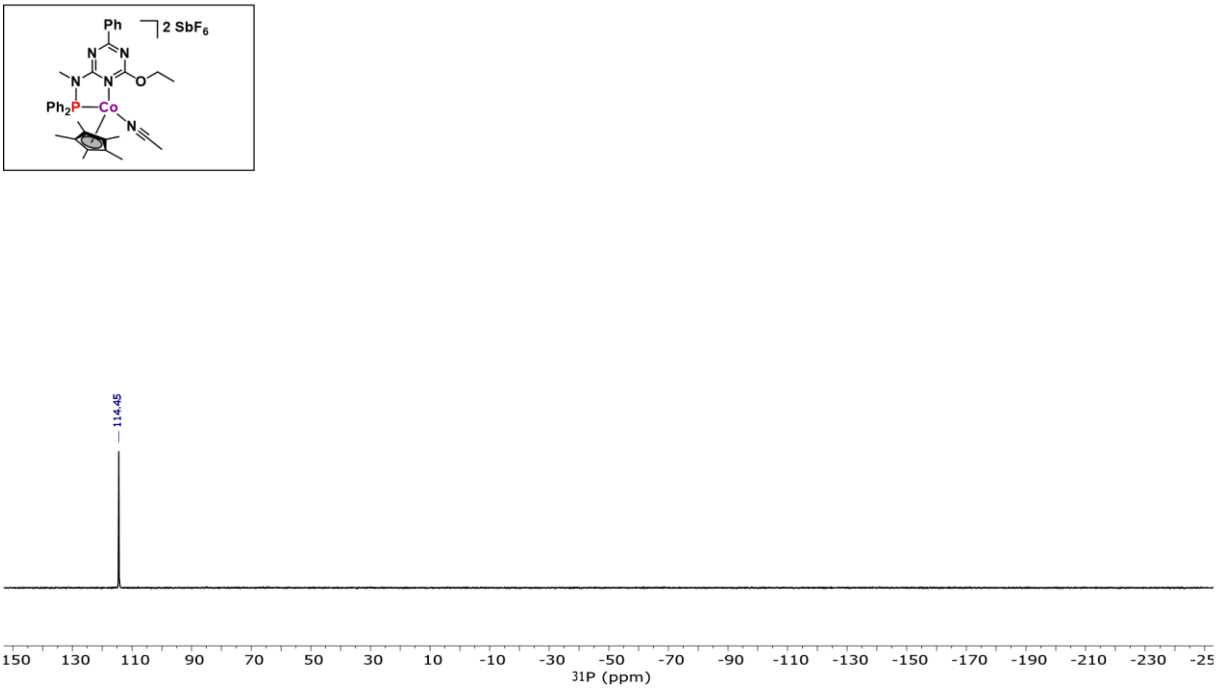

Figure S11 – **$^{31}P\{^{1}H\}$ NMR (162 MHz, CD_2Cl_2, 293 K)** spectrum of complex **6**.

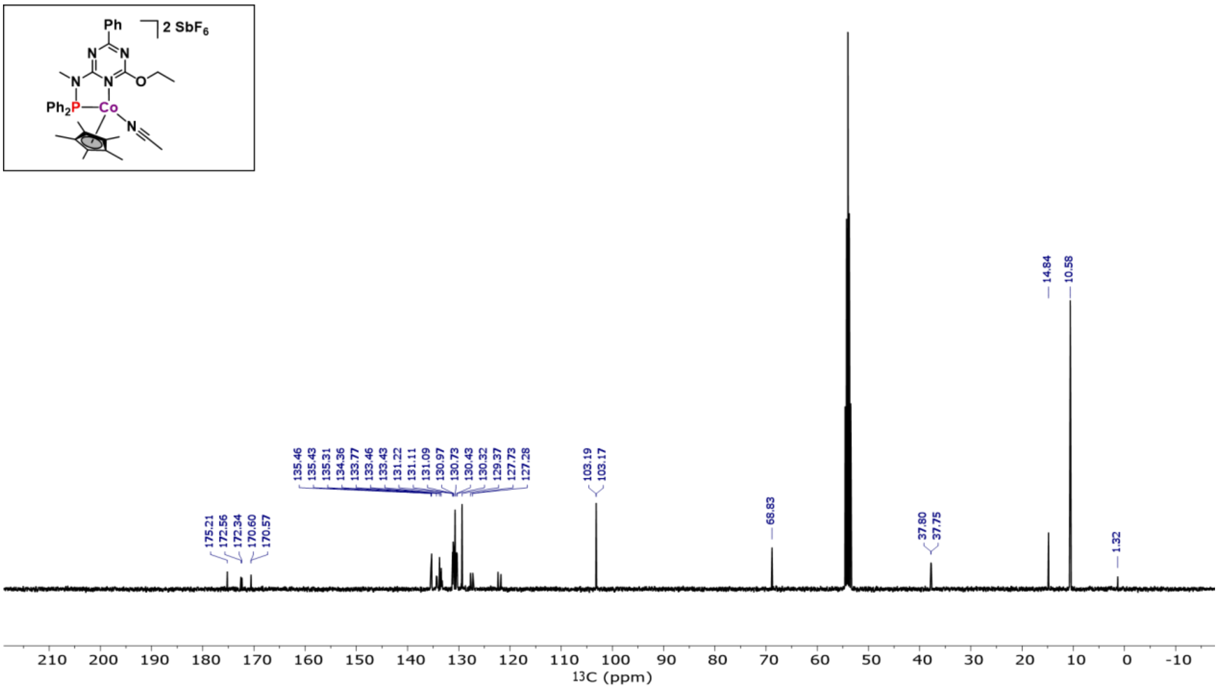

Figure S12 – **$^{13}C\{^{1}H\}$ NMR (101 MHz, CD_2Cl_2, 293 K)** spectrum of complex **6**.

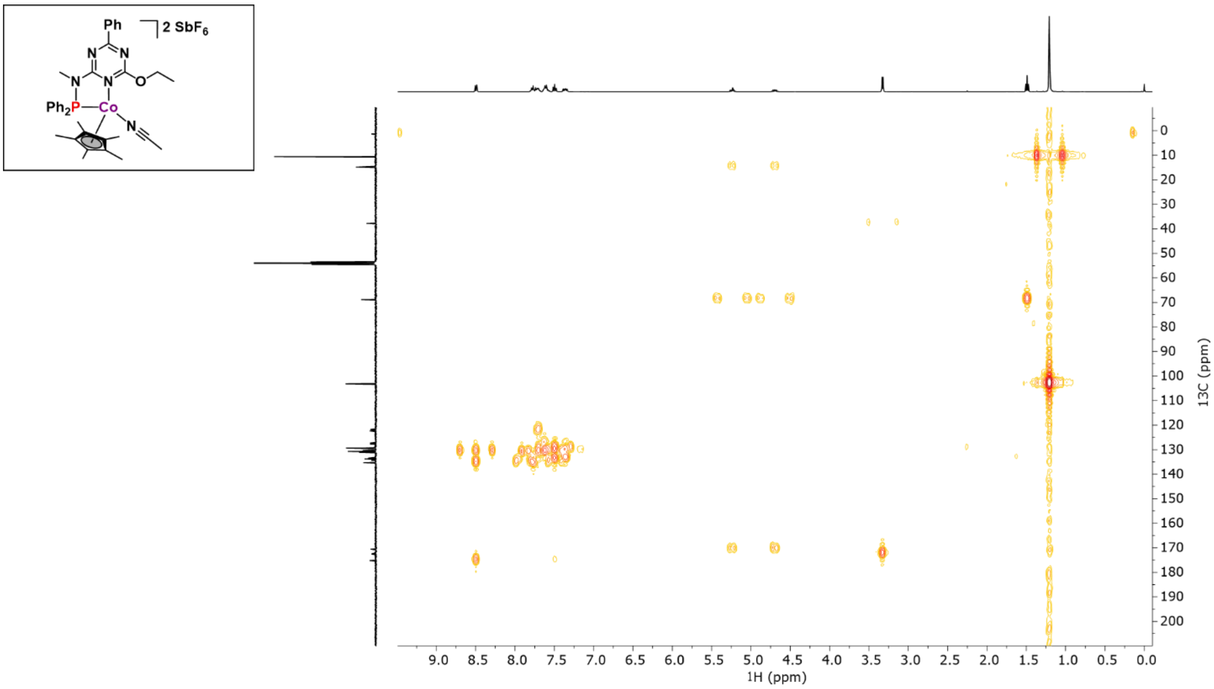

Figure S13 – **^{1}H-^{13}C HMBC NMR (400, 101 MHz, CD_2Cl_2, 293 K)** spectrum of complex **6**.

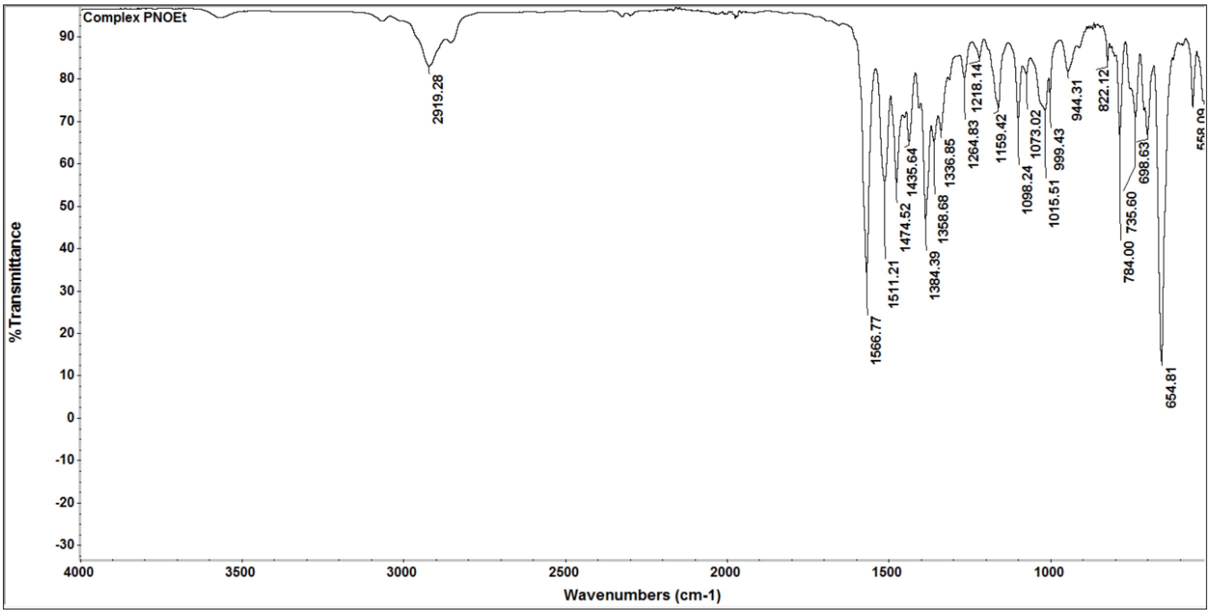

Figure S14 – **IR (Diamond – ATR, neat)** spectrum of complex **6**.

3. Parameter Optimization for (*Z*)-Silyl Enol Ether Synthesis

3.1. Solvent Selection

Table S1 – Optimization of the reaction condition for (*Z*)-silyl enol ethers: **Solvent**. [a]Yields are based on ^{1}H NMR relative to mesitylene (0.2 mmol) as an internal standard.

Solvent Screening

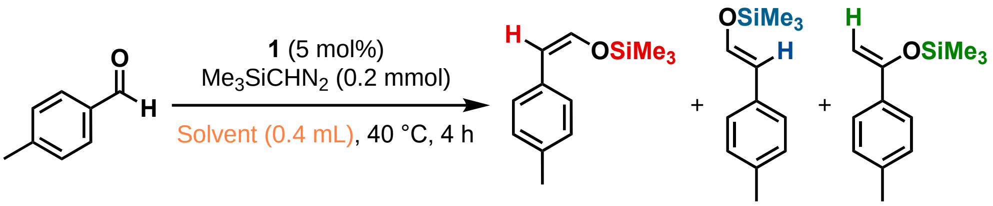

0.2 mmol — **3b** — **4b** — **5b**

Entry	Deviation from above	Yields (%)[a] **3b**:**4b**:**5b**
1	THF	82:09:00
**2**	**CH_2Cl_2**	**87:13:00**
3	CH_3CN	76:14:00
4	Toluene	69:11:02
5	Mesitylene	62:10:06
6	1,4 - Dioxane	76:16:06

3.2. Temperature Regulation

Table S2 – Optimization of the reaction condition for (*Z*)-silyl enol ethers: **Temperature**. [a]Yields are based on ^{1}H NMR relative to mesitylene (0.2 mmol) as an internal standard.

Temperature Screening

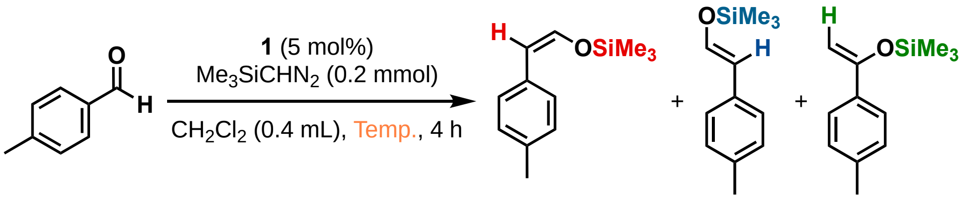

0.2 mmol — **3b** — **4b** — **5b**

Entry	Deviation from above	Yields (%)[a] **3b**:**4b**:**5b**
1	40 °C	87:13:00
**2**	**RT**	**86:13:00**
3	0 °C	82:13:00
4	-50 °C	80:11:00

3.3. Catalyst Loading

Table S3– Optimization of the reaction condition for (*Z*)-silyl enol ethers: **Catalyst loading**. [a]Yields are based on ^{1}H NMR relative to mesitylene (0.2 mmol) as an internal standard.

Screening of Catalyst Loading

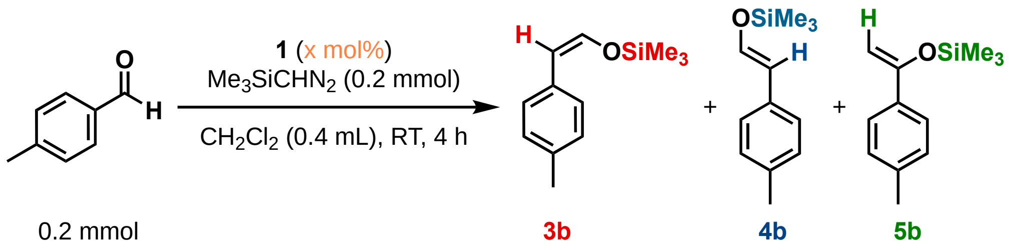

Entry	Deviation from above	Yields (%)[a] 3b:4b:5b
1	0.5 mol%	76:13:03
2	1.0 mol%	70:13:06
3	2.5 mol%	77:13:02
**4**	**5.0 mol%**	**87:13:00**

3.4. Reaction Duration

Table S4– Optimization of the reaction condition for (*Z*)-silyl enol ethers: **Reaction Time**. [a]Yields are based on ^{1}H NMR relative to mesitylene (0.2 mmol) as an internal standard.

Screening of Reaction Time

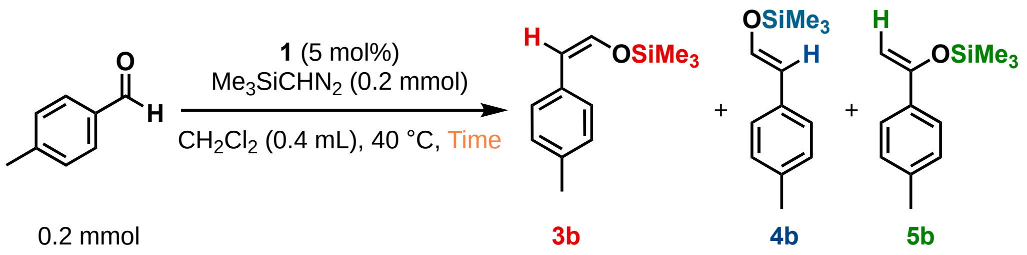

Entry	Deviation from above	Yields (%)[a] 3b:4b:5b
1	5 min	85:13:00
2	20 min	85:13:00
3	30 min	85:13:00
**4**	**60 min**	**87:13:00**
5	120 min	87:13:00

4. Protocol for (*Z*)-Silyl Enol Ether Synthesis

In an oven-dried 7 mL reaction vial under an argon atmosphere, the aldehyde substrate **2** (0.2 mmol), complex **1** (12.56 mg, 5 mol%), and dichloromethane (0.4 mL) were added. Me_3SiCHN_2 (100 µL, 0.2 mmol) was then introduced dropwise using a micropipette while stirring. The resulting mixture was stirred for 1 hour at 25 ºC. Subsequently, the solvent was removed *in vacuo*. The crude reaction mixture was extracted using pentane (3 × 5 mL), and the solution was concentrated. Mesitylene (0.2 mmol) was added as a standard to assess the conversion and yield of the reaction. The product was purified by silica gel column chromatography using dry and degassed Et_2O/Pentane mixtures as eluting solvents.

Note: The purification procedure should be performed under an argon atmosphere, and the use of dry solvents is mandatory to prevent the hydrolysis of silyl enol ethers to corresponding aldehydes or ketones.

*(Z)-Trimethyl(styryloxy)silane (**3a**)*[3]

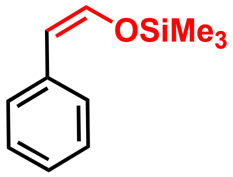

Compound **3a** was synthesized following the general procedure, employing benzaldehyde (20.4 µL, 0.2 mmol) as the substrate. The crude product was then purified by silica gel column chromatography, with an Et_2O/pentane (0.5:99.5) mixture as the eluent.

Formula: $C_{11}H_{16}OSi$

Molecular weight: 192.33

Appearance: colorless oil.

Isolated yield: 67% (26 mg, 0.13 mmol).

^{1}H NMR (400 MHz, $CDCl_3$, 296 K): δ 7.62 (d, J = 8.4 Hz, 2H), 7.29 (t, J = 7.7 Hz, 2H), 7.18 – 7.12 (m, 1H), 6.41 (d, J = 6.6 Hz, 1H), 5.33 (d, J = 6.6 Hz, 1H), 0.28 (s, 9H).

^{13}C{^{1}H} NMR (101 MHz, $CDCl_3$, 296 K): δ 140.0, 136.3, 128.3 (2C), 125.9, 109.6, -0.3.

HRMS (ESI$^+$): Calcd. for $C_{11}H_{17}OSi$ $[M + H]^+$: 193.1043; Found 193.1046.

*(Z)-Trimethyl((4-methylstyryl)oxy)silane (**3b**)*

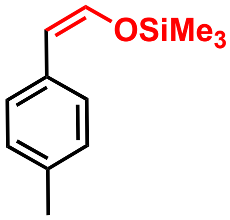

Compound **3b** was synthesized following the general procedure, employing 4-methylbenzaldehyde (23.6 μL, 0.2 mmol) as the substrate. The crude product was then purified by silica gel column chromatography, with an Et_2O/pentane (0.5:99.5) mixture as the eluent.

Formula: $C_{12}H_{18}OSi$

Molecular weight: 206.36

Appearance: colorless oil.

Isolated yield: 85% (35 mg, 0.17 mmol).

^{1}H NMR (400 MHz, $CDCl_3$, 296 K): δ 7.53 (d, *J* = 8.2 Hz, 2H), 7.23-7.05 (m, 2H), 6.38 (d, *J* = 6.6 Hz, 1H), 5.33 (d, *J* = 6.5 Hz, 1H), 2.34 (s, 3H), 0.29 (s, 9H).

^{13}C{^{1}H} NMR (101 MHz, $CDCl_3$, 296 K): δ 139.2, 135.5, 133.4, 129.0, 128.3, 109.5, 21.3, -0.3.

HRMS (ESI$^+$): Calcd. for $C_{12}H_{19}OSi$ $[M + H]^+$: 207.1200; Found 207.1203.

*(Z)-((2,5-Dimethylstyryl)oxy)trimethylsilane (**3c**)*

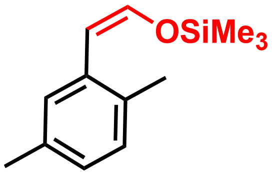

Compound **3c** was synthesized following the general procedure, employing 2,5-dimethyl benzaldehyde (28.2 μL, 0.2 mmol) as the substrate. The crude product was then purified by silica gel column chromatography, with an Et_2O/pentane (0.1:99.9) mixture as the eluent.

Formula: $C_{13}H_{20}OSi$

Molecular weight: 220.39

Appearance: colorless oil.

Isolated yield: 76% (34 mg, 0.15 mmol).

^{1}H NMR (400 MHz, C_6D_6, 296 K): δ 8.15 (s, 1H), 7.04 (d, *J* = 7.6 Hz, 1H), 6.90 (m, 1H), 6.33 (d, *J* = 6.7 Hz, 1H), 5.53 (d, *J* = 6.7 Hz, 1H), 2.27 (s, 3H), 2.24 (s, 3H), 0.06 (s, 9H).

^{13}C{^{1}H} NMR (101 MHz, C_6D_6, 296 K): δ 139.4, 134.8 (2C), 132.2, 130.5, 130.2, 127.2, 107.8, 21.4, 19.9, -0.7.

HRMS (GC-EI): Calcd. for $C_{13}H_{20}OSi$ $[M]^+$: 220.1278; Found 220.1277.

*(Z)-((4-Isopropylstyryl)oxy)trimethylsilane (**3d**)*

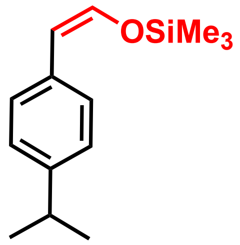

Compound **3d** was synthesized following the general procedure, employing cuminaldehyde (30.2 μL, 0.2 mmol) as the substrate. The crude product was then purified by silica gel column chromatography, with an Et_2O/pentane (0.5:99.5) mixture as the eluent.

Formula: $C_{14}H_{22}OSi$

Molecular weight: 234.41

Appearance: colorless oil.

Isolated yield: 76% (36 mg, 0.15 mmol).

^{1}H NMR (400 MHz, C_6D_6, 296 K): δ 7.81 (d, J = 8.3 Hz, 2H), 7.17 (d, J = 8.2 Hz, 2H), 6.27 (d, J = 6.5 Hz, 1H), 5.43 (d, J = 6.6 Hz, 1H), 2.74 (p, J = 6.9 Hz, 1H), 1.16 (d, J = 6.9 Hz, 6H), 0.08 (s, 9H).

^{13}C{^{1}H} NMR (101 MHz, C_6D_6, 296 K): δ 146.6, 139.2, 134.6, 129.0, 126.6, 110.4, 34.3, 24.2, -0.7.

HRMS (GC-EI): Calcd. for $C_{14}H_{22}OSi$ $[M]^+$: 234.1434; Found 234.1434.

*(Z)-Trimethyl((2,4,6-trimethylstyryl)oxy)silane (**3e**)*

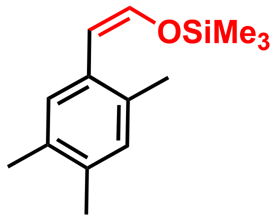

Compound **3e** was synthesized following the general procedure, employing 2,4,6-trimethylbenzaldehyde (29.6 mg, 0.2 mmol) as the substrate. The crude product was then purified by silica gel column chromatography, with an Et_2O/pentane (0:100) mixture as the eluent.

Formula: $C_{14}H_{22}OSi$

Molecular weight: 234.41

Appearance: colorless oil

Isolated yield: 73% (34 mg, 0.15 mmol).

^{1}H NMR (400 MHz, C_6D_6, 296 K): δ 8.14 (s, 1H), 6.91 (s, 1H), 6.34 (d, J = 6.7 Hz, 1H), 5.56 (d, J = 6.7 Hz, 1H), 2.26 (s, 3H), 2.18 (s, 3H), 2.08 (s, 3H), 0.08 (s, 9H).

$^{13}C\{^{1}H\}$ NMR (101 MHz, C_6D_6, 296 K): δ 138.8, 134.1, 133.3, 132.6, 132.5, 131.7, 131.1, 107.9, 19.8, 19.7, 19.5, -0.6.

HRMS (GC-EI): Calcd. for $C_{14}H_{22}OSi$ $[M]^+$: 234.1434; Found 234.1434.

*(Z)-Trimethyl((2,3,4,5,6-pentamethylstyryl)oxy)silane (**3f**)*

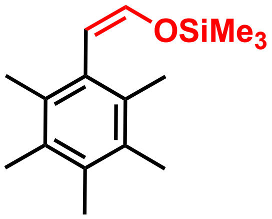

Compound **3f** was synthesized following the general procedure, employing pentamethyl benzaldehyde (35.2 mg, 0.2 mmol) as the substrate. The crude product was then purified by silica gel column chromatography, with an Et_2O/pentane (0:100) mixture as the eluent.

Formula: $C_{16}H_{26}OSi$

Molecular weight: 262.47

Appearance: colorless oil

Isolated yield: 80% (42 mg, 0.16 mmol).

^{1}H NMR (400 MHz, C_6D_6, 296 K): δ 6.30 (d, J = 6.5 Hz, 1H), 5.52 (d, J = 6.5 Hz, 1H), 2.36 (s, 6H), 2.13 (s, 6H), 2.09 (s, 3H), 0.03 (s, 9H)

$^{13}C\{^{1}H\}$ NMR (101 MHz, C_6D_6, 296 K): δ 138.3, 133.0, 132.1, 131.9, 131.8, 111.3, 18.2, 16.8 (2C), -0.5.

HRMS(GC-EI): Calcd. for $C_{16}H_{26}OSi$ $[M]^+$: 262.1764; Found 262.1764.

*(Z)-((2-Ethynylstyryl)oxy)trimethylsilane (**3g**)*

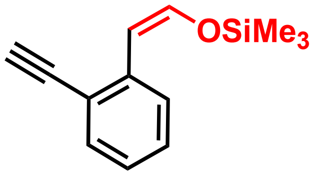

Compound **3g** was synthesized following the general procedure, employing 2-ethynylbenzaldehyde (28.5 mg, 0.2 mmol) as the substrate. The crude product was then purified by silica gel column chromatography, with an Et_2O/pentane (0.5:99.5) mixture as the eluent.

Formula: $C_{13}H_{16}OSi$

Molecular weight: 216.36

Appearance: yellow oil

Isolated yield: 69% (30 mg, 0.18 mmol) as a mixture of *(Z)* and *(E)* isomers (75:25).

^{1}H NMR (400 MHz, CDCl$_3$, 296 K): δ 8.19 (dd, *J* = 8.1, 1.2 Hz, 1H), 7.51-7.43 (m, 1H), 7.30 (td, *J* = 7.7, 1.5 Hz, 1H), 7.10 – 7.07 (m, 1H), 6.52 (d, *J* = 6.7 Hz, 1H), 5.93 (d, *J* = 6.7 Hz, 1H), 3.30 (s, 1H), 0.28 (s, 9H).

^{13}C{^{1}H} NMR (101 MHz, CDCl$_3$, 296 K): δ 141.4, 138.3, 132.8, 128.8, 128.5, 125.5, 119.9, 106.6, 82.9, 81.2, -0.3.

HRMS (GC-EI): Calcd. for $C_{13}H_{16}OSi$ $[M]^+$: 216.0965; Found 216.0964.

*(Z)-((2-([1,1'-Biphenyl]-4-yl)vinyl)oxy)trimethylsilane (**3h**)*

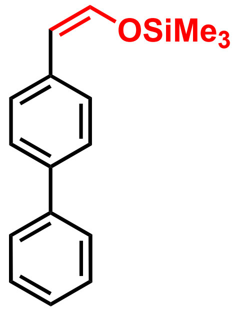

Compound **3h** was synthesized following the general procedure, employing biphenyl-4-carboxyaldehyde (36.44 mg, 0.2 mmol) as the substrate. The crude product was then purified by silica gel column chromatography, with an Et_2O/pentane (0.1:99.9) mixture as the eluent.

Formula: $C_{17}H_{20}OSi$

Molecular weight: 268.43

Appearance: yellow oil.

Isolated yield: 73% (39 mg, 0.15 mmol).

^{1}H NMR (400 MHz, CDCl$_3$, 296 K): δ 7.70 (d, *J* = 8.4 Hz, 2H), 7.61 (d, *J* = 7.0 Hz, 2H), 7.55 (d, *J* = 8.4 Hz, 2H), 7.43 (t, *J* = 7.7 Hz, 2H), 7.32 (t, *J* = 7.4 Hz, 1H), 6.45 (d, *J* = 6.5 Hz, 1H), 5.39 (d, *J* = 6.6 Hz, 1H), 0.30 (s, 9H).

^{13}C{^{1}H} NMR (101 MHz, CDCl$_3$, 296 K): δ 141.3, 140.3, 138.5, 135.5, 128.9, 128.7, 127.1, 127.0, 126.9, 109.2, -0.3.

HRMS (GC-EI): Calcd. for $C_{17}H_{20}OSi$ $[M]^+$: 268.1278; Found 268.1277.

*(Z)-Trimethyl((2-(naphthalen-2-yl)vinyl)oxy)silane (**3i**)*

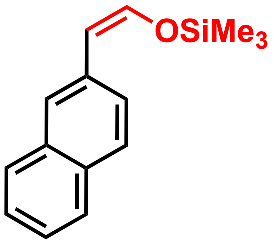

Compound **3i** was synthesized following the general procedure, employing 2-napthaldehyde (31.2 mg, 0.2 mmol) as the substrate. The crude product was then purified by silica gel column chromatography, with an Et_2O/pentane (0.1:99.9) mixture as the eluent.

Formula: $C_{15}H_{18}OSi$

Molecular weight: 242.39

Appearance: colorless oil

Isolated yield: 71% (34 mg, 0.14 mmol).

^{1}H NMR (400 MHz, C_6D_6, 296 K): δ 8.17 (s, 1H), 8.10 – 8.04 (m, 1H), 7.73 (d, J = 8.2 Hz, 1H), 7.70 (d, J = 8.6 Hz, 1H), 7.63 (d, J = 8.0 Hz, 1H), 7.30 – 7.21 (m, 2H), 6.34 (d, J = 6.5 Hz, 1H), 5.54 (d, J = 6.6 Hz, 1H), 0.08 (s, 9H).

^{13}C{^{1}H} NMR (101 MHz, C_6D_6, 296 K): δ 140.4, 134.5, 134.4, 132.7, 127.6, 127.3, 126.2, 125.5, 110.6, -0.7.

HRMS(GC-EI): Calcd. for $C_{15}H_{18}OSi$ $[M]^+$: 242.1121; Found 242.1120.

*(Z)-((2-(Anthracen-9-yl)vinyl)oxy)trimethylsilane (**3j**)*

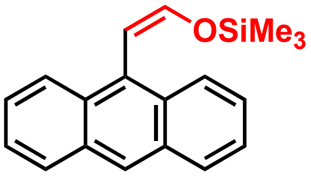

Compound **3j** was synthesized following the general procedure, employing anthracene-9-carbaldehyde (41.25 mg, 0.2 mmol) as the substrate. The crude product was then purified by silica gel column chromatography, with an Et_2O/pentane (0.1:99.9) mixture as the eluent.

Formula: $C_{19}H_{20}OSi$

Molecular weight: 292.45

Appearance: yellow oil

Isolated yield: 38% (22 mg, 0.08 mmol).

^{1}H NMR (400 MHz, $CDCl_3$, 296 K): δ 8.37 (s, 1H), 8.25 – 8.18 (m, 2H), 7.99 (dd, J = 6.3, 3.5 Hz, 2H), 7.48 – 7.42 (m, 4H), 6.86 (d, J = 6.5 Hz, 1H), 6.16 (d, J = 6.5 Hz, 1H), 0.02 (s, 9H).

^{13}C{^{1}H} NMR (101 MHz, $CDCl_3$, 296 K): δ 141.1, 131.6 (2C), 130.0, 129.8 (2C), 128.6 (2C), 127.2 (2C), 126.1, 125.1 (2C), 124.9 (2C), 105.9, -0.2.

HRMS (GC-EI): Calcd. for $C_{19}H_{20}OSi$ $[M]^+$: 292.1278; Found 292.1279.

*(Z)-Trimethyl((2-(phenanthren-9-yl)vinyl)oxy)silane (**3k**)*

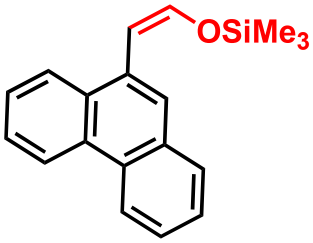

Compound **3k** was synthesized following the general procedure, employing 9-phenanthrenecarboxaldehyde (41.3 mg, 0.2 mmol) as the substrate. The crude product was then purified by silica gel column chromatography, with an Et_2O/pentane (0.1:99.9) mixture as the eluent.

Formula: $C_{19}H_{20}OSi$

Molecular weight: 292.45

Appearance: colorless oil

Isolated yield: 70% (41 mg, 0.14 mmol).

^{1}H NMR (400 MHz, C_6D_6, 296 K): δ 8.56 (d, J = 7.0 Hz, 2H), 8.48 – 8.45 (m, 1H), 8.23 (dd, J = 7.0, 2.6 Hz, 1H), 7.83 – 7.77 (m, 1H), 7.50 – 7.43 (m, 2H), 7.39 – 7.37 (m, 2H), 6.53 (d, J = 6.6 Hz, 1H), 6.02 (d, J = 6.7 Hz, 1H), 0.03 (s, 9H).

^{13}C{^{1}H} NMR (101 MHz, C_6D_6, 296 K): δ 141.1, 132.7, 131.6, 131.1, 130.6, 130.1, 128.8, 126.9, 126.6, 126.3, 125.2, 123.5, 122.9, 106.9, -0.6.

HRMS (GC-EI): Calcd. for $C_{19}H_{20}OSi$ $[M]^+$: 292.1278; Found 292.1277.

*(Z)-((2-(9H-Fluoren-2-yl)vinyl)oxy)trimethylsilane (**3l**)*

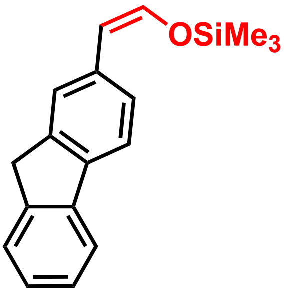

Compound **3l** was synthesized following the general procedure, employing fluorene-2-carboxyaldehyde (38.85 mg, 0.2 mmol) as the substrate. The crude product was then purified by silica gel column chromatography, with an Et_2O/pentane (0.1:99.9) mixture as the eluent.

Formula: $C_{18}H_{20}OSi$

Molecular weight: 280.44

Appearance: yellow oil.

Isolated yield: 71% (39 mg, 0.14 mmol).

^{1}H NMR (400 MHz, CDCl$_3$, 296 K): δ 7.84 (s, 1H), 7.76 – 7.70 (m, 2H), 7.64 (d, *J* = 7.8 Hz, 1H), 7.52 (d, *J* = 7.58 Hz, 1H), 7.36 (t, *J* = 7.5 Hz, 1H), 7.29-7.25 (m, 1H), 6.43 (d, *J* = 6.5 Hz, 1H), 5.42 (d, *J* = 6.6 Hz, 1H), 3.89 (s, 2H), 0.31 (s, 9H).

^{13}C{^{1}H} NMR (101 MHz, CDCl$_3$, 296 K): δ 143.6, 143.4, 142.0, 139.7, 139.5, 135.1, 127.2, 126.8, 126.4, 125.1, 124.8, 119.8, 119.7, 110.0, 37.1, -0.3.

HRMS(GC-EI): Calcd. for $C_{18}H_{20}OSi$ [M]$^+$: 280.1278; Found 280.1278.

*(Z)-(4-Fluorostyryl)oxy)trimethylsilane (**3m**)*

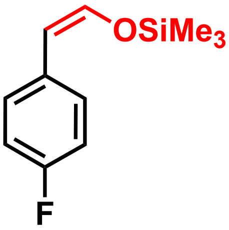

Compound **3m** was synthesized following the general procedure, employing 4-fluorobenzaldehyde (21.5 μL, 0.2 mmol) as the substrate. The crude product was then purified by silica gel column chromatography, with an Et_2O/pentane (0.5:99.5) mixture as the eluent.

Formula: $C_{11}H_{15}FOSi$

Molecular weight: 210.32

Appearance: colorless oil.

Isolated yield: 71% (30 mg, 0.14 mmol).

^{1}H NMR (400 MHz, CDCl$_3$, 296 K): δ 7.59 (dd, *J* = 8.6, 5.8 Hz, 2H), 6.97 (t, *J* = 8.8 Hz, 2H), 6.38 (d, *J* = 6.5 Hz, 1H), 5.30 (d, *J* = 6.5 Hz, 1H), 0.27 (d, *J* = 2.2 Hz, 9H).

^{13}C{^{1}H} NMR (101 MHz, CDCl$_3$, 296 K): δ 151.3, 139.5, 132.4, 129.8, 115.2, 108.5, -0.3.

HRMS (GC-EI): Calcd. for $C_{11}H_{15}FOSi$ [M]$^+$ 210.0870; Found 210.0870.

*(Z)-((4-Chlorostyryl)oxy)trimethylsilane (**3n**)*[4]

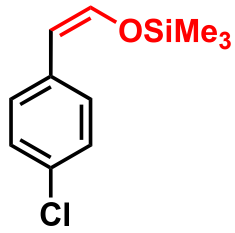

Compound **3n** was synthesized following the general procedure, employing 4-chlorobenzaldehyde (28.1 mg, 0.2 mmol) as the substrate. The crude product was then purified by silica gel column chromatography, with an Et_2O/pentane (0.5:99.5) mixture as the eluent.

Formula: $C_{11}H_{15}ClOSi$

Molecular weight: 226.78

Appearance: colorless oil.

Isolated yield: 75% (34 mg, 0.15 mmol).

^{1}H NMR (400 MHz, $CDCl_3$, 296 K): δ 7.55 (d, *J* = 8.6 Hz, 2H), 7.24 (d, *J* = 8.6 Hz, 2H), 6.42 (d, *J* = 6.5 Hz, 1H), 5.29 (d, *J* = 6.6 Hz, 1H), 0.28 (s, 9H).

^{13}C{^{1}H} NMR (101 MHz, $CDCl_3$, 296 K): δ 140.5, 134.8, 131.1, 129.5, 128.3, 108.4, -0.4.

HRMS (GC-EI): Calcd. for $C_{11}H_{15}ClOSi$ $[M]^+$: 226.0575; Found 226.0574.

*(Z)-((3-Bromostyryl)oxy)trimethylsilane (**3o**)*

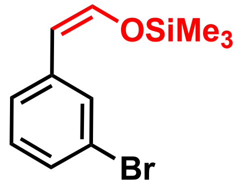

Compound **3o** was synthesized following the general procedure, employing 3-bromobenzaldehyde (23.32 μL, 0.2 mmol) as the substrate. The crude product was then purified by silica gel column chromatography, with an Et_2O/pentane (0.5:99.5) mixture as the eluent.

Formula: $C_{11}H_{15}BrOSi$

Molecular weight: 271.23

Appearance: colorless oil.

Isolated yield: 68% (37 mg, 0.14 mmol).

^{1}H NMR (400 MHz, C_6D_6, 296 K): δ 8.12 (t, *J* = 1.8 Hz, 1H), 7.50-7.47 (m, 1H), 7.18 (dd, *J* = 2.1, 1.0 Hz, 1H), 6.84 (t, *J* = 7.9 Hz, 1H), 6.17 (d, *J* = 6.5 Hz, 1H), 5.15 (d, *J* = 6.5 Hz, 1H), 0.00 (s, 9H).

^{13}C{^{1}H} NMR (101 MHz, C_6D_6, 296 K): δ 141.2, 139.0, 131.7, 130.0, 129.0, 127.1, 122.9, 108.8, -0.8.

HRMS(GC-EI): Calcd. for $C_{11}H_{15}BrOSi$ $[M]^+$: 270.0070; Found 270.0070.

*(Z)-((4-Methoxystyryl)oxy)trimethylsilane (**3p**)* [5]

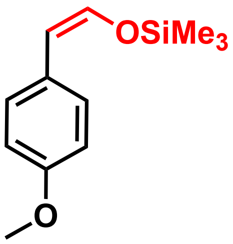

Compound **3p** was synthesized following the general procedure, employing 4-methoxybenzaldehyde (24.3 µL, 0.2 mmol) as the substrate. The crude product was then purified by silica gel column chromatography, with an Et_2O/pentane (0.5:99.5) mixture as the eluent.

Formula: $C_{12}H_{18}O_2Si$

Molecular weight: 222.36

Appearance: colorless oil.

Isolated yield: 81% (36 mg, 0.16 mmol).

^{1}H NMR (400 MHz, $CDCl_3$, 296 K): δ 7.56 (d, J = 7.0 Hz, 2H), 6.84 (d, J = 6.9 Hz, 2H), 6.33 (d, J = 6.5 Hz, 1H), 5.29 (d, J = 6.5 Hz, 1H), 3.80 (s, 3H), 0.27 (s, 9H).

^{13}C{^{1}H} NMR (101 MHz, $CDCl_3$, 296 K): δ 157.7, 138.3, 129.5, 129.2, 113.7, 109.1, 55.4, -0.3.

HRMS(GC-EI): Calcd. for $C_{12}H_{18}O_2Si$ $[M]^+$: 222.1071; Found 222.1069.

*(Z)-((2-Methoxystyryl)oxy)trimethylsilane (**3q**)*

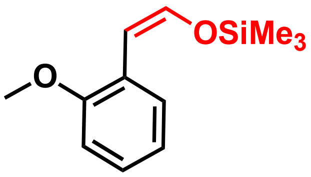

Compound **3q** was synthesized following the general procedure, employing 2-methoxybenzaldehyde (27.2 mg, 0.2 mmol) as the substrate. The crude product was then purified by silica gel column chromatography, with an Et_2O/pentane (0.5:99.5) mixture as the eluent.

Formula: $C_{12}H_{18}O_2Si$

Molecular weight: 222.36

Appearance: colorless oil

Isolated yield: 65% (29 mg, 0.13 mmol).

^{1}H NMR (400 MHz, C_6D_6, 296 K): δ 8.60 (dd, *J* = 7.2, 2.3 Hz, 1H), 7.09 – 7.02 (m, 2H), 6.61 – 6.58 (m, 1H), 6.41 (d, *J* = 6.8 Hz, 1H), 6.24 (d, *J* = 6.8 Hz, 1H), 3.35 (s, 3H), 0.04 (s, 9H).

^{13}C{^{1}H} NMR (101 MHz, C_6D_6, 296 K): δ 156.4, 139.7, 130.3, 127.2, 125.7, 120.8, 110.6, 103.7, 55.0, -0.7.

HRMS(ESI$^+$): Calcd. for $C_{12}H_{18}O_2SiNa$ [M + Na]$^+$: 245.0968; Found 245.0970.

*(Z)-((2,5-Dimethoxystyryl)oxy)trimethylsilane (**3r**)*

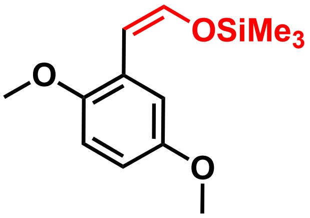

Compound **3r** was synthesized following the general procedure, employing 2,5-dimethoxybenzaldehyde (33.23 mg, 0.2 mmol) as the substrate. The crude product was then purified by silica gel column chromatography, with an Et_2O/pentane (0.5:99.5) mixture as the eluent.

Formula: $C_{13}H_{20}O_3Si$

Molecular weight: 252.39

Appearance: colorless oil

Isolated yield: 71% (36 mg, 0.14 mmol).

^{1}H NMR (400 MHz, C_6D_6, 296 K): δ 8.29 (d, *J* = 4.2 Hz, 1H), 6.74 (dd, *J* = 8.3, 3.7 Hz, 1H), 6.57 (d, *J* = 7.8 Hz, 1H), 6.39 (d, *J* = 5.6 Hz, 1H), 6.24 (d, *J* = 6.8 Hz, 1H), 3.53 (s, 3H), 3.39 (s, 3H), 0.03 (s, 9H).

^{13}C{^{1}H} NMR (101 MHz, C_6D_6, 296 K): δ 154.1, 151.0, 140.0, 126.5, 115.8, 112.5, 111.7, 103.8, 55.8, 55.2, -0.7.

HRMS(ESI$^+$): Calcd. for $C_{13}H_{20}O_3SiNa$ [M + Na]$^+$: 275.1074; Found 275.1075.

*(Z)-((4-Butoxystyryl)oxy)trimethylsilane (**3s**)*

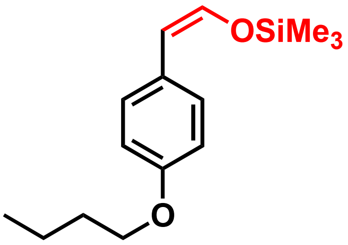

Compound **3s** was synthesized following the general procedure, employing 4-butoxybenzaldehyde (34.6 µL, 0.2 mmol) as the substrate. The crude product was then purified by silica gel column chromatography, with an Et_2O/pentane (0.5:99.5) mixture as the eluent.

Formula: $C_{15}H_{24}O_2Si$

Molecular weight: 264.44

Appearance: colorless oil.

Isolated yield: 80% (42 mg, 0.16 mmol).

^{1}H NMR (400 MHz, CDCl$_3$, 296 K): δ 7.57 – 7.53 (m, 2H), 6.83 (d, *J* = 8.9 Hz, 2H), 6.32 (d, *J* = 6.6 Hz, 1H), 5.28 (d, *J* = 6.6 Hz, 1H), 3.96 (t, *J* = 6.5 Hz, 2H), 1.76 (t, *J* = 7.7 Hz, 2H), 1.52 – 1.45 (m, 2H), 0.97 (t, *J* = 7.4 Hz, 3H), 0.27 (s, 9H).

^{13}C{^{1}H} NMR (101 MHz, CDCl$_3$, 296 K): δ 157.3, 138.1, 129.5, 129.0, 114.3, 109.2, 67.8, 31.5, 19.4, 14.0, -0.3.

HRMS(ESI$^+$): Calcd. for $C_{15}H_{24}O_2SiNa$ $[M + Na]^+$: 287.1438; Found 287.1439.

*(Z)-((4-(Benzyloxy)styryl)oxy)trimethylsilane (**3t**)*

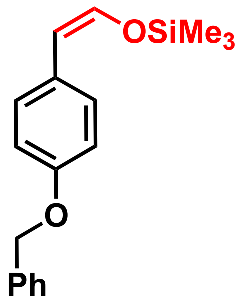

Compound **3t** was synthesized following the general procedure, employing 4-(benzyloxy)-benzaldehyde (42.5 mg, 0.2 mmol) as the substrate. The crude product was then purified by silica gel column chromatography, with an Et_2O/pentane (0.1:99.9) mixture as the eluent.

Formula: $C_{18}H_{22}O_2Si$

Molecular weight: 298.46

Appearance: colorless oil

Isolated yield: 85% (51 mg, 0.17 mmol).

^{1}H NMR (400 MHz, C$_6$D$_6$, 296 K): δ 7.78 (d, *J* = 8.8 Hz, 2H), 7.24 (d, *J* = 7.2 Hz, 2H), 7.13 (d, *J* = 7.7 Hz, 2H), 7.08 (d, *J* = 7.3 Hz, 1H), 6.96 (d, *J* = 8.9 Hz, 2H), 6.24 (d, *J* = 6.5 Hz, 1H), 5.39 (d, *J* = 6.6 Hz, 1H), 4.72 (s, 2H), 0.07 (s, 9H).

^{13}C{^{1}H} NMR (101 MHz, C$_6$D$_6$, 296 K): δ 157.6, 138.3, 137.8, 130.1, 130.0, 128.7, 127.9, 127.7, 115.0, 110.0, 69.9, -0.7.

HRMS (GC-EI): Calcd. for $C_{18}H_{22}O_2Si$ $[M]^+$: 298.1383; Found 298.1384.

*(Z)-4-(2-((Trimethylsilyl)oxy)vinyl)phenol (**3u**)*

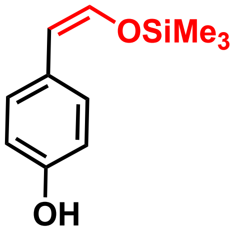

Compound **3u** was synthesized following a modified version of the general procedure, employing 4-hydroxybenzaldehyde (24.42 mg, 0.2 mmol) as the substrate.

Modifications: Me_3SiCHN_2 (200µL, 0.4 mmol).

Formula: $C_{11}H_{16}O_2Si$

Molecular weight: 208.33

Appearance: colorless oil

NMR-yield: *Z*:*E*:**5** = 80:12:8

Conversion: >99%

*(Z)-N,N-Dimethyl-4-(2-((trimethylsilyl)oxy)vinyl)aniline (**3v**)* [4]

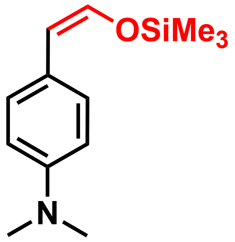

Compound **3v** was synthesized following the general procedure, employing 4-diemethylaminobenzaldehyde (33.23 mg, 0.2 mmol) as the substrate. The crude product was then purified by silica gel column chromatography, with an Et_2O/pentane (0.5:99.5) mixture as the eluent.

Formula: $C_{13}H_{21}NOSi$

Molecular weight: 235.40

Appearance: colorless oil

Isolated yield: 85% (36 mg, 0.15 mmol).

^{1}H NMR (400 MHz, C_6D_6, 296 K): δ 7.87 (d, J = 8.9 Hz, 2H), 6.69 (d, J = 8.9 Hz, 2H), 6.27 (d, J = 6.5 Hz, 1H), 5.49 (d, J = 6.5 Hz, 1H), 2.52 (s, 6H), 0.12 (s, 9H).

^{13}C{^{1}H} NMR (101 MHz, C_6D_6, 296 K): δ 149.3, 136.9, 129.9, 125.9, 112.8, 110.9, 40.3, -0.6.

HRMS(ESI$^+$): Calcd. for $C_{13}H_{22}NOSi$ $[M + H]^+$: 236.1465; Found 236.1465.

*tert-Butyl (Z)-(4-(2-((trimethylsilyl)oxy)vinyl)phenyl)carbamate (**3w**)*

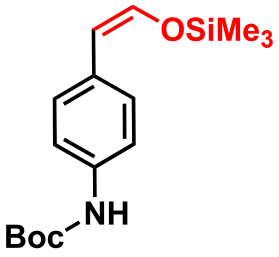

Compound **3w** was synthesized following the general procedure, employing 4-(Boc-amino)benzaldehyde (44.3 mg, 0.2 mmol) as the substrate. The crude product was then purified by silica gel column chromatography, with an Et_2O/pentane (0.5:99.5) mixture as the eluent.

Formula: $C_{16}H_{25}NO_3Si$

Molecular weight: 307.47

Appearance: colorless oil

Isolated yield: 81% (50 mg, 0.16 mmol).

^{1}H NMR (400 MHz, C_6D_6, 296 K): δ 7.77 – 7.71 (m, 2H), 7.35 (d, J = 8.1 Hz, 2H), 6.20 (d, J = 6.5 Hz, 1H), 5.97 (s, 1H), 5.32 (d, J = 6.6 Hz, 1H), 1.42 (s, 9H), 0.03 (s, 9H).

^{13}C{^{1}H} NMR (101 MHz, C_6D_6, 296 K): δ 152.5, 138.8, 136.9, 131.7, 129.4, 118.3, 110.0, 30.2, 28.4, -0.7.

HRMS(ESI$^+$): Calcd. for $C_{16}H_{25}NO_3SiNa$ $[M + Na]^+$: 330.1496; Found 330.1496.

*(Z)-Trimethyl((4-(methylthio)styryl)oxy)silane (**3x**)*

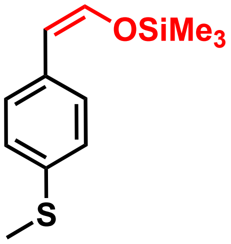

Compound **3x** was synthesized following the general procedure, employing 4-methylthiobenzaldehyde (26.6 μL, 0.2 mmol) as the substrate. The crude product was then purified by silica gel column chromatography, with an Et_2O/pentane (0.1:99.9) mixture as the eluent.

Formula: $C_{12}H_{18}OSSi$

Molecular weight: 238.42

Appearance: colorless oil

Isolated yield: 78% (37 mg, 0.16 mmol).

^{1}H NMR (400 MHz, C_6D_6, 296 K): δ 7.71 (d, J = 8.4 Hz, 2H), 7.21 (d, J = 8.5 Hz, 2H), 6.24 (d, J = 6.5 Hz, 1H), 5.34 (d, J = 6.5 Hz, 1H), 2.01 (s, 3H), 0.05 (s, 9H).

$^{13}C\{^{1}H\}$ NMR (101 MHz, C_6D_6, 296 K): δ 139.7, 133.8, 129.3, 127.1 109.9, 101.9, 15.7, -0.7.

HRMS(GC-EI): Calcd. for $C_{12}H_{18}OSSi$ $[M]^+$: 238.0842; Found 238.0841.

*(Z)-Trimethyl((2-(thiophen-2-yl)vinyl)oxy)silane (**3y**)* [4]

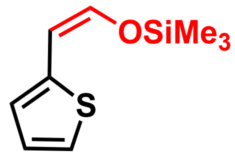

Compound **3y** was synthesized following a modified version of the general procedure, employing thiophene-2-carboxaldehyde (18.4 µL, 0.2 mmol) as the substrate.

Modifications: Me_3SiCHN_2 (200 µL, 0.4 mmol).

The crude product was then purified by silica gel column chromatography, with an Et_2O/pentane (0.5:99.5) mixture as the eluent.

Formula: $C_9H_{14}OSSi$

Molecular weight: 198.36

Appearance: Yellow oil.

Isolated yield: 72% (29 mg, 0.16 mmol).

^{1}H NMR (400 MHz, $CDCl_3$, 296 K): δ 7.17 (d, J = 4.5 Hz, 1H), 6.96 (d, J = 4.6 Hz, 2H), 6.39 (d, J = 5.9 Hz, 1H), 5.81 (d, J = 6.8 Hz, 1H), 0.07 (s, 9H).

$^{13}C\{^{1}H\}$ NMR (101 MHz, $CDCl_3$, 296 K): δ 138.6, 137.9, 126.1, 124.4, 124.2, 104.9, -0.3.

HRMS(GC-EI): Calcd. for $C_9H_{14}OSSi$ $[M]^+$: 198.0529; Found 198.0527.

*(Z)-Trimethyl((4-(thiophen-2-yl)styryl)oxy)silane (**3z**)*

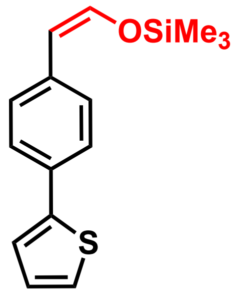

Compound **3z** was synthesized following the general procedure, employing 4-(2-thienyl)-benzaldehyde (37.6, 0.2 mmol) as the substrate. The crude product was then purified by silica gel column chromatography, with an Et_2O/pentane (0.1:99.9) mixture as the eluent.

Formula: $C_{15}H_{18}OSSi$

Molecular weight: 274.45

Appearance: white oil

Isolated yield: 75% (41 mg, 0.15 mmol).

^{1}H NMR (400 MHz, $CDCl_3$, 296 K): δ 7.63 (d, *J* = 8.4 Hz, 2H), 7.54 (d, *J* = 8.4 Hz, 2H), 7.29 (d, *J* = 3.6 Hz, 1H), 7.24 (d, *J* = 3.9 Hz, 1H), 7.07 (dd, *J* = 5.1, 3.6 Hz, 1H), 6.43 (d, *J* = 6.5 Hz, 1H), 5.35 (d, *J* = 6.5 Hz, 1H), 0.29 (s, 9H).

$^{13}C\{^1H\}$ NMR (101 MHz, $CDCl_3$, 296 K): δ 144.9, 140.4, 135.7, 131.8, 128.7, 128.1, 125.8, 124.4, 122.7, 109.1, -0.3.

HRMS(GC-EI): Calcd. for $C_{15}H_{18}OSSi$: $[M]^+$: 274.0842; Found 274.0841.

*(Z)-((2-(Benzo[b]thiophen-3-yl)vinyl)oxy)trimethylsilane (**3aa**)*

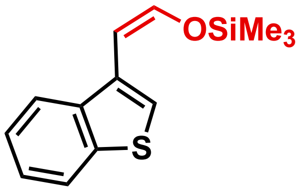

Compound **3aa** was synthesized following a modified version of the general procedure, employing benzo[b]thiophene-3-carboxaldehyde (32.4 mg, 0.2 mmol) as the substrate.

Modifications: Me_3SiCHN_2 (200μL, 0.4 mmol).

The crude product was then purified by silica gel column chromatography, with an Et_2O/pentane (1:99) mixture as the eluent.

Formula: $C_{13}H_{16}OSSi$

Molecular weight: 248.42

Appearance: yellow oil

Isolated yield: 73% (36 mg, 0.18 mmol).

^{1}H NMR (400 MHz, C_6D_6, 296 K): δ 8.18 (s, 1H), 7.77 (d, *J* = 8.1 Hz, 1H), 7.63 (d, *J* = 8.0 Hz, 1H), 7.23 (ddd, *J* = 8.1, 7.1, 1.1 Hz, 1H), 7.12 (ddd, *J* = 8.2, 6.9, 1.2 Hz, 1H), 6.39 (d, *J* = 6.2 Hz, 1H), 5.78 (d, *J* = 6.3 Hz, 1H), 0.04 (s, 9H).

$^{13}C\{^1H\}$ NMR (101 MHz, C_6D_6, 296 K): δ 140.6, 139.9, 139.1, 130.7, 124.5, 124.2, 123.6, 123.0, 121.7, 101.9, -0.7.

HRMS(GC-EI): Calcd. for $C_{13}H_{16}OSSi$ $[M]^+$: 248.0686; Found 248.0684.

*(Z)-((2-(Benzo[b]thiophen-2-yl)vinyl)oxy)trimethylsilane (**3ab**)*

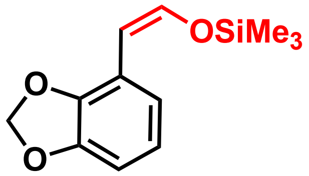

Compound **3ab** was synthesized following the general procedure, employing 2,3-(methylenedioxy)-benzaldehyde (30.0 mg, 0.2 mmol) as the substrate. The crude product was then purified by silica gel column chromatography, with an Et_2O/pentane (0:100) mixture as the eluent.

Formula: $C_{12}H_{16}O_3Si$

Molecular weight: 236.34

Appearance: colorless oil

Isolated yield: 60% (30 mg, 0.12 mmol).

^{1}H NMR (400 MHz, C_6D_6, 296 K): δ 8.03 (d, J = 8.2 Hz, 1H), 6.80 (t, J = 7.9 Hz, 1H), 6.62 (dd, J = 7.7, 1.2 Hz, 1H), 6.31 (d, J = 6.6 Hz, 1H), 5.80 (d, J = 6.5 Hz, 1H), 5.33 (s, 2H), 0.03 (s, 9H).

^{13}C{^{1}H} NMR (101 MHz, C_6D_6, 296 K): δ 147.5, 144.7, 141.1, 122.4, 121.7, 119.6, 106.6, 102.4, 100.4, -0.8.

HRMS(GC-EI): Calcd. for $C_{12}H_{16}O_3Si$ $[M]^+$: 236.0863; Found 236.0863.

*1,3-Bis((Z)-2-((trimethylsilyl)oxy)vinyl)benzene (**3ac**)*

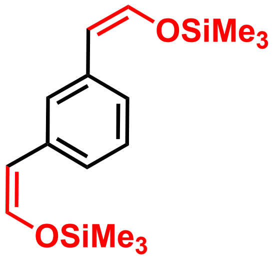

Compound **3ac** was synthesized following a modified version of the general procedure, employing isophthaldehyde (26.8 mg, 0.2 mmol) as the substrate.

Modifications: Me_3SiCHN_2 (200µL, 0.4 mmol).

Formula: $C_{16}H_{26}O_2Si_2$

Molecular weight: 306.55

NMR-yield: *Z*:*E*:**5** = 74:14:12

Conversion: >99%

*1,4-Bis((Z)-2-((trimethylsilyl)oxy)vinyl)benzene (**3ad**)*

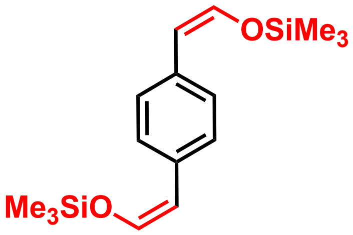

Compound **3ad** was synthesized following a modified version of the general procedure, employing terephthaldehyde (26.8 mg, 0.2 mmol) as the substrate.

Modifications: Me_3SiCHN_2 (200µL, 0.4 mmol).

Formula: $C_{16}H_{26}O_2Si_2$

Molecular weight: 306.55

NMR-yield: *Z*:*E*:**5** = 76:18:6

Conversion: >99%

*(Z)-4-(2-((Trimethylsilyl)oxy)vinyl)benzonitrile (**3ae**)*

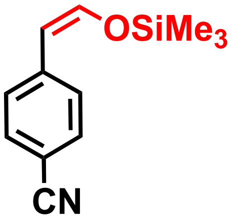

Compound **3ae** was synthesized following the general procedure, employing 4-cyanobenzaldehyde (26.2 mg, 0.2 mmol) as the substrate.

Formula: $C_{12}H_{15}NOSi$

Molecular weight: 217.34

NMR Yield: *Z/E/***5** = 62:12:26

Conversion: 94%

HRMS(ESI$^+$): Calcd. for $C_{12}H_{19}OSiNa$ $[M + Na]^+$: 240.0815; Found 240.0817.

*(Z)-Trimethyl((3-nitrostyryl)oxy)silane (**3af**)*

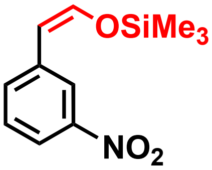

Compound **3af** was synthesized following the general procedure, employing 3-nitrobenzaldehyde (30.2 mg, 0.2 mmol) as the substrate.

Formula: $C_{11}H_{15}NO_3Si$

Molecular weight: 237.33

NMR Yield: *Z*:*E*:**5** = 66:8:26

Conversion: 96%

HRMS(ESI$^+$): Calcd. for $C_{11}H_{15}NO_3SiNa$ $[M + Na]^+$: 260.0713; Found 260.0715.

*(Z)-Trimethyl((4-(methylsulfonyl)styryl)oxy)silane (**3ag**)*

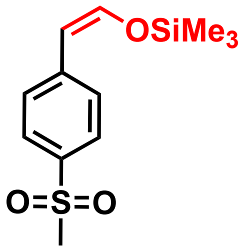

Compound **3ag** was synthesized following the general procedure, employing 4-(methylsulfonyl)benzaldehyde (9.21 mg, 0.05 mmol) as the substrate.

Formula: $C_{12}H_{18}O_3SSi$

Molecular weight: 270.42

NMR Yield: *Z*:*E*:**5** = 60:15:25

Conversion: 98%

HRMS(GC-EI): Calcd. For $C_{12}H_{18}O_3SSi$ $[M]^+$: 270.0740; Found 270.0740.

*(Z)-((2-Bromo-5-(trifluoromethyl)styryl)oxy)trimethylsilane (**3ah**)*

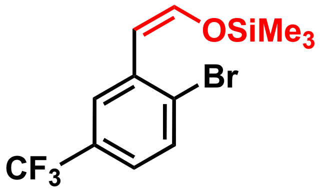

Compound **3ah** was synthesized following the general procedure, employing 2-bromo-5-(trifluoromethyl)benzaldehyde (30.17 µL, 0.2 mmol) as the substrate.

Formula: $C_{12}H_{14}BrF_3OSi$

Molecular weight: 339.23

NMR yield: *Z*:*E*:**5** = 52:14:34

Conversion: >99%

HRMS(GC-EI): Calcd. For $C_{12}H_{14}BrF_3OSi$ $[M]^+$: 337.9944; Found 337.9943.

*(Z)-Trimethyl((2-(perfluorophenyl)vinyl)oxy)silane (**3ai**)*

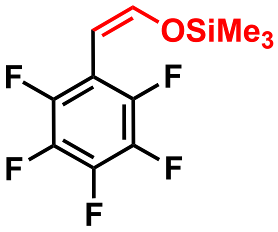

Compound **3ai** was synthesized following the general procedure, employing pentafluorobenzaldehyde (24.7 µL, 0.2 mmol) as the substrate.

Formula: $C_{11}H_{11}F_5OSi$

Molecular weight: 282.29

Appearance: colorless oil

NMR yield: *Z*:*E*:**5** = 21:8:71

Conversion: 98%

HRMS(GC-EI): Calcd. For $C_{11}H_{11}F_5OSi$ $[M]^+$: 282.0494; Found 282.0493.

5. Empirical Mechanistic Studies

5.1. Catalyst Screening under Optimized Conditions

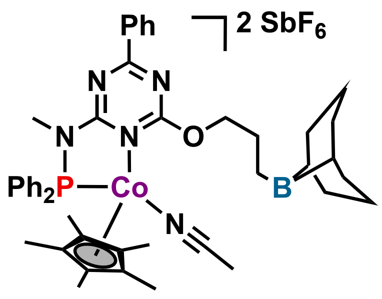

1

Complex **1** underwent testing as a catalyst in accordance with the general procedure for (*Z*)-silyl enol ether synthesis. Subsequently, product distribution was determined through NMR analysis:

3b, **4b**, and **5b** were produced in a ratio of 87:13:00, respectively. Moreover, a conversion of >99% was achieved.

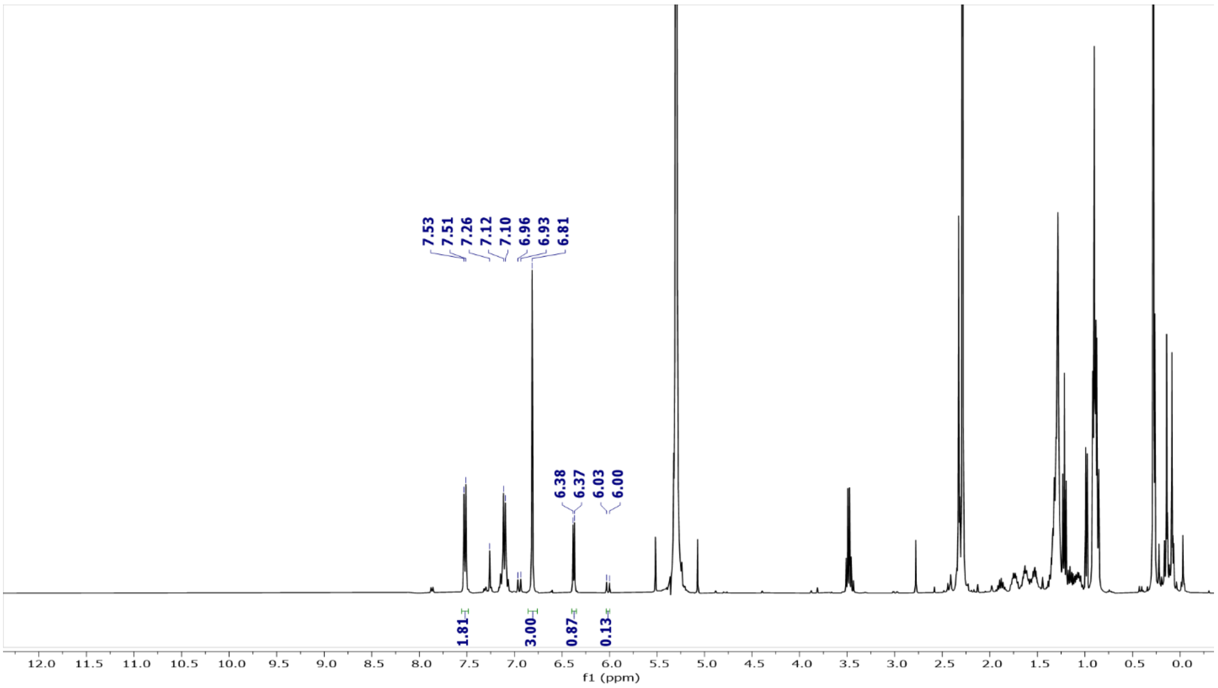

Figure S15 – **^{1}H NMR (400 MHz, $CDCl_3$, 293 K)** crude spectrum of the reaction mixture under optimized reaction conditions.

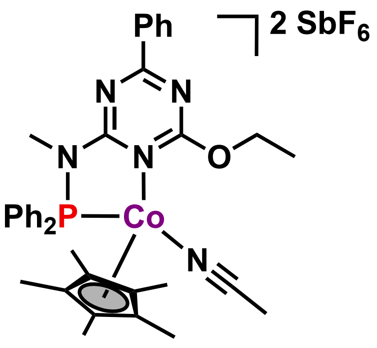

6

Complex **6** underwent testing as a catalyst in accordance with the general procedure for (*Z*)-silyl enol ether synthesis. Subsequently, product distribution was determined through NMR analysis:

3b, **4b**, and **5b** were produced in a ratio of 62:11:00, respectively. Moreover, a conversion of >99% was achieved.

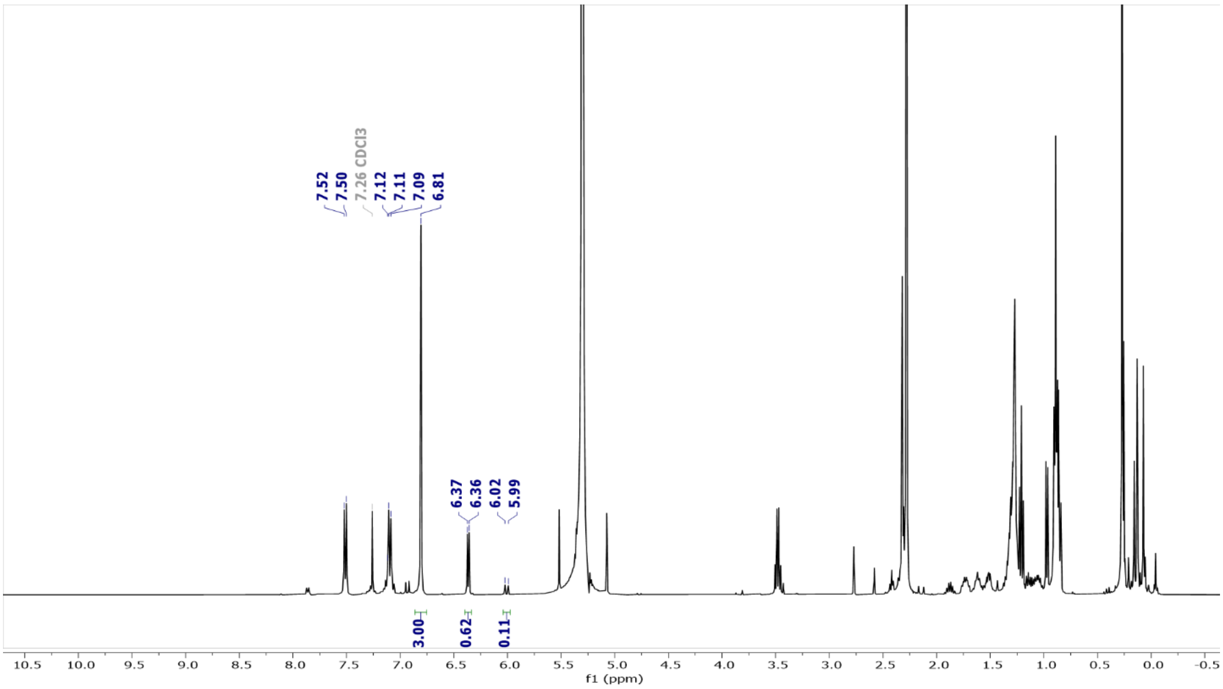

Figure S16 – **^{1}H NMR (400 MHz, $CDCl_3$, 293 K)** crude spectrum of the reaction mixture under optimized reaction conditions.

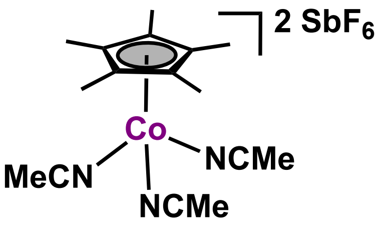

7

Complex **7** underwent testing as a catalyst in accordance with the general procedure for (*Z*)-silyl enol ether synthesis. Subsequently, product distribution was determined through NMR analysis:

3b, **4b**, and **5b** were produced in a ratio of 50:09:0, respectively. Moreover, a conversion of 83% was achieved.

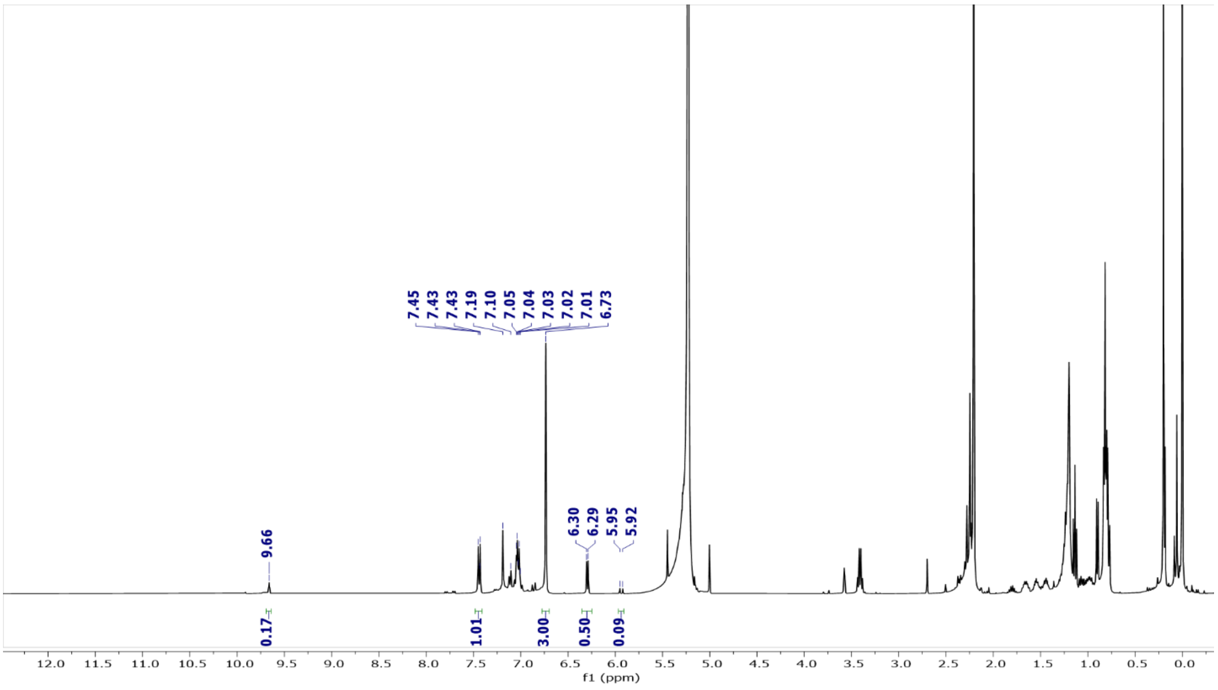

Figure S17 – **^{1}H NMR (400 MHz, $CDCl_3$, 293 K)** crude spectrum of the reaction mixture under optimized reaction conditions.

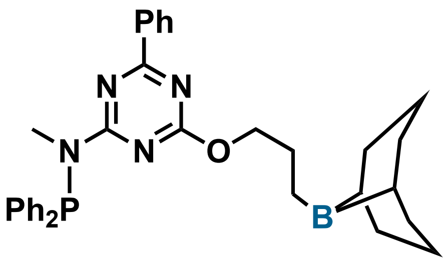

A

Ligand **A** underwent testing as a catalyst in accordance with the general procedure for (*Z*)-silyl enol ether synthesis. Subsequently, product distribution was determined through NMR analysis:

3b, **4b**, and **5b** were produced in a ratio of 0:0:0, respectively.

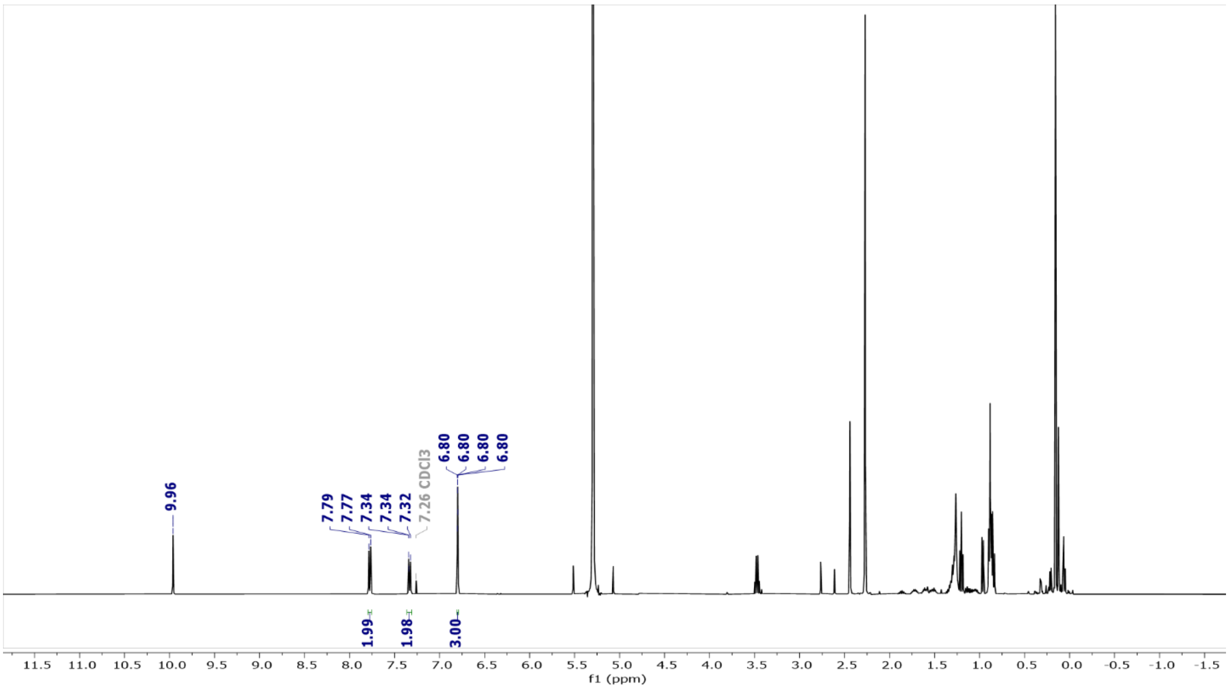

Figure S18 – **^{1}H NMR (400 MHz, $CDCl_3$, 293 K)** crude spectrum of the reaction mixture using optimized reaction conditions.

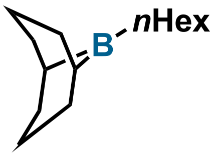

8

Compound **8** underwent testing as a catalyst in accordance with the general procedure for (*Z*)-silyl enol ether synthesis. Subsequently, product distribution was determined through NMR analysis:

3b, **4b**, and **5b** were produced in a ratio of 0:0:0, respectively. Additionally, no evidence of starting material conversion was observed.

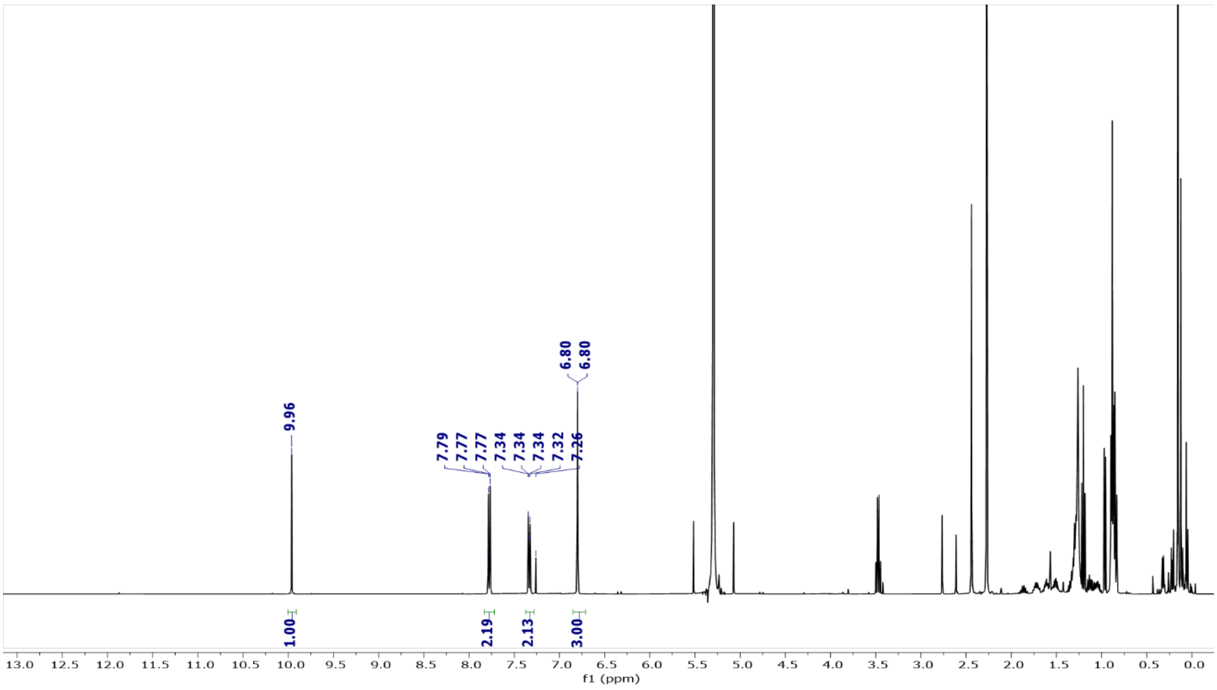

Figure S19 – **^{1}H NMR (400 MHz, $CDCl_3$, 293 K)** crude spectrum of the reaction mixture using optimized reaction conditions.

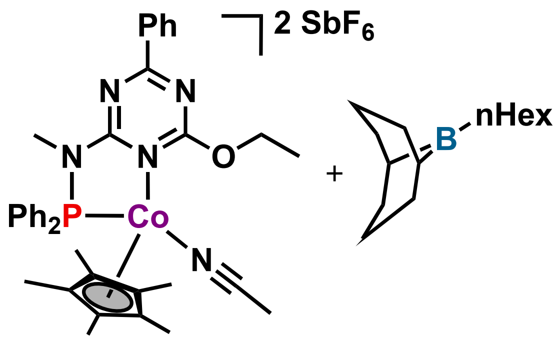

9

System **9** underwent testing as a catalyst in accordance with the general procedure for (*Z*)-silyl enol ether synthesis. Subsequently, product distribution was determined through NMR analysis:

3b, **4b**, and **5b** were produced in a ratio of 80:17:00, respectively. Moreover, a conversion of 97% was achieved.

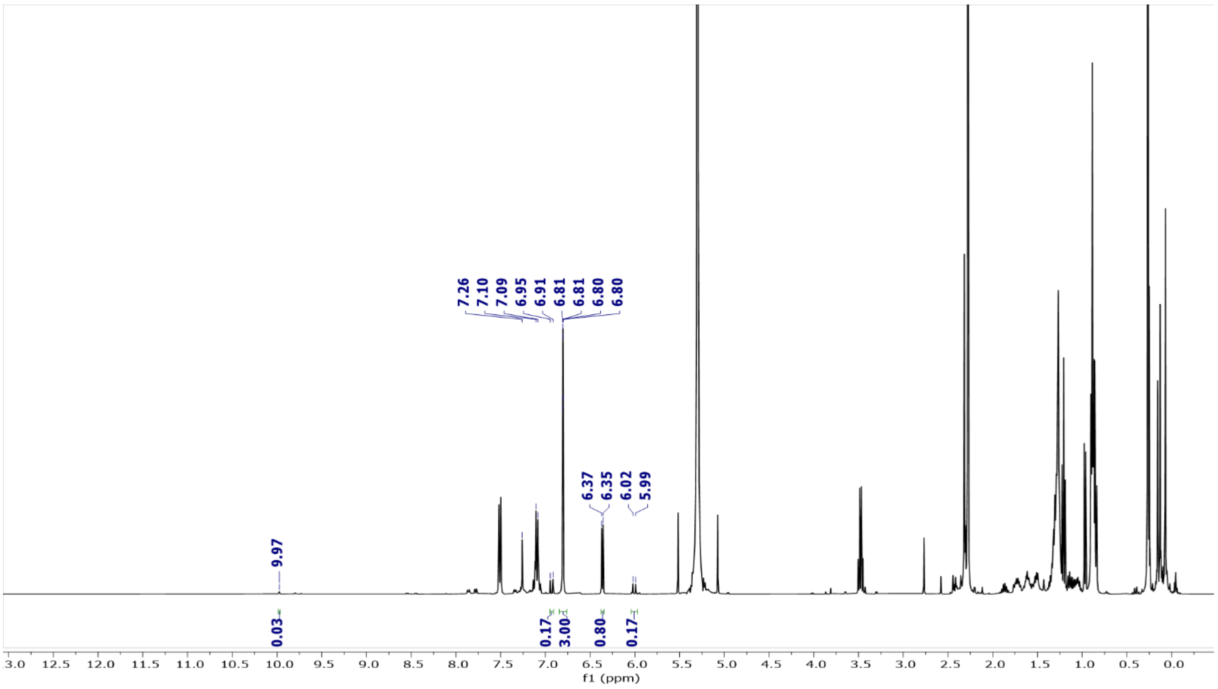

Figure S20 – **^{1}H NMR (400 MHz, CDCl$_3$, 293 K)** crude spectrum of the reaction mixture using optimized reaction conditions.

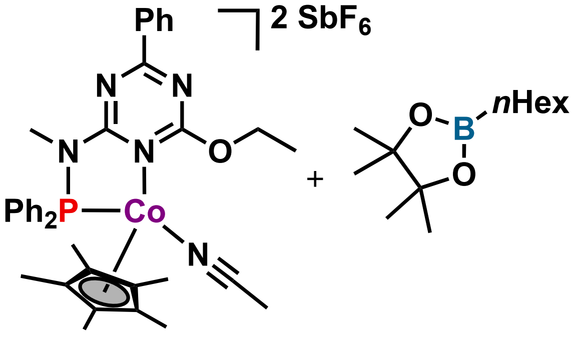

10

System **10** underwent testing as a catalyst in accordance with the general procedure for (*Z*)-silyl enol ether synthesis. Subsequently, product distribution was determined through NMR analysis:

3b, **4b**, and **5b** were produced in a ratio of 62:23:00, respectively. Moreover, a conversion of 85% was achieved.

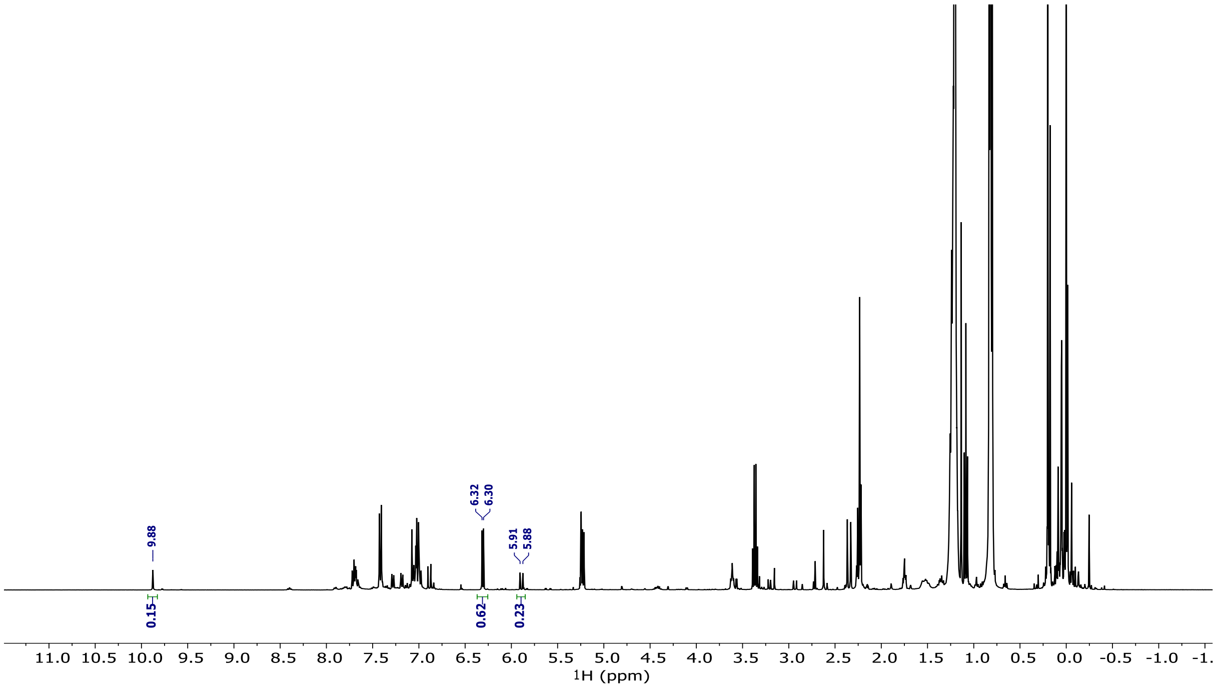

Figure S21 – **^{1}H NMR (400 MHz, $CDCl_3$, 293 K)** crude spectrum of the reaction mixture using optimized reaction conditions.

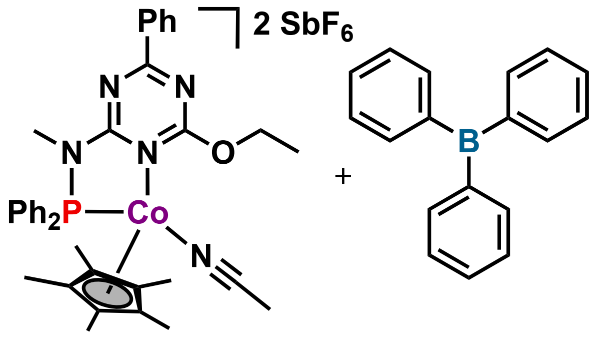

11

System **11** underwent testing as a catalyst in accordance with the general procedure for (*Z*)-silyl enol ether synthesis. Subsequently, product distribution was determined through NMR analysis:

3b, **4b**, and **5b** were produced in a ratio of 77:16:00, respectively. Moreover, a conversion of 93% was achieved.

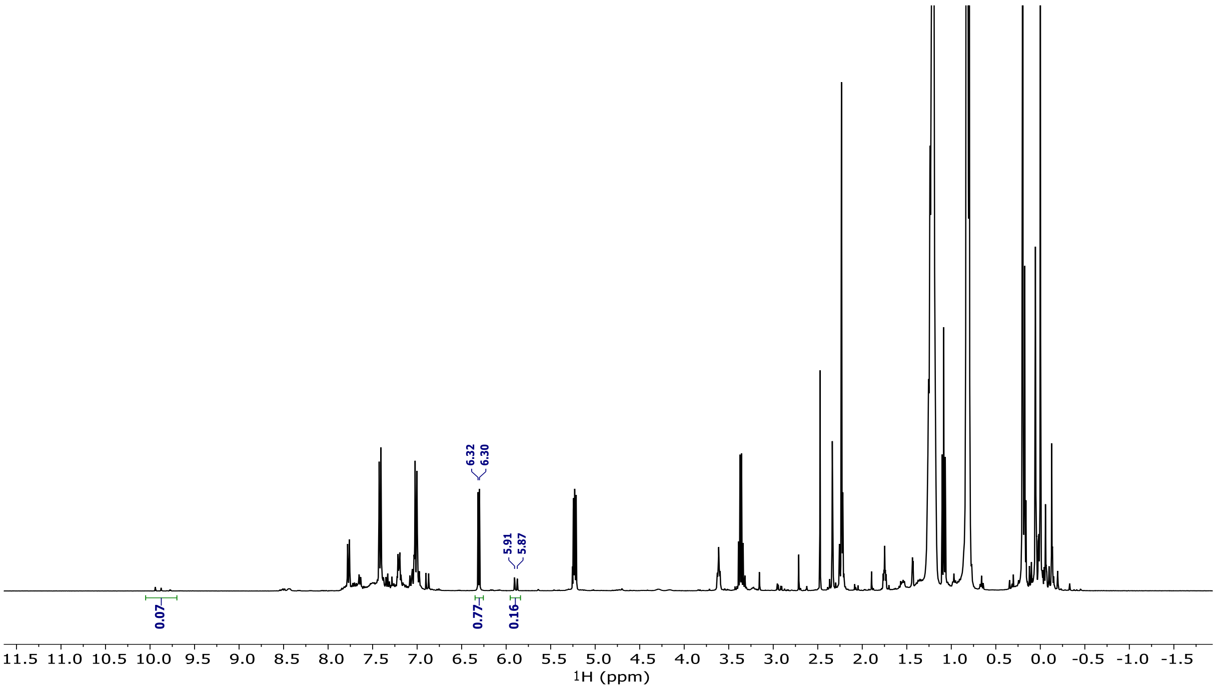

Figure S22 – **^{1}H NMR (400 MHz, $CDCl_3$, 293 K)** crude spectrum of the reaction mixture using optimized reaction conditions.

$Fe(OTf)_2$

12

Complex **12** underwent testing as a catalyst in accordance with the general procedure for (*Z*)-silyl enol ether synthesis. Subsequently, product distribution was determined through NMR analysis:

3b, **4b**, and **5b** were produced in a ratio of 0:0:0, respectively. Moreover, a conversion of 66% was achieved.

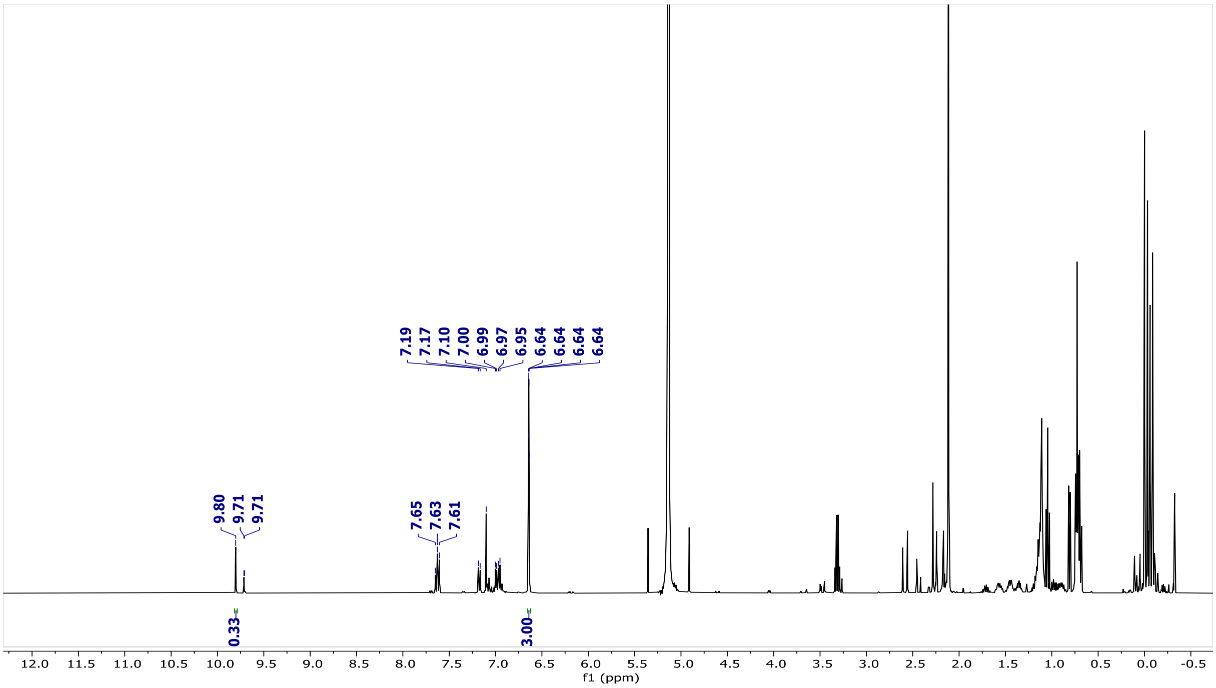

Figure S23 – **^{1}H NMR (400 MHz, $CDCl_3$, 293 K)** crude spectrum of the reaction mixture under optimized reaction conditions.

Fe(OTf)₃

13

Complex **13** underwent testing as a catalyst in accordance with the general procedure for (*Z*)-silyl enol ether synthesis. Subsequently, product distribution was determined through NMR analysis:

3b, **4b**, and **5b** were produced in a ratio of 18:4:25, respectively. Moreover, a conversion of >99% was achieved.

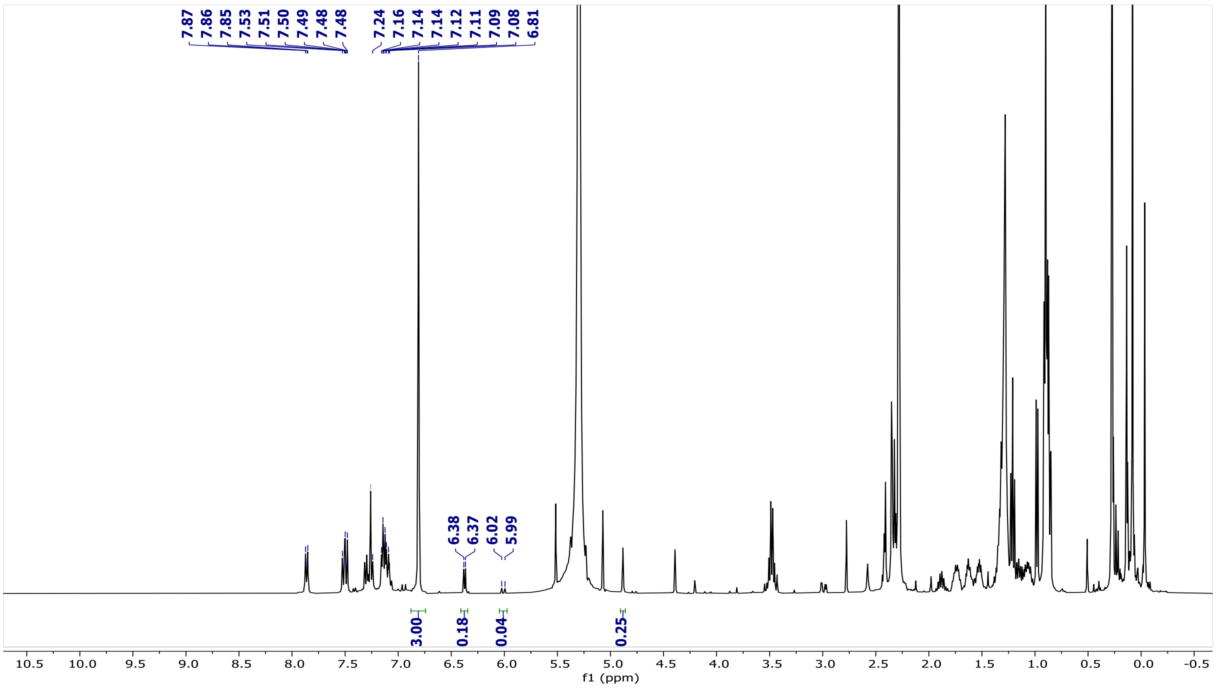

Figure S24 – **^{1}H NMR (400 MHz, $CDCl_3$, 293 K)** crude spectrum of the reaction mixture under optimized reaction conditions.

**FeBr$_3$**<br><br>**14**	Complex **14** underwent testing as a catalyst in accordance with the general procedure for (*Z*)-silyl enol ether synthesis. Subsequently, product distribution was determined through NMR analysis:<br><br>**3b**, **4b**, and **5b** were produced in a ratio of 19:4:0, respectively. Moreover, a conversion of >99% was achieved.

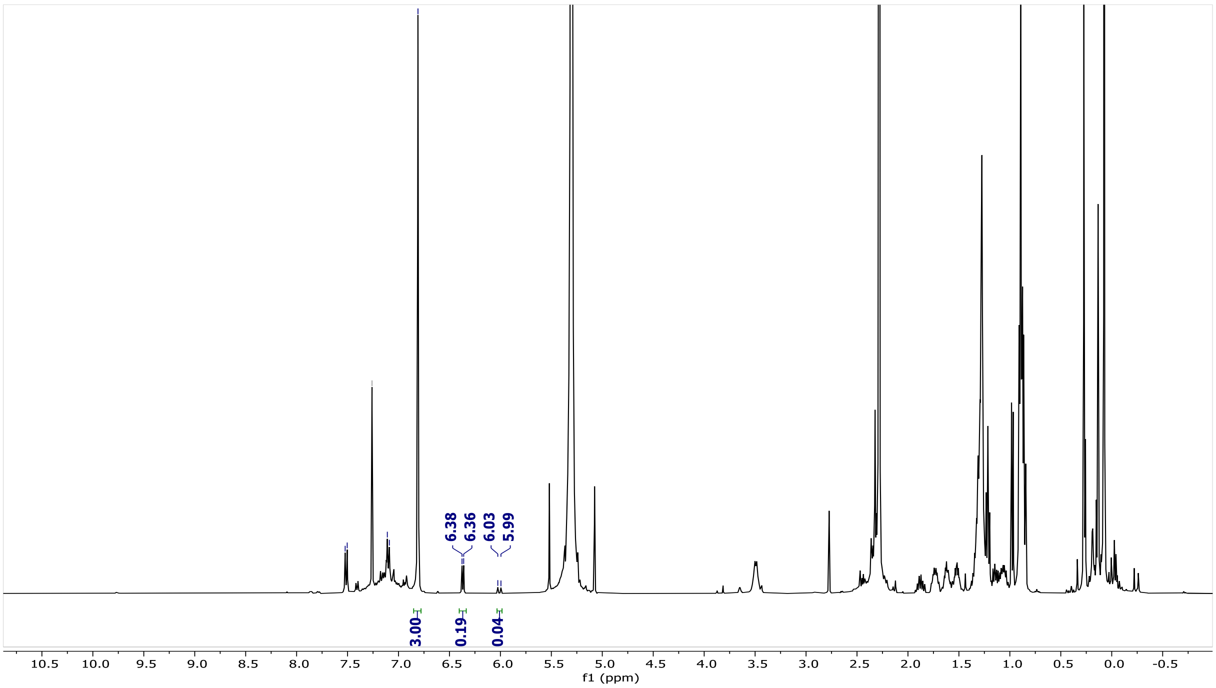

Figure S25 – **^{1}H NMR (400 MHz, CDCl$_3$, 293 K)** crude spectrum of the reaction mixture under optimized reaction conditions.

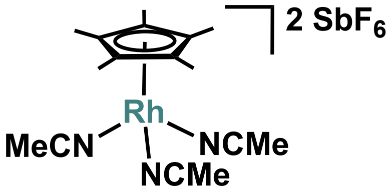

15

Complex **15** underwent testing as a catalyst in accordance with the general procedure for (*Z*)-silyl enol ether synthesis. Subsequently, product distribution was determined through NMR analysis:

3b, **4b**, and **5b** were produced in a ratio of 56:12:0, respectively. Moreover, a conversion of >99% was achieved.

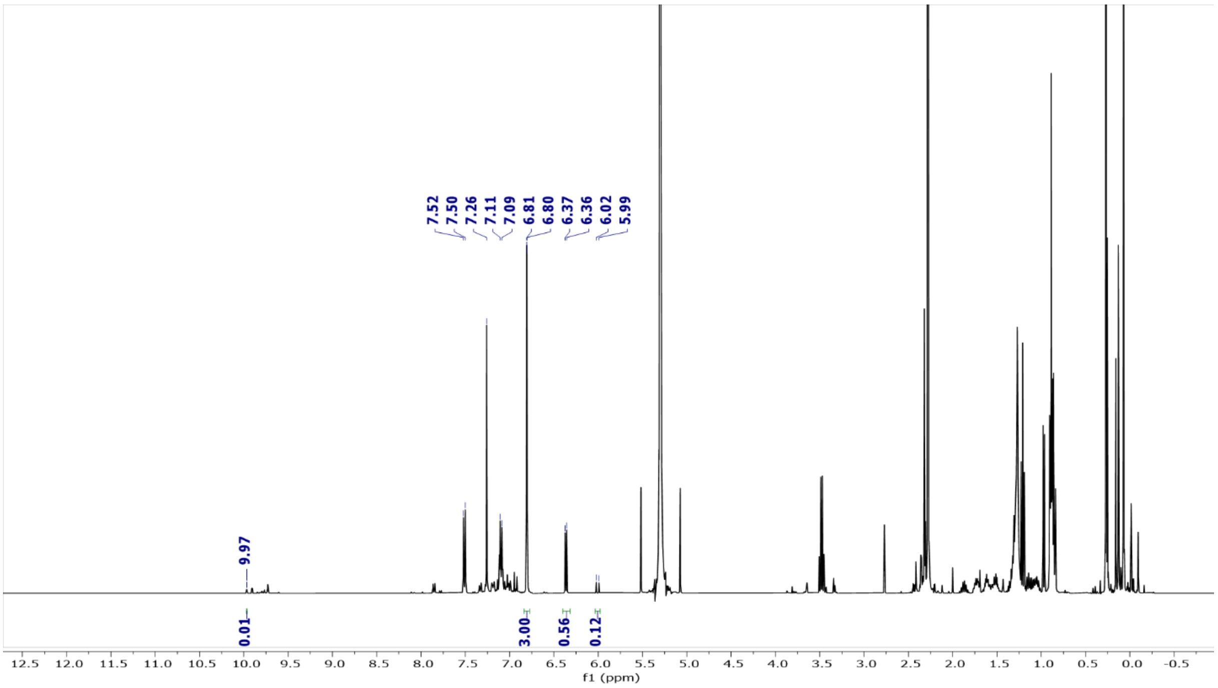

Figure S26 – **^{1}H NMR (400 MHz, $CDCl_3$, 293 K)** crude spectrum of the reaction mixture using optimized reaction conditions.

Ni(diglyme)Br$_2$

16

Complex **16** underwent testing as a catalyst in accordance with the general procedure for (*Z*)-silyl enol ether synthesis. Subsequently, product distribution was determined through NMR analysis:

3b, **4b**, and **5b** were produced in a ratio of 0:0:0, respectively. Moreover, a conversion of 37% was achieved.

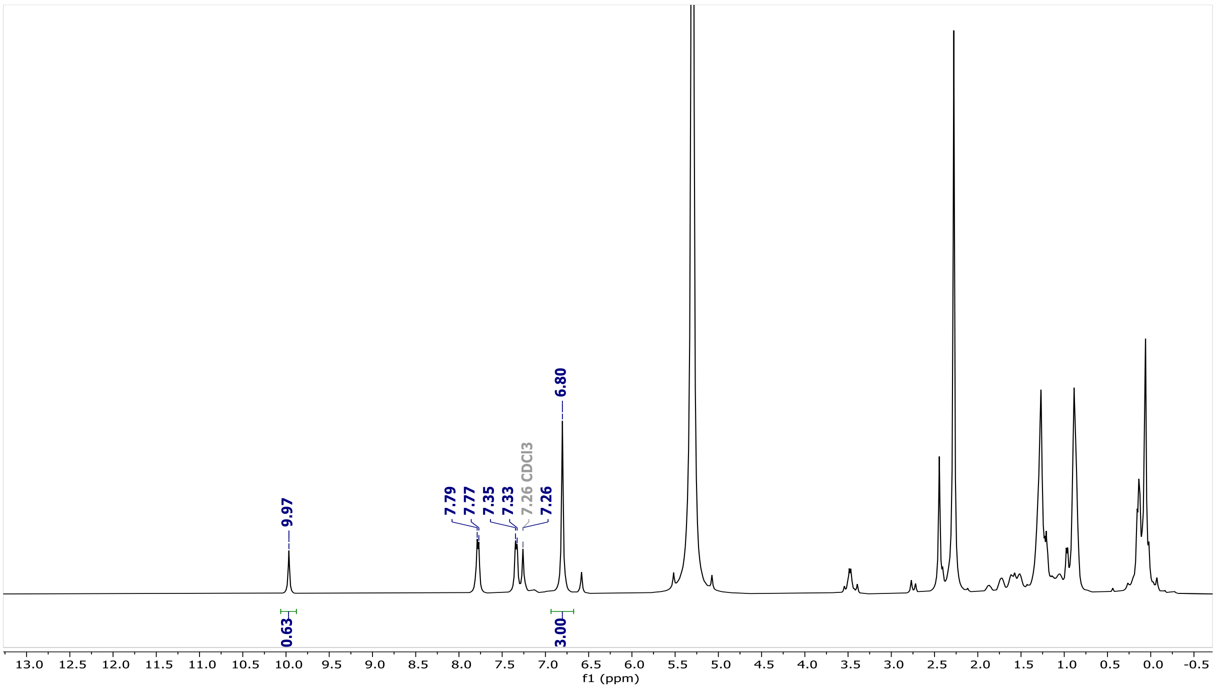

Figure S27 – **^{1}H NMR (400 MHz, $CDCl_3$, 293 K)** crude spectrum of the reaction mixture under optimized reaction conditions.

CuCl

17

Complex **17** underwent testing as a catalyst in accordance with the general procedure for (*Z*)-silyl enol ether synthesis. Subsequently, product distribution was determined through NMR analysis:

3b, **4b**, and **5b** were produced in a ratio of 0:0:0, respectively. Moreover, a conversion of 46% was achieved.

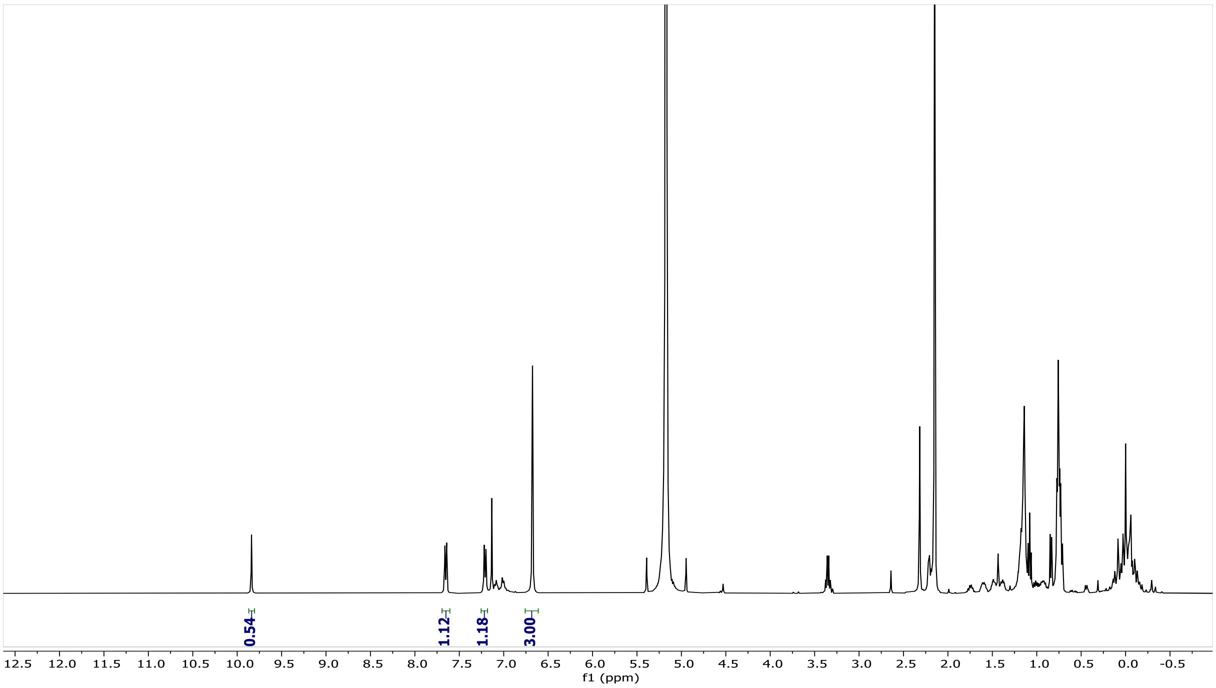

Figure S28 – **^{1}H NMR (400 MHz, $CDCl_3$, 293 K)** crude spectrum of the reaction mixture under optimized reaction conditions.

$Sn(OTf)_2$

18

Complex **18** underwent testing as a catalyst in accordance with the general procedure for (*Z*)-silyl enol ether synthesis. Subsequently, product distribution was determined through NMR analysis:

3b, **4b**, and **5b** were produced in a ratio of 58:14:0, respectively. Moreover, a conversion of >99% was achieved.

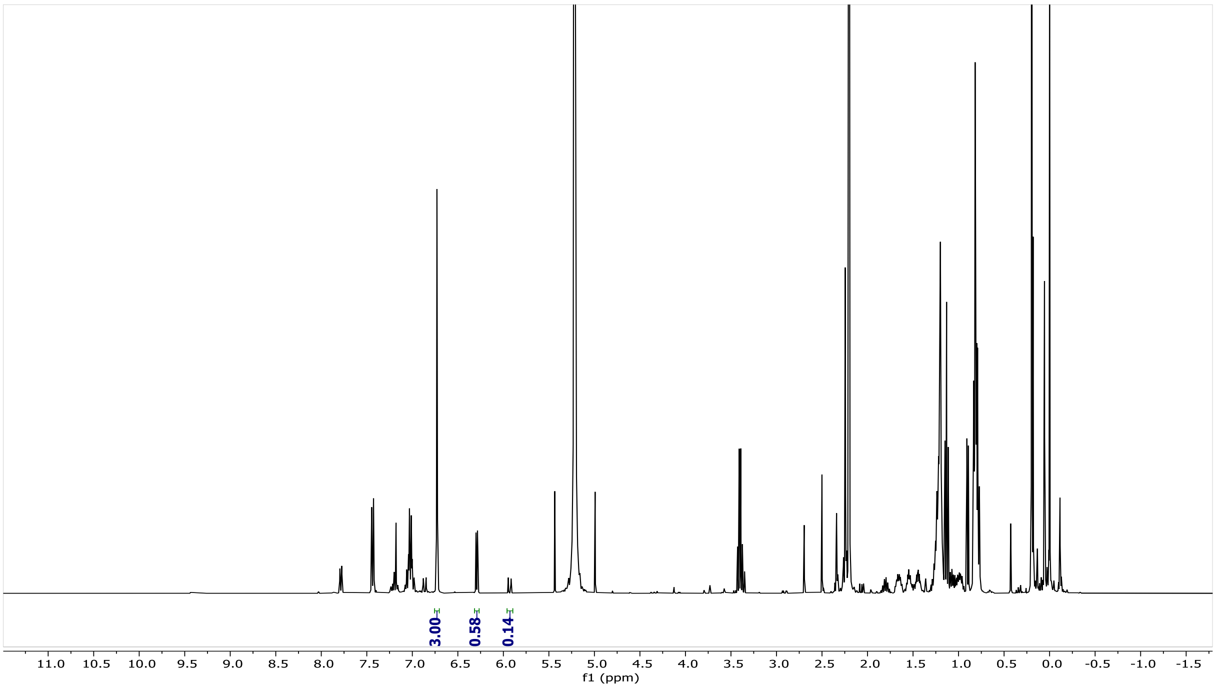

Figure S29 – **^{1}H NMR (400 MHz, $CDCl_3$, 293 K)** crude spectrum of the reaction mixture under optimized reaction conditions.

Pd(OAc)$_2$

19

Complex **19** underwent testing as a catalyst in accordance with the general procedure for (*Z*)-silyl enol ether synthesis. Subsequently, product distribution was determined through NMR analysis:

3b, **4b**, and **5b** were produced in a ratio of 0:0:0, respectively. Moreover, a conversion of 0% was achieved.

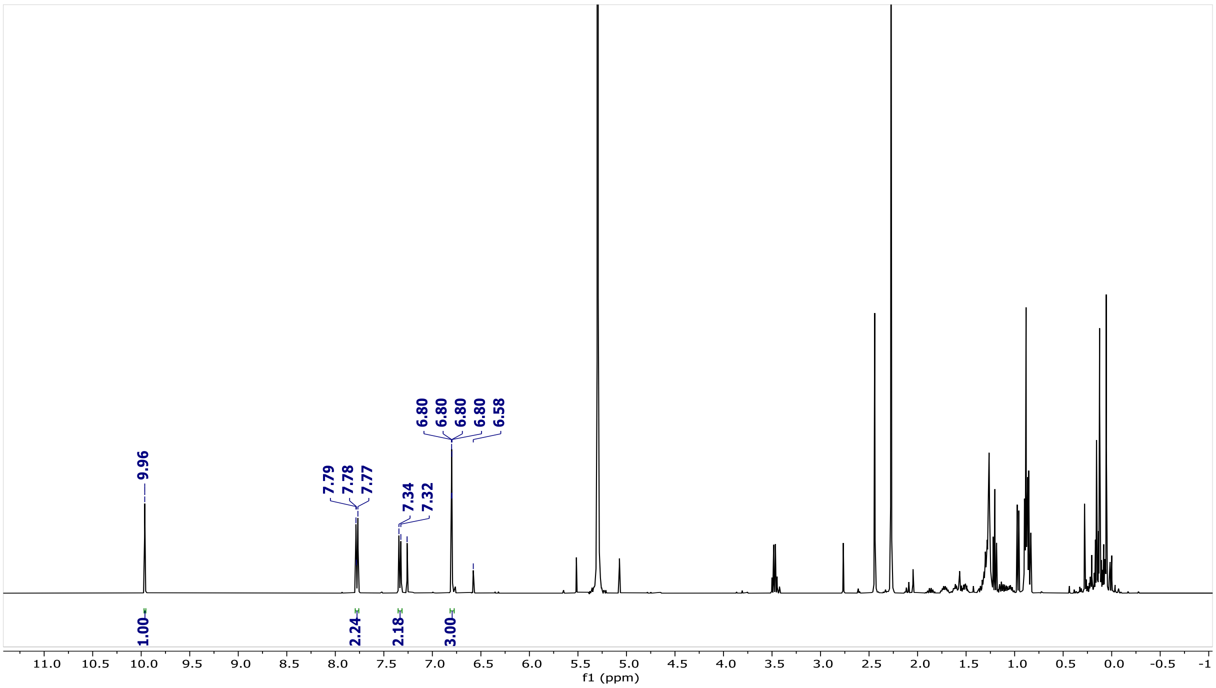

Figure S30 – **^{1}H NMR (400 MHz, $CDCl_3$, 293 K)** crude spectrum of the reaction mixture under optimized reaction conditions.

$AlCl_3$

20

Complex **20** underwent testing as a catalyst in accordance with the general procedure for (*Z*)-silyl enol ether synthesis. Subsequently, product distribution was determined through NMR analysis:

3b, **4b**, and **5b** were produced in a ratio of 25:4:9, respectively. Moreover, a conversion of >99% was achieved.

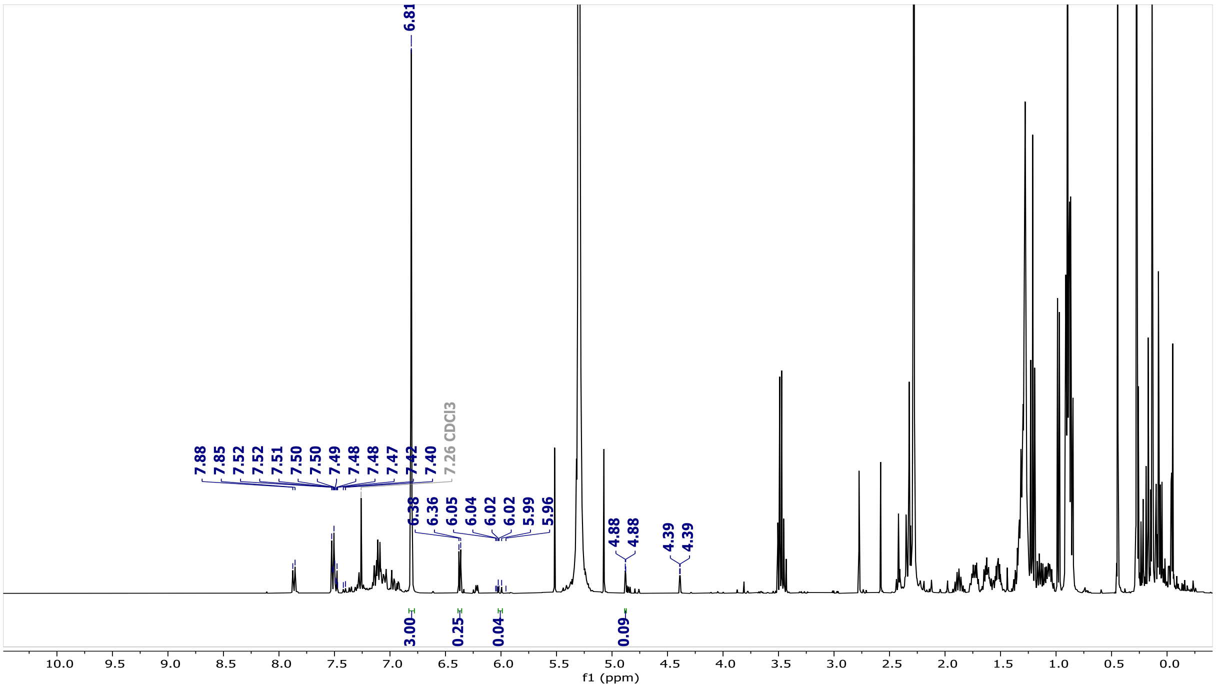

Figure S31 – **^{1}H NMR (400 MHz, $CDCl_3$, 293 K)** crude spectrum of the reaction mixture under optimized reaction conditions.

$ZrCl_4$

21

Complex **21** underwent testing as a catalyst in accordance with the general procedure for (*Z*)-silyl enol ether synthesis. Subsequently, product distribution was determined through NMR analysis:

3b, **4b**, and **5b** were produced in a ratio of 7:1:0, respectively. Moreover, a conversion of >99% was achieved.

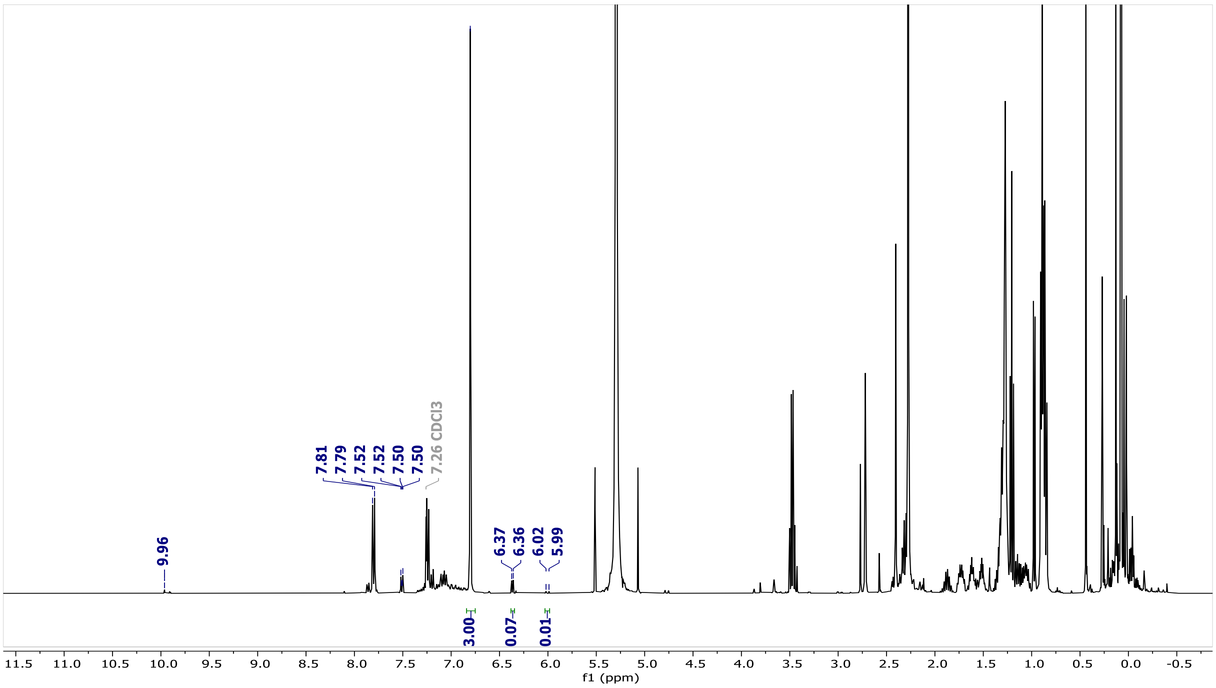

Figure S32 – **^{1}H NMR (400 MHz, $CDCl_3$, 293 K)** crude spectrum of the reaction mixture under optimized reaction conditions.

$IrCl_3 \cdot xH_2O$

22

Complex **22** underwent testing as a catalyst in accordance with the general procedure for (*Z*)-silyl enol ether synthesis. Subsequently, product distribution was determined through NMR analysis:

3b, **4b**, and **5b** were produced in a ratio of 0:0:0, respectively. Moreover, a conversion of 0% was achieved.

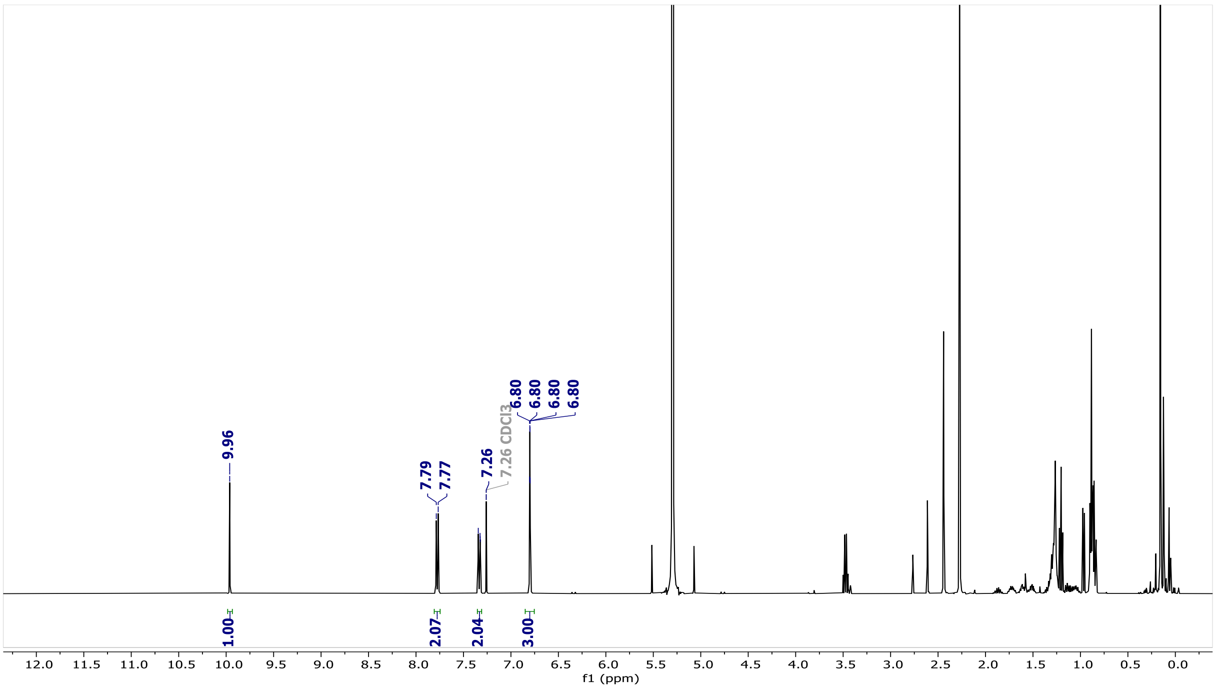

Figure S33 – **^{1}H NMR (400 MHz, $CDCl_3$, 293 K)** crude spectrum of the reaction mixture under optimized reaction conditions.

5.2. Investigating the Properties of the Lewis Acidic Boron Center in 1

5.2.1. Catalytic Reaction in the Presence of MeOH

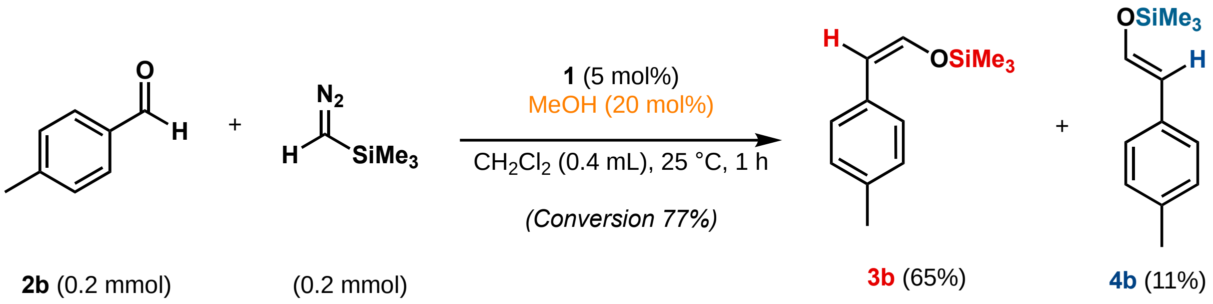

Procedure: In an oven-dried 7 mL reaction vial, compound **1** (12.6 mg, 0.01 mmol) and methanol (4 µL, 0.1 mmol) were combined in a CH_2Cl_2 (0.4 mL) solution. This mixture was stirred for 1 h at room temperature. Subsequently, *p*-tolyl aldehyde (23.6 µL, 0.2 mmol) was introduced to the same vial, followed by the dropwise addition of Me_3SiCHN_2 (100 µL, 0.2 mmol). Stirring continued for an additional 1 h at 25 °C. Upon reaction completion, mesitylene (27.8 µL, 0.2 mmol), serving as an internal standard, was added, and an aliquot was extracted to analyze the crude reaction mixture. The resulting crude ^1H NMR indicated reduced reactivity compared to reactions under optimized conditions. This observation might be attributed to the formation of a MeOH-borane adduct, which could potentially affect the binding of the substrate aldehyde to the boron arm, thereby influencing reactivity.

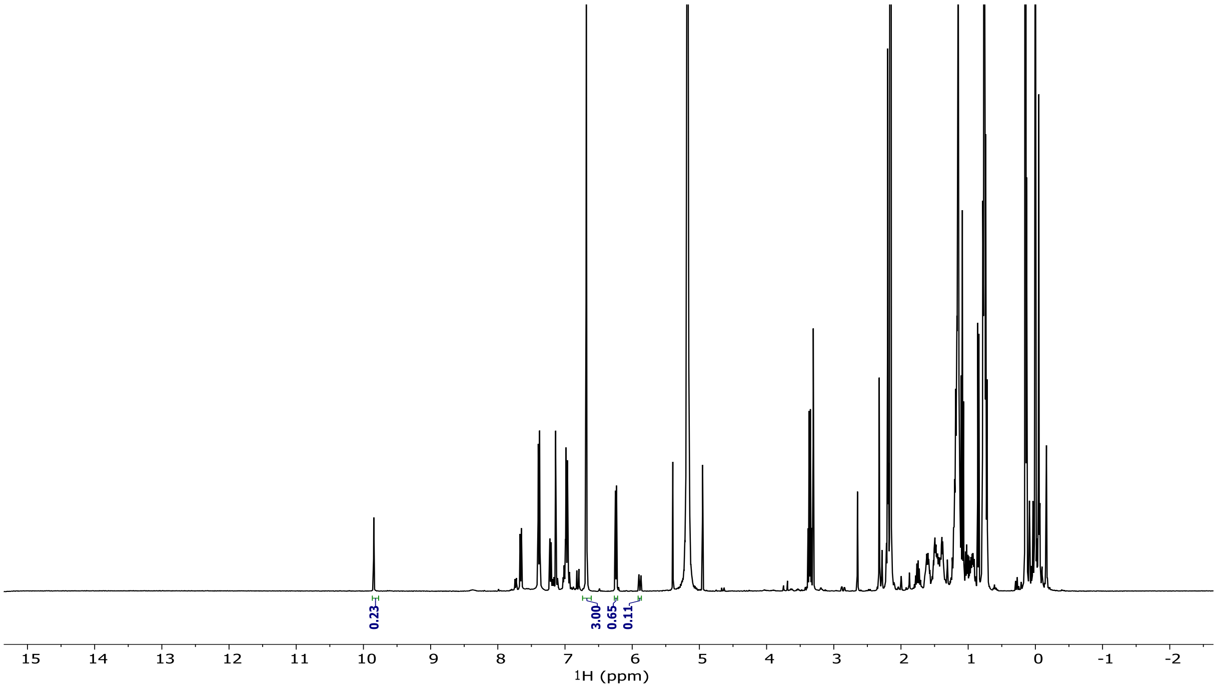

Figure S34 – **^1H NMR (400 MHz, $CDCl_3$, 293 K)** spectrum of the crude reaction mixture in the presence of 0.1 mmol of MeOH.

5.2.2. Catalytic Reaction in the Presence of Et_3N

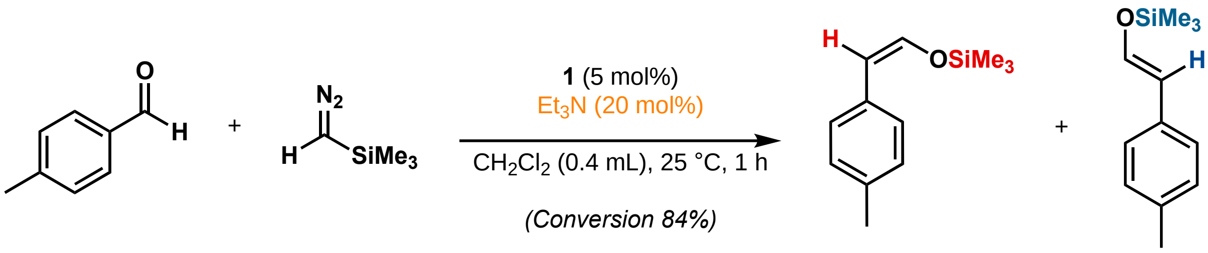

Procedure: In an oven-dried 7 mL reaction vial under argon, compound **1** (12.6 mg, 0.01 mmol) and Et_3N (2 µL, 0.02 mmol) were combined in a CH_2Cl_2 (0.4 mL) solution. *p*-tolyl aldehyde (23.6 µL, 0.2 mmol) was introduced into the same vial, followed by the dropwise addition of Me_3SiCHN_2 (100 µL, 0.2 mmol). The resultant mixture underwent stirring for 1 h at 25 ºC. Subsequent to this interval, mesitylene (27.8 µL, 0.2 mmol) was incorporated as an internal standard. An aliquot of the mixture was then transferred to an NMR tube. Using ^{1}H NMR, the crude reaction mixture's yield and conversion were monitored. Analysis revealed that the formation of the silyl enol ether was somewhat inhibited. This can be attributed to the Lewis acidic nature of the boron arm; Et_3N may neutralize it by forming an LA-LB adduct, thereby impacting the efficacy of the boron arm as the reaction progresses.

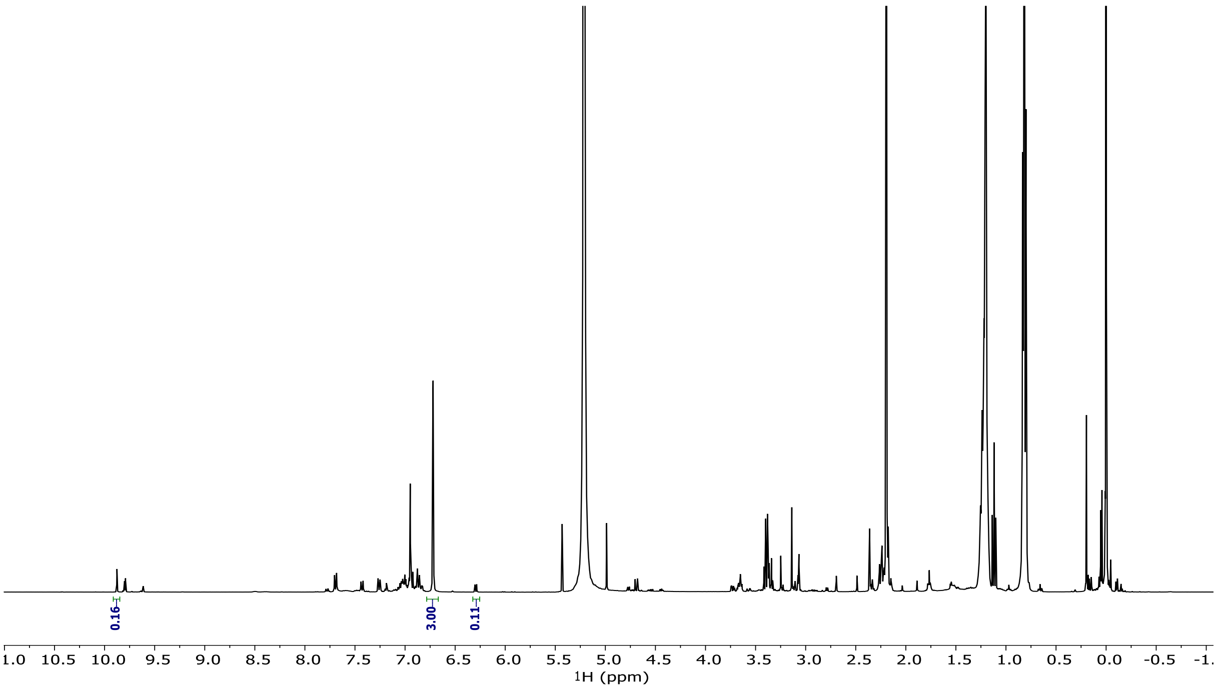

Figure S35 – **^{1}H NMR (400 MHz, $CDCl_3$, 293 K)** spectrum of the crude reaction mixture in the presence of 10 mol% NEt_3.

5.3. Additive Interaction with Boron Atom in Secondary Coordination Sphere

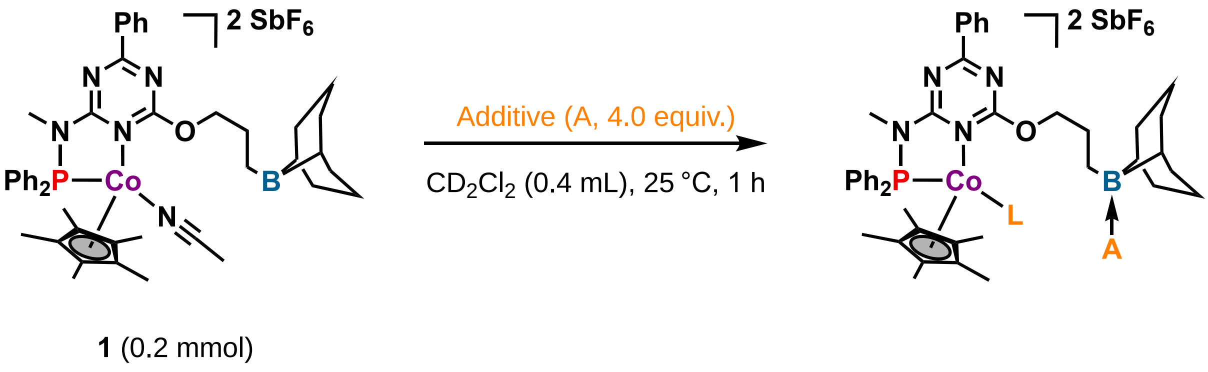

Procedure: In an oven-dried 7 mL reaction vial within a glove box, complex **1** (30.0 mg, 0.03 mmol) and Additive (6.0 equiv.) were combined in a CH_2Cl_2-d_2 (0.4 mL) solution. The mixture underwent stirring for 1 h at 25 ºC. Upon completion of the reaction, the entire solution was decanted into an NMR tube under an argon atmosphere, then sealed with parafilm prior to NMR analysis. Presented below are the stacked ^{11}B NMR spectra of the different crude reaction mixtures.

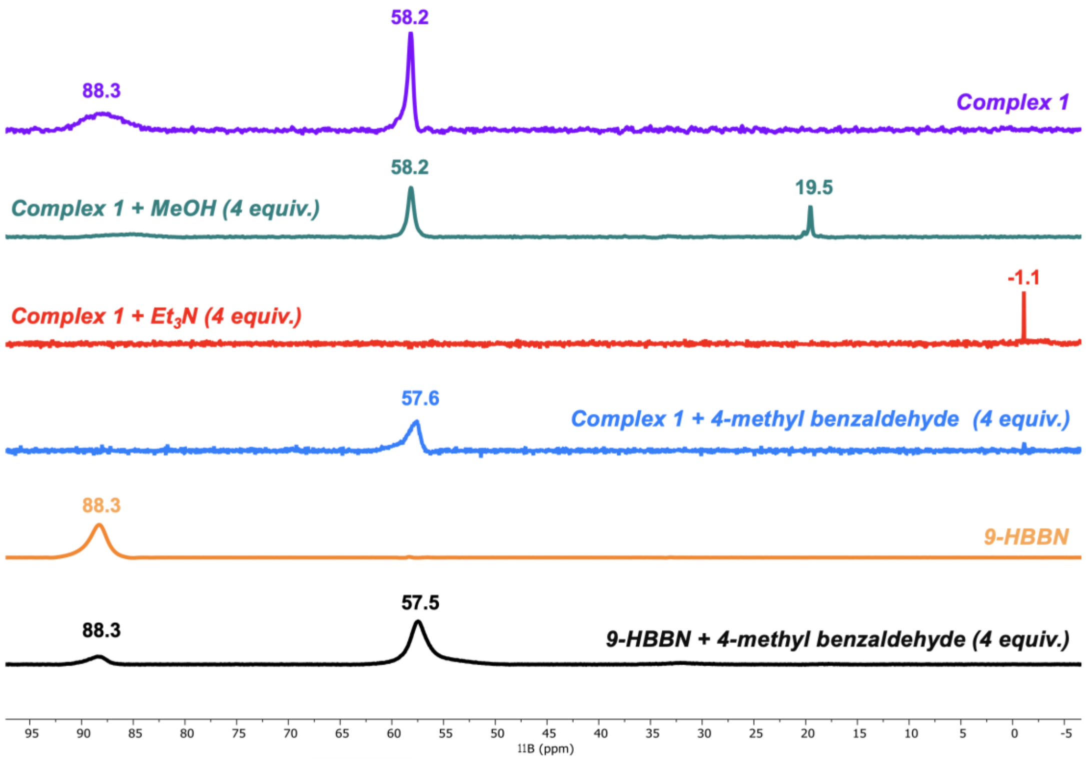

Figure S36 –**^{11}B NMR (126 MHz, CD_2Cl_2, 293 K)** stacked spectra of the crude reaction mixtures in the presence of 6.0 equiv of additives with respect to complex **1**.

5.4. Exploring the Formation of Hypothetical Metal Carbenes

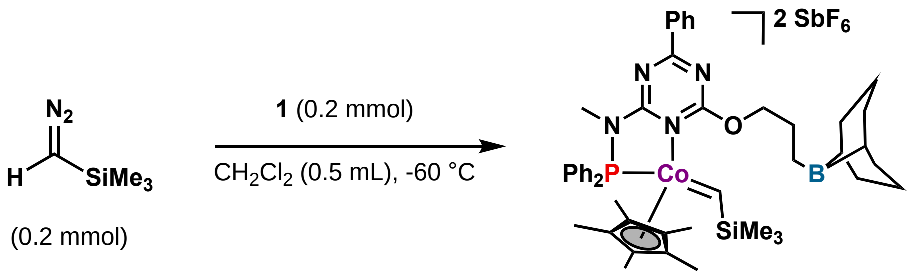

1’

(Tentative molecular structure)

In a glovebox, under an argon atmosphere, [CoCp*(MeCN)(**PN**$^{tzn\text{-}B}$)](SbF_6)$_2$ (25.2 mg, 0.02 mmol) was dissolved in 0.5 mL of CH_2Cl_2. The resulting mixture was then removed from the glovebox under argon and cooled to -60°C using an acetone/liquid nitrogen bath. Subsequently, Me_3SiCHN_2 (10 µL, 0.02 mmol) and Me_3CN (ca. 0.1 mL) were rapidly added to the sample under argon and directly injected into the mass spectrometer. This analysis confirmed the high-resolution molecular mass, consistent with the expected cobalt carbene **1’**.

HRMS (ESI): Calcd. For $C_{47}H_{63}BCoN_4OPSi$ $[M]^{2+}$: 414.19614; Found: 414.19617.

```
Mass to be matched (m/z): 414.19617   charge: 2

Mass tolerance: ±0.005
restriction of atom numbers:
       B        C       Co        H        N        O        P       Si
     1-1     1-60      1-1    1-100      1-4      1-3      1-2      1-1
Number of calculated formulas: 3
Formula                        Diff. (ppm)     theor. m/z
C47 H63 B Co N4 O P Si             0.07        414.19614
C49 H65 B Co N O2 P Si            -1.55        414.19681
C45 H68 B Co N2 O2 P2 Si          -1.92        414.19697
```

Elektrospray-ionisation pos. ions

Characteristicial ions(doubly charged):
310.10 , 414.196 , 434.71

Additionally characteristical ions:
913

In this measurement there are no references
for your wanted product

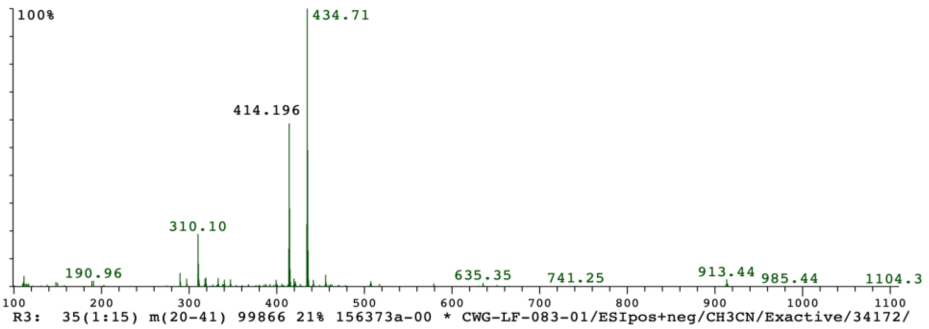

R3: 35(1:15) m(20-41) 99866 21% 156373a-00 * CWG-LF-083-01/ESIpos+neg/CH3CN/Exactive/34172/

Elektrospray-ionisation neg. ions

Characteristicial ions:
235 = [Sb1F6]-

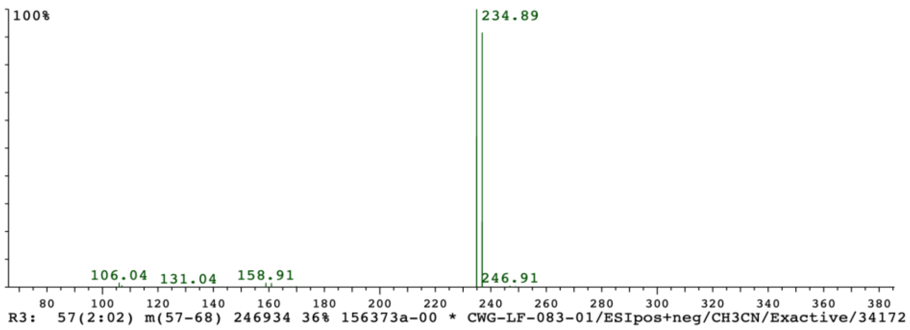

R3: 57(2:02) m(57-68) 246934 36% 156373a-00 * CWG-LF-083-01/ESIpos+neg/CH3CN/Exactive/34172

Figure S37 – **ESI-HRMS** spectra post-reaction of **1** with TMSCHN$_2$, indicating the potential formation of **1’**.

5.5. Investigation of Alkenyl Migration and 1,2-Brook Rearrangement

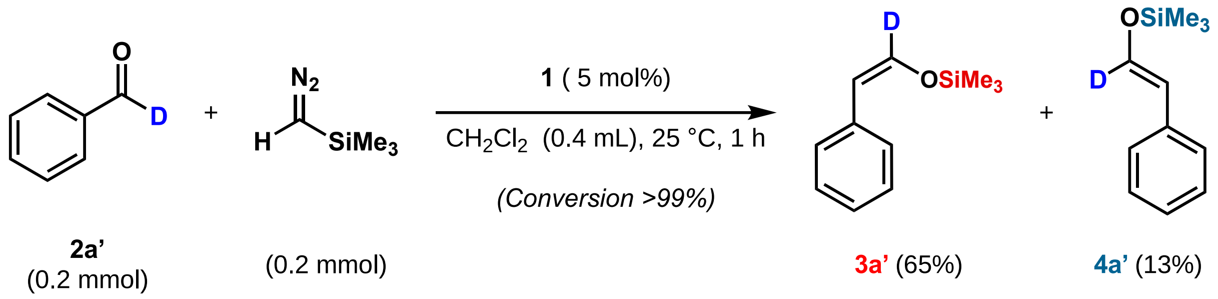

Procedure: Benzaldehyde-α-d_1 (20.6 µL, 0.2 mmol) and catalyst **1** (12.6 mg, 0.01 mmol, 5 mol%) were introduced into a 7 mL reaction vial within a glove box under an argon atmosphere. Subsequently, 0.4 mL of CH_2Cl_2 was added. This was followed by the dropwise addition of Me_3SiCHN_2 (100 µL, 0.2 mmol) and the reaction mixture was stirred for 1 h at room temperature. Upon completion of the reaction, mesitylene (27.8 µL, 0.2 mmol), serving as an internal standard, was added to the same vial, from which an aliquot was subsequently extracted. The crude reaction mixture was then analyzed using ^1H, $^{13}C\{^1H\}$ ^1D NMR and ^1H-^1H COSY, ^1H-^{13}C HMBC, ^1H-^{13}C HSQC NMR spectroscopy. H-D exchange studies indicated that the formation of product **3a'** was dominant over **4a'**.

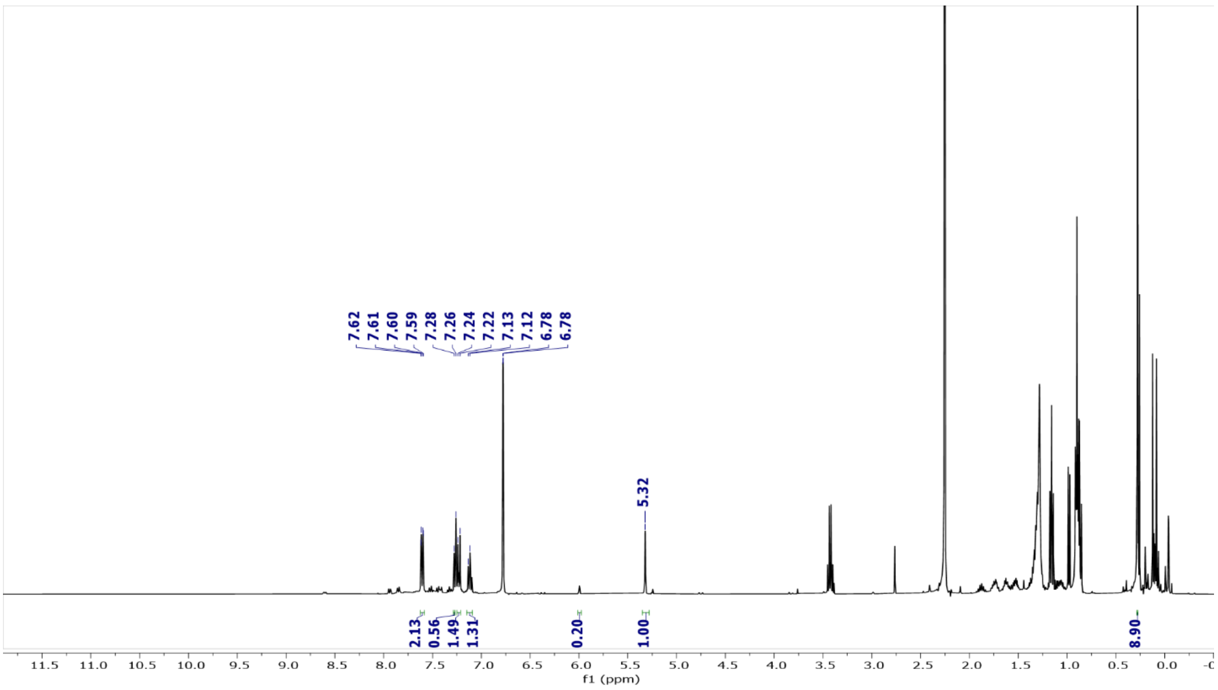

Figure S38 – **^1H NMR (400 MHz, CD_2Cl_2, 293 K)** spectrum of deuterium exchange studies using benzaldehyde-α-d_1 (0.2 mmol) and $TMSCHN_2$ (0.2 mmol) at optimized reaction condition.

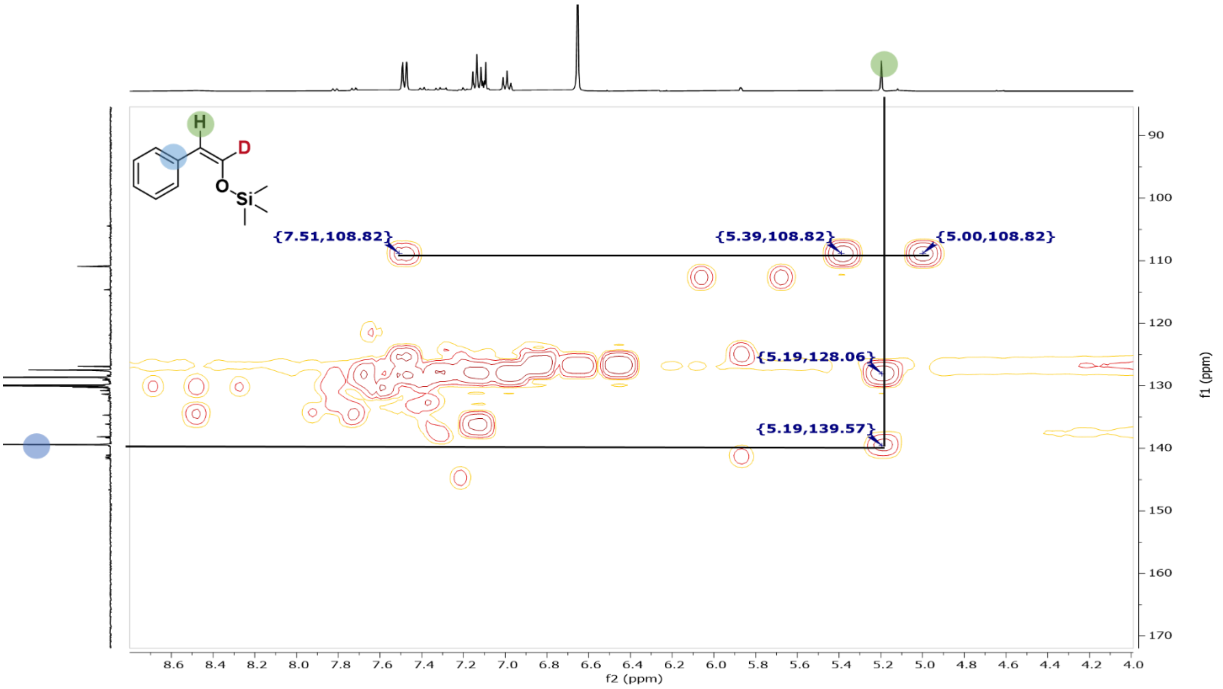

Figure S39 – **^{1}H – ^{13}C HSQC NMR (400 MHz, CD_2Cl_2, 293 K)** spectrum of deuterium exchange studies using benzaldehyde-α-d1 (0.2 mmol) and $TMSCHN_2$ (0.2 mmol) at optimized reaction conditions.

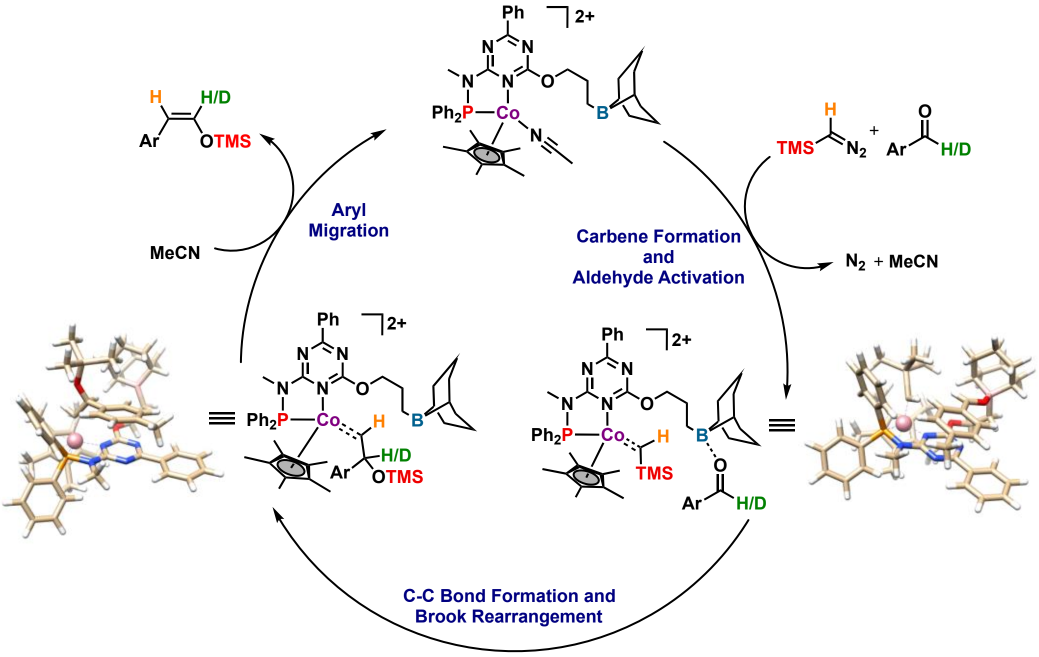

Scheme S1 – The deuteration experiment demonstrates that the reaction pathway involves a 1,4-Brook rearrangement,[6] characterized by the migration of $SiMe_3$ to oxygen. This is then followed by the migration of an aryl group to the vicinal carbon center.[5]

5.6. Tempo Experiment

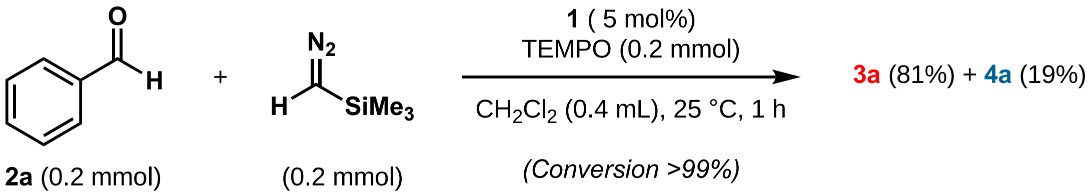

Procedure: In an oven-dried 7 mL reaction vial under argon, compound **1** (12.6 mg, 0.01 mmol) and TEMPO (31.25 mg, 0.2 mmol) were combined in a CH_2Cl_2 (0.4 mL) solution. Benzaldehyde (20.4 µL, 0.2 mmol) was introduced into the same vial, followed by the dropwise addition of Me_3SiCHN_2 (100 µL, 0.2 mmol). The resultant mixture underwent stirring for 1 h at 25 ºC. Subsequent to this interval, mesitylene (27.8 µL, 0.2 mmol) was incorporated as an internal standard. An aliquot of the mixture was then transferred to an NMR tube. Using ^{1}H NMR, the crude reaction mixture's yield and conversion were monitored.

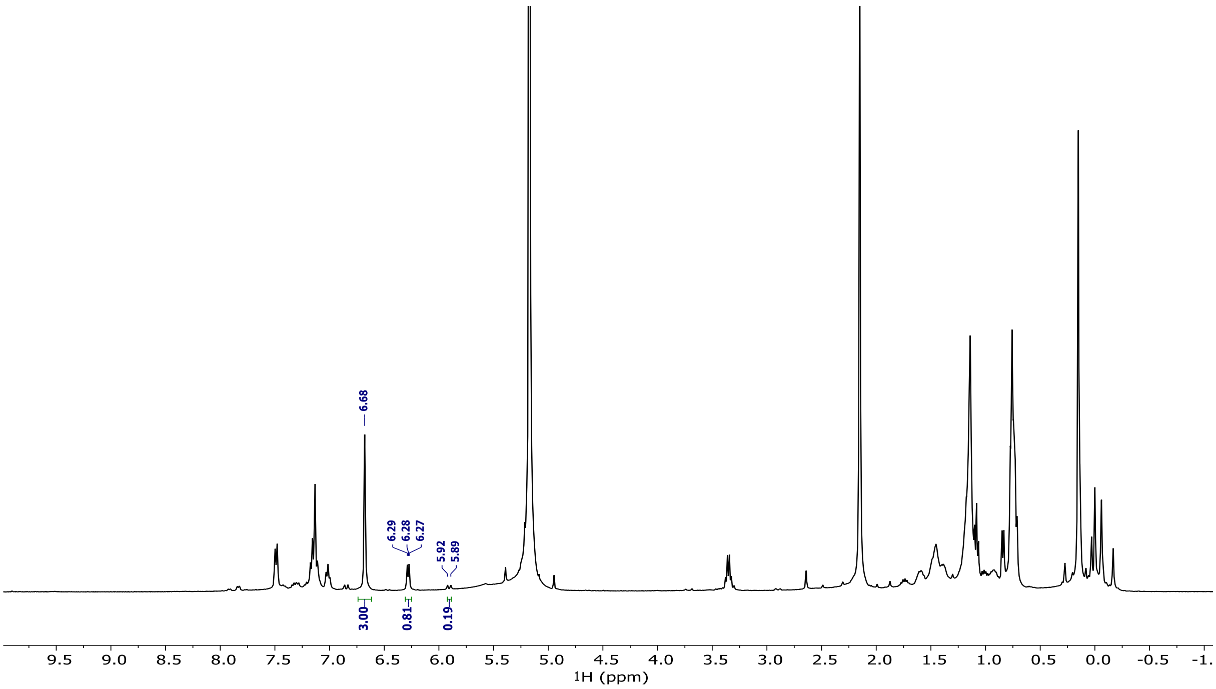

Figure S40 – **^{1}H NMR (400 MHz, CD_2Cl_2, 293 K)** spectrum of crude reaction mixture.

5.7. Exploring Product Formation and Intermediate Generation from Mixtures of Complex 1 and Me_3SiCHN_2

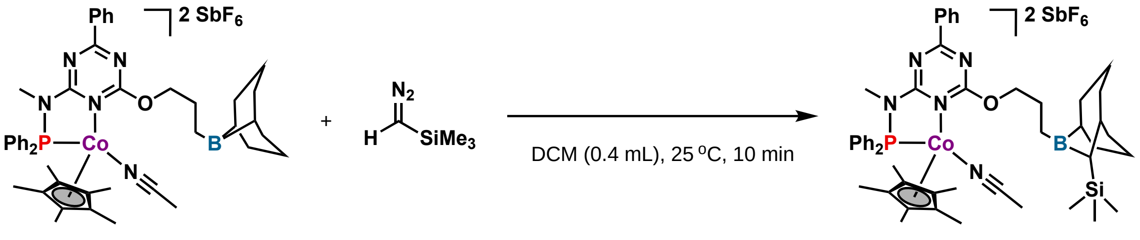

1
(0.03 mmol)
(0.03 mmol)
1”

Under an argon atmosphere within a glovebox, complex **1** (25.2 mg, 0.02 mmol) was mixed with Me_3SiCHN_2 (10 µL, 0.02 mmol) in 1 mL of CH_2Cl_2. Stirring the mixture for 30 minutes led to the full consumption of Me_3SiCHN_2. The solvent was then evaporated under vacuum, and the residual mixture underwent two pentane washes. After drying, the reaction mixture underwent NMR spectroscopy analysis, specifically ^{1}H, ^{1}H–^{1}H COSY, ^{1}H-^{29}Si HMBC, and ^{1}H-^{13}C HMBC. This analysis identified the presence of a new species, denoted as **1”**. The ^{1}H-^{29}Si HMBC exhibited a relationship between the emergent Si species and the proton ^{1}H located at 1.43 (likely the CH proton of the inserted carbon). Furthermore, a pronounced long-range correlation (HMBC) was evident from the TMS-methyl ^{1}H signal. A notable correlation was observed at d_{13C} = 40 ppm, aligning most appropriately with a carbon neighboring the boron. This carbon demonstrated HMBC correlations to aliphatic ^{1}Hs, consistent with the BBN ring positions. The ESI-HRMS analysis further corroborated the formation of species **1”**.[7]

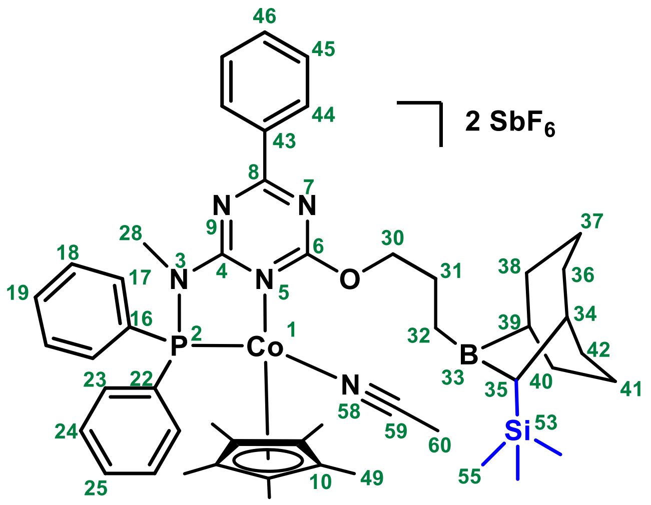

^{1}H NMR (600 MHz, CD_2Cl_2, 298 K) δ = 8.59 (d, J=8.3, 2H, 44), 7.90 - 7.83 (m, 3H, 17, 19), 7.83 - 7.77 (m, 2H, 18), 7.77 - 7.65 (m, 4H, 24, 25, 46), 7.59 (dd, J=11.9, 7.5, 2H, 45), 7.53 - 7.40 (m, 2H, 23), 5.16 (tdd, J=11.0, 11.0, 9.1, 5.4, 1H, 30"), 4.68 (tdd, J=11.0, 11.0, 5.4, 2.6, 1H, 30'), 3.45 (dd, J=5.2, 1.8, 3H, 28), 2.49 - 2.34 (m, 1H, 34), 2.23 - 2.06 (m, 2H, 31', 31"), 2.05 - 1.98 (m, 1H, 39), 1.93 - 1.82 (m, 1H, 37'), 1.76 - 1.73 (m, 3H, 60), 1.72 - 1.55 (m, 7H, 36', 36", 38', 40, 42', 42"), 1.54 - 1.46 (m, 3H, 37", 38", 41"), 1.44 - 1.40 (m, 1H, 35), 1.36 - 1.29 (m, 17H, 32, 49), 1.02 - 0.91 (m, 1H, 41'), 0.15 (s, 9H, 55).

^{13}C NMR (151 MHz, CD_2Cl_2, 298 K) δ = 177.30 (8), 174.49 (d, J=22.1, 4), 172.77 (d, J=2.1, 6), 137.54 (d, J=3.2, 19), 137.43 (d, J=2.1, 46), 136.40 (br s, 17), 135.72 (43), 135.59 (br s, 25), 135.19 (59), 133.25 (d, J=11.9, 18), 133.14 (d), J=11.6, 24), 132.81 (44), 132.48 (d, J=11.5, 23), 131.43 (45), 129.56 (d, J=45.4, 22), 124.10 (d, J=58.3, 16), 106.01 - 104.58 (m, 10), 76.90 (30), 42.64 (br s, 35), 39.96 (d, J=4.8, 28), 37.46 (34), 36.35 - 36.30 (m, 36), 33.62 - 33.51 (m, 42), 33.18-33.04 (m, 39), 31.61 (38), 27.74 - 27.65 (m, 40, 41), 26.97 (m, 31), 26.87-26.79 (m, 32), 24.29 (d, J=1.8, 37), 12.73 (49), 5.42 (60), 4.13 - 3.98 (m, 55).

^{31}P NMR (243 MHz, CD_2Cl_2, 298 K) δ = 114.18 (d, J=26.5, 2).

IR (Diamond – ATR, neat), υ (cm^{-1}): 557.04 (m) 653.61 (s) 708.52 (m) 751.63 (w) 782.97 (m) 835.93 (m) 953.18 (m) 998.76 (w) 1014.63 (w) 1072.09 (w) 1097.52 (w) 1158.28 (w) 1259.40 (w) 1353.99 (m) 1383.44 (m) 1404.52 (w) 1436.16 (w) 1473.44 (m) 1510.08 (m) 1566.20 (m) 2908.92 (br, w).

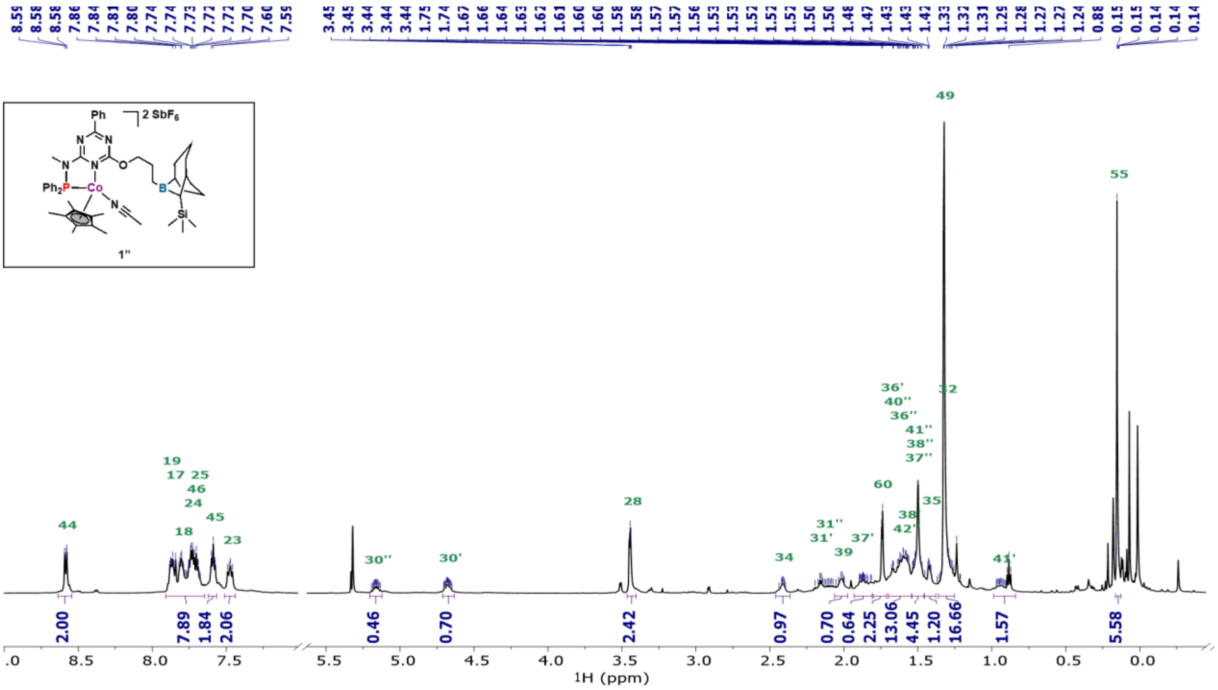

Figure S41 – **^{1}H NMR (600 MHz, CD_2Cl_2, 298 K)** spectrum of complex **1”**.

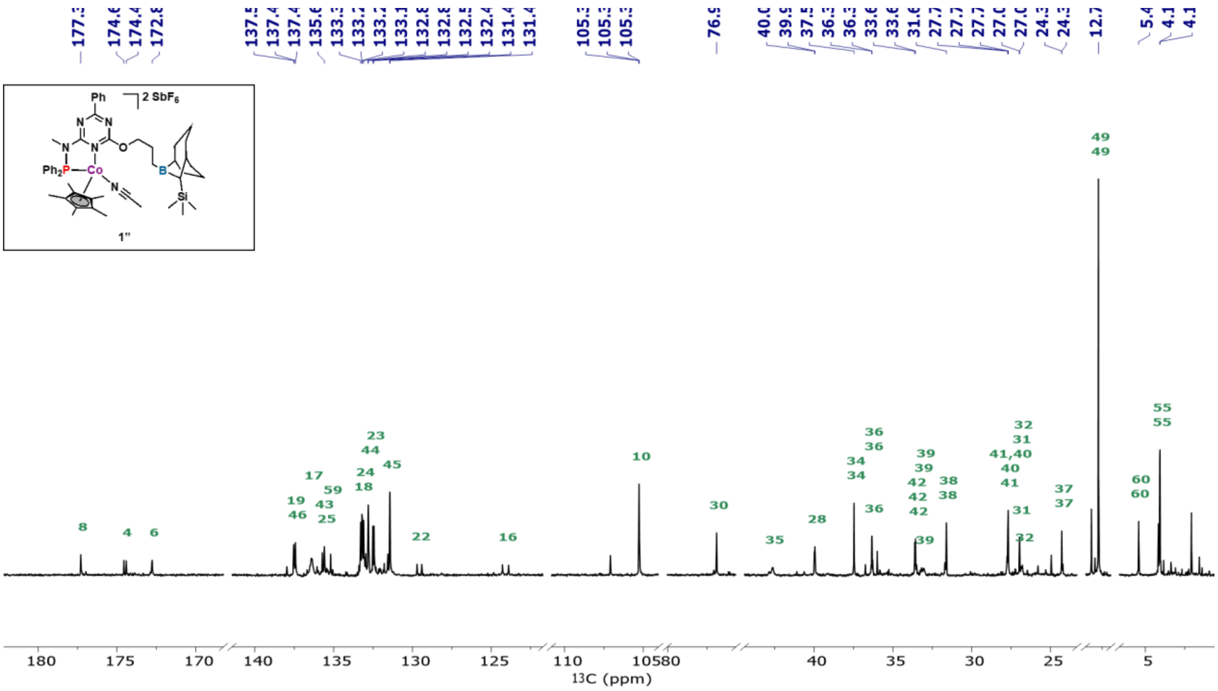

Figure S42 – **$^{13}C\{^{1}H\}$ NMR (151 MHz, CD_2Cl_2, 298 K)** spectrum of complex **1”**.

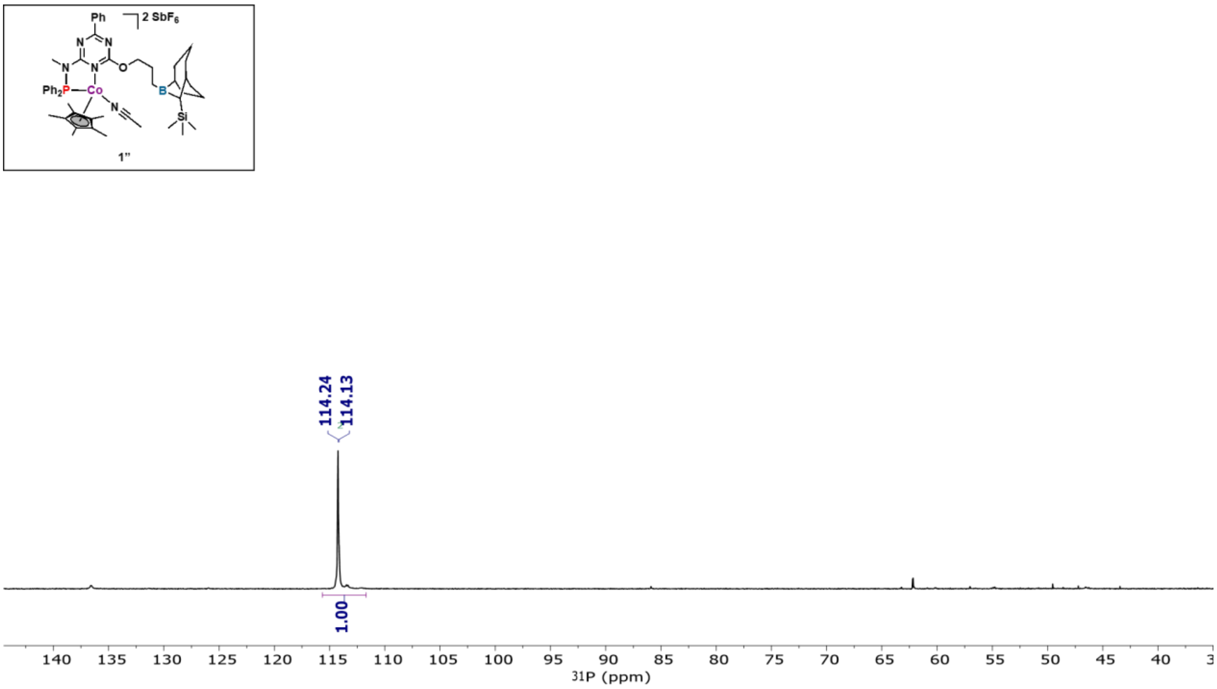

Figure S43 – ^{31}**P{^{1}H} NMR (243 MHz, CD_2Cl_2, 298 K)** spectrum of complex **1”**.

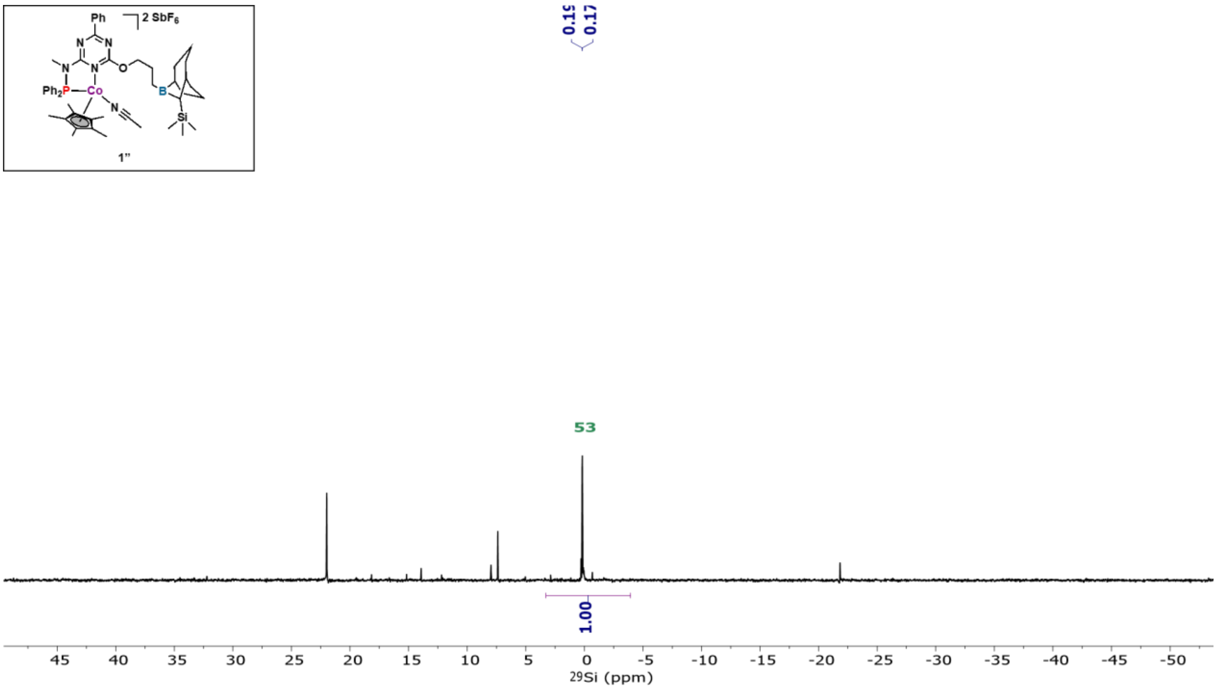

Figure S44 – ^{29}**Si NMR (119 MHz, CD_2Cl_2, 298 K)** spectrum of complex **1”**.

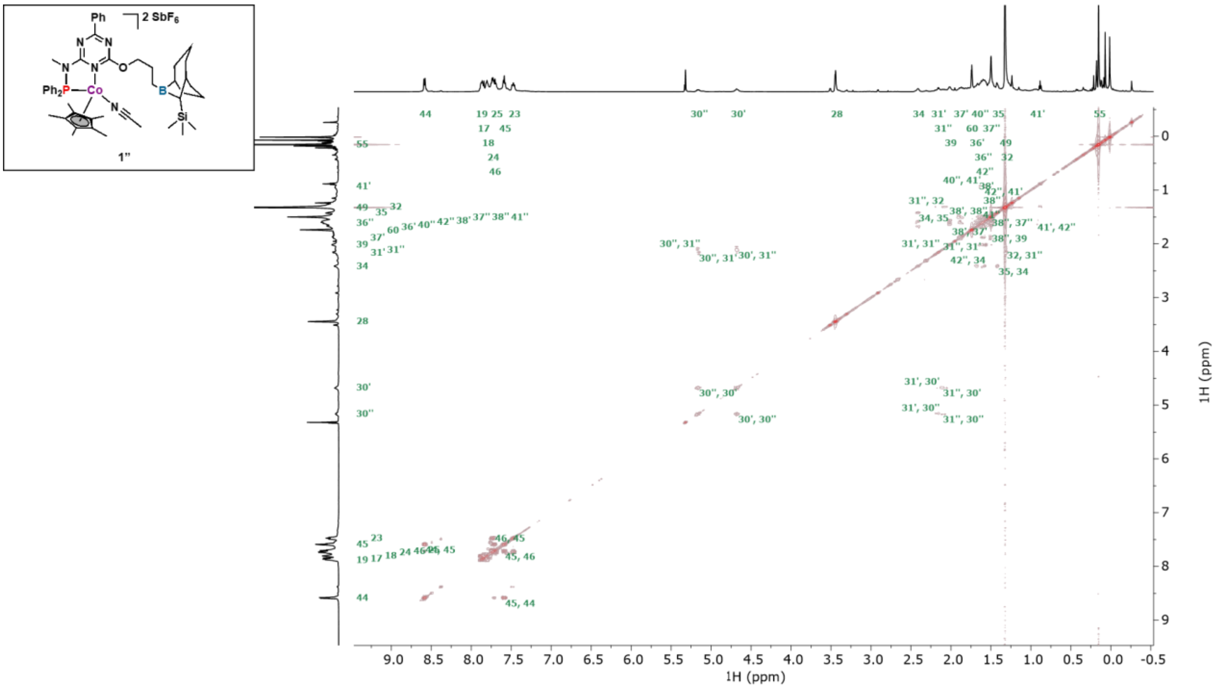

Figure S45 – **^{1}H – ^{1}H COSY NMR (600 MHz, CD_2Cl_2, 298 K)** spectrum of complex **1”**.

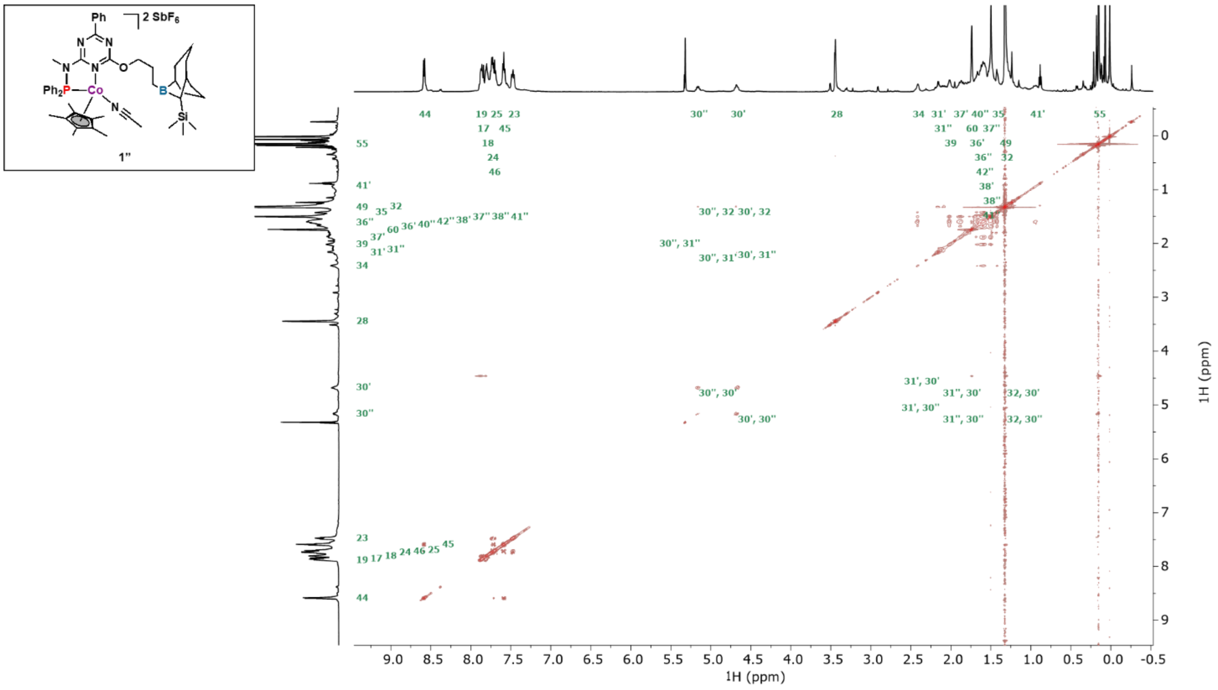

Figure S46 – **^{1}H – ^{1}H TOCSY NMR (600 MHz, CD_2Cl_2, 298 K)** spectrum of complex **1”**.

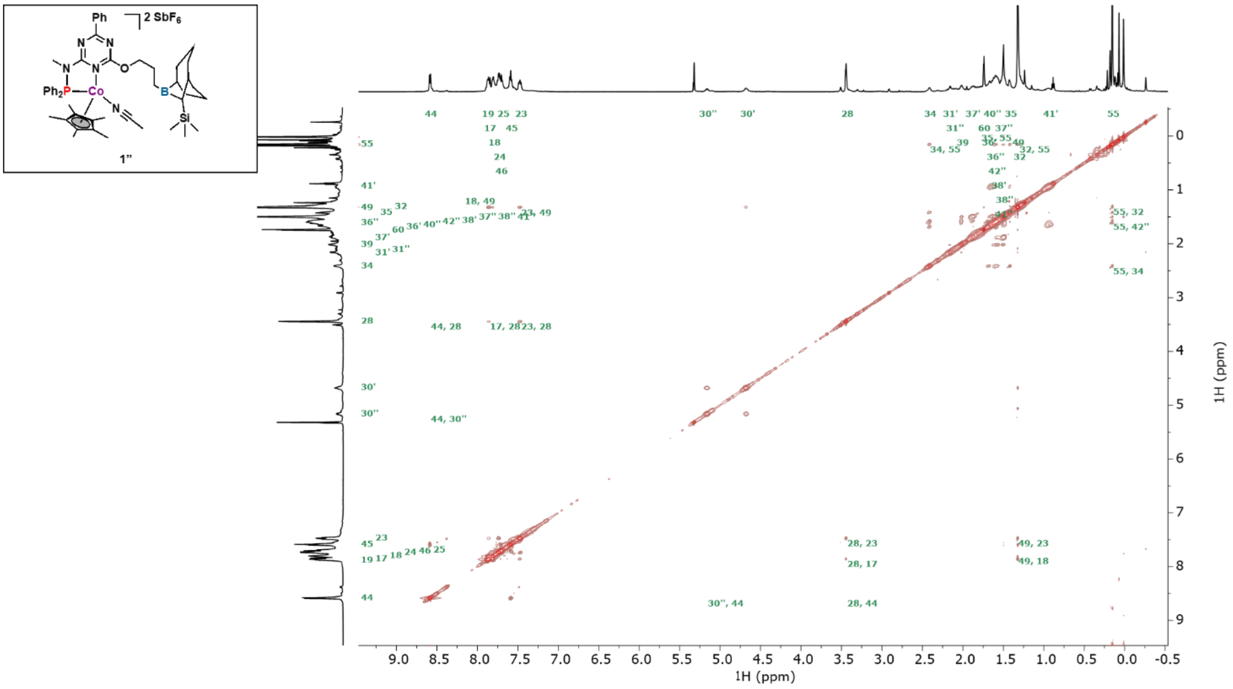

Figure S47 – **^{1}H – ^{1}H NOESY NMR (600 MHz, CD_2Cl_2, 298 K)** spectrum of complex **1”**.

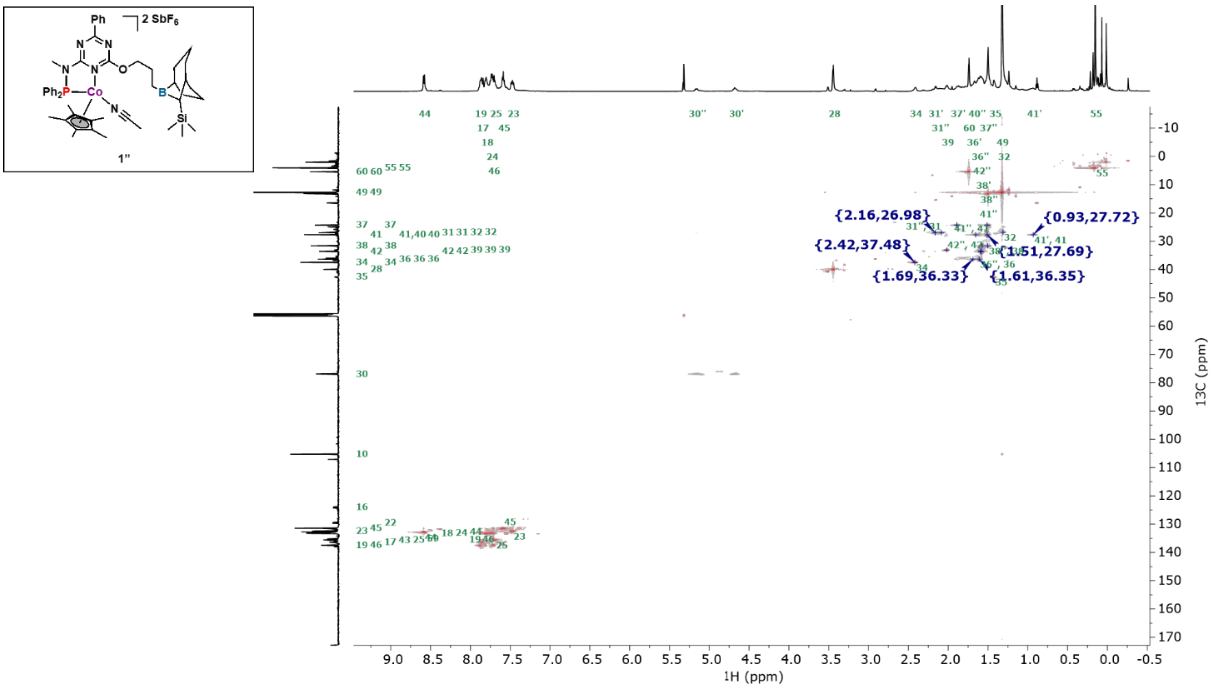

Figure S48 – **^{1}H – $^{13}C\{^{1}H\}$ HSQC NMR (600, 151 MHz, CD_2Cl_2, 298 K)** spectrum of complex **1”**.

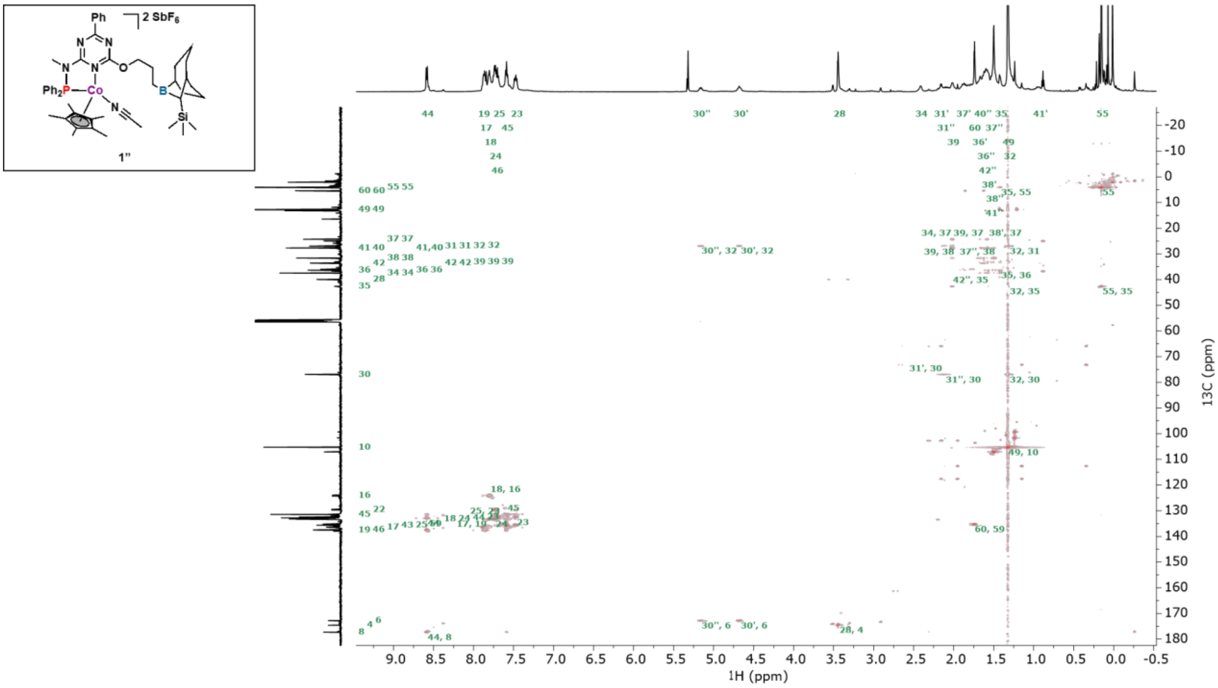

Figure S49 – **^{1}H – $^{13}C\{^{1}H\}$ HMBC NMR (600, 151 MHz, CD_2Cl_2, 298 K)** spectrum of complex **1”**.

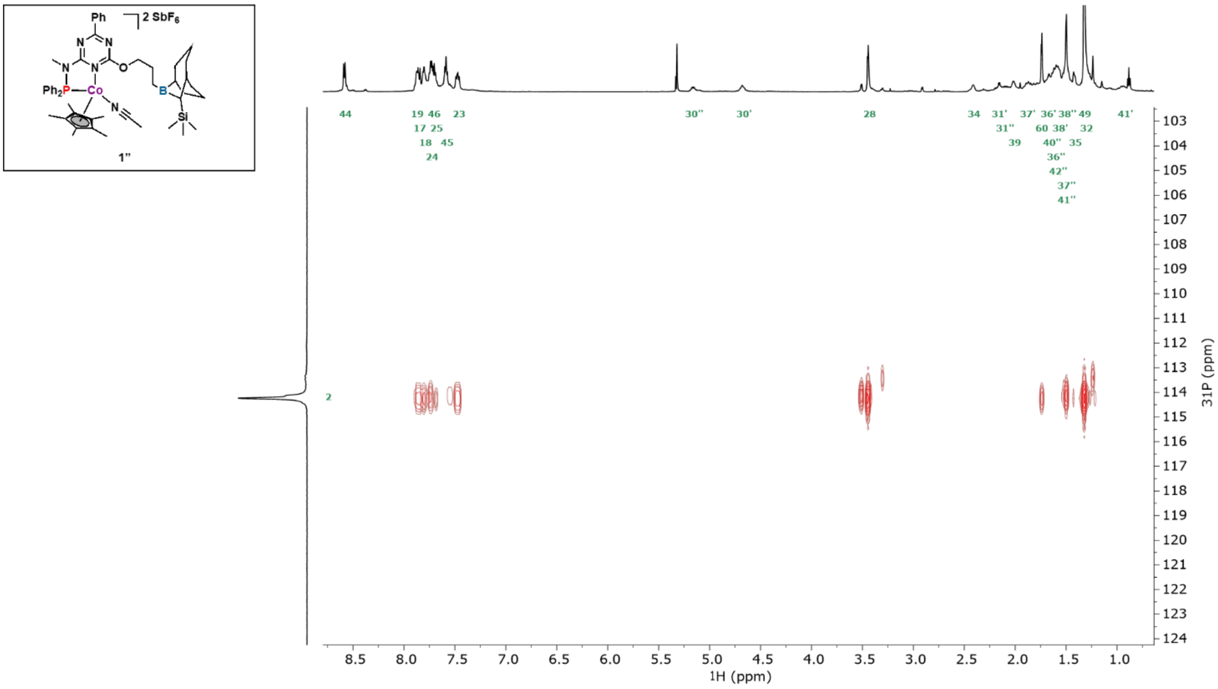

Figure S50 – **^{1}H – $^{31}P\{^{1}H\}$ HSQC NMR (600, 243 MHz, CD_2Cl_2, 298 K)** spectrum of complex **1”**.

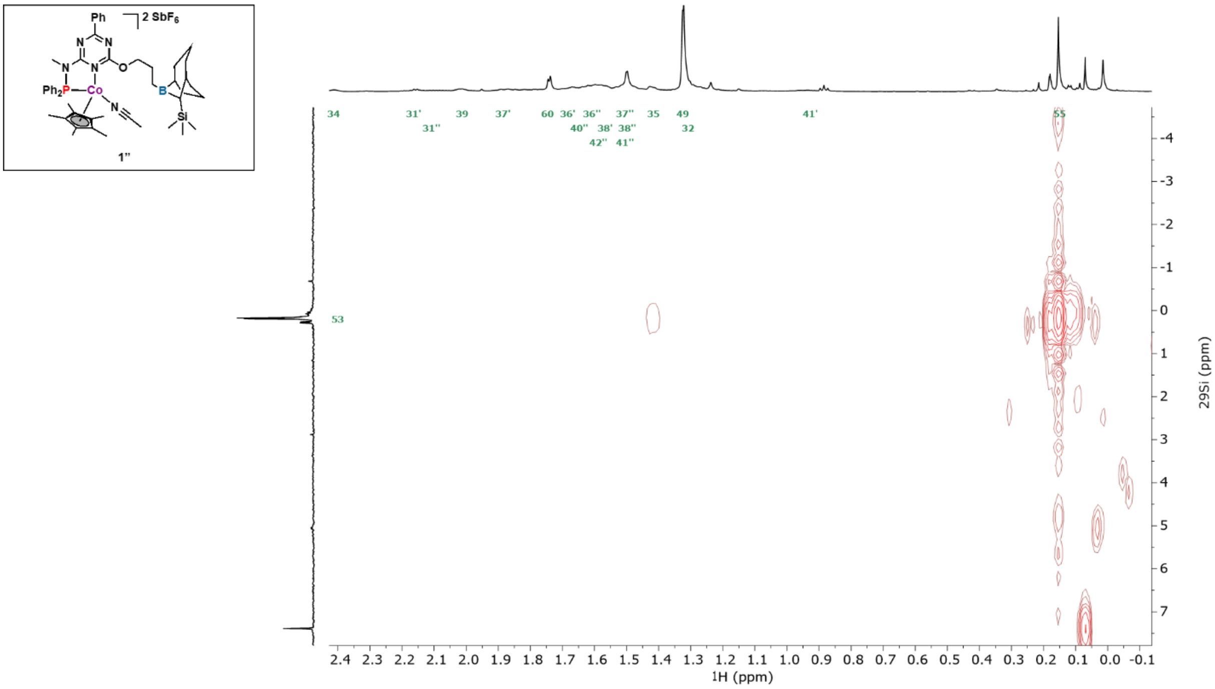

Figure S51 – **^{1}H – ^{29}Si HMBC NMR (600, 119 MHz, CD_2Cl_2, 298 K)** spectrum of complex **1”**.

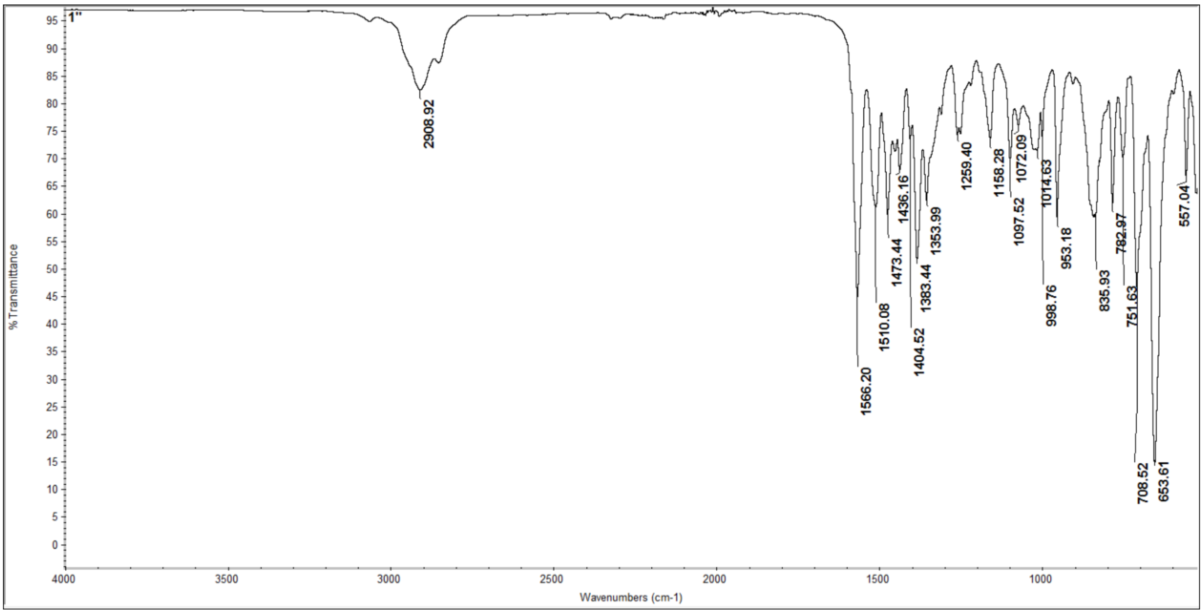

Figure S52 – **IR (Diamond – ATR, neat)** spectrum of complex **1”**.

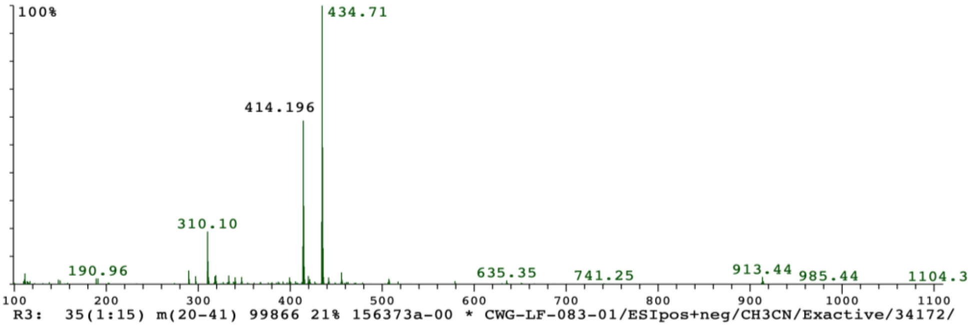

Elektrospray-ionisation neg. ions

Characteristicial ions:
235 = [Sb1F6]-

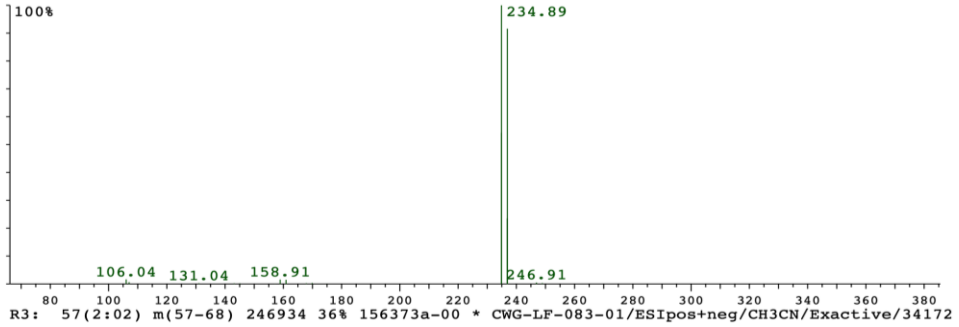

MassLib V9.4 *** Angegebene Mol.-Gewichte u. Massenzahlen basieren auf dem häufigsten Isotop der Elemente ***MPI für Kohlenforschung

Mass to be matched (m/z): 414.19617 charge: 2

Mass tolerance: ±0.005
restriction of atom numbers:

B	C	Co	H	N	O	P	Si
1-1	1-60	1-1	1-100	1-4	1-3	1-2	1-1

Number of calculated formulas: 3

Formula	Diff. (ppm)	theor. m/z
C47 H63 B Co N4 O P Si	0.07	414.19614
C49 H65 B Co N O2 P Si	-1.55	414.19681
C45 H68 B Co N2 O2 P2 Si	-1.92	414.19697

Figure S53 – **ESI-HRMS** spectra post-reaction of **1** with TMSCHN_2, indicating the potential formation of **1”**.

5.7.1. Reaction of 1 with TMSCHN$_2$

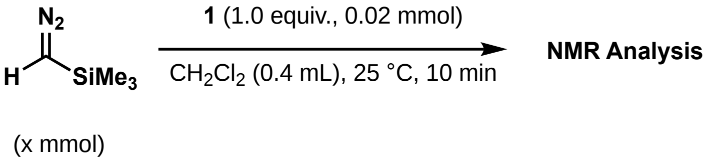

Procedure: Complex **1** (23.6 mg, 0.02 mmol) was introduced in a CH_2Cl_2 (0.4 mL) solution within an oven-dried 7 mL reaction vial inside a glove box under argon. Me_3SiCHN_2 was then added dropwise to the stirring reaction mixture. Upon the addition of Me_3SiCHN_2, N_2 evolution was observed. Stirring of the resultant solution continued for 1 h at 25 °C. Post-reaction, an aliquot of the crude reaction mixture was analyzed by ^{1}H NMR.

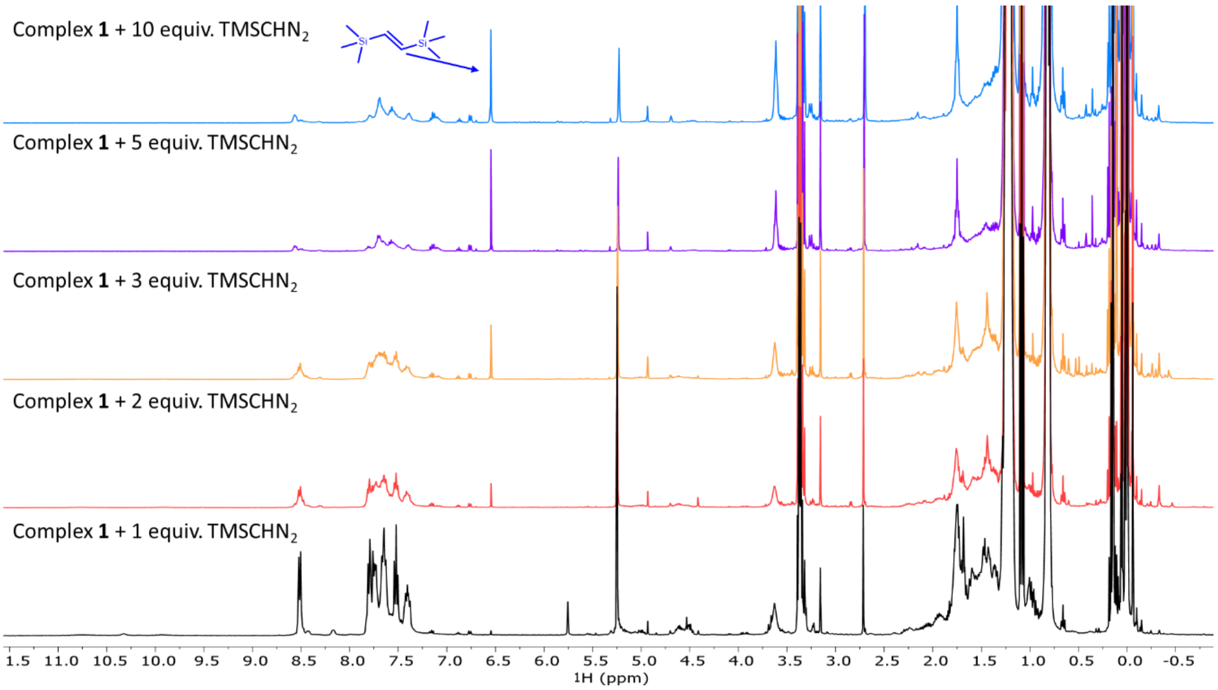

Figure S54 – **^{1}H NMR (400 MHz, CD_2Cl_2, 293 K)** spectrum of crude reaction mixture.

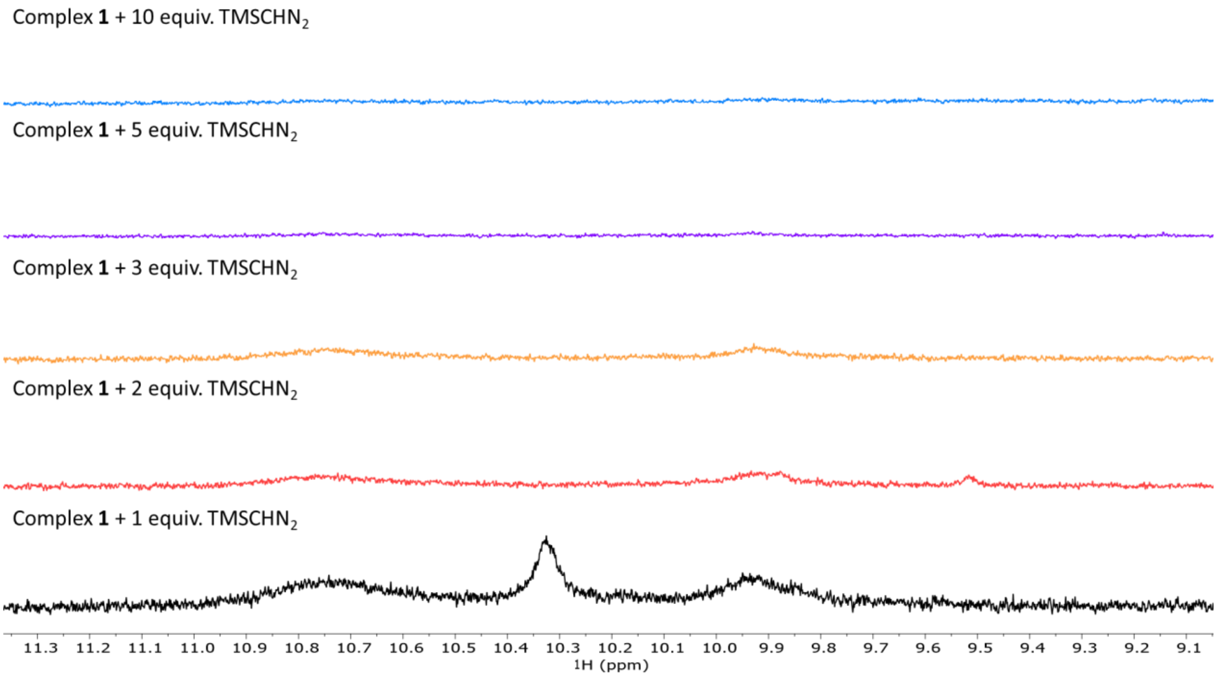

Figure S55 – **^{1}H NMR (400 MHz, CD_2Cl_2, 293 K)** spectrum of crude reaction mixture.

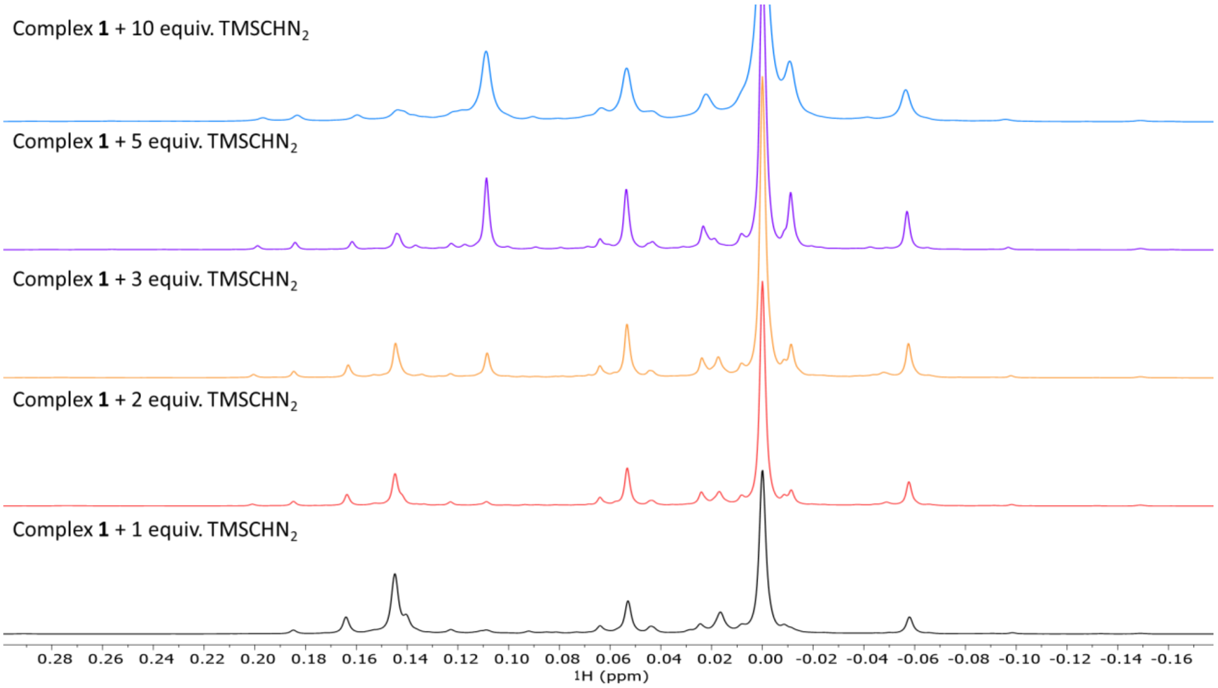

Figure S56 – **^{1}H NMR (400 MHz, CD_2Cl_2, 293 K)** spectrum of crude reaction mixture.

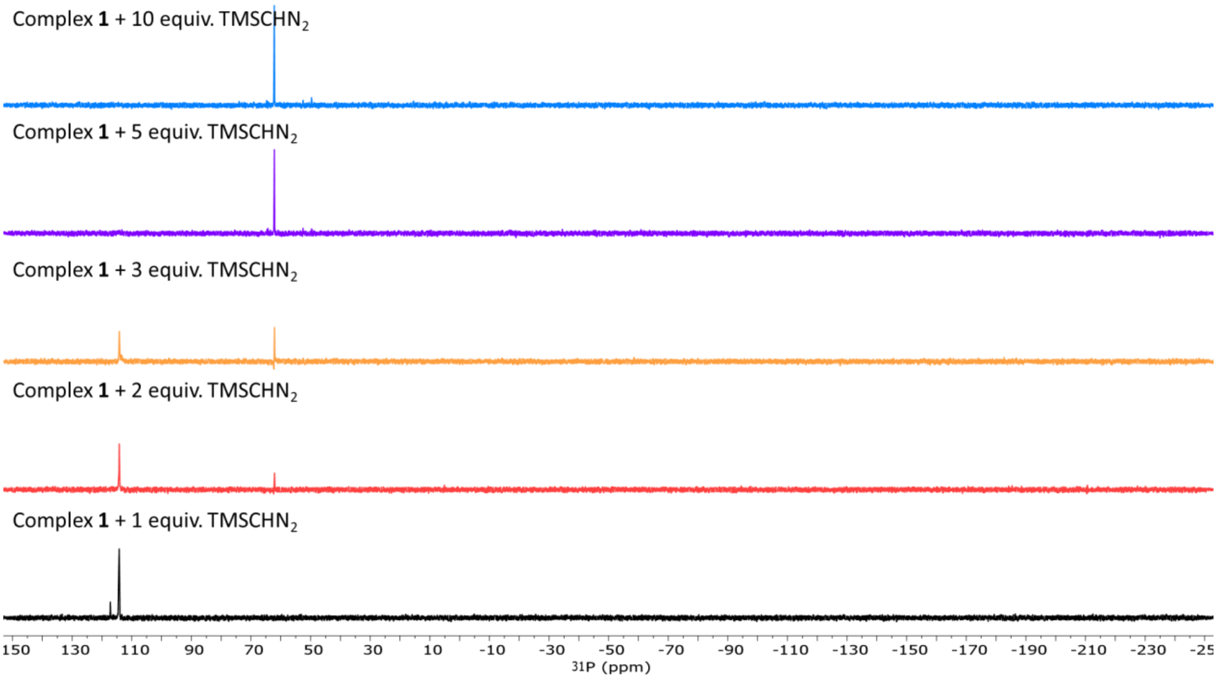

Figure S57 – **$^{31}P\{^{1}H\}$ NMR (400 MHz, CD_2Cl_2, 293 K)** spectrum of crude reaction mixture.

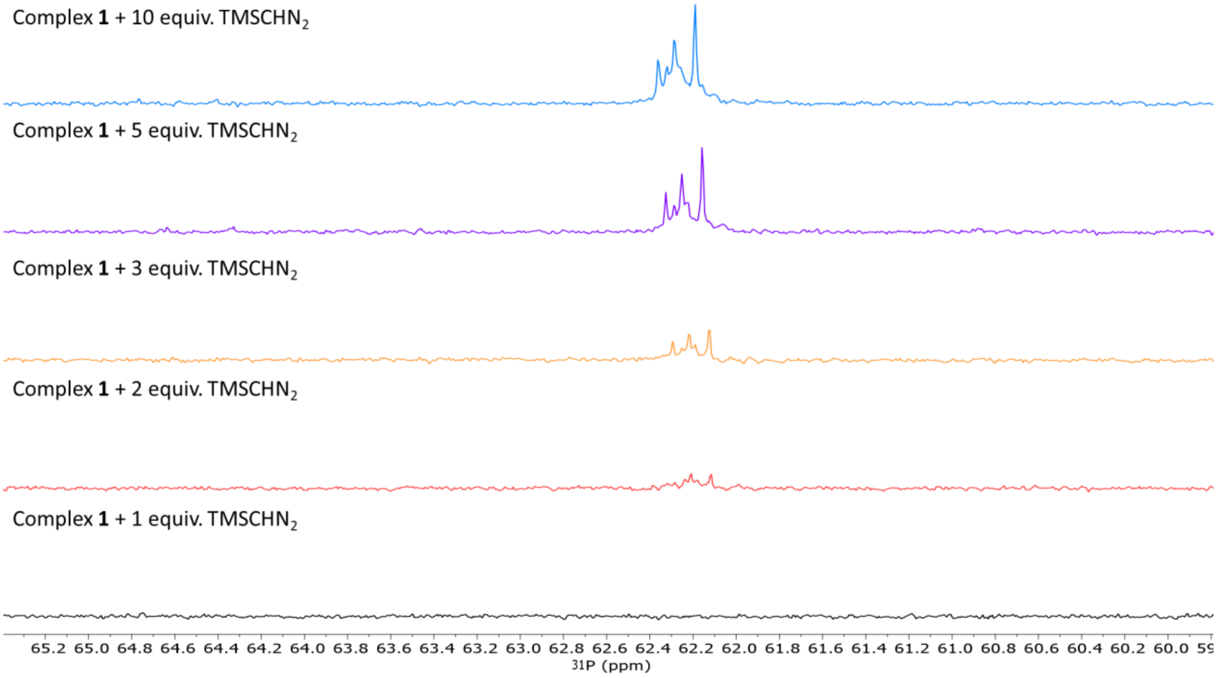

Figure S58 – **$^{31}P\{^{1}H\}$ NMR (400 MHz, CD_2Cl_2, 293 K)** spectrum of crude reaction mixture.

5.7.2. Analyzing Product Formation in the Reaction Between 1 (5 mol%) and Me_3SiCHN_2

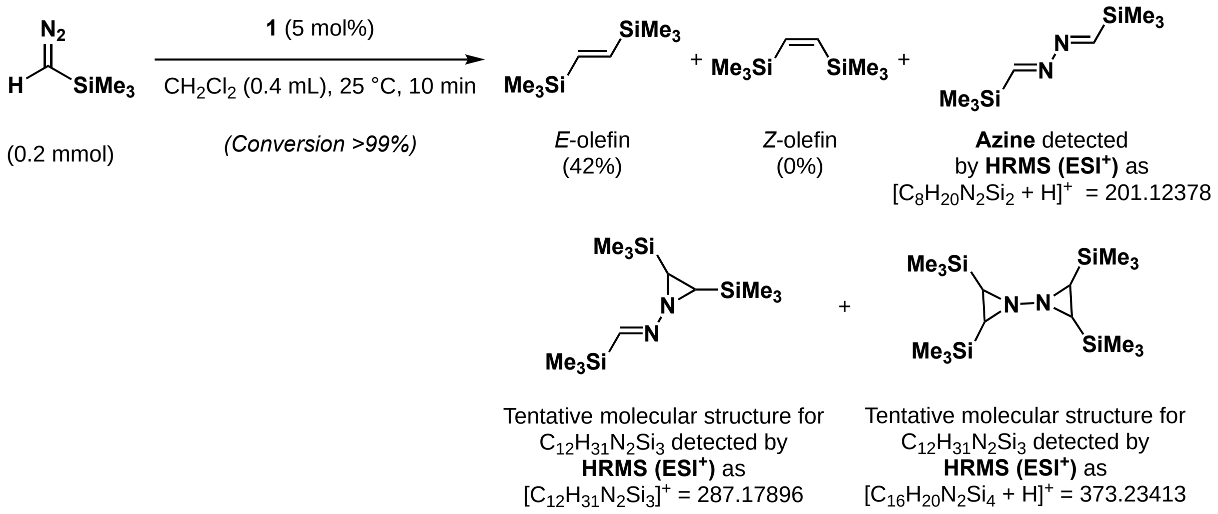

Procedure: In an oven-dried 8 mL reaction vial under an argon atmosphere, complex **1** (12.56 mg, 5 mol%), and dichloromethane (0.4 mL) were added. Me_3SiCHN_2 (100 µL, 0.2 mmol) was then introduced dropwise using a micropipette while stirring. The resulting mixture was stirred for 1 hour at 25 ºC. Subsequently, the solvent was removed *in vacuo*. The crude reaction mixture was extracted using pentane (3 × 5 mL), and the solution was concentrated. Mesitylene (0.2 mmol) was added as a standard to assess the conversion and yield of the reaction and also was analyzed by mass spectrometry.

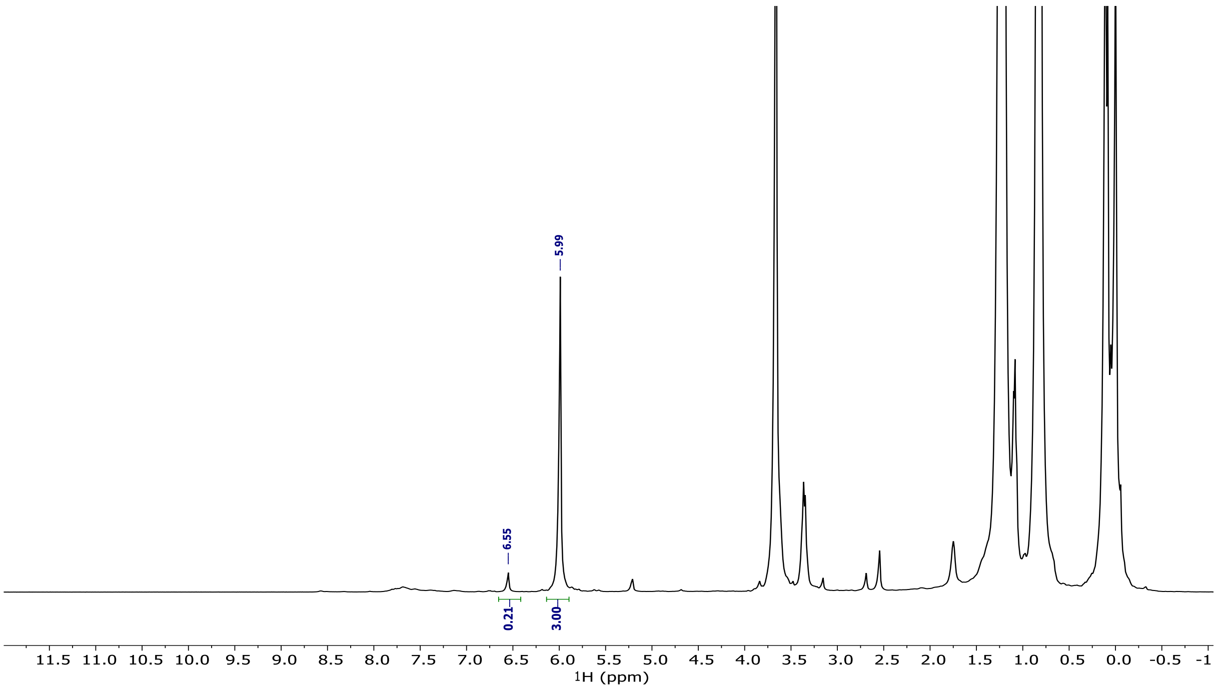

Figure S59 – **^{1}H NMR (400 MHz, CD_2Cl_2, 293 K)** spectrum of crude reaction mixture.

second interpretation

electrospray-ionization (Sol.: CH2Cl2+CH3CN) pos. ions

molecular weight 200, 286 et al.

characteristical ions
201 = [200 + H]+
287 = [286 + H]+

additional characteristical ions
74, 88, 102, 114, 116, 130, 144, 158, 174, 188, 202, 216, 373, 409

additional characteristical ions (doubly charged)
318, 387

Datum	28.07.2023
File:	E44702a-00.RAW
Analyse:	CWG-VC-815-01
WLG: Werle, Christophe	
Ionisierung:	ESIpos+neg
Lösungsmittel:	CH2Cl2+CH3CN
Spektrometer:	Exactive
Auswerter:	Kampen (2242)

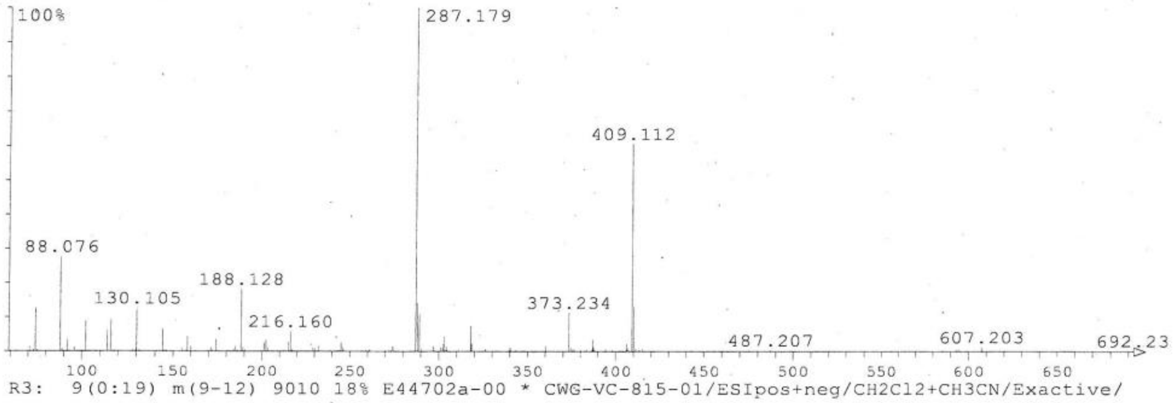

Mass to be matched (m/z): 373.234100 Charge: 1

Mass Tolerance: ±0.050000
Restriction of atom numbers:

C	H	N	Si
1-150	1-150	2-2	4-4

Number of calculated Formulas: 1

Formula	Diff.(ppm)	theor. m/z
C16 H41 N2 Si4	0.10	373.234137

Suggestion:
C16H20N2Si4 MW: 372

Characteristic ions:
373 = [372 + H]+

Figure S60 – **ESI-HRMS** spectra of the reaction mixture following the reaction of **1** (5 mol%) with $TMSCHN_2$.

Mass to be matched (m/z): 201.12379 charge: 1

Mass tolerance: ±0.005
restriction of atom numbers:

C	H	N	O	Si
1-100	1-100	0-2	0-10	0-2

Number of calculated formulas: 3

Formula	Diff. (ppm)	theor. m/z
C8 H21 N2 Si2	-0.05	201.12378
C9 H17 N2 O3	-2.09	201.12337
C14 H17 O1	17.90	201.12739

Datum	28.07.2023
Analyse:	E44702a-00.RAW
Sigel:	CWG-VC-815-01
WLG: Werle,Christophe	
Messung:	HRMS
Methode:	ESIpos+neg
Lösungsmittel:	CH2Cl2+CH3CN
Spektrometer:	Exactive
Auswerter:	Kampen (2242)

possible elemental composition of m/z 201

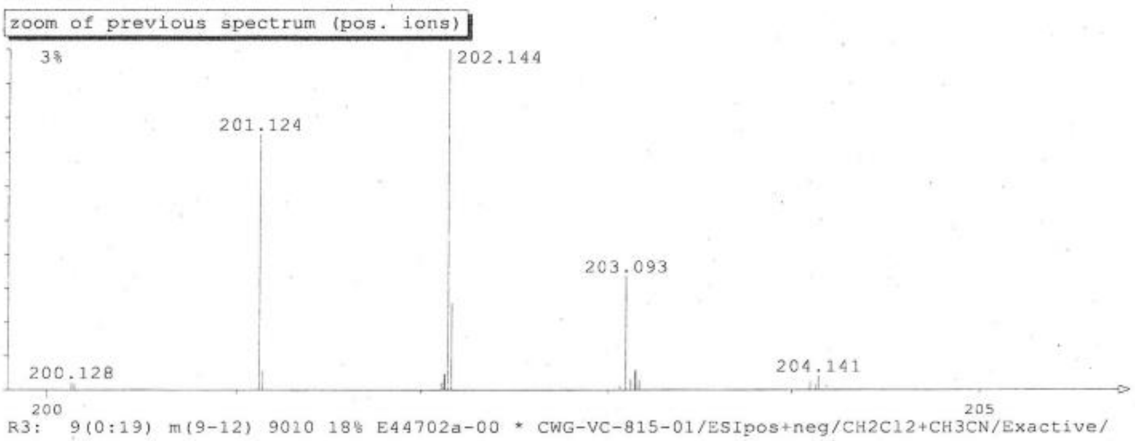

Mass to be matched (m/z): 287.17895 charge: 1

Mass tolerance: ±0.002
restriction of atom numbers:

C	H	N	Si
1-30	1-60	1-5	1-3

Number of calculated formulas: 1

Formula	Diff. (ppm)	theor. m/z
C12 H31 N2 Si3	0.03	287.17896

Datum:	28.07.2023
Analyse:	E44702a-00
Sigel:	CWG-VC-815-01
	WLG: Werle,Christophe
Method:	HR-MS
Ionis. :	ESIpos
solvent :	CH2Cl2 + CH3CN
Spectrometer:	Exactive
Auswerter:	Marcus, Tel:2234

Figure S61 – **ESI-HRMS** spectra of the reaction mixture following the reaction of **1** (5 mol%) with $TMSCHN_2$.

5.7.3. Probing the Impact of Reagent Addition Sequence: Commencing with Me_3SiCHN_2 and Subsequently Introducing Aldehyde.

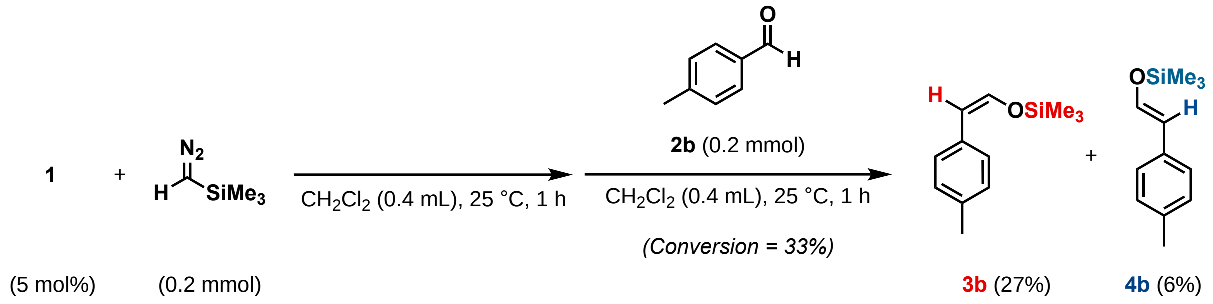

Procedure: Within an 8 mL oven-dried reaction vial under an argon atmosphere, complex **1** (12.6 mg, 5 mol%) and dichloromethane-d_2 (0.4 mL) were introduced. Subsequently, Me_3SiCHN_2 (100 µL, 0.2 mmol) was added dropwise using a micropipette under continuous stirring. This mixture was then stirred for 1 hour at 25 ºC. After this period, 4-methylbenzaldehyde (23.6 µL, 0.2 mmol) was added to the reaction mixture, followed by an additional 1-hour stirring. The mixture was subsequently analyzed by NMR spectrometry. The NMR data revealed a 32% conversion of the aldehyde: 27% as the *Z* product and 6% as the *E* product. This contrasts with a >99% conversion, comprising 83% *Z* and 17% *E* products, observed under standard reaction conditions.

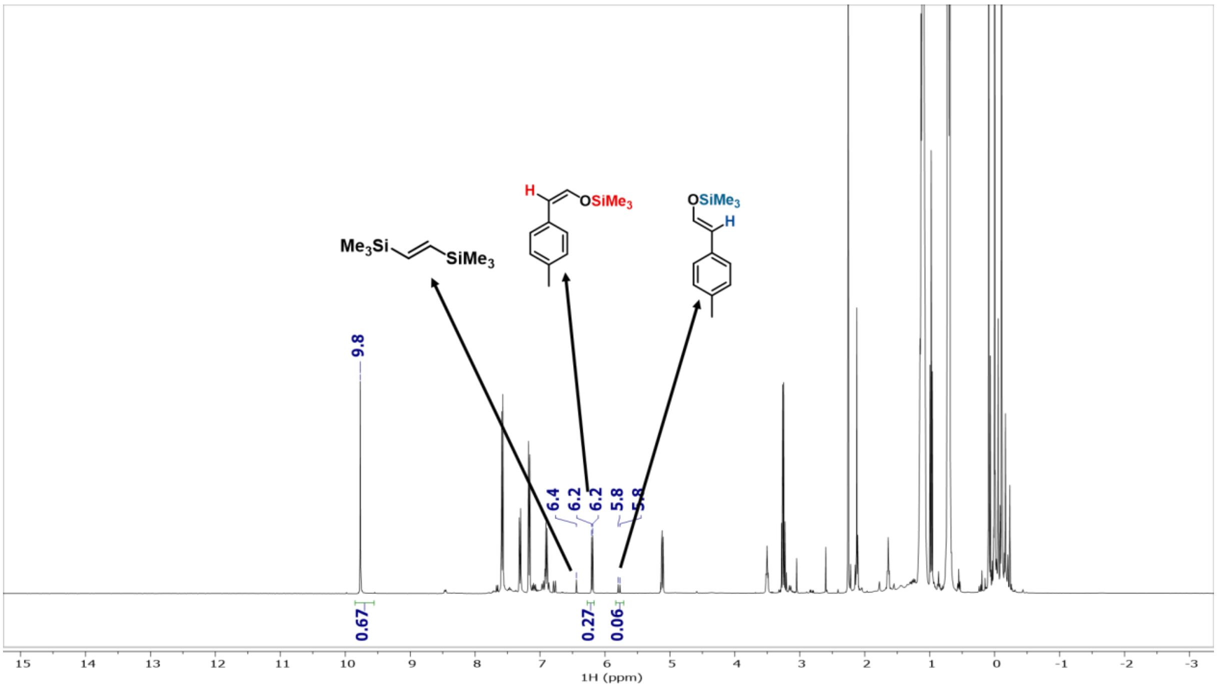

Figure S62 – **^{1}H NMR (400 MHz, CD_2Cl_2, 293 K)** spectrum of the crude reaction mixture.

5.8. Control Experiments

5.8.1. Reaction of Aldehyde with TMSCHN₂

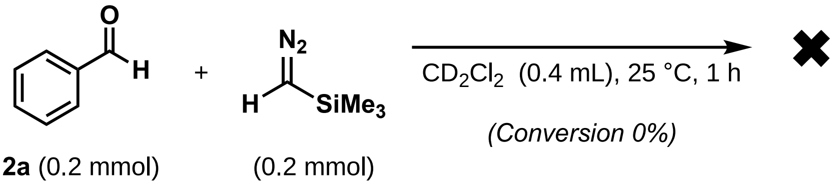

Procedure: A reaction vial (7 mL) was oven-dried and backfilled with argon. Benzaldehyde (20.4 µL, 0.2 mmol) was then introduced to the vial, followed by the addition of Me_3SiCHN_2 (100 µL, 0.2 mmol) in a deuterated dichloromethane solvent (CD_2Cl_2, 0.4 mL). The resulting solution was stirred at ambient temperature (25 ºC) for 60 minutes. Subsequently, the reaction mixture was transferred to an NMR tube for analysis. ^1H NMR spectroscopy was employed to evaluate the yield and conversion of the crude product. The analytical data indicated no detectable chemical reaction between the benzaldehyde and Me_3SiCHN_2 under the conditions applied.

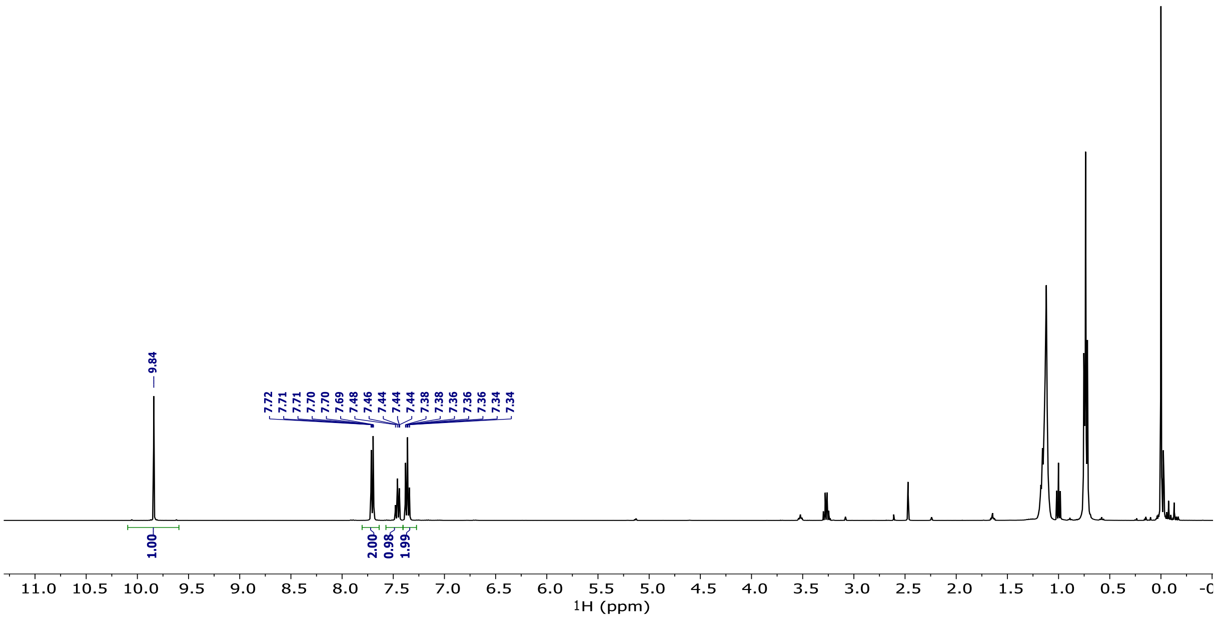

Figure S63 – **^{1}H NMR (400 MHz, CD_2Cl_2, 293 K)** spectrum of the crude reaction mixture.

5.8.2. Reaction of Aldehyde with TMSCHN2 in presence of 9-HBBN

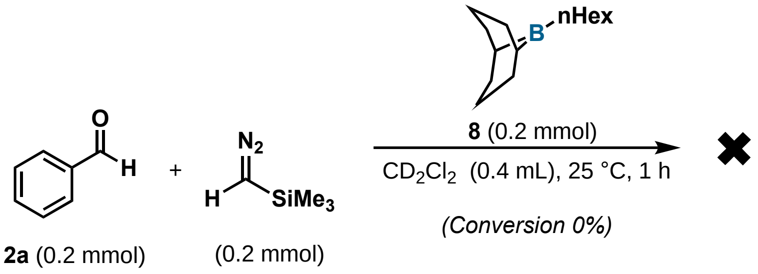

Procedure: In a 7 mL vial previously dried and purged with argon, a mixture of benzaldehyde (20.4 µL, 0.2 mmol), Me_3SiCHN_2 (100 µL, 0.2 mmol), and 9-HBBN (41.3 mg, 0.2 mmol) was prepared in deuterated dichloromethane (CD_2Cl_2, 0.4 mL). This mixture was stirred at room temperature (25 ºC) for one hour. Afterward, it was directly transferred into an NMR tube for analysis. The yield and conversion of the reaction mixture were assessed by ^1H NMR spectroscopy, which indicated no discernible reaction between the benzaldehyde and Me_3SiCHN_2.

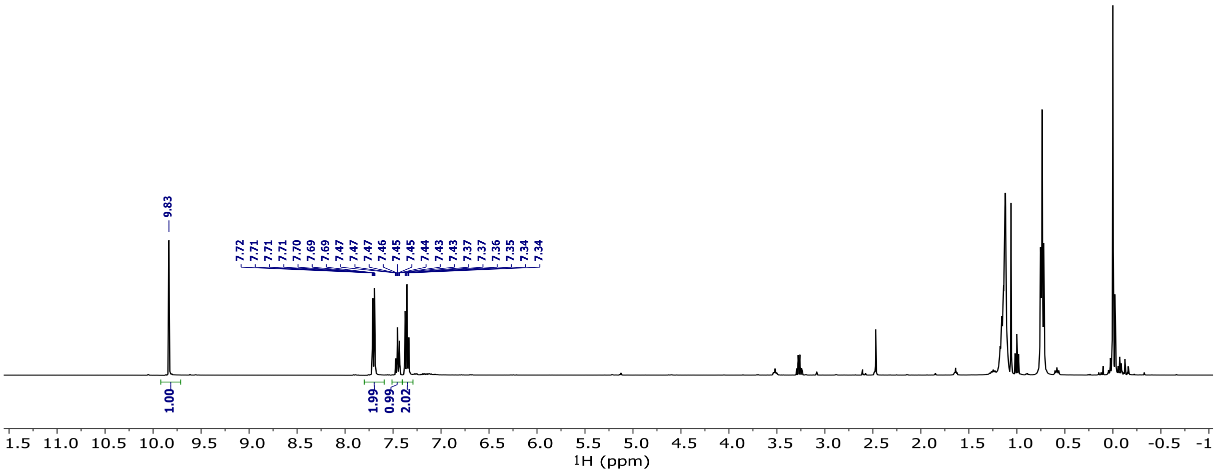

Figure S64 – **^1H NMR (400 MHz, CD_2Cl_2, 293 K)** spectrum of the crude reaction mixture.

6. ^{1}H and ^{13}C NMR Spectral Analysis of (*Z*)-Silyl Enol Ethers

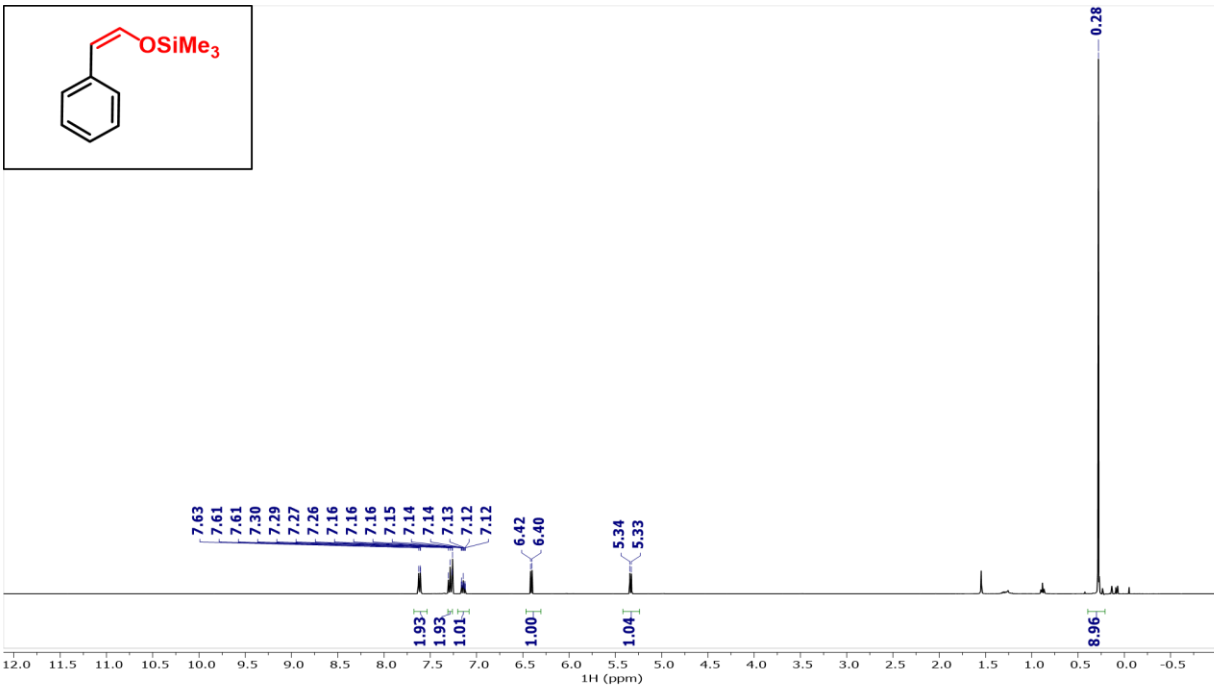

Figure S65 – **^{1}H NMR (400 MHz, $CDCl_3$, 296 K)** spectrum of compound **3a**.

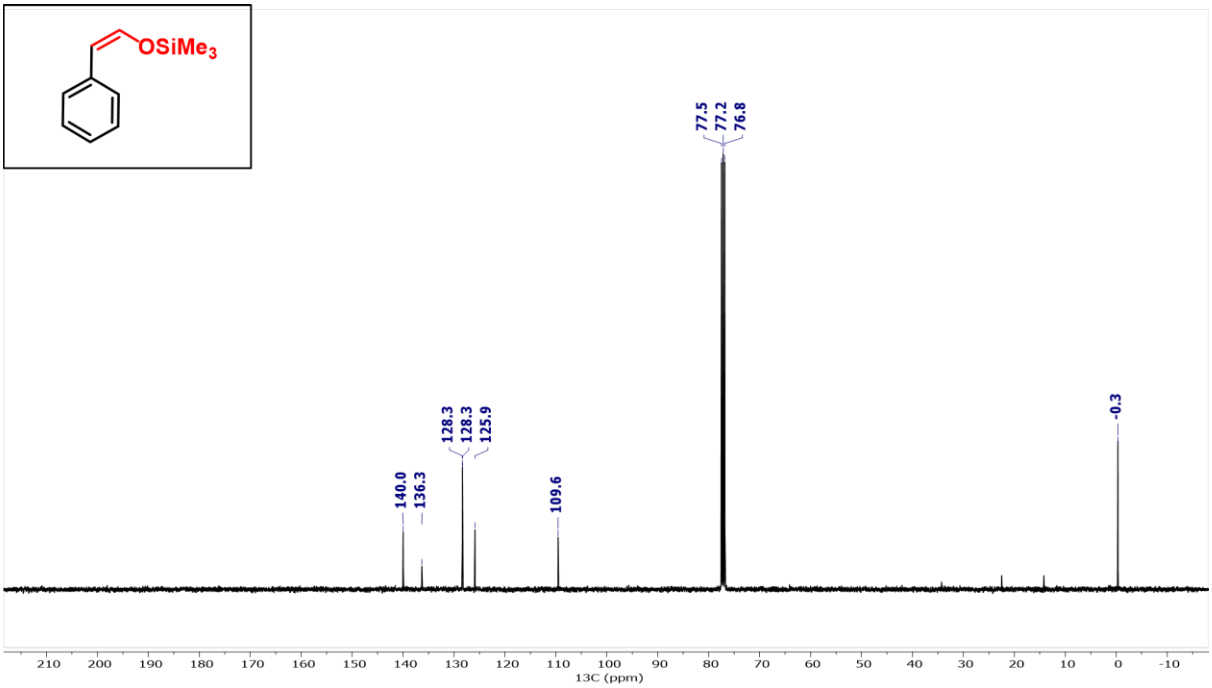

Figure S66 – **$^{13}C\{^{1}H\}$ NMR (101 MHz, $CDCl_3$, 296 K)** spectrum of compound **3a**.

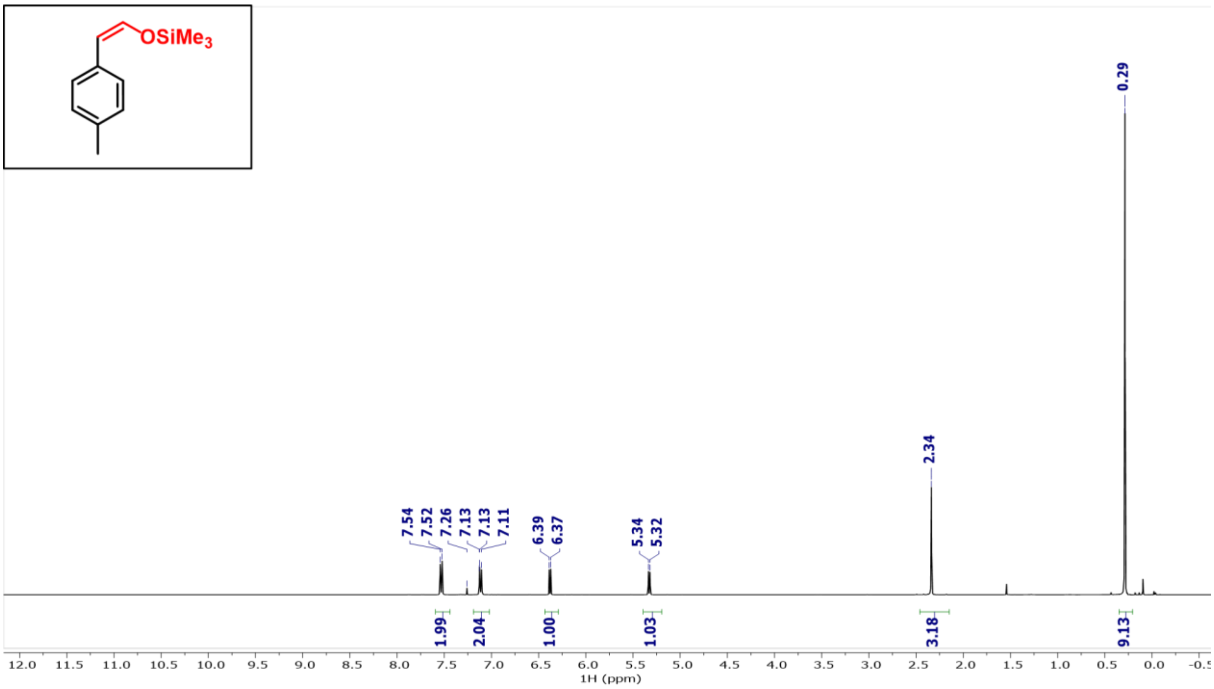

Figure S67 – **^{1}H NMR (400 MHz, $CDCl_3$, 296 K)** spectrum of compound **3b**.

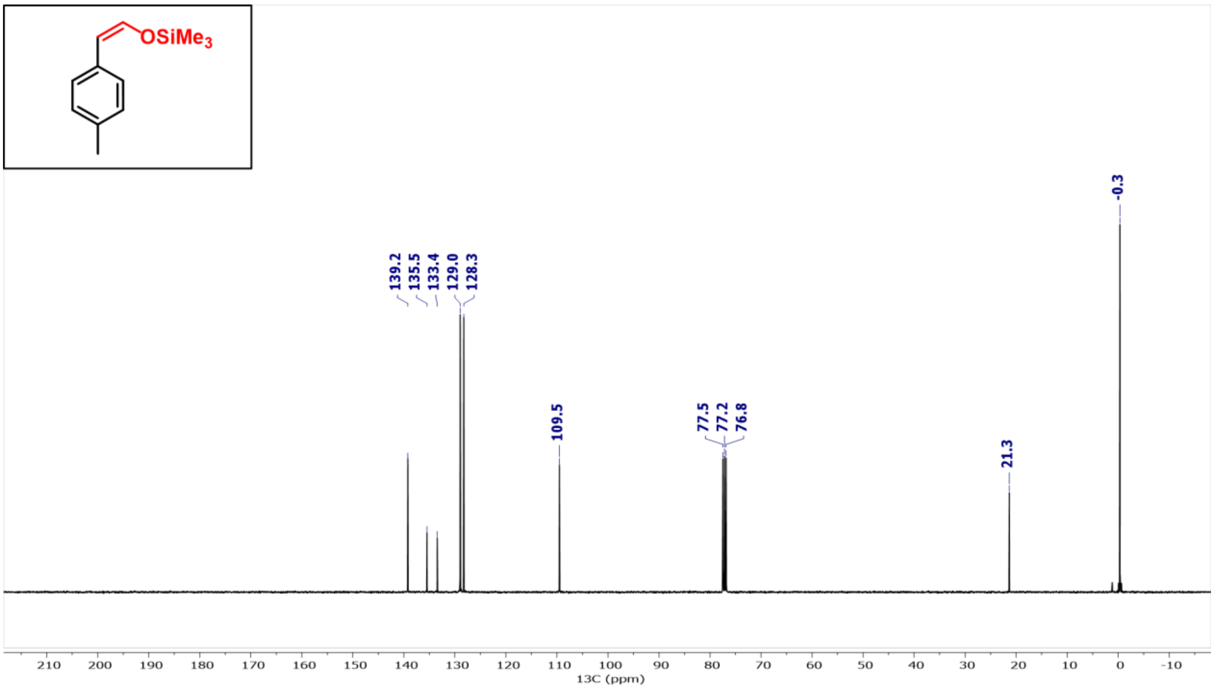

Figure S68 – **^{13}C{^{1}H} NMR (101 MHz, $CDCl_3$, 296 K)** spectrum of compound **3b**.

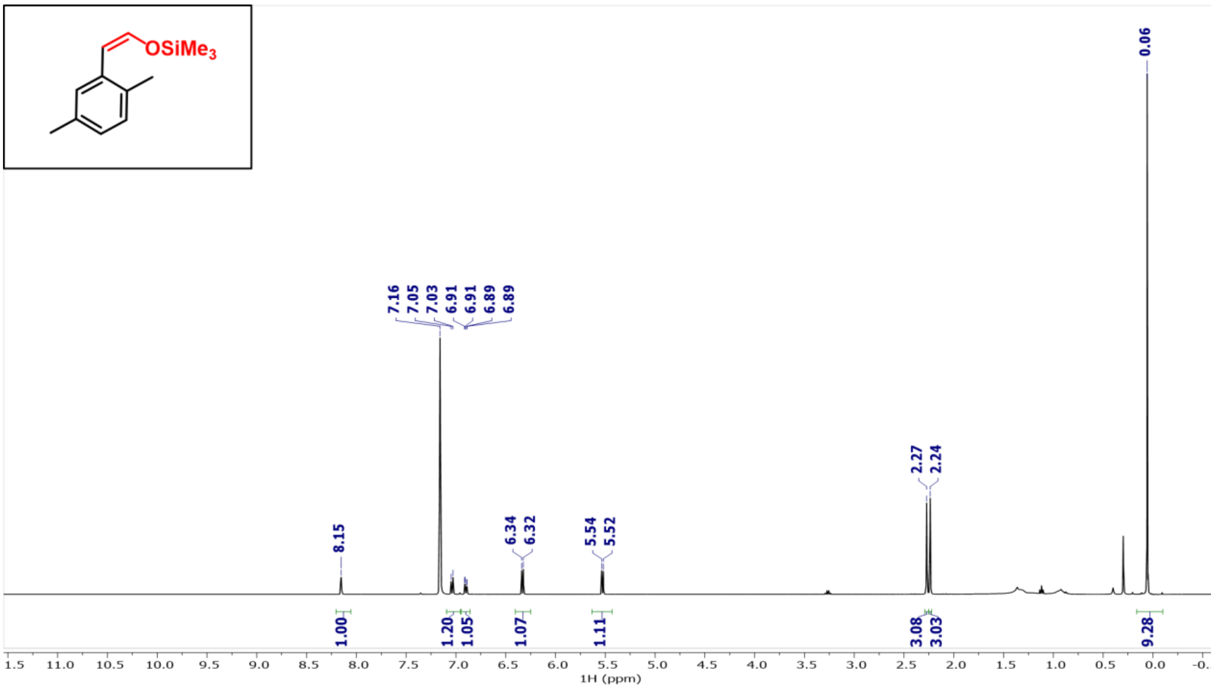

Figure S69 – **^{1}H NMR (400 MHz, C_6D_6, 296 K)** spectrum of compound **3c**.

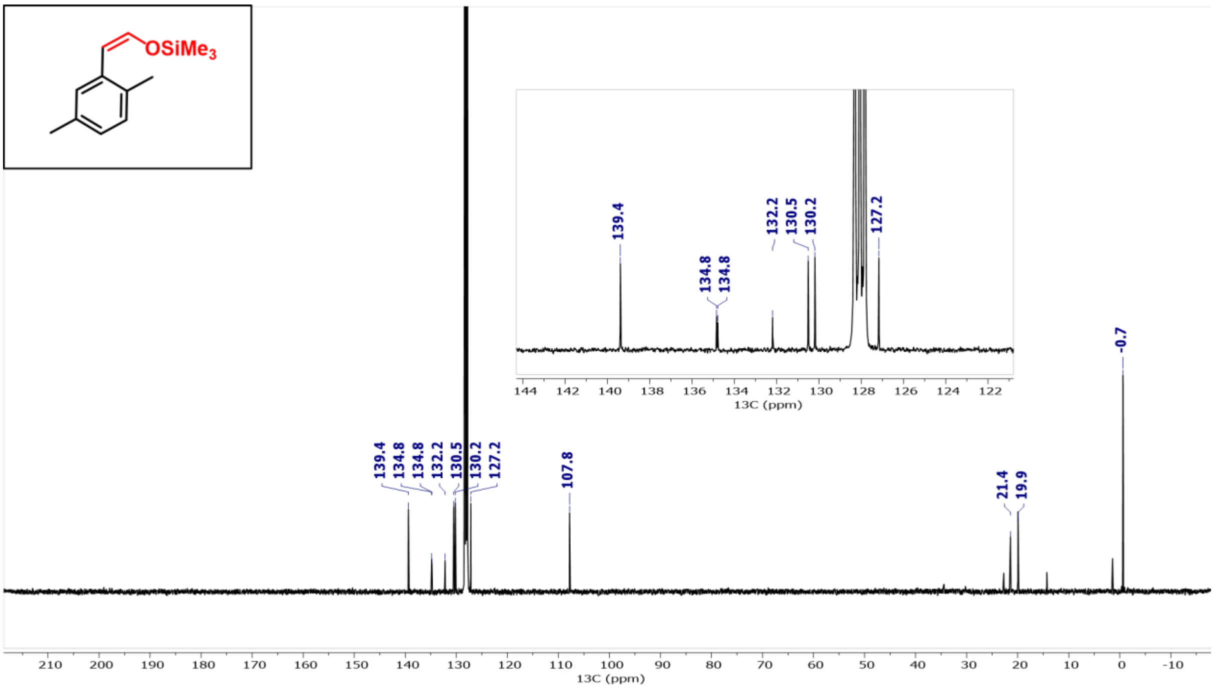

Figure S70 – **^{13}C{^{1}H} NMR (101 MHz, C_6D_6, 296 K)** spectrum of compound **3c**.

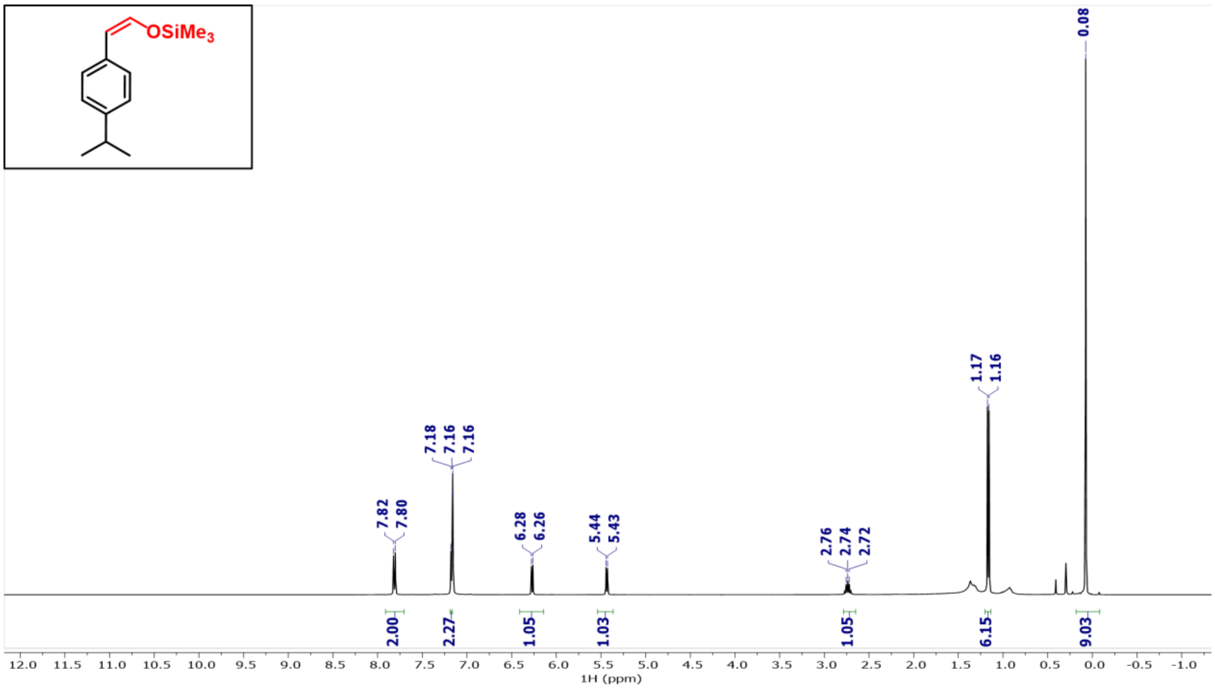

Figure S71 – **^{1}H NMR (400 MHz, C_6D_6, 296 K)** spectrum of compound 3d.

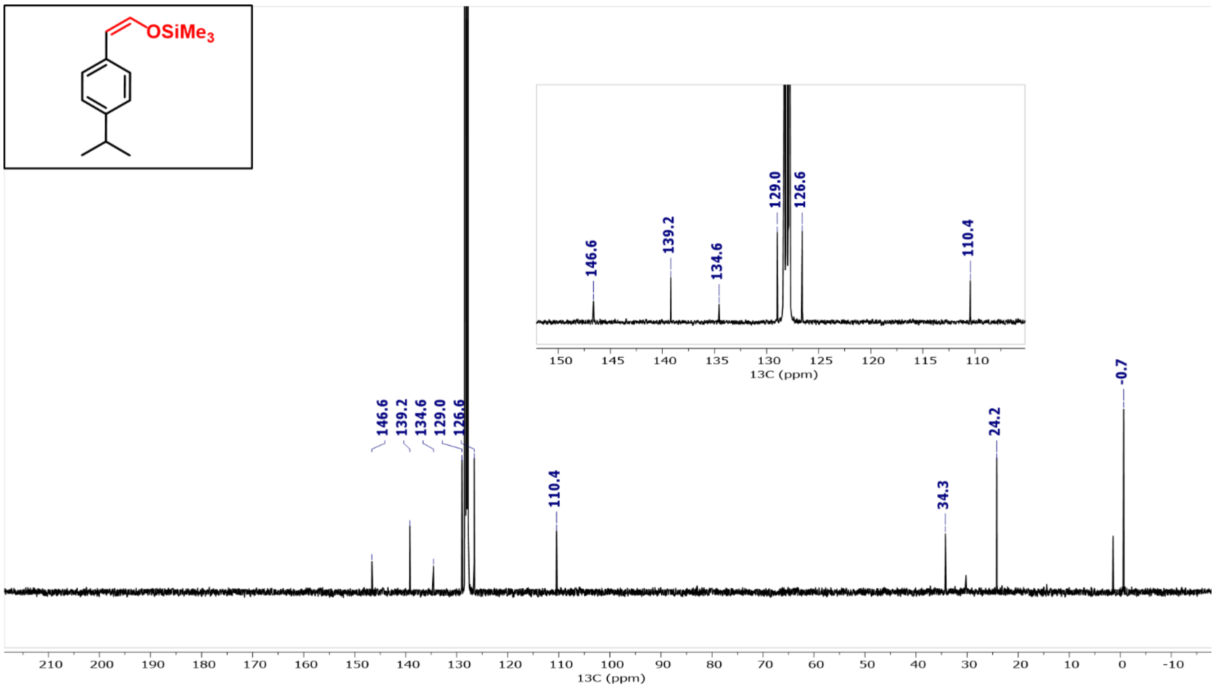

Figure S72 – **^{13}C{^{1}H} NMR (101 MHz, C_6D_6, 296 K)** spectrum of compound 3d.

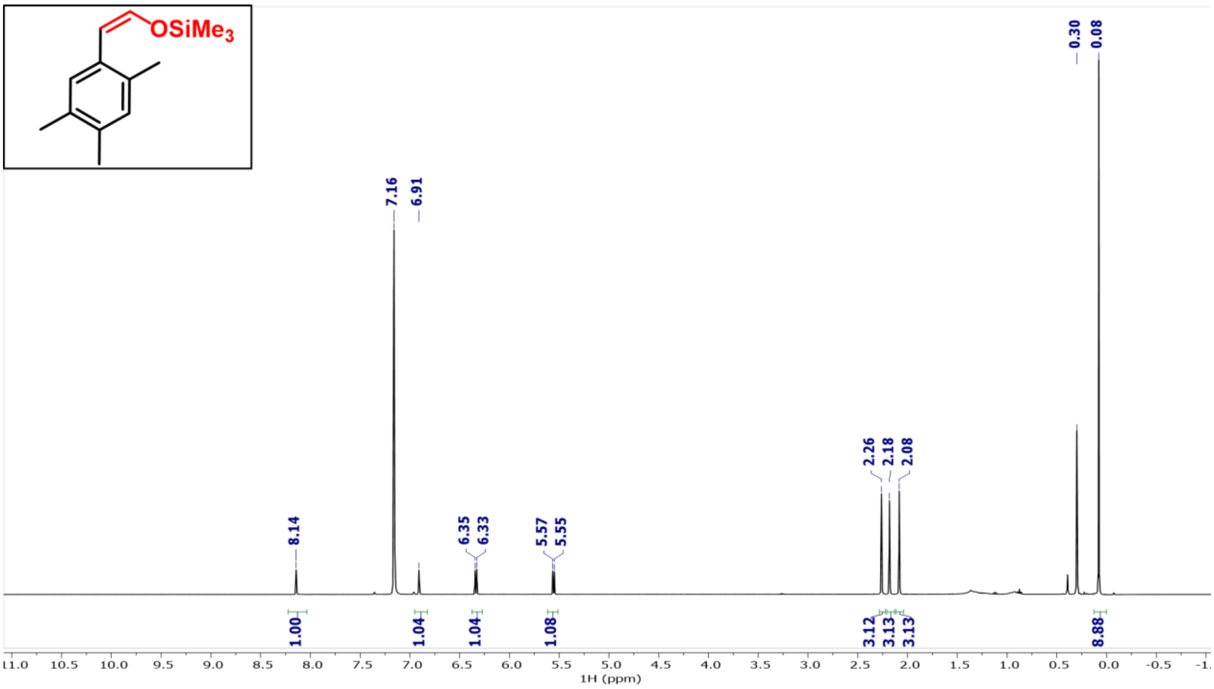

Figure S73 – [1]**H NMR (400 MHz, C_6D_6, 296 K)** spectrum of compound **3e**.

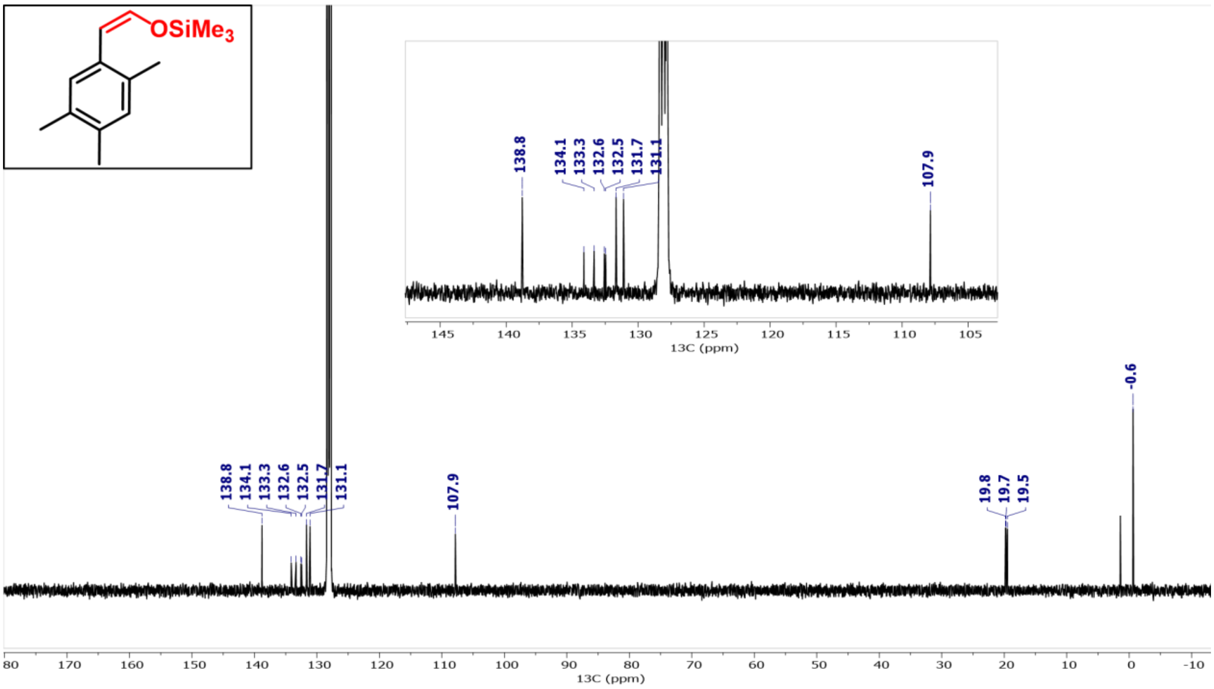

Figure S74 – **$^{13}C\{^1H\}$ NMR (101 MHz, C_6D_6, 296 K)** spectrum of compound **3e**.

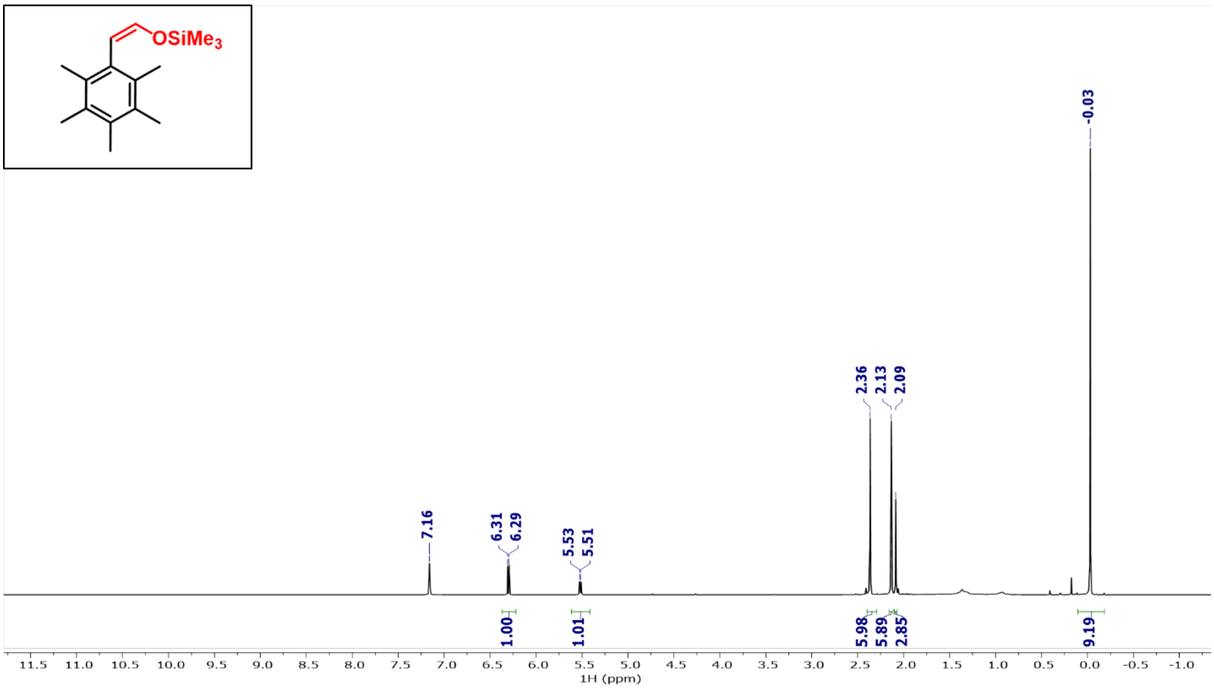

Figure S75 – [1]**H NMR (400 MHz, C_6D_6, 296 K)** spectrum of compound **3f**.

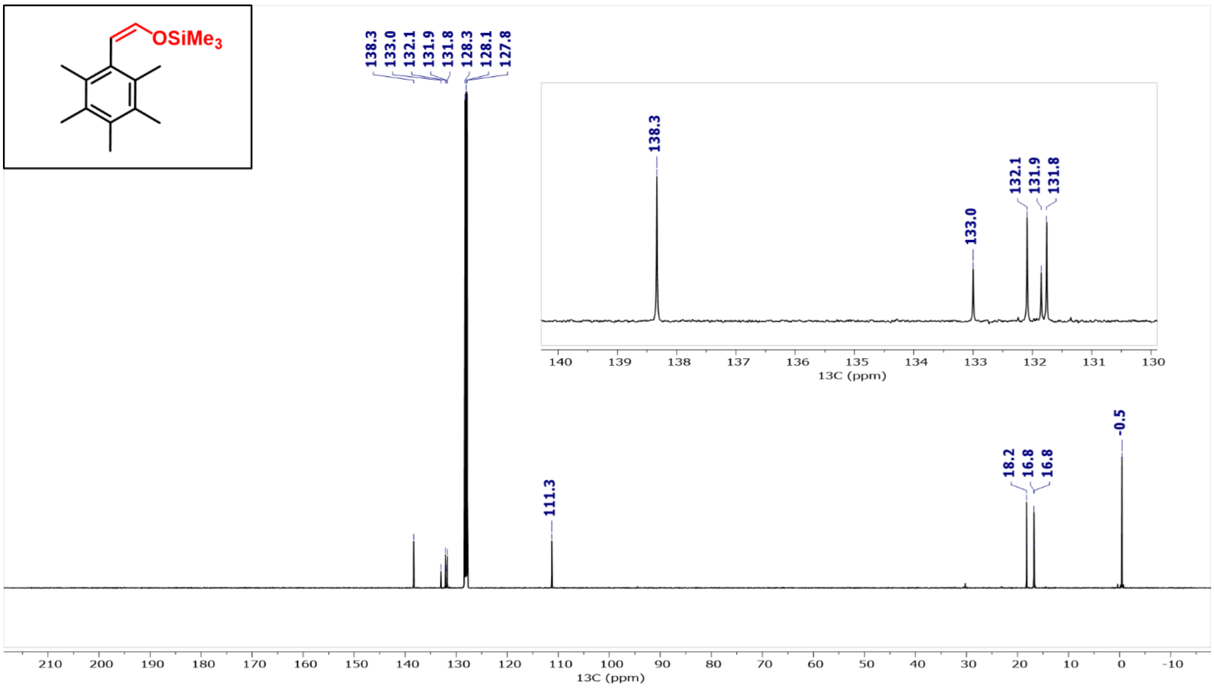

Figure S76 – **$^{13}C\{^1H\}$ NMR (101 MHz, C_6D_6, 296 K)** spectrum of compound **3f**.

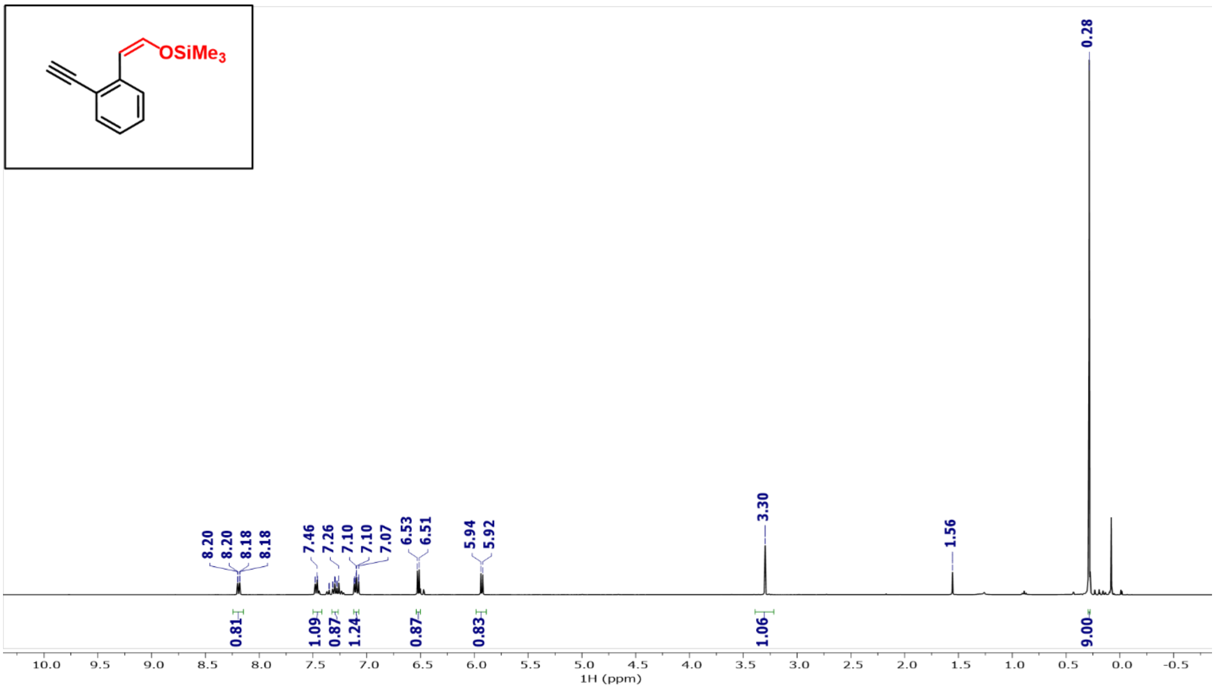

Figure S77 – **^{1}H NMR (400 MHz, $CDCl_3$, 296 K)** spectrum of compound **3g**.

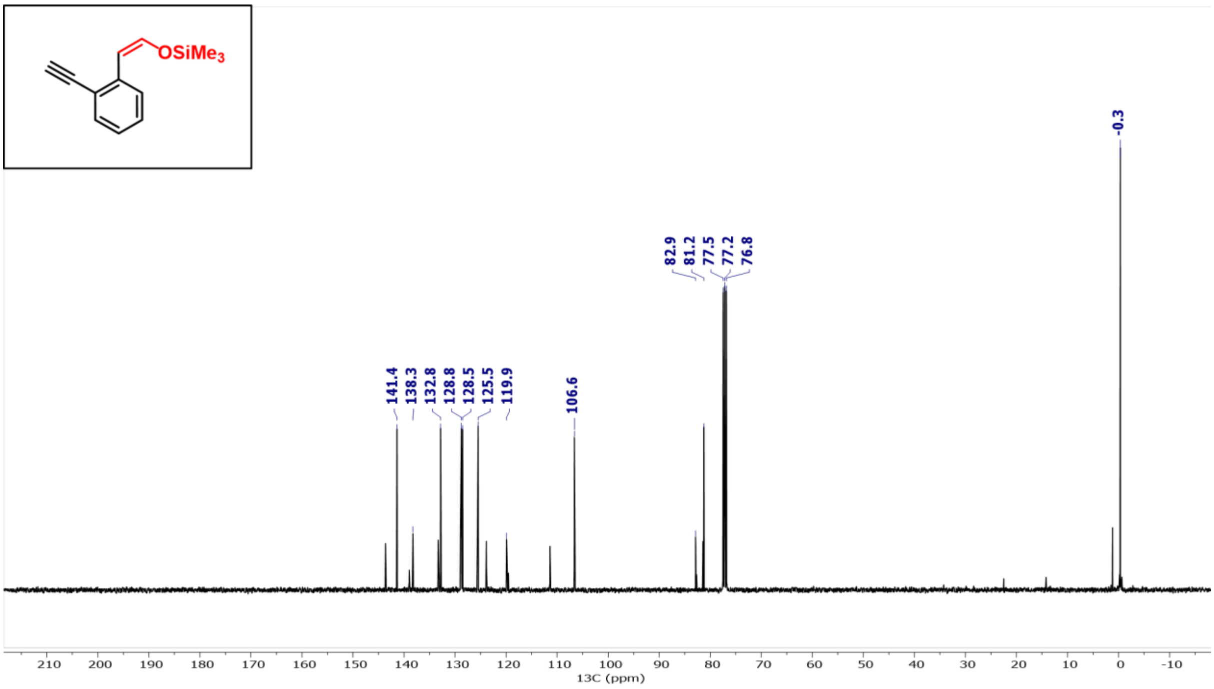

Figure S78 – **^{13}C{^{1}H} NMR (400 MHz, $CDCl_3$, 296 K)** spectrum of compound **3g**.

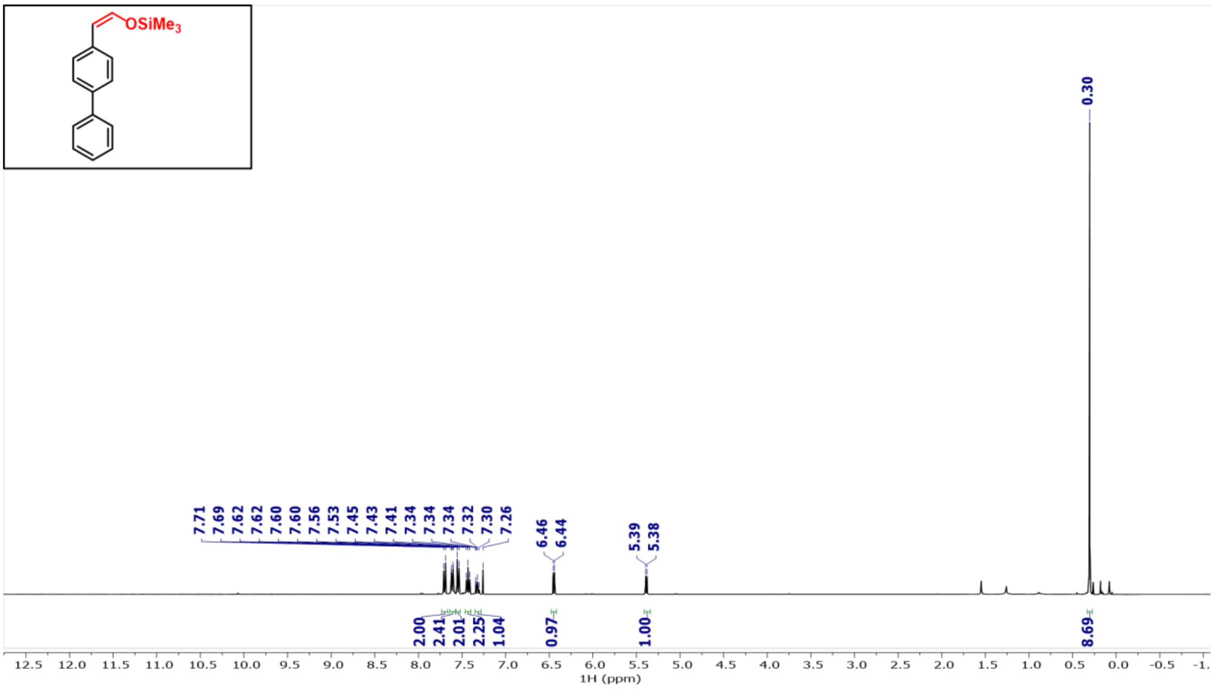

Figure S79 – [1]**H NMR (400 MHz, CDCl₃, 296 K)** spectrum of compound **3h**.

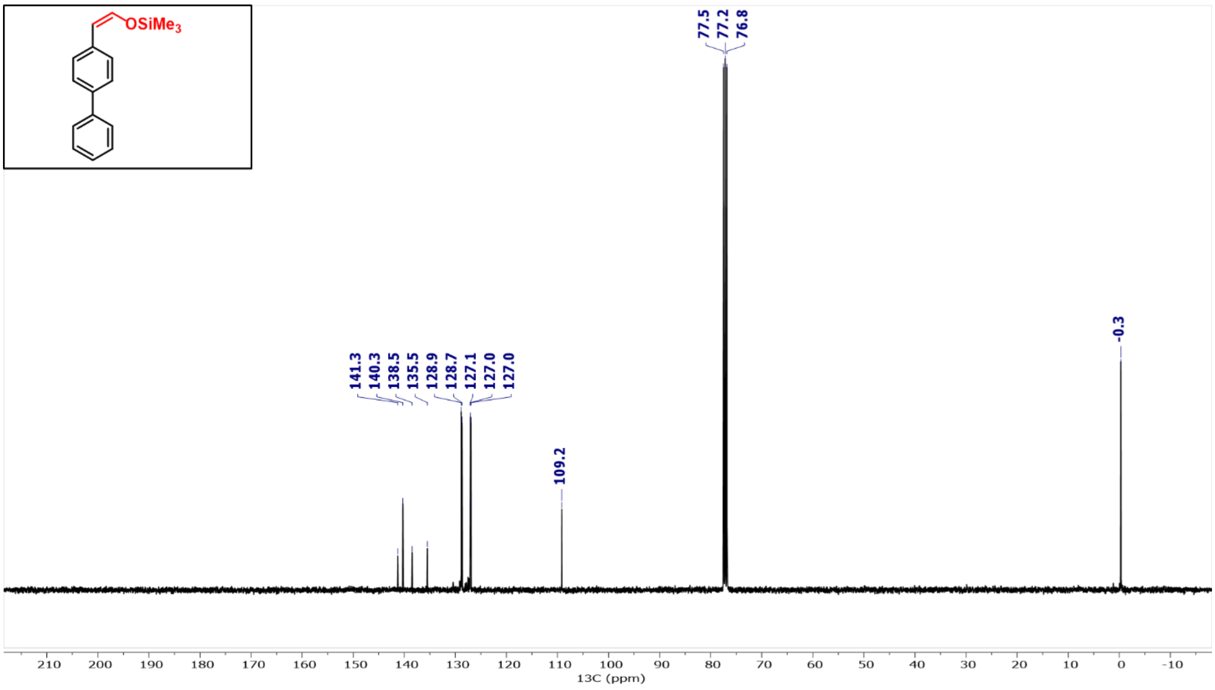

Figure S80 – ^{13}**C{**^{1}**H} NMR (101 MHz, CDCl₃, 296 K)** spectrum of compound **3h**.

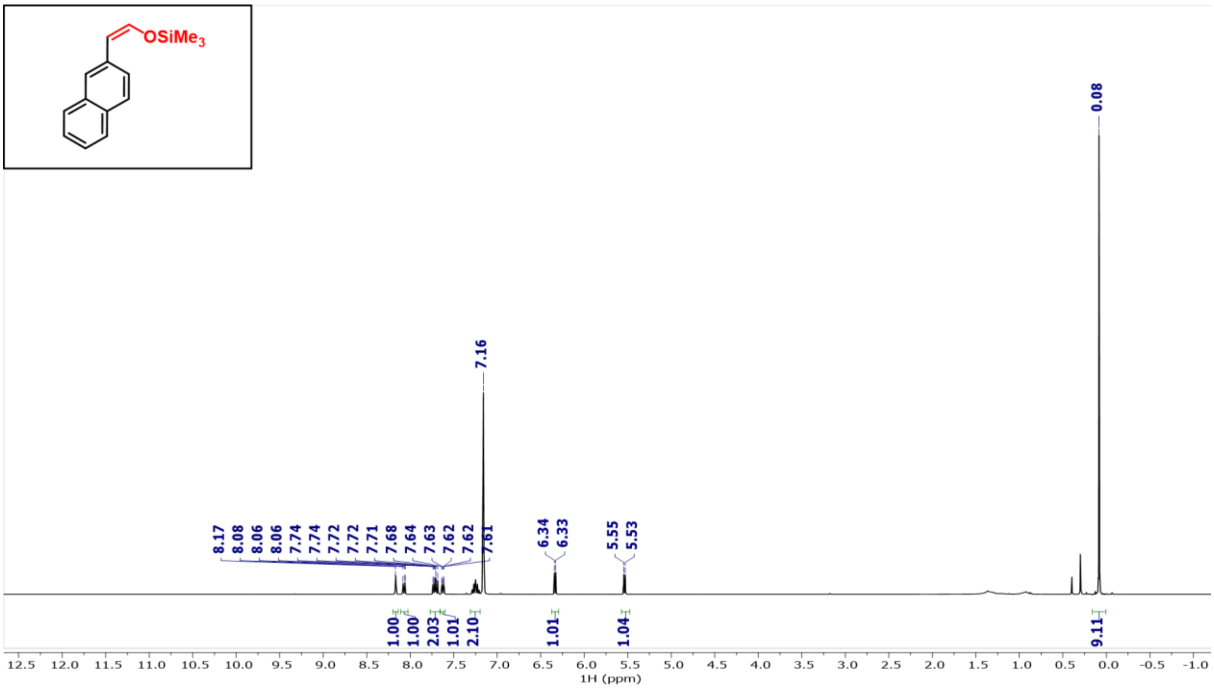

Figure S81 – **^{1}H NMR (400 MHz, C_6D_6, 296 K)** spectrum of compound **3i**.

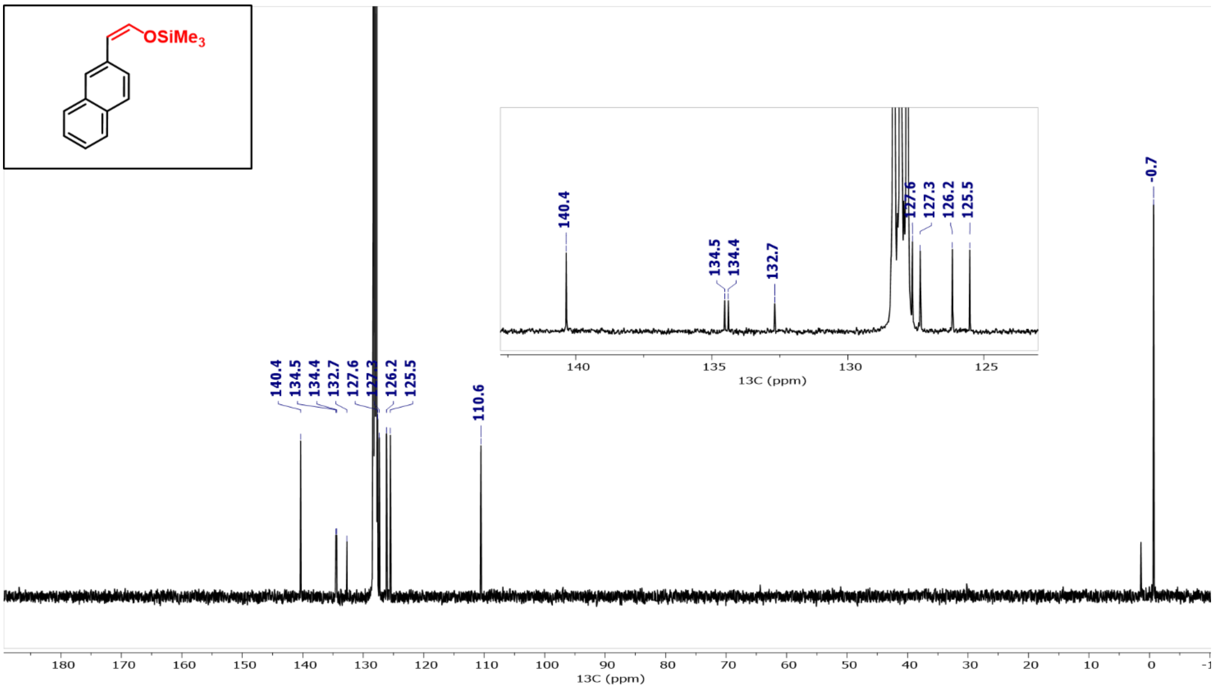

Figure S82 – **^{13}C{^{1}H} NMR (101 MHz, C_6D_6, 296 K**) spectrum of compound **3i**.

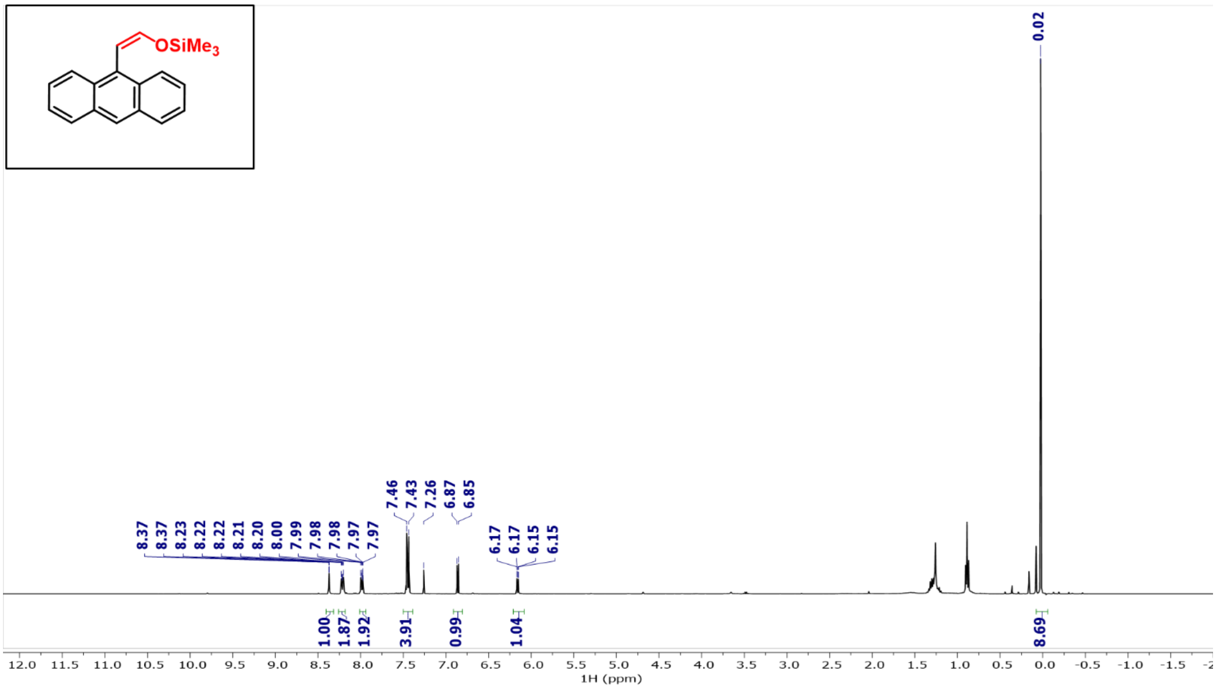

Figure S83 – **^{1}H NMR (400 MHz, $CDCl_3$, 296 K)** spectrum of compound **3j**.

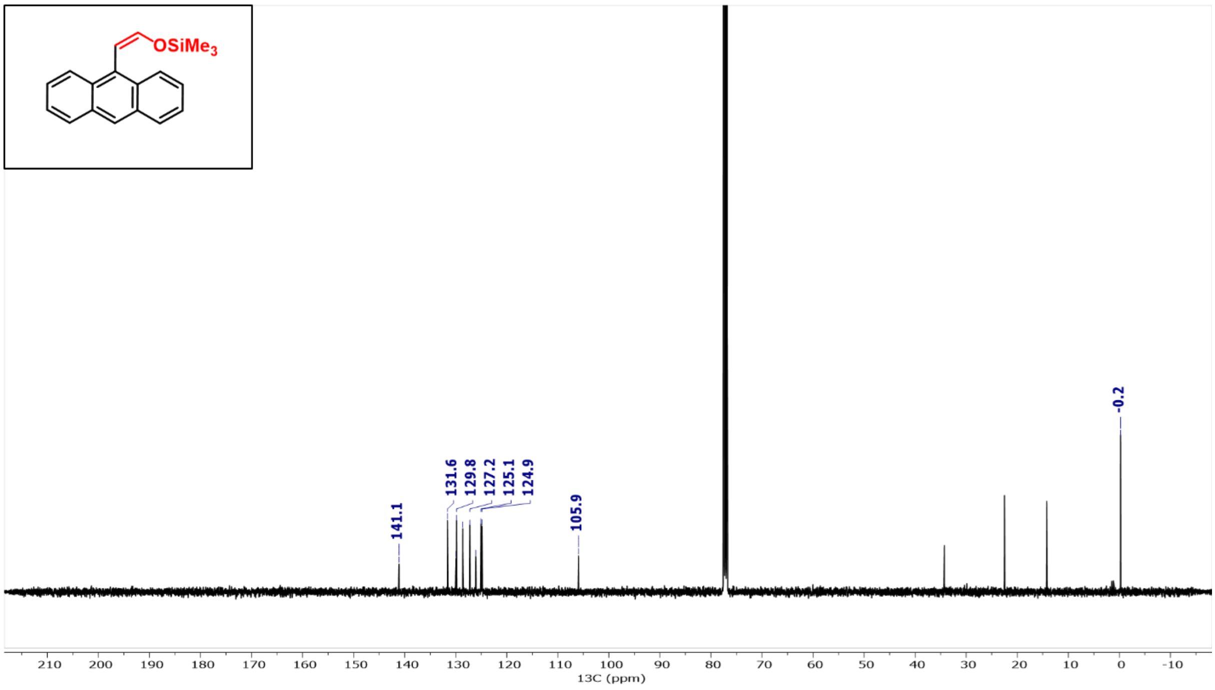

Figure S84 – **$^{13}C\{^1H\}$ NMR (101 MHz, $CDCl_3$, 296 K)** spectrum of compound **3j**.

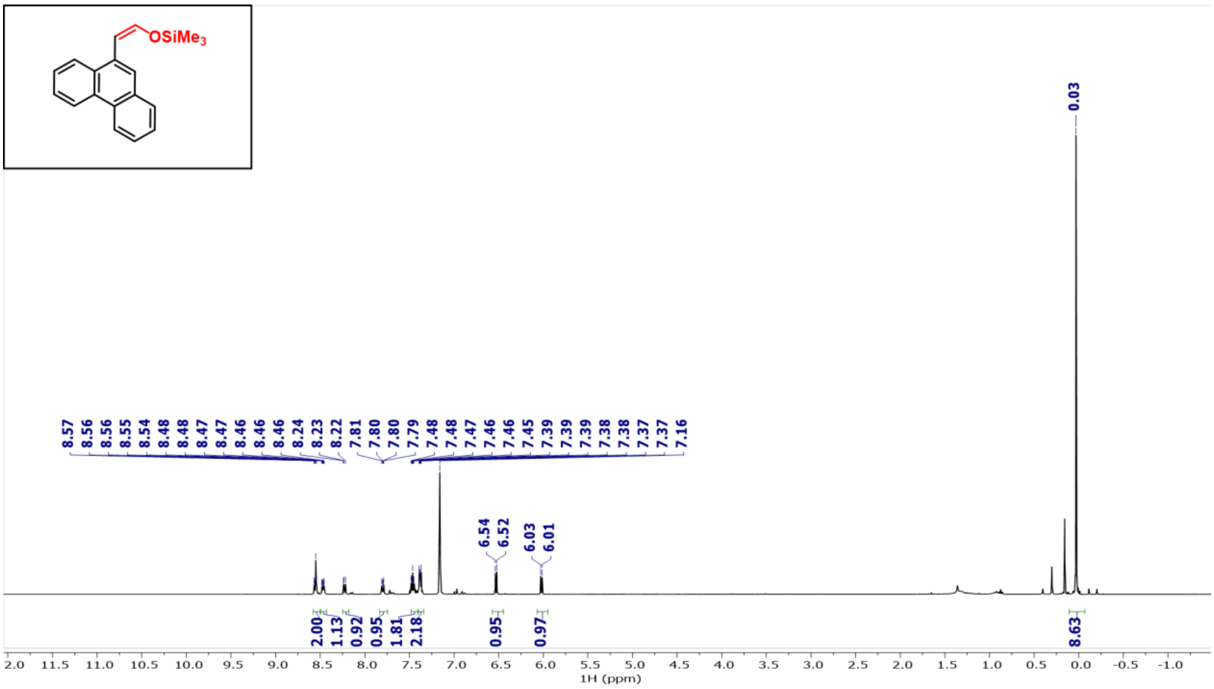

Figure S85 – ^{1}**H NMR (400 MHz, C_6D_6, 296 K)** spectrum of compound **3k**.

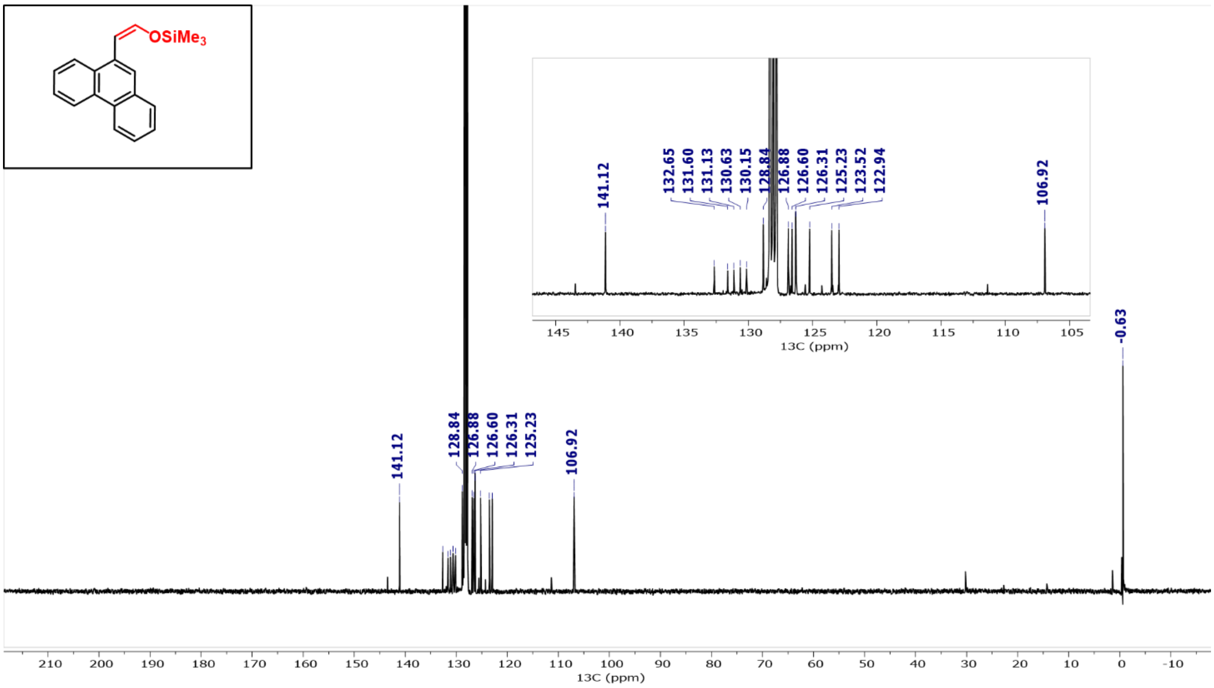

Figure S86 – ^{13}**C{^{1}H} NMR (101 MHz, C_6D_6, 296 K)** spectrum of compound **3k**.

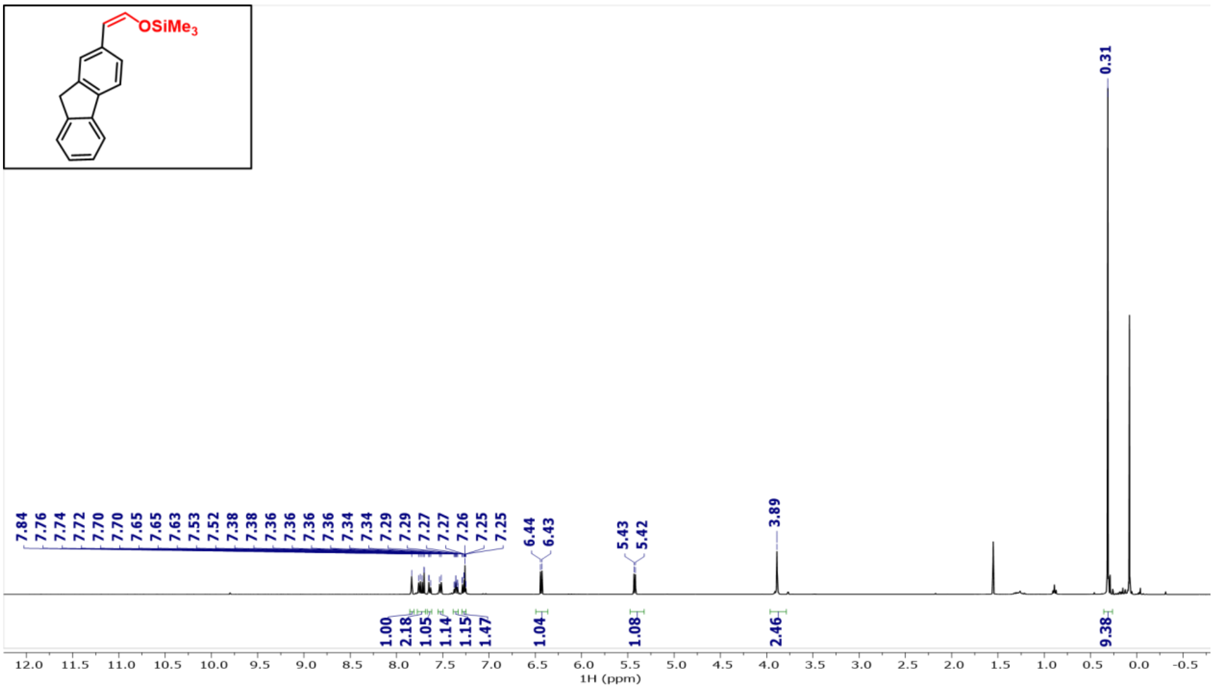

Figure S87 – **^{1}H NMR (400 MHz, $CDCl_3$, 296 K)** spectrum of compound **3l**.

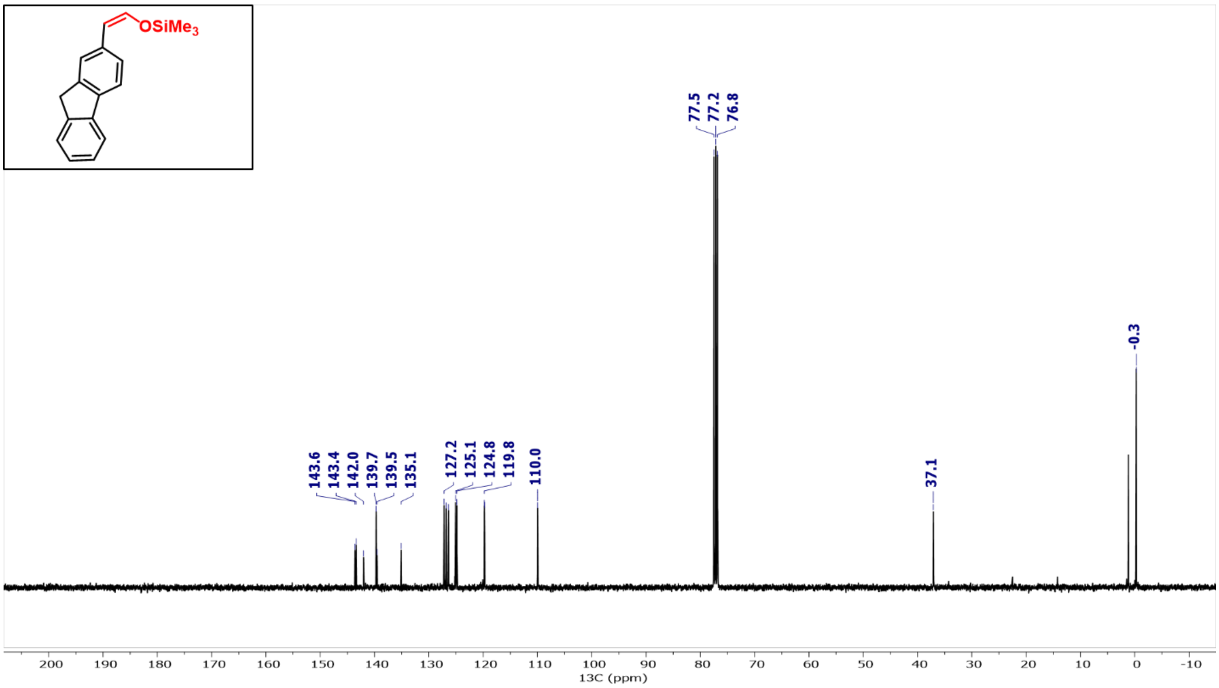

Figure S88 – **^{13}C{^{1}H} NMR (101 MHz, $CDCl_3$, 296 K**) spectrum of compound **3l**.

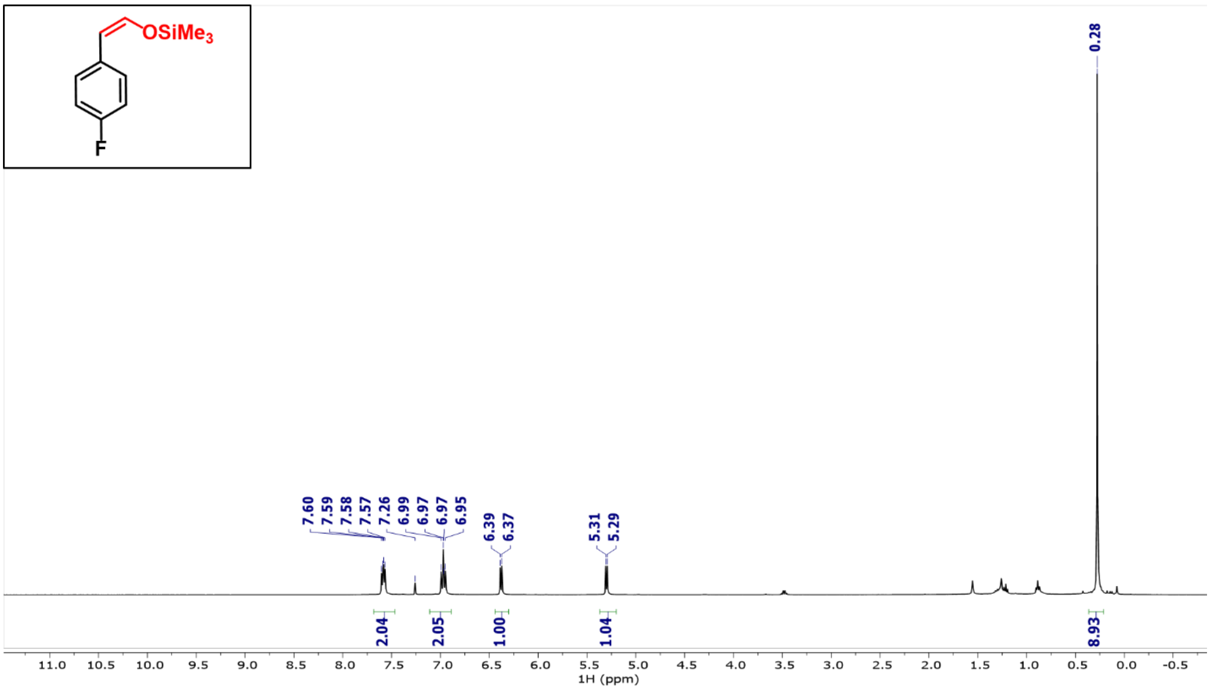

Figure S89 – **^{1}H NMR (400 MHz, CDCl$_3$, 296 K)** spectrum of compound **3m**.

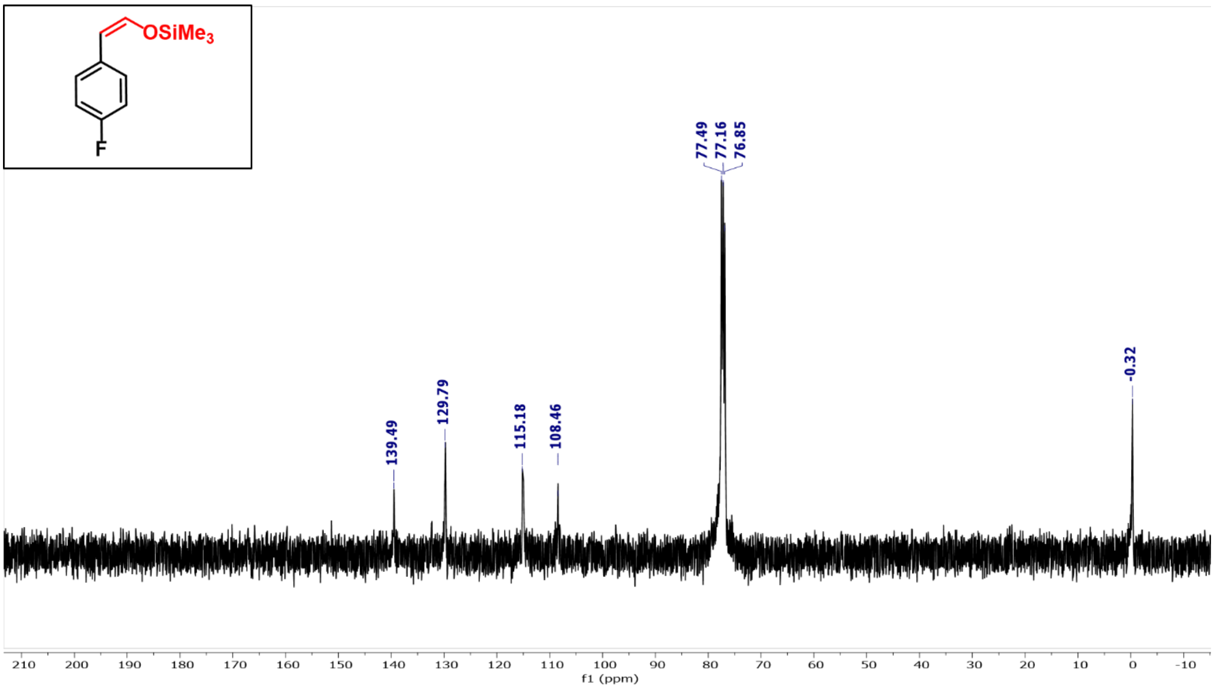

Figure S90 – **^{13}C{^{1}H} NMR (101 MHz, CDCl$_3$, 296 K)** spectrum of compound **3m**.

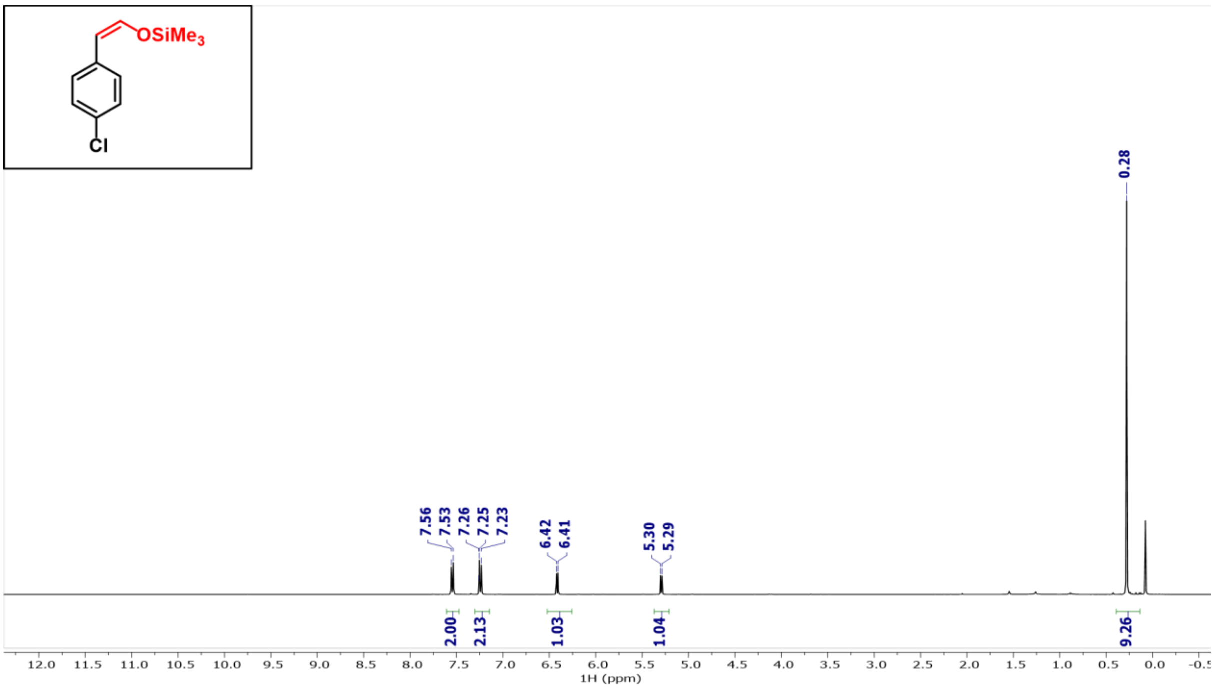

Figure S91 – **^{1}H NMR (400 MHz, $CDCl_3$, 296 K)** spectrum of compound **3n**.

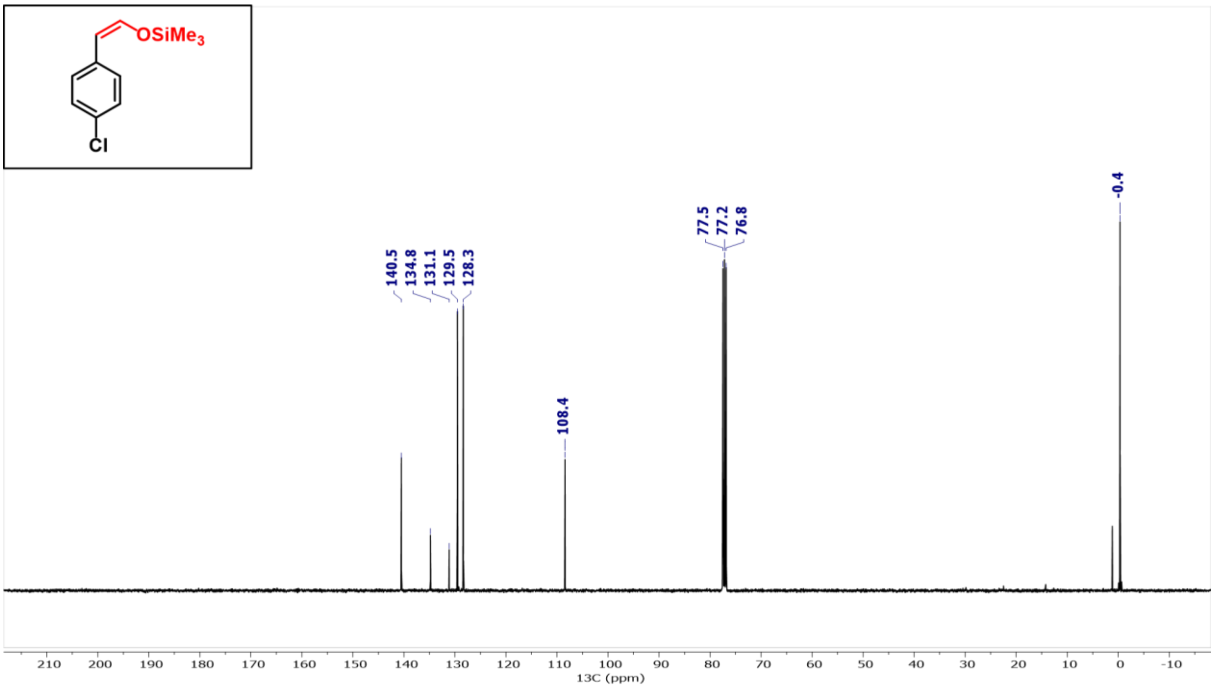

Figure S92 – **^{13}C{^{1}H} NMR (101 MHz, $CDCl_3$, 296 K)** spectrum of compound **3n**.

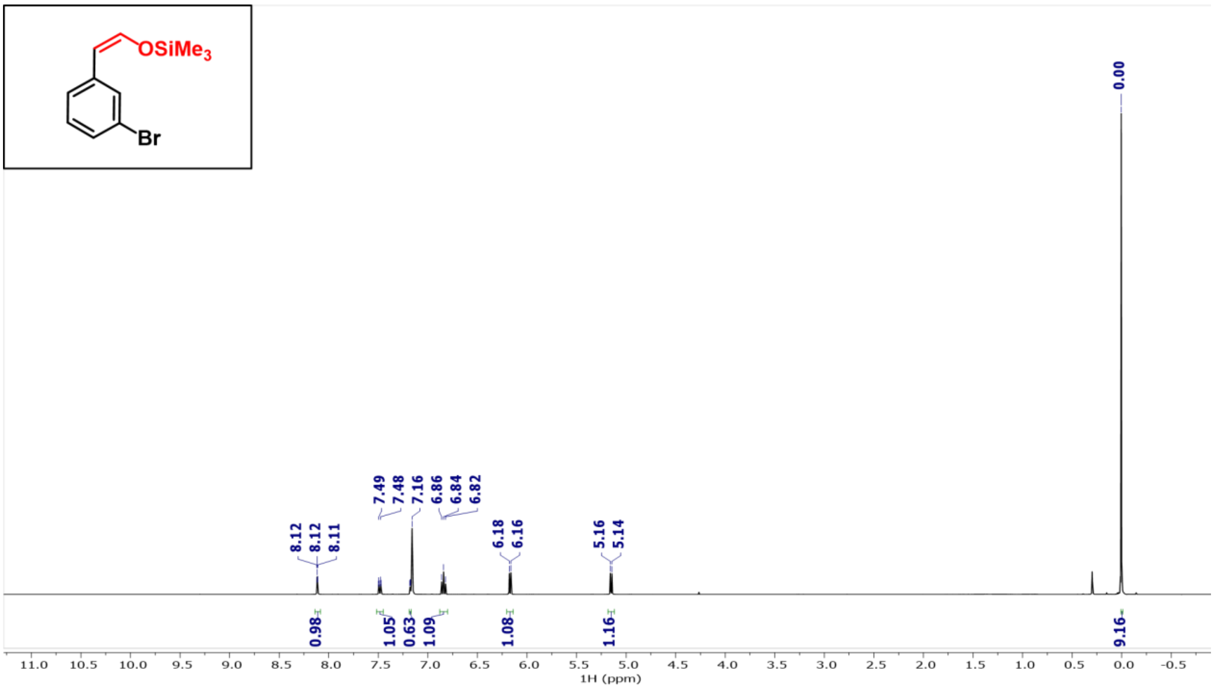

Figure S93 – **^{1}H NMR (400 MHz, C_6D_6, 296 K)** spectrum of compound **3o**.

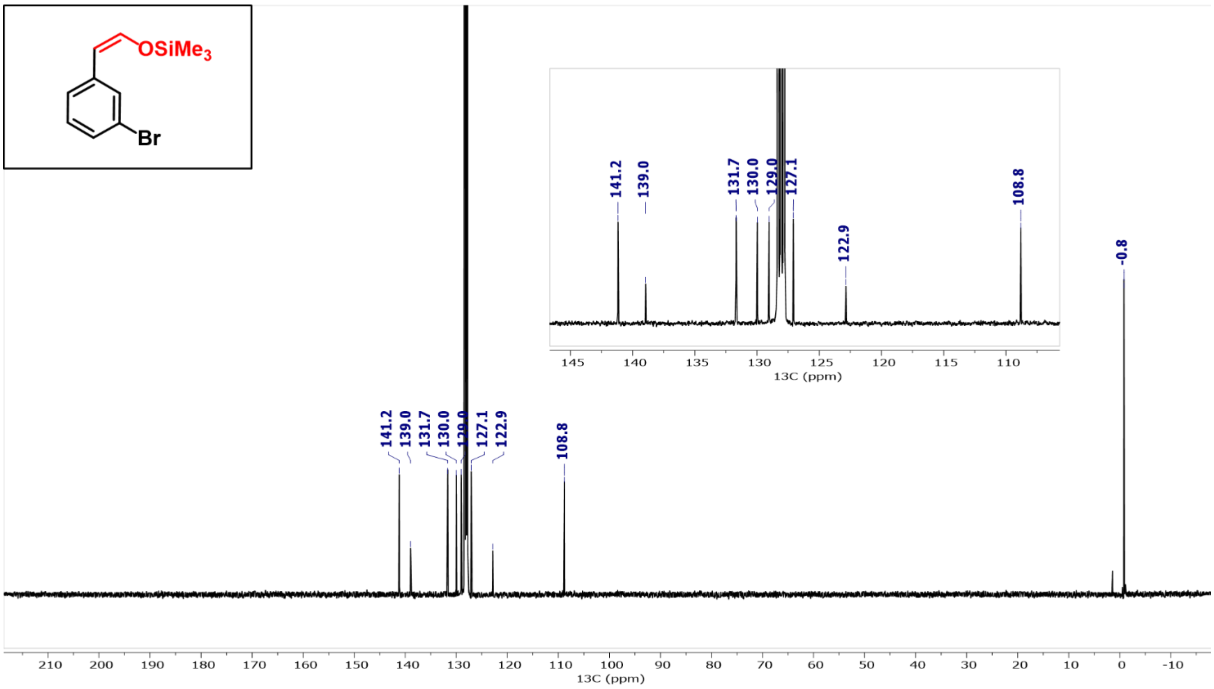

Figure S94 – **^{13}C{^{1}H} NMR (101 MHz, C_6D_6, 296 K)** spectrum of compound **3o**.

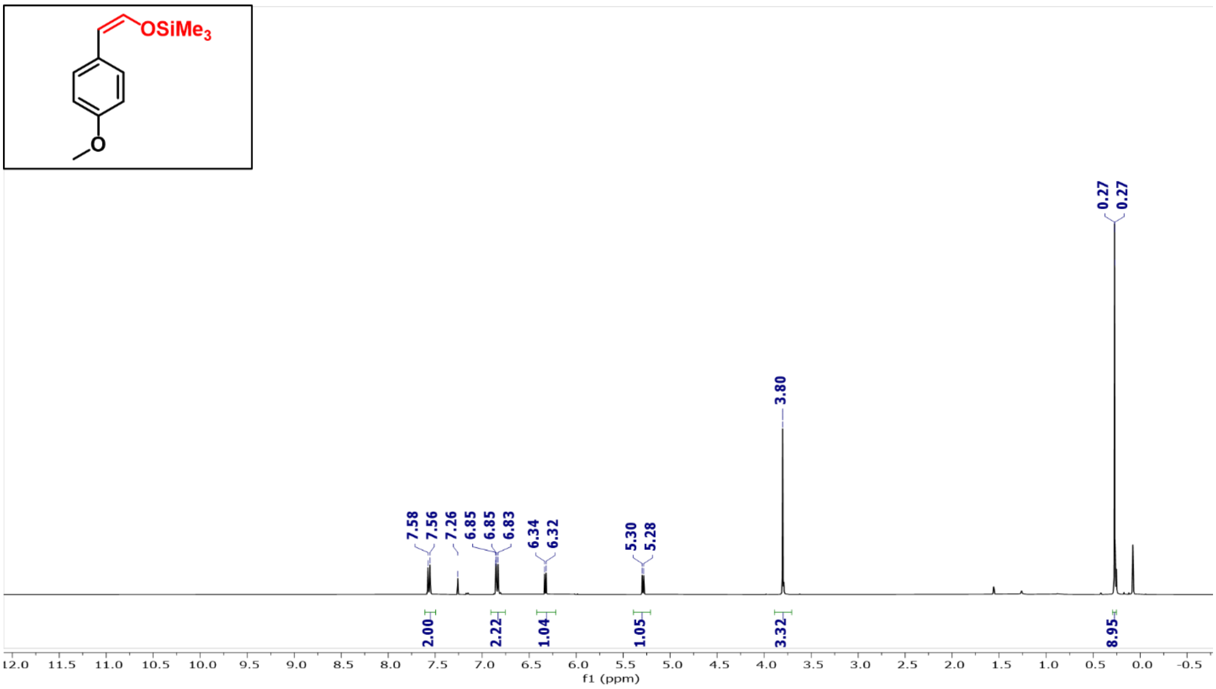

Figure S95 – **^{1}H NMR (400 MHz, CDCl₃, 296 K)** spectrum of compound **3p**.

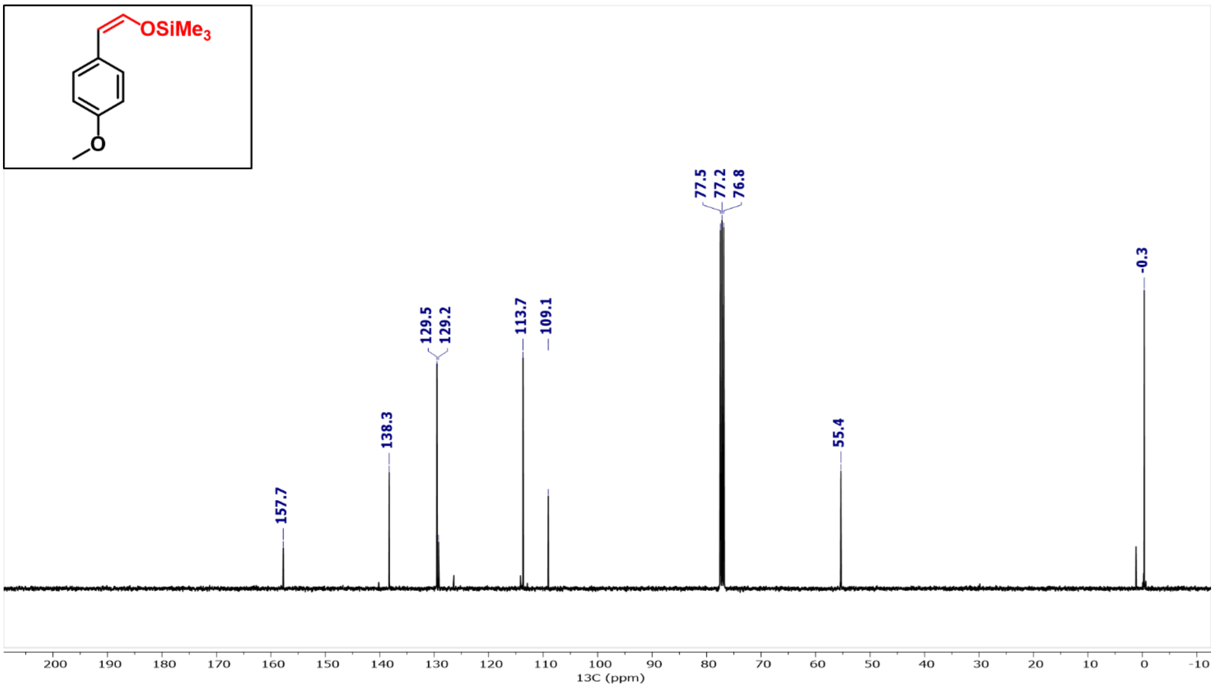

Figure S96 – **^{13}C{^{1}H} NMR (101 MHz, CDCl₃, 296 K)** spectrum of compound **3p**.

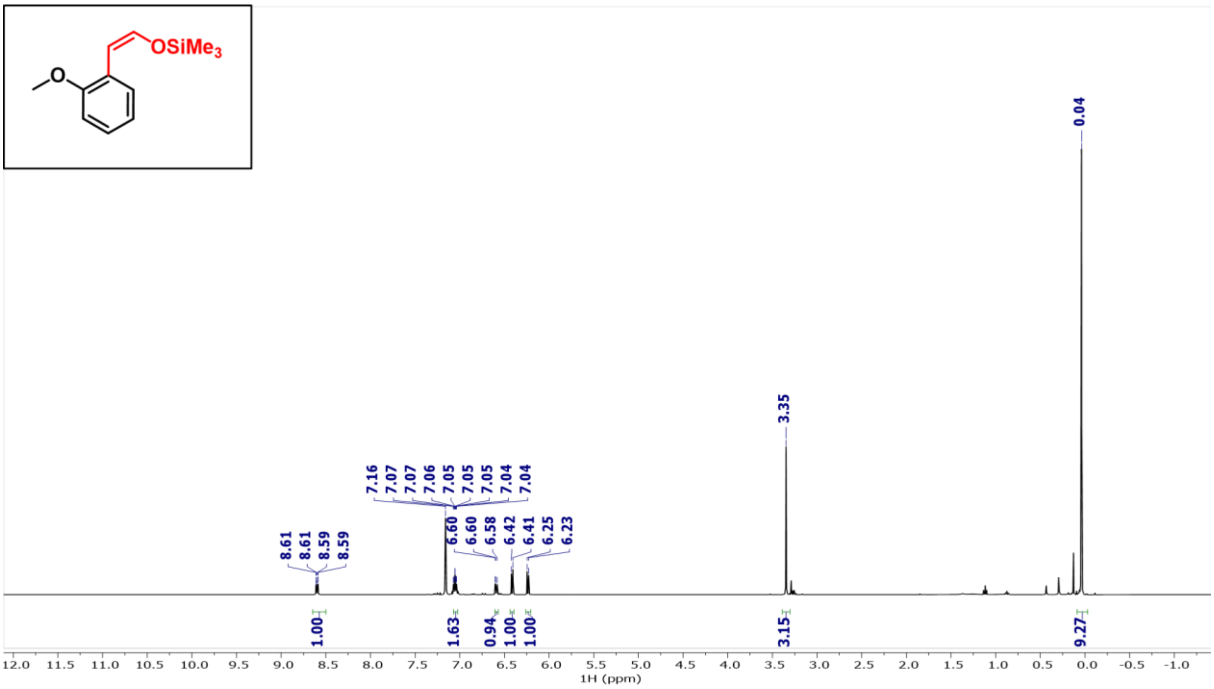

Figure S97 – [1]**H NMR (400 MHz, C_6D_6, 296 K)** spectrum of compound **3q**.

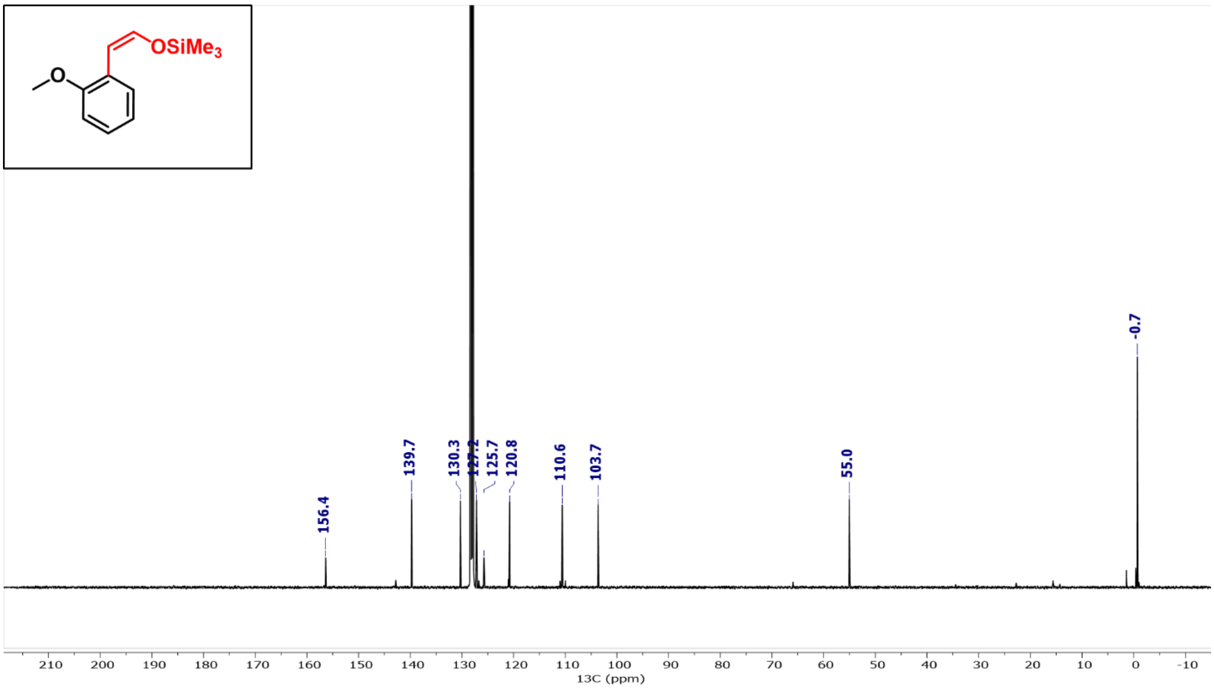

Figure S98 – [13]**C{^{1}H} NMR (101 MHz, C_6D_6, 296 K)** spectrum of compound **3q**.

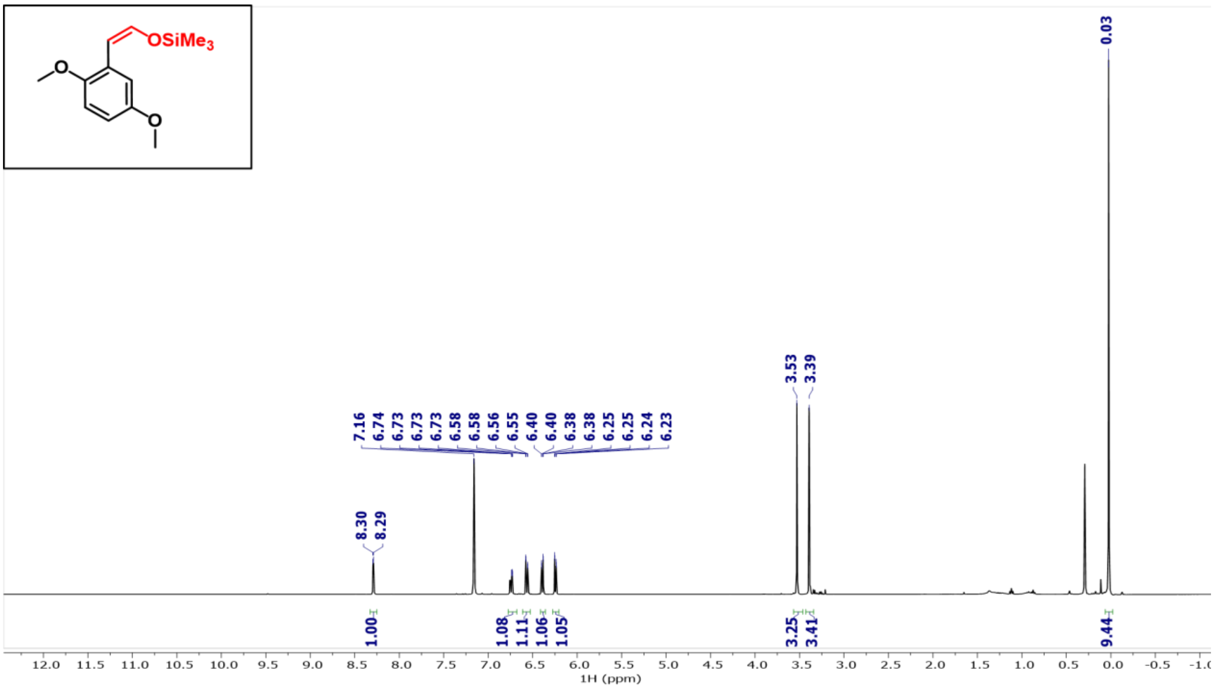

Figure S99 – **^{1}H NMR (400 MHz, C_6D_6, 296 K)** spectrum of compound **3r**.

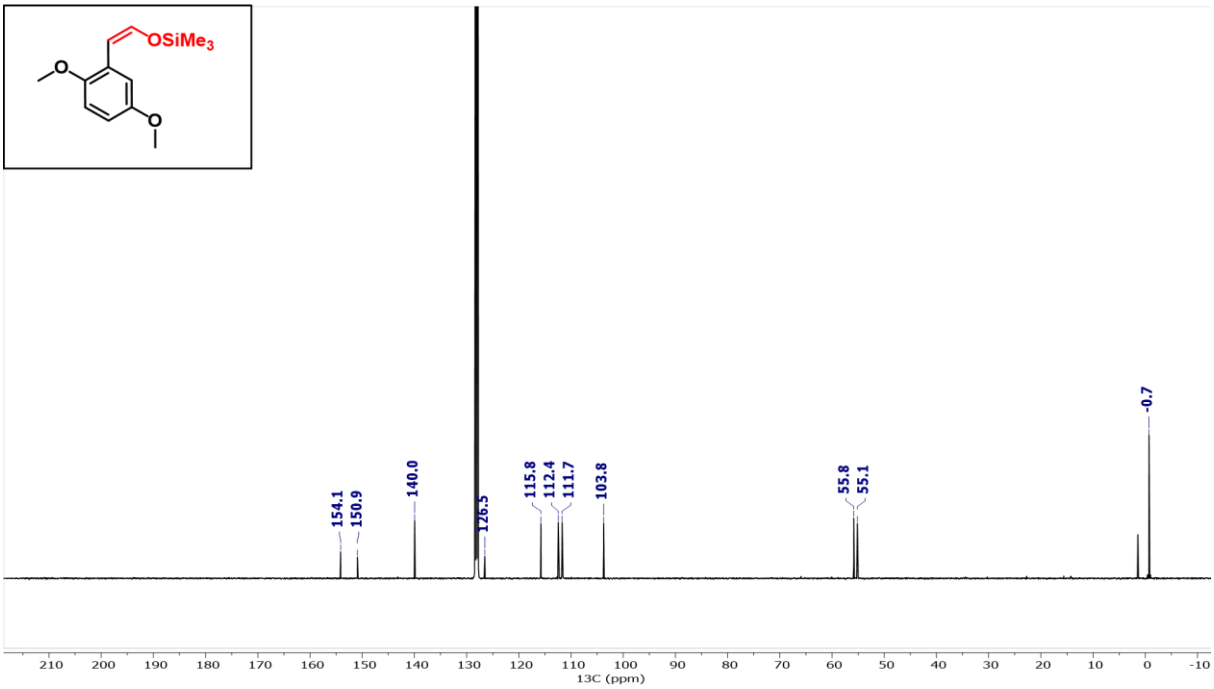

Figure S100 – **^{13}C{^{1}H} NMR (101 MHz, C_6D_6, 296 K)** spectrum of compound **3r**.

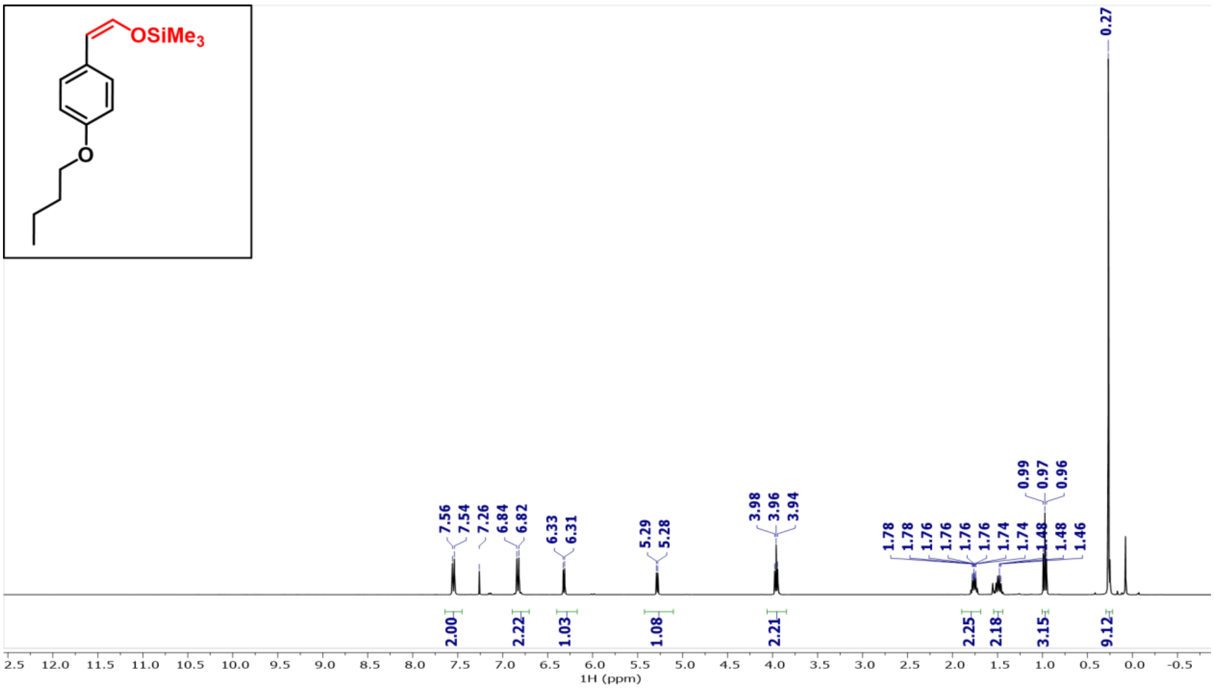

Figure S101 – **^{1}H NMR (400 MHz, CDCl$_3$, 296 K)** spectrum of compound **3s**.

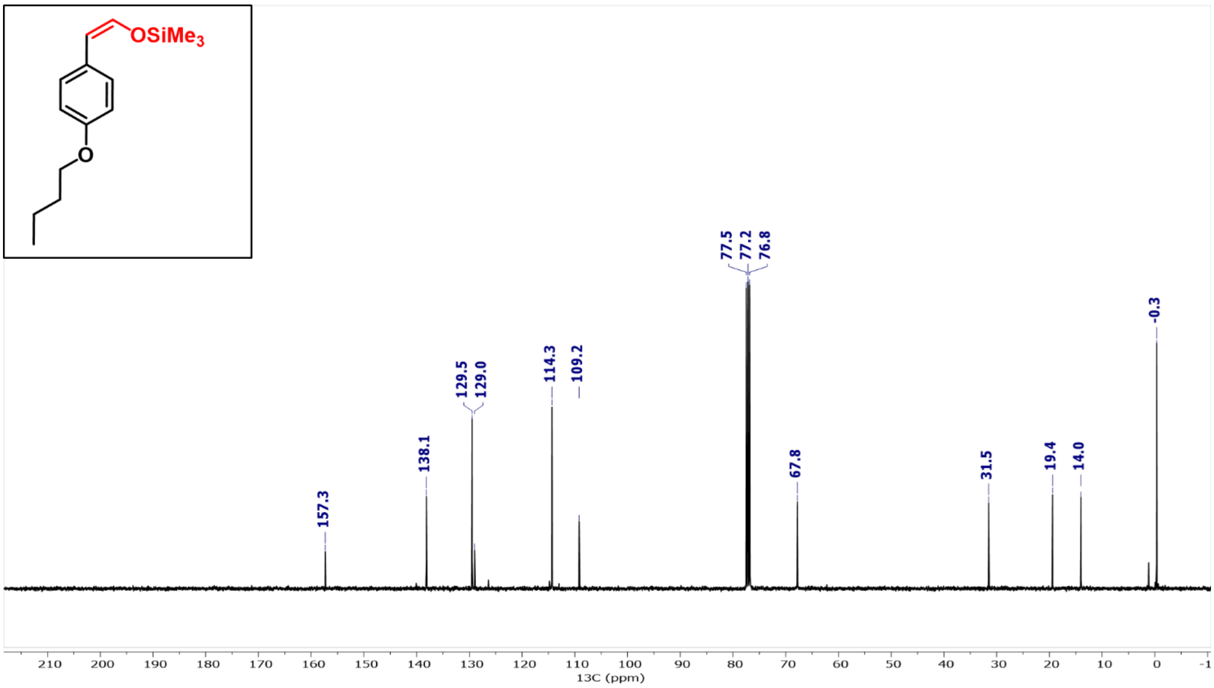

Figure S102 – **^{13}C{^{1}H} NMR (101 MHz, CDCl$_3$, 296 K)** spectrum of compound **3s**.

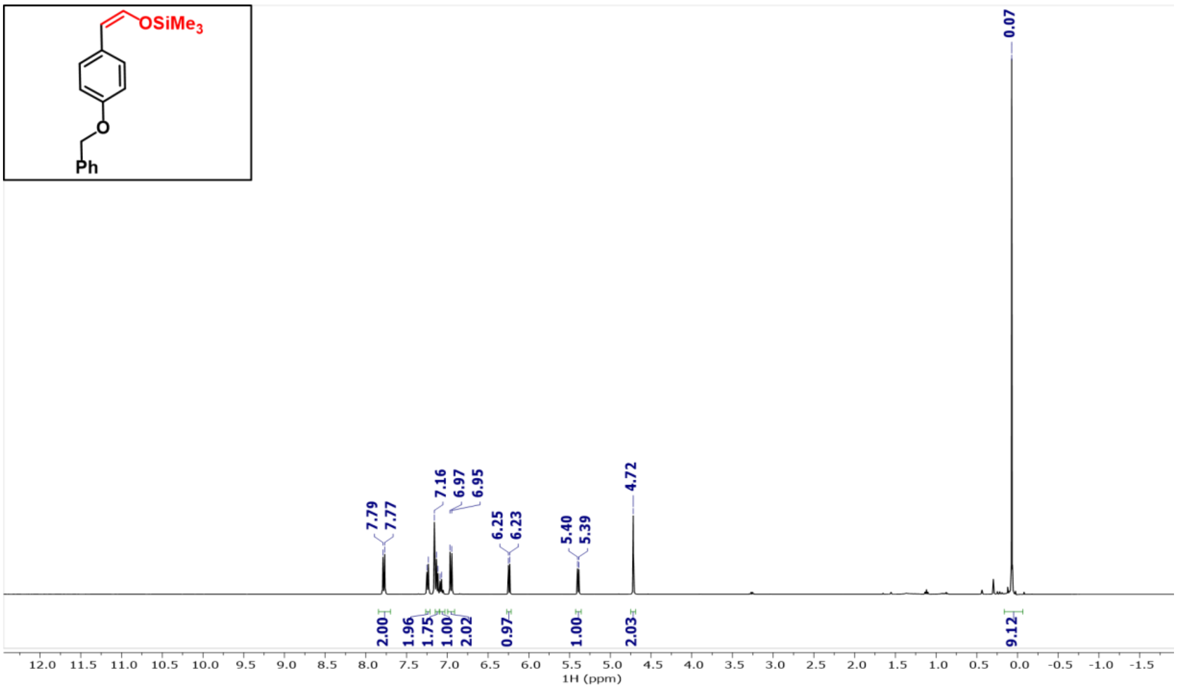

Figure S103 – **^{1}H NMR (400 MHz, C_6D_6, 296 K)** spectrum of compound **3t**.

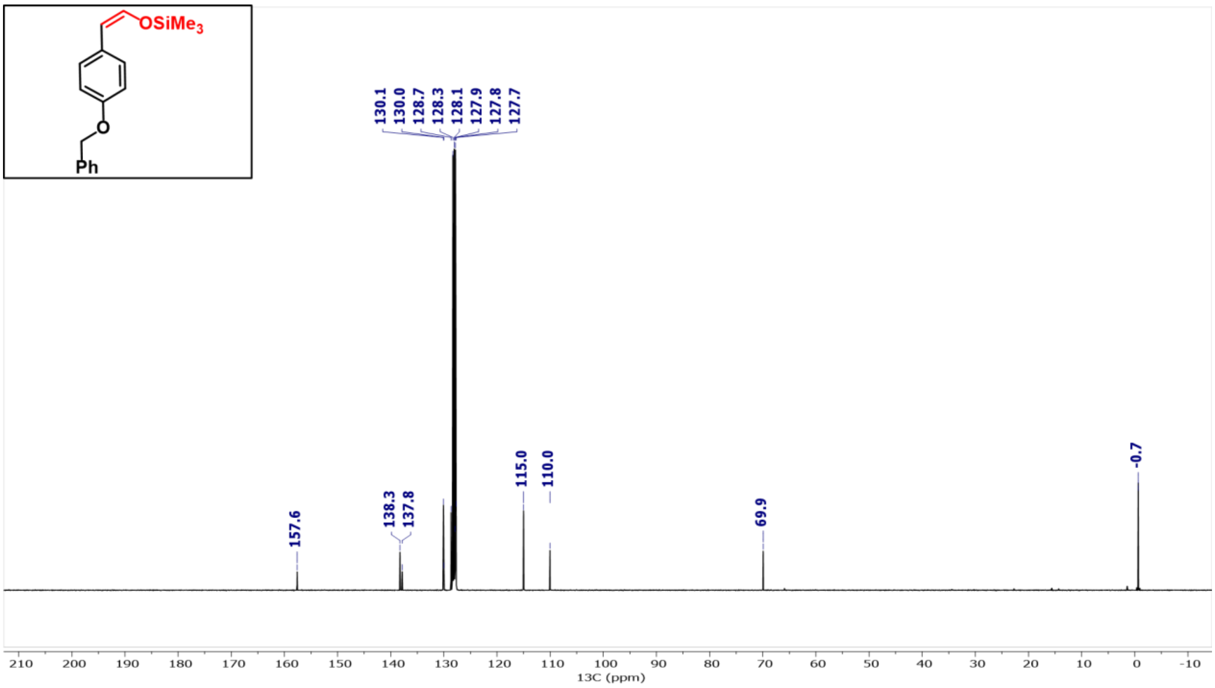

Figure S104 – **^{13}C{^{1}H} NMR (101 MHz, C_6D_6, 296 K)** spectrum of compound **3t**.

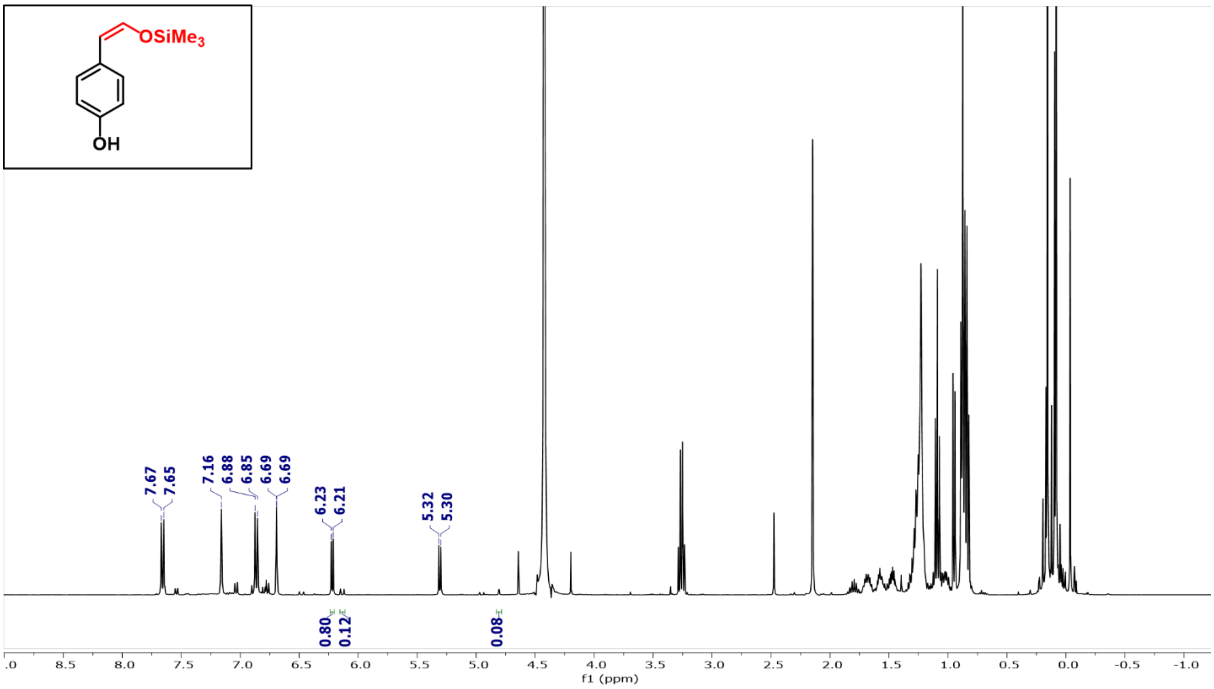

Figure S105 – **^{1}H NMR (400 MHz, C_6D_6, 296 K)** crude NMR spectrum of compound **3u**.

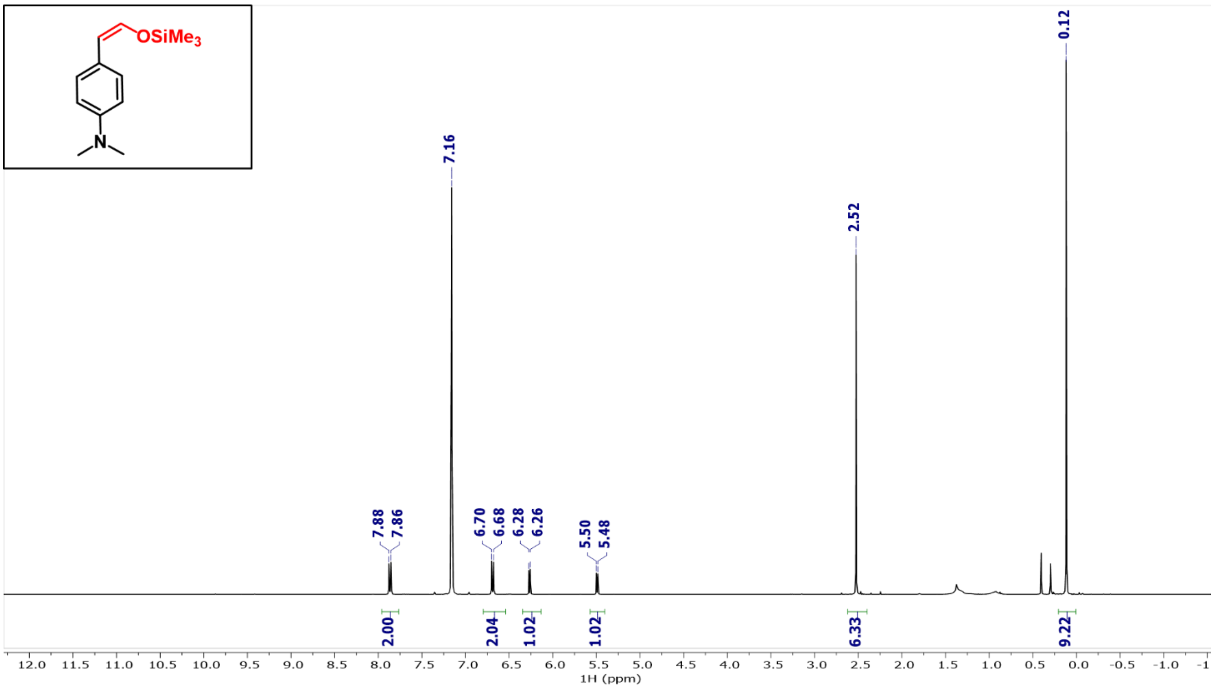

Figure S106 – **^{1}H NMR (400 MHz, C_6D_6, 296 K)** spectrum of compound **3v**.

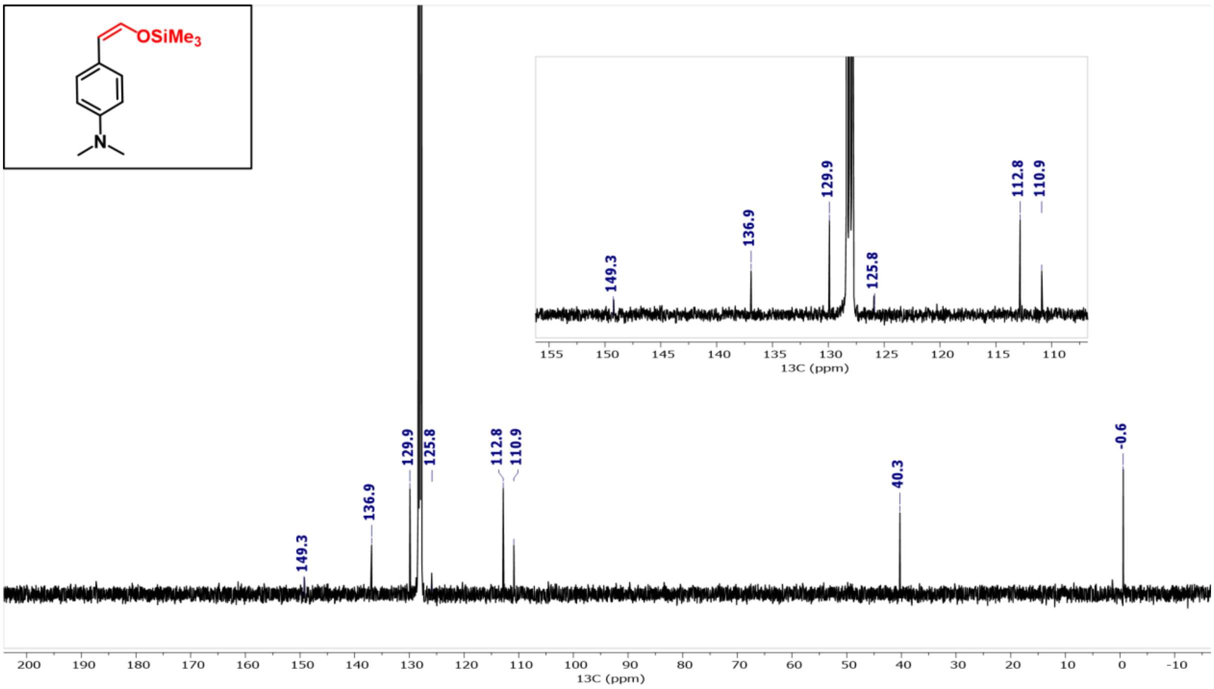

Figure S107 – **$^{13}C\{^{1}H\}$ NMR (101 MHz, C_6D_6, 296 K)** spectrum of compound **3v**.

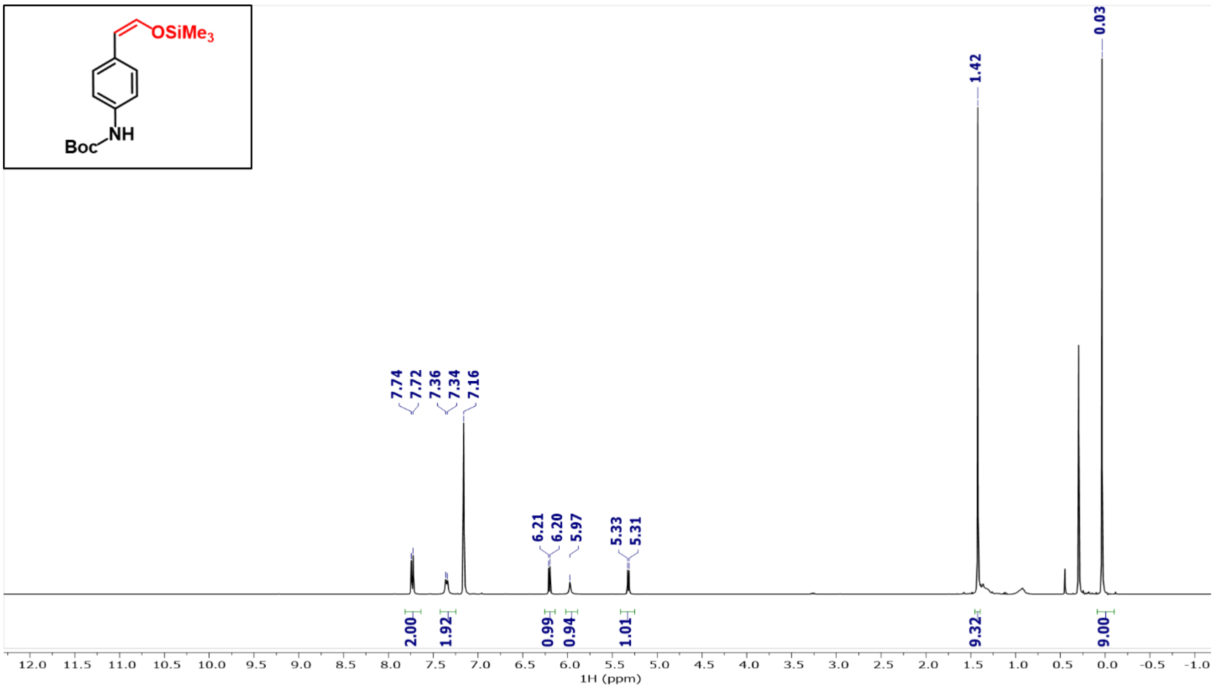

Figure S108 – **^{1}H NMR (400 MHz, C_6D_6, 296 K)** spectrum of compound **3w**.

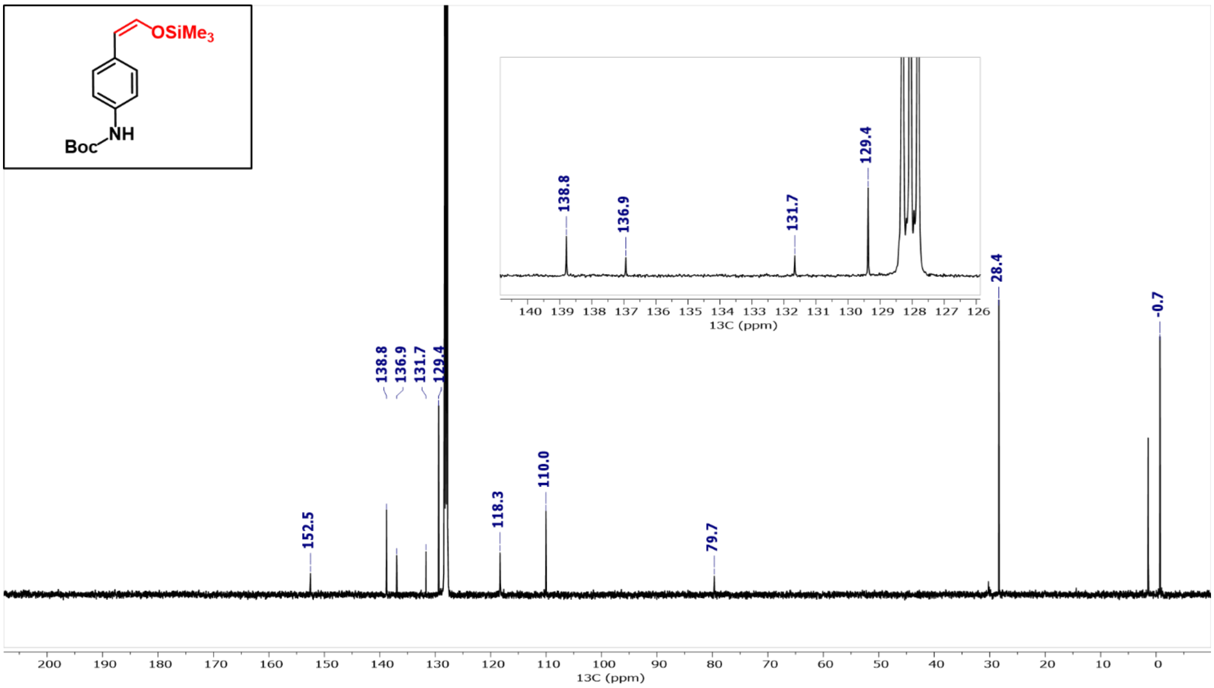

Figure S109 – **^{13}C{^{1}H} NMR (101 MHz, C_6D_6, 296 K)** spectrum of compound **3w**.

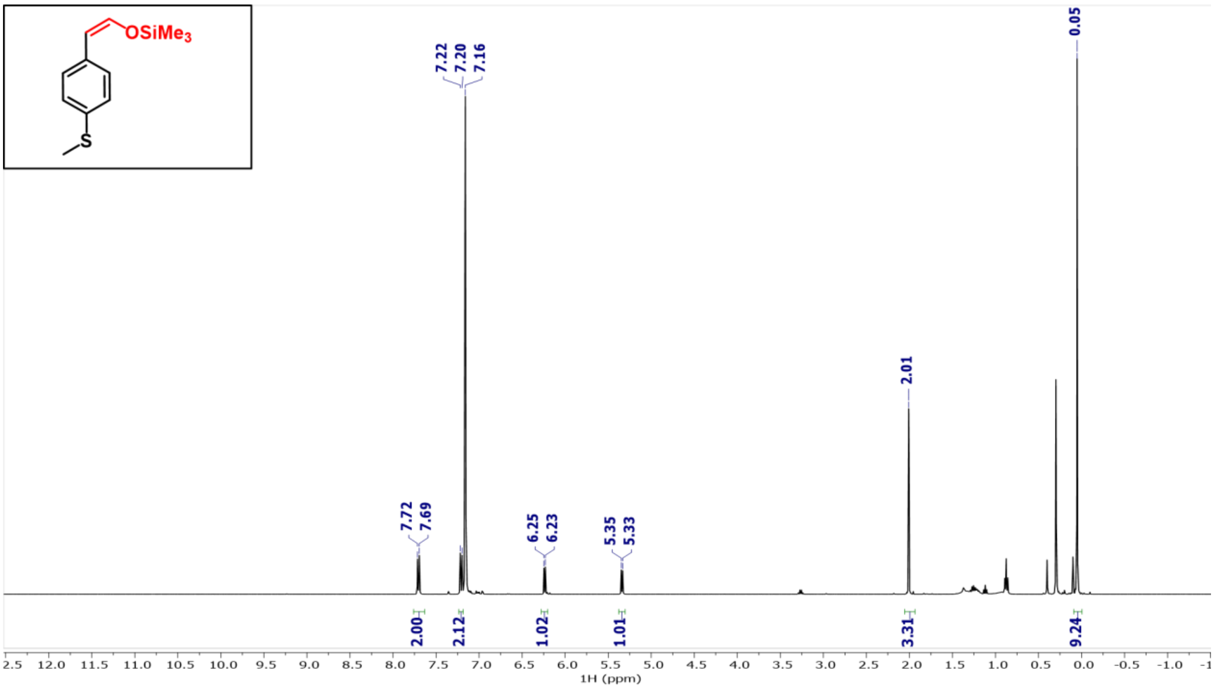

Figure S110 – **^{1}H NMR (400 MHz, C_6D_6, 296 K)** spectrum of compound **3x**.

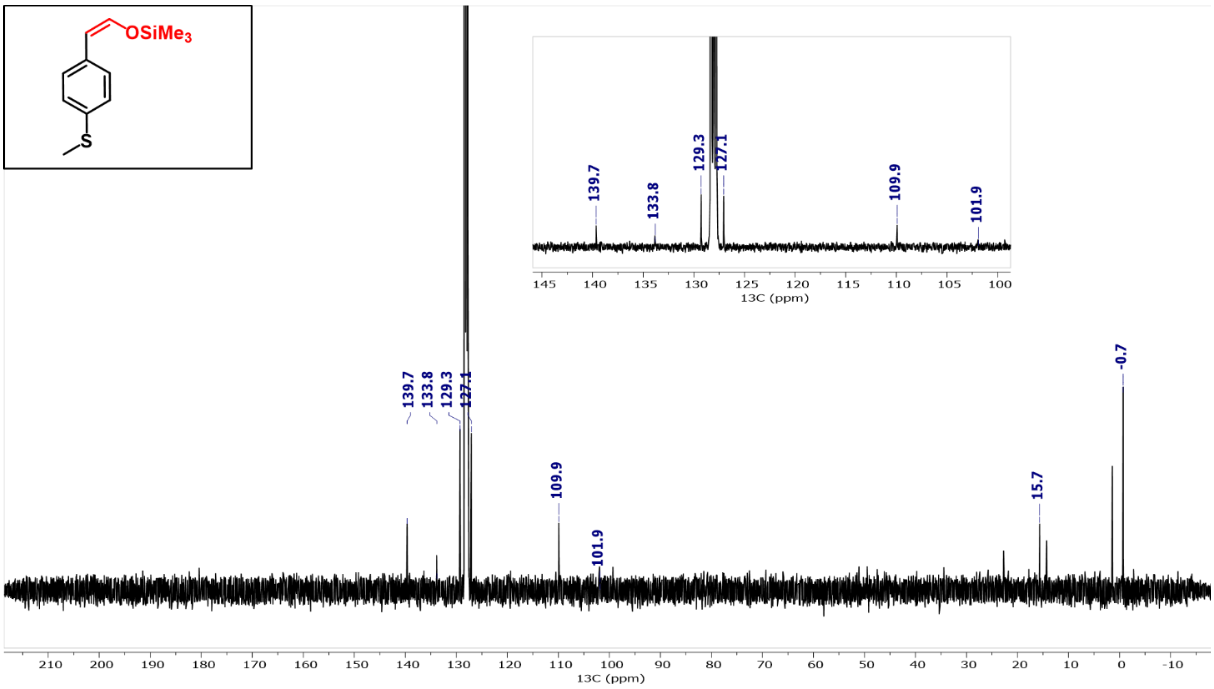

Figure S111 – **$^{13}C\{^{1}H\}$ NMR (101 MHz, C_6D_6, 296 K)** spectrum of compound **3x**.

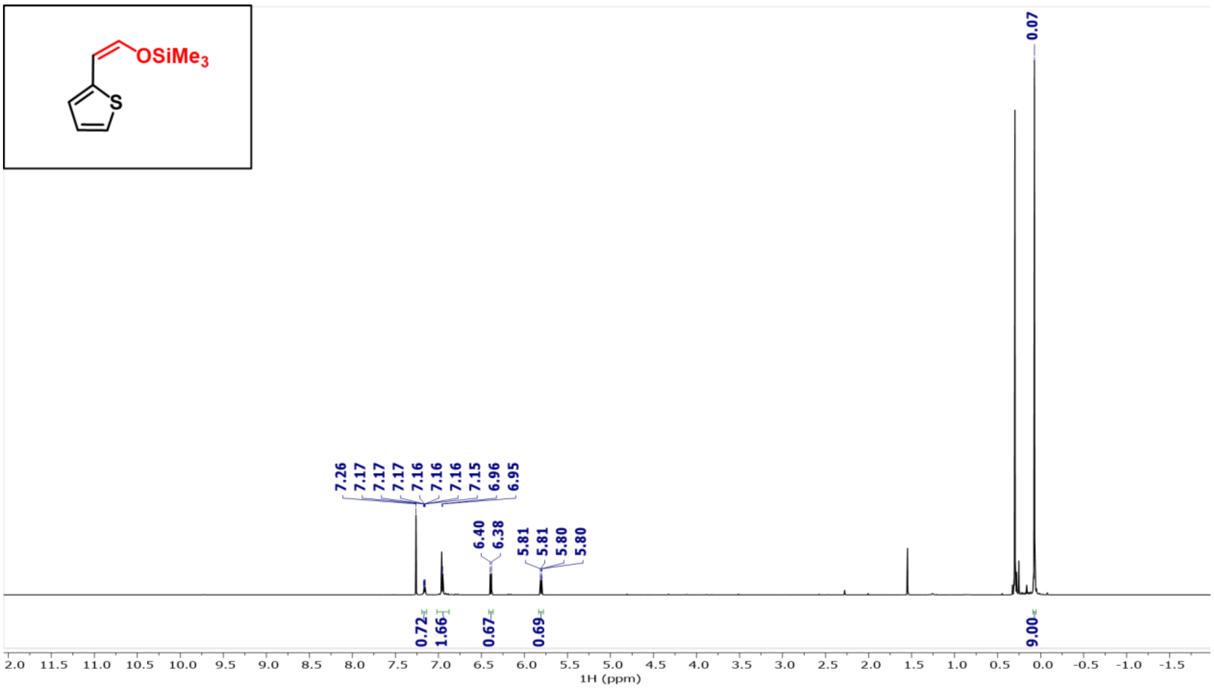

Figure S112 – **^{1}H NMR (400 MHz, CDCl$_3$, 296 K)** spectrum of compound **3y**.

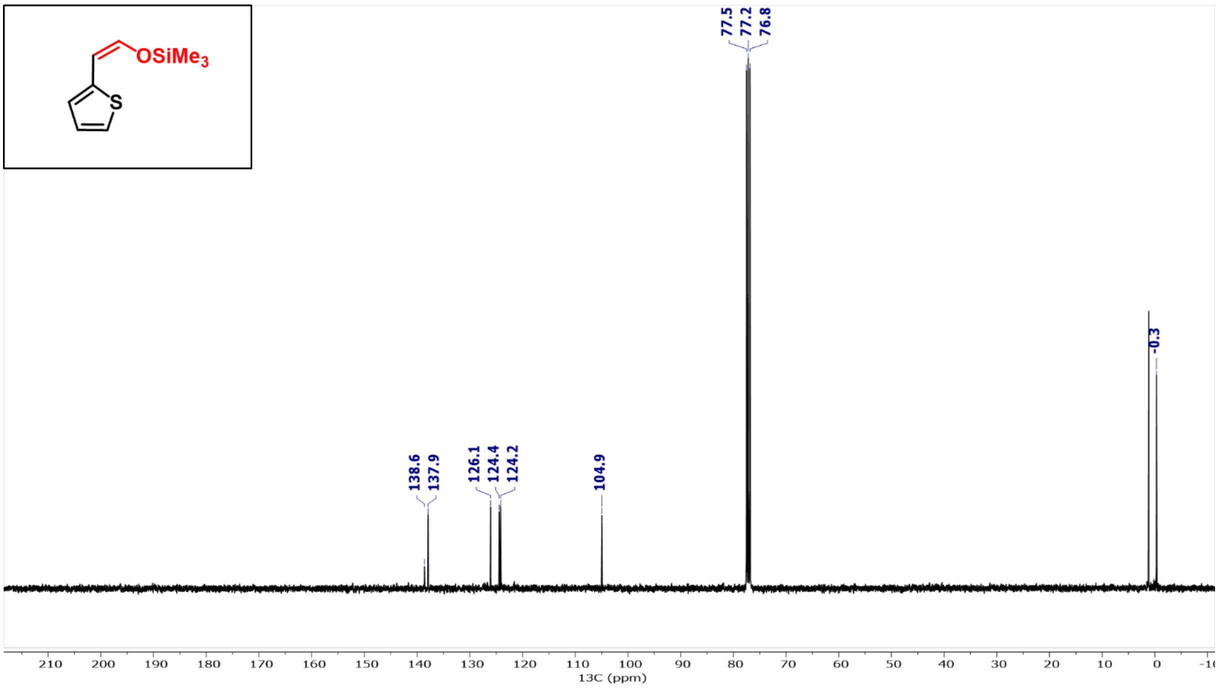

Figure S113 – **^{13}C{^{1}H} NMR (101 MHz, CDCl$_3$, 296 K)** spectrum of compound **3y**.

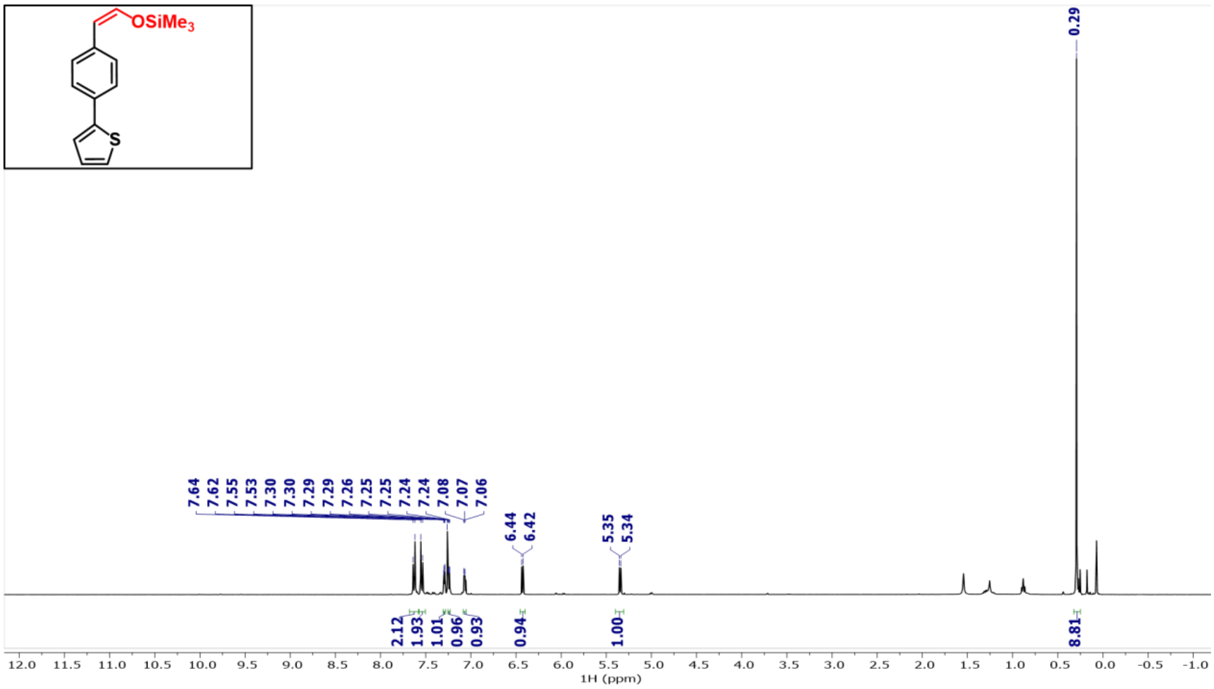

Figure S114 – ^{1}**H NMR (400 MHz, CDCl$_3$, 296 K)** spectrum of compound **3z**.

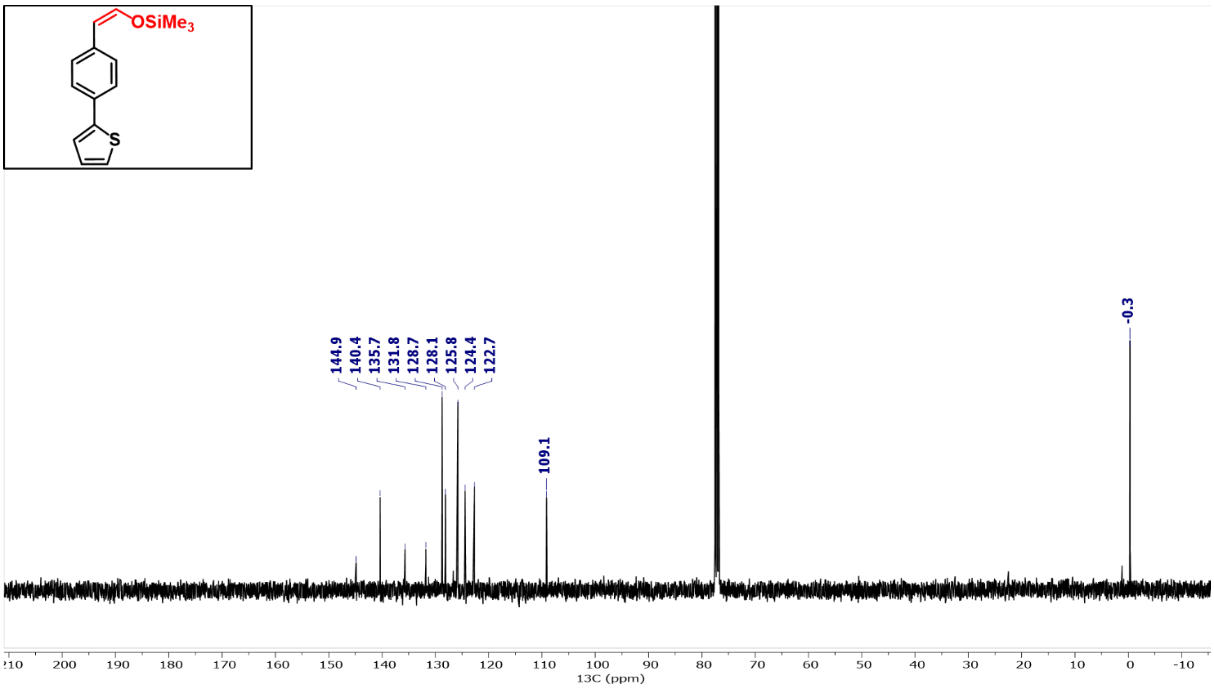

Figure S115 – ^{13}**C{^{1}H} NMR (101 MHz, CDCl$_3$, 296 K)** spectrum of compound **3z**.

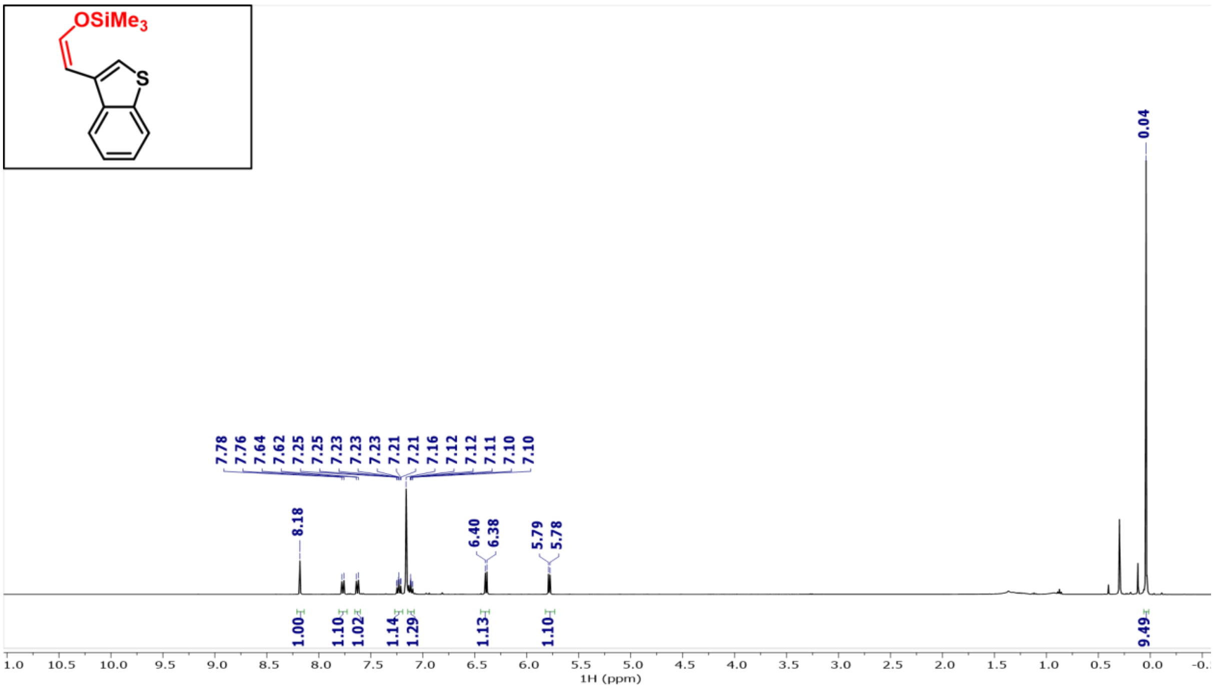

Figure S116 – **^{1}H NMR (400 MHz, C_6D_6, 296 K)** spectrum of compound **3aa**.

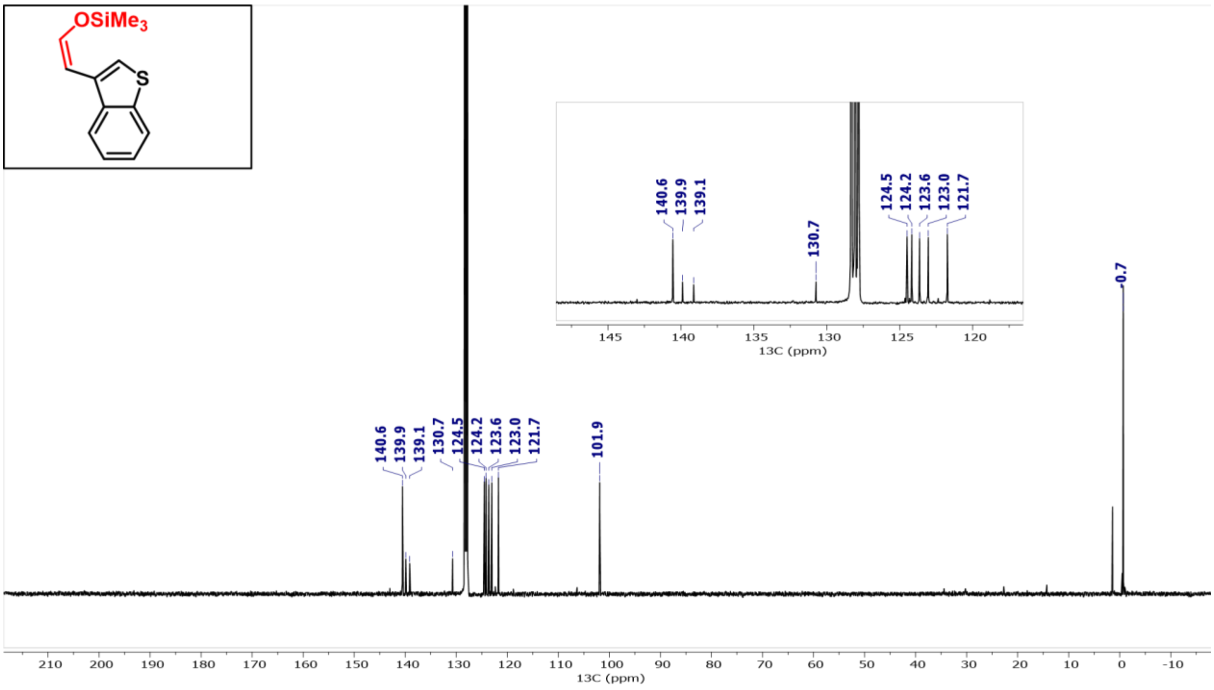

Figure S117 – **^{13}C{^{1}H} NMR (101 MHz, C_6D_6, 296 K)** spectrum of compound **3aa**.

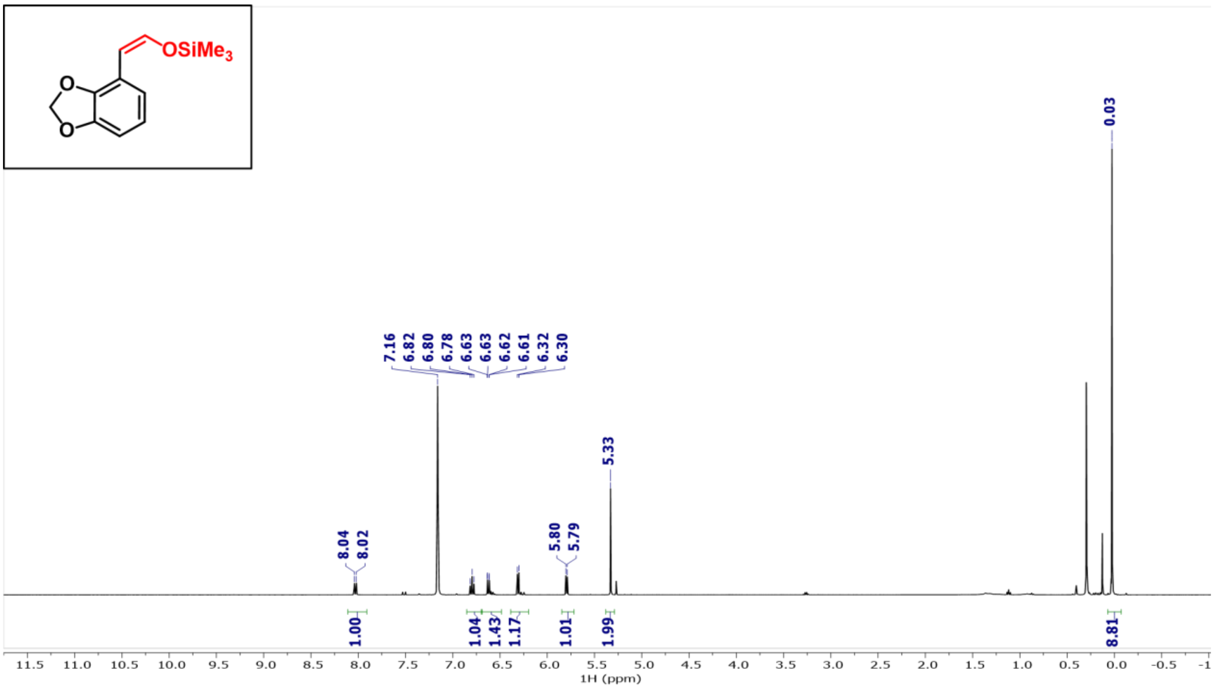

Figure S118 – [1]**H NMR (400 MHz, C_6D_6, 296 K)** spectrum of compound **3ab**.

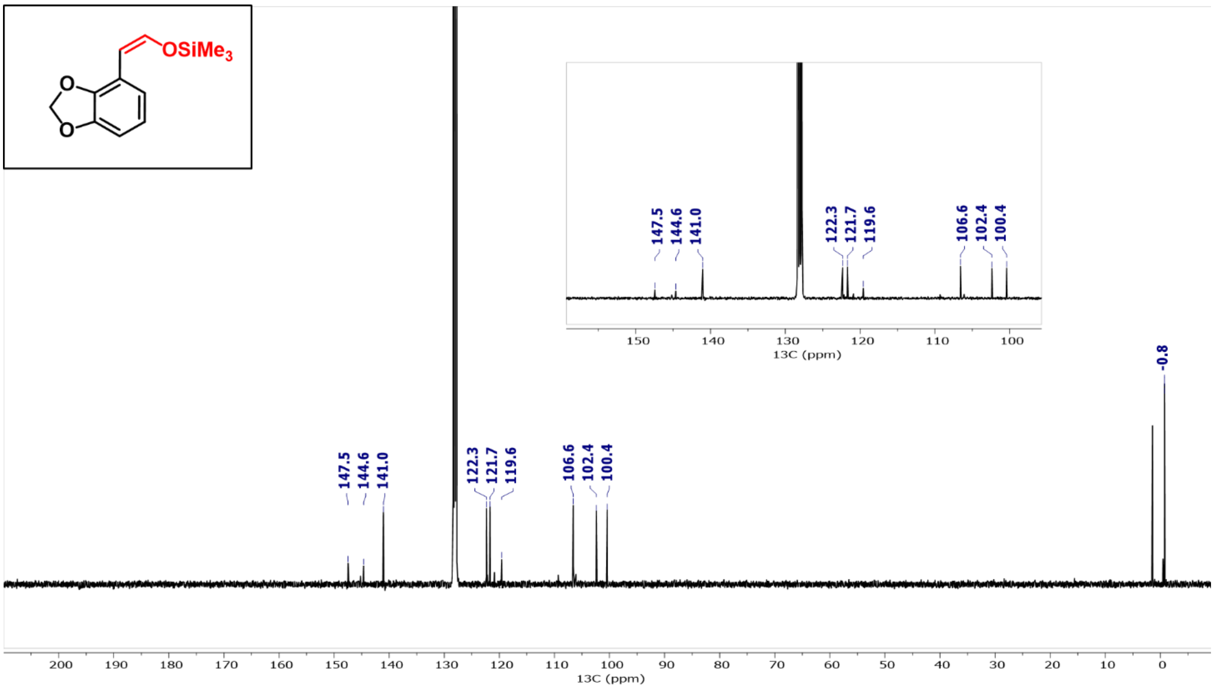

Figure S119 – ^{13}**C{**^{1}**H} NMR (101 MHz, C_6D_6, 296 K)** spectrum of compound **3ab**.

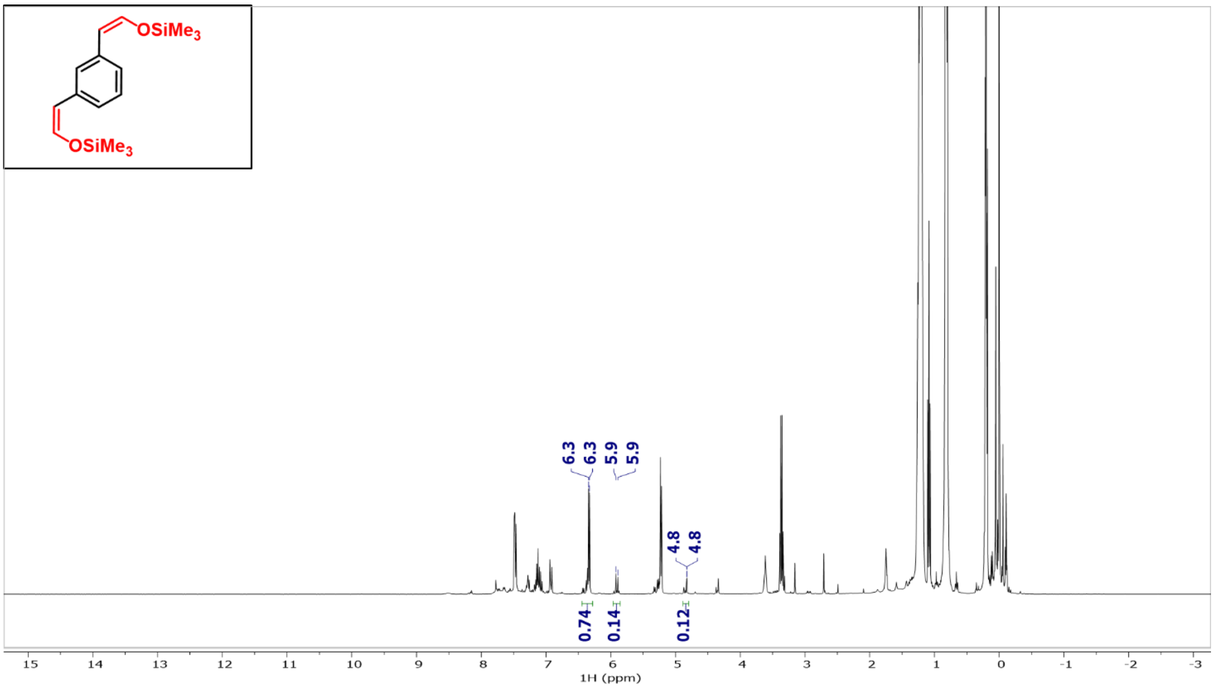

Figure S120 – [1]**H NMR (400 MHz, CDCl3, 296 K)** crude NMR spectrum of compound **3ac**.

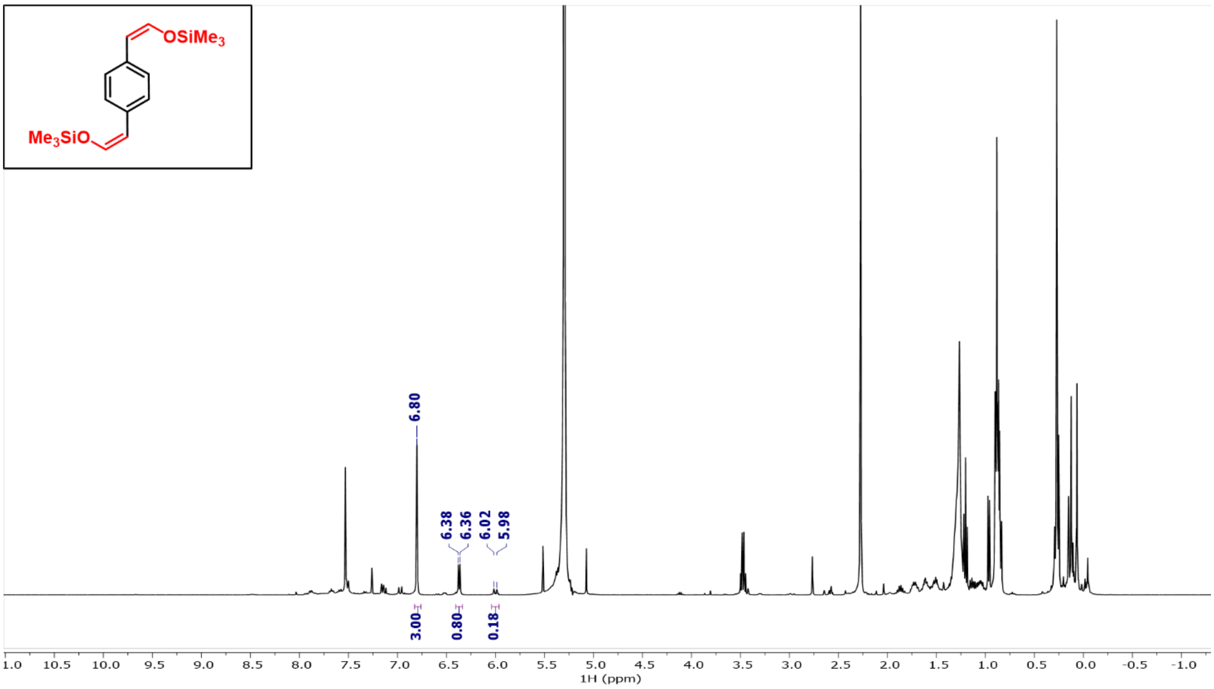

Figure S121 – [1]**H NMR (400 MHz, CDCl3, 296 K)** spectrum of compound **3ad**.

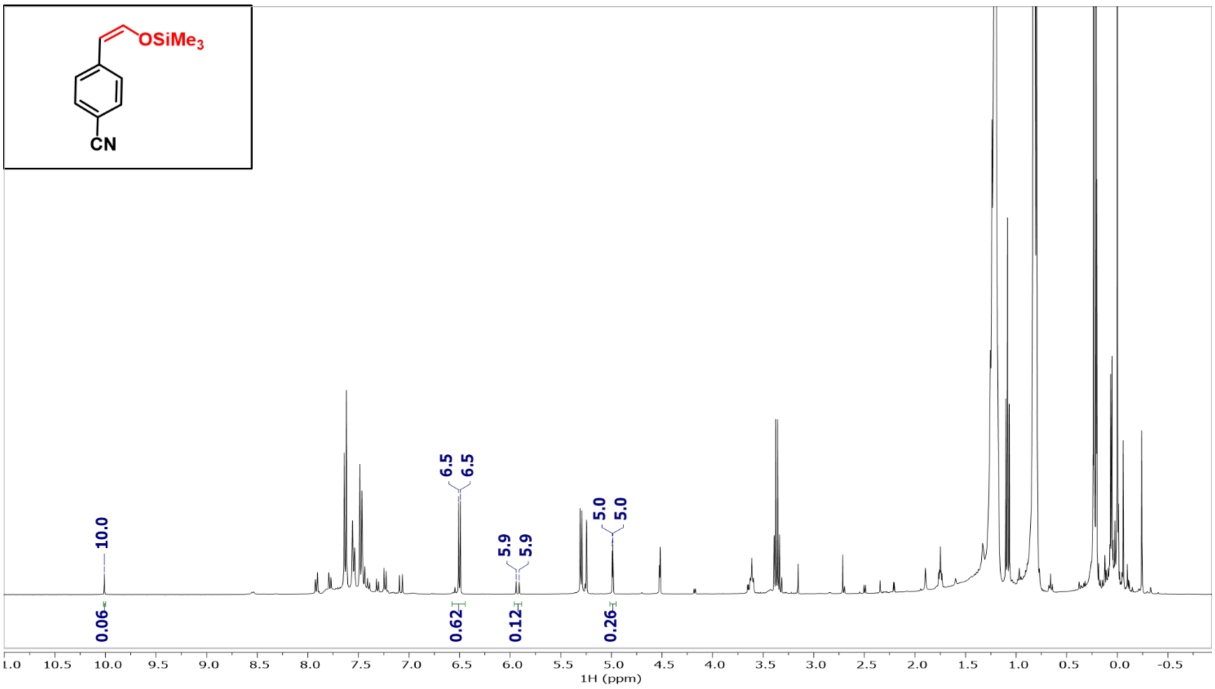

Figure S122 – [1]**H NMR (400 MHz, $CDCl_3$, 296 K)** crude spectrum of compound **3ae**.

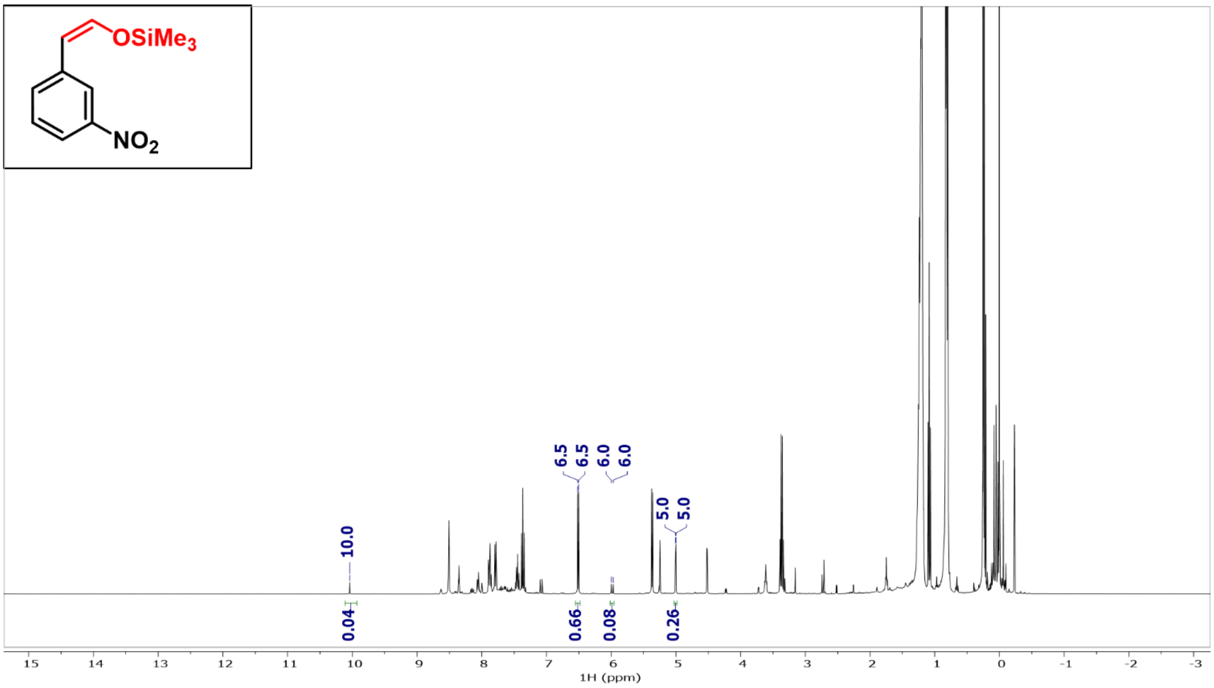

Figure S123 – [1]**H NMR (400 MHz, $CDCl_3$, 296 K)** crude spectrum of compound **3af**.

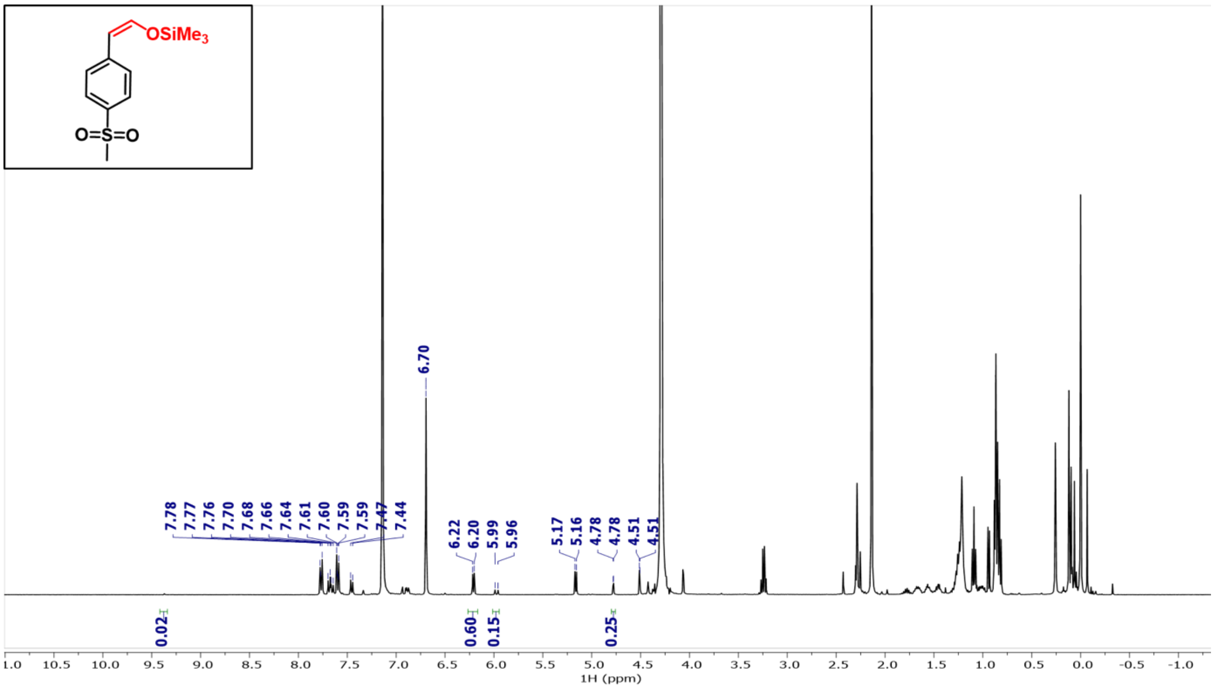

Figure S124 – **^{1}H NMR (400 MHz, $CDCl_3$, 296 K)** crude spectrum of compound **3ag**.

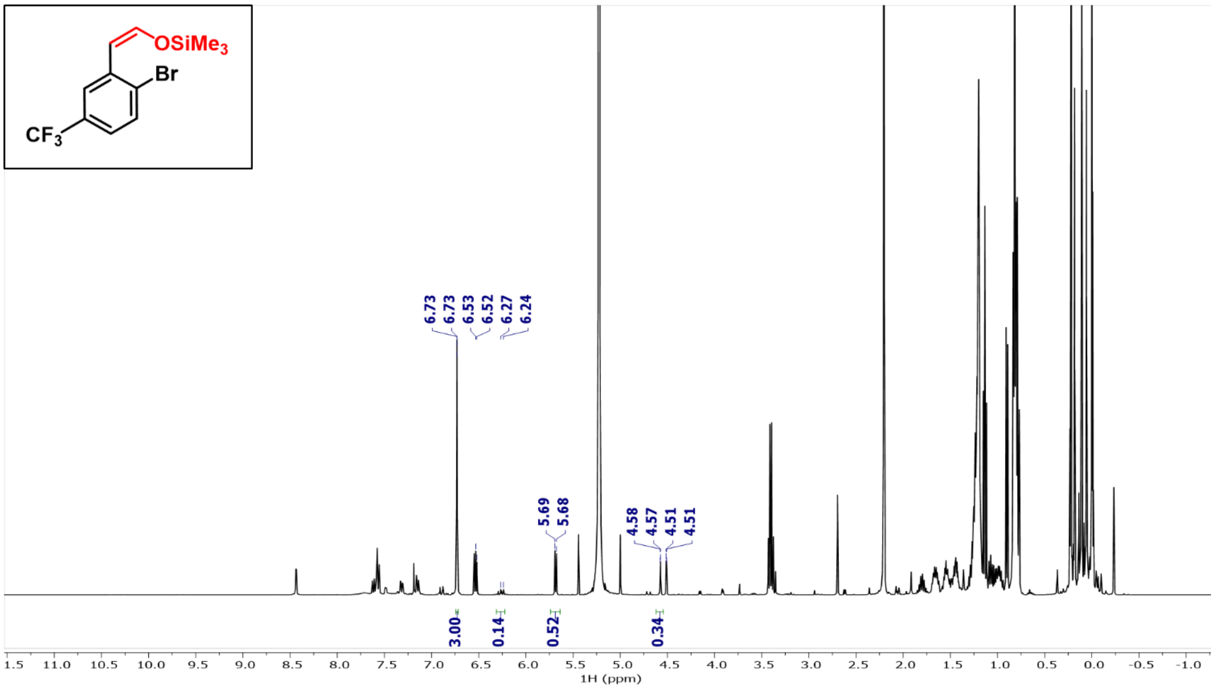

Figure S125 – **^{1}H NMR (400 MHz, $CDCl_3$, 296 K)** crude spectrum of compound **3ah**.

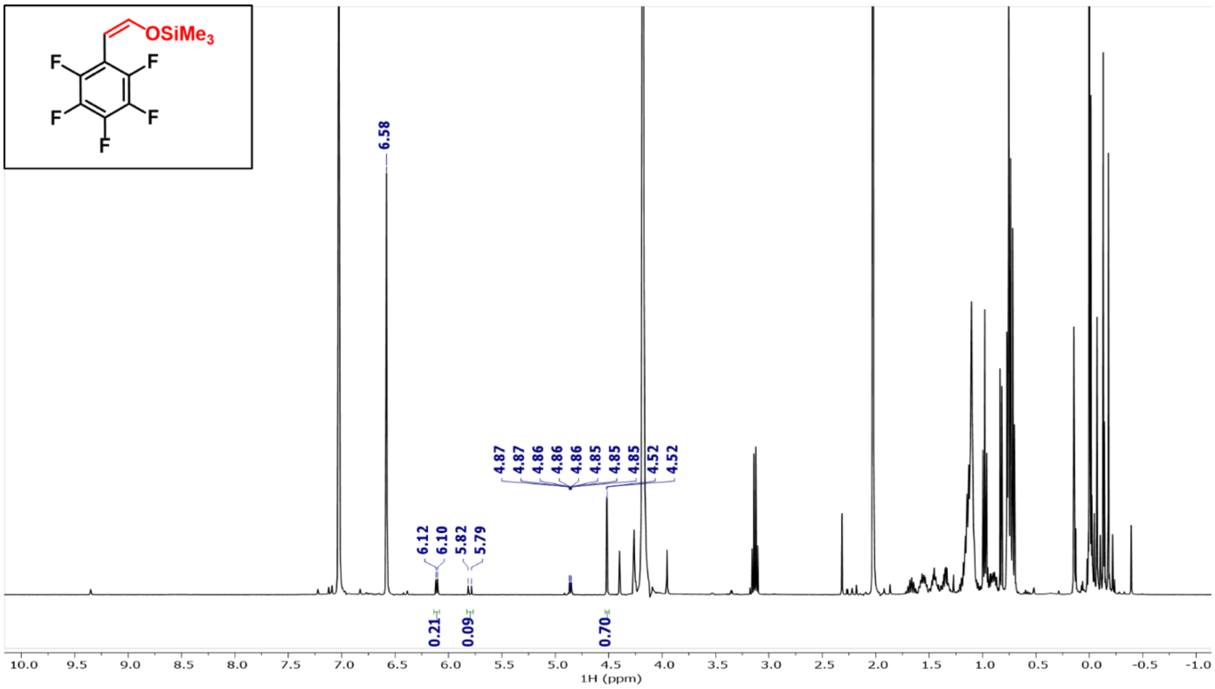

Figure S126 – **^{1}H NMR (400 MHz, $CDCl_3$, 296 K)** crude spectrum of compound **3ai**.

7. Computational study

7.1. Computational Details

7.1.1. Electronic Structure Calculations

All electronic structure calculations were performed using the ORCA 5.0 program package.[8] Geometry optimizations were conducted at the RI B3LYP/def2-svp D3BJ CPCM(CH2Cl2) level of theory,[9]and all identified minima were verified by normal mode analysis. For selected species, we conducted relaxed coordinate scans to assess potential reaction pathways. Typically, this involved scanning a specific interatomic distance while allowing other degrees of freedom to adjust.

It's crucial to underscore that these results, while indicative, only offer preliminary insights. They can hint at potential intermediates or rule out certain pathways. However, a comprehensive kinetic, spectroscopic, and computational study (also using larger basis sets and a broader variety of methods) is imperative for a definitive elucidation of the mechanism.

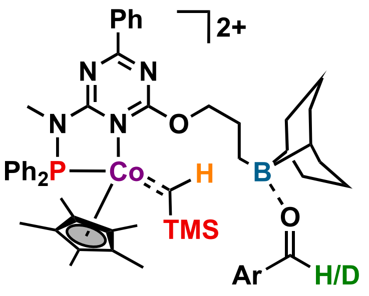

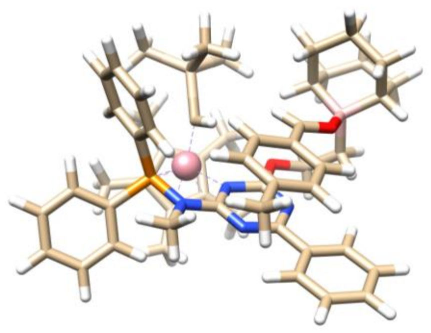

E =-4503.46602857 a.u.

G=-4502.43020826 a.u.

ΔG=0 kJ/mol

First, the carbene **ThSI1** was confirmed as a minimum structure through geometry optimization and set the stage for an initial mechanistic exploration. Subsequent discussions will reference Gibbs free energies relative to this species.

While several ether or epoxy species were identified, they either exhibited unfavorable relative energies or had notably high activation energy estimates from coordinate scans. Such observations suggest intricate underlying rearrangements that currently remain elusive and warrant further detailed investigation.

**ThSI2**	**ThSI3**	**ThSI4**
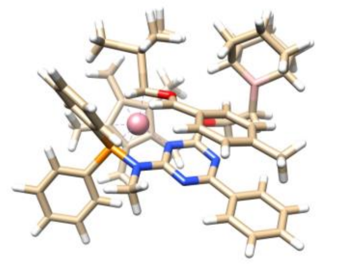	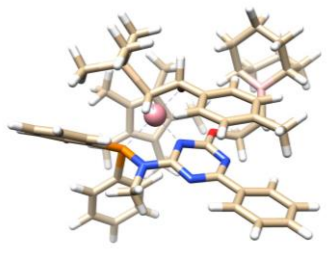	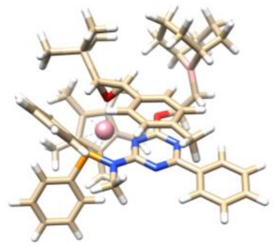
E = -4503.47164548 a.u.	E = -4503.38907887 a.u.	E = -4503.50122065 a.u.
G= -4502.43390954 a.u.	G= -4502.35877202 a.u.	G= -4502.46500492 a.u.
ΔG= -10 kJ/mol	ΔG= +188 kJ/mol	ΔG= -91 kJ/mol

A minimum structure (**ThSI5**) was determined for the product of the successive Brook rearrangement.

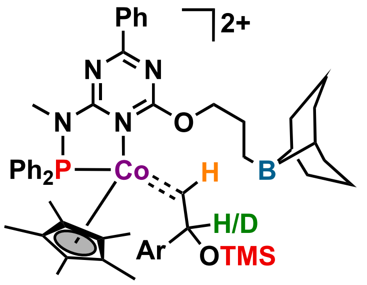	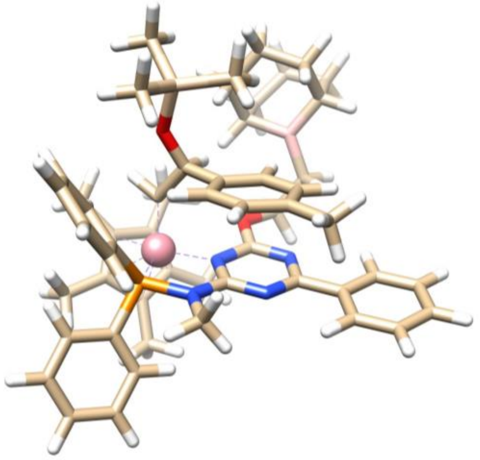
	E= -4503.50758909 a.u.
	G= -4502.47362106 a.u.
	ΔG= -114 kJ/mol

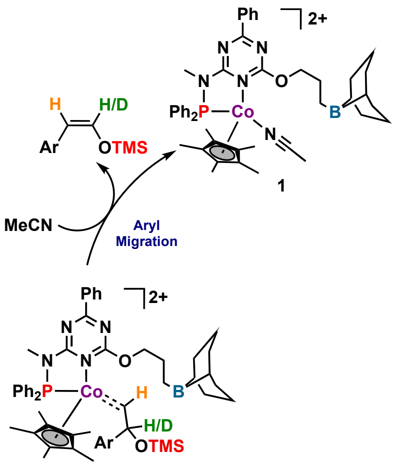

***E*-isomer:** The aryl migration was probed using a coordinate scan along the C-C distance between two carbon atoms in the configuration, specifically, the bond between the migrating aryl carbon and the recipient CH carbon for the *trans* product. The structures **ThSI6a**, **b**, and **c** represent the initial configuration, the highest energy point, and the rearrangement product, respectively. Even this preliminary scan reveals a notably exothermic pathway with an energy barrier under 10 kJ/mol, bolstering the hypothesis that a similar rearrangement concludes the reaction path.

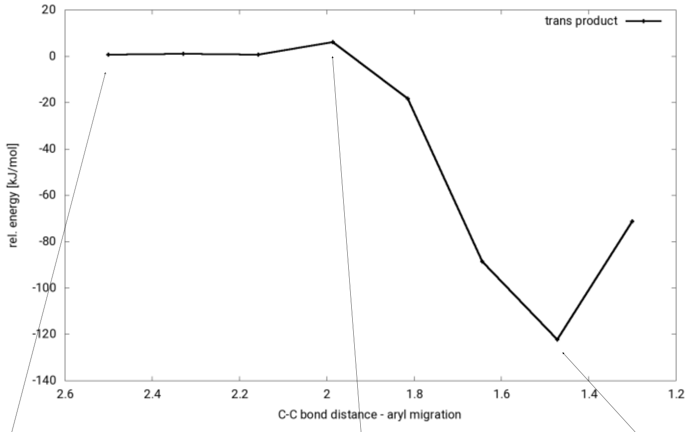

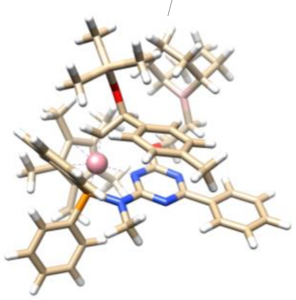	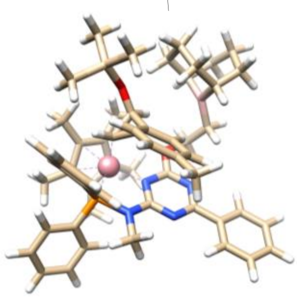	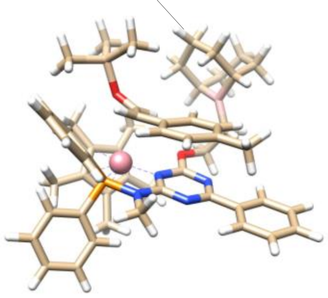
**ThSI6a**: E_{rel} = 0 kJ/mol	**ThSI6b**: E_{rel}= + 5 kJ/mol	**ThSI6c**: -123 kJ/mol

***Z*-isomer:** A comparative coordinate scan for the aryl migration process revealed reaction energetics and potential barriers closely aligned with those of the *trans* rearrangement. However, comparing the initial and final relative energies of the *trans* and *cis* educts and products exhibits that the *cis* educt is lower in energy by 9 kJ/mol and the *cis* product is lower in energy by 5 kJ/mol. While a more detailed analysis of all intra- and intermolecular interactions is required for a more complete picture, these results already hint at a strong preference for the cis product, which is in agreement with the experimental observations.

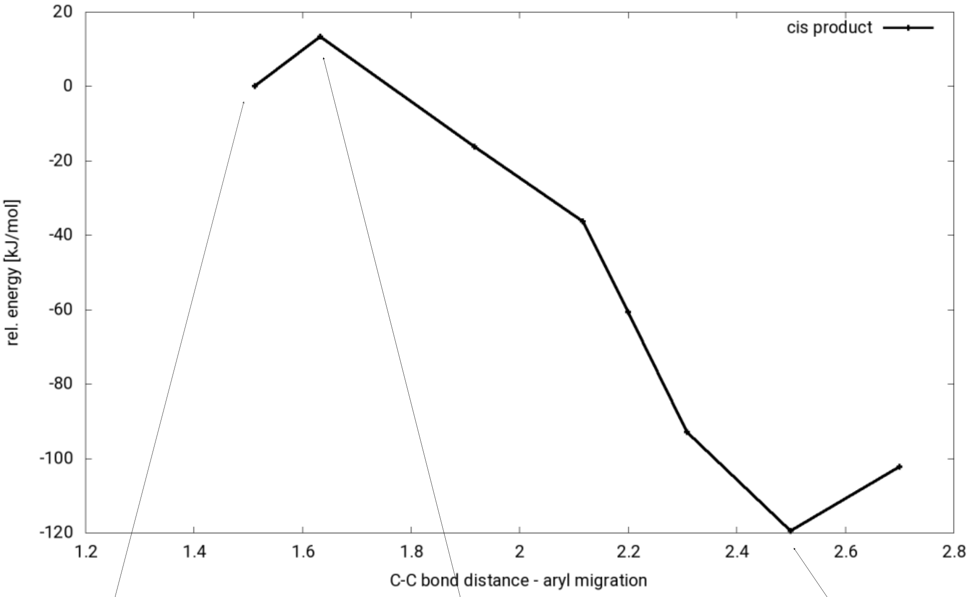

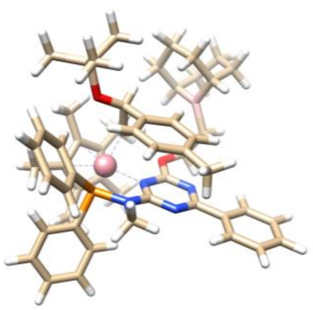	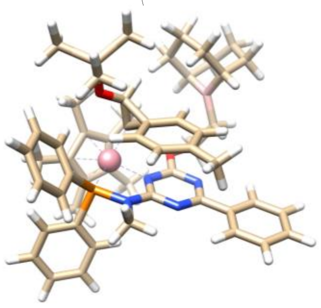	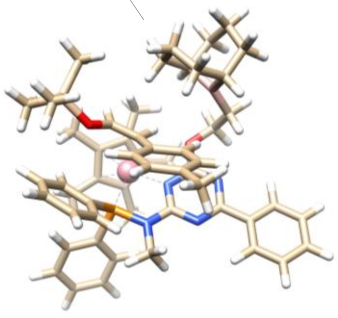
**ThSI7a**: E_{rel} = 0 kJ/mol	**ThSI7b :** E_{rel} = +13 kJ/mol	**ThSI7c**: E_{rel} = -120 kJ/mol

The adduct with the final rearranged *cis* product (**ThSI8**) has also been obtained confirming a strongly exothermic overall reaction energy starting from the carbene.

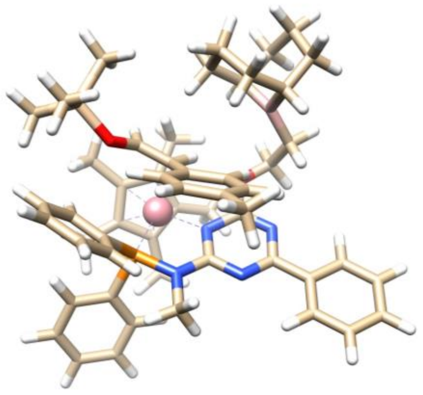

E = -4503.55335675 a.u.

G= -4502.51654233 a.u.

ΔG= -227 kJ/mol

7.1.2. Tentative Mechanistic Model for *Z*-Isomer Selectivity

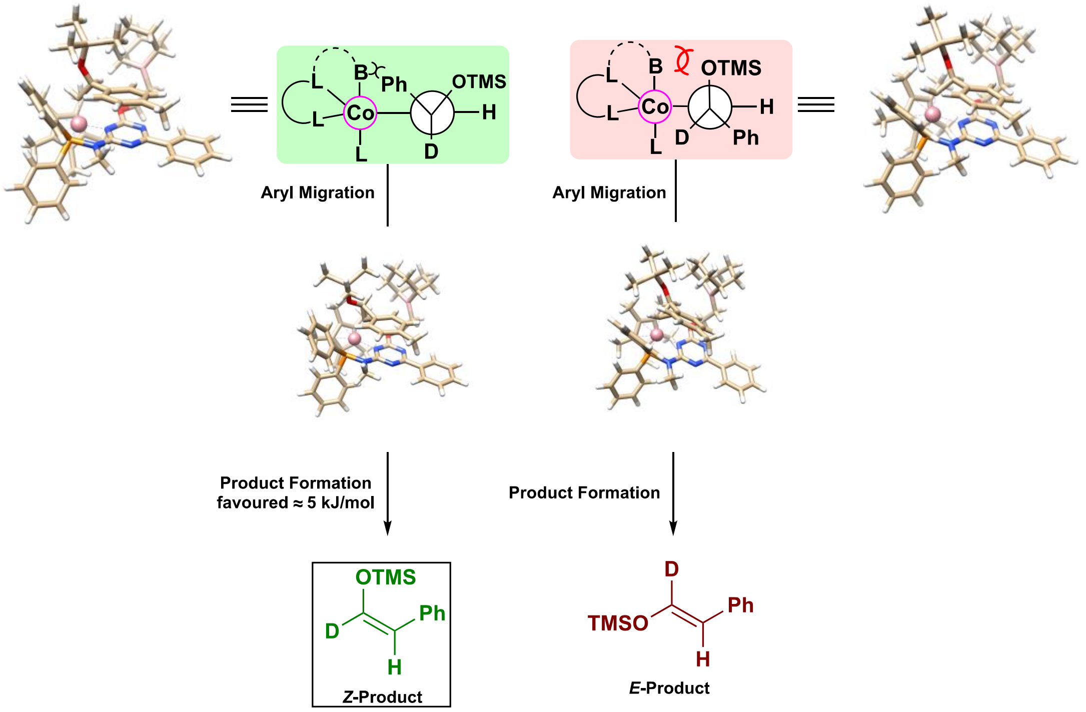

Figure S127 – Tentative model.

7.2. xyz Coordinates of Computed Structures

7.2.1. Structure ThSI1

E = -4503.46602857 a.u. G= -4502.43020826 a.u.

133

P	3.71790709257432	8.13682820461947	3.66028727575752
C	3.97269294820796	6.51333912412729	8.29552022926095
N	4.23103869782815	7.76810292142350	8.69156352949666
O	4.41478600895436	9.95991221044073	8.08322928348851
C	4.82223202668040	10.33901259185031	9.45587878338776
H	5.72401910210458	9.74743939297946	9.66934370043827
C	3.79177569831985	10.22248288252137	10.56518859084041
H	2.75941782474167	10.21697711533991	1.90711346847716
C	0.62430529215101	9.96119656019166	1.71778969665806
H	0.51306109329690	10.80728094303041	1.03696373409727
H	-1.21726846379699	7.56479043522133	3.30702534572875
H	1.02241509199541	6.95556360969757	4.15393476784509
C	4.52339413986128	7.30116752732368	2.26310113751383
C	6.17221775581744	5.70538548257374	1.47686980306333
H	6.94279403550106	4.96178304696839	1.69225399163547
H	6.34903771671141	5.50860610921580	-0.67071200855576
C	4.83183248365100	6.94576056279874	-0.11479522630372
C	4.17149821738136	7.58907707409934	0.93421022260254
H	3.97000423879525	4.79460526832724	4.66935465236851
H	2.89169286845703	5.46421418000414	3.40863550433908
H	2.28004125494461	5.21111751841406	5.07390354619642
C	3.87750676221231	5.45850409826604	9.31538604604749
C	3.61851616013705	4.12902896716304	8.93382721281046
C	3.46660958180772	3.14046348594767	9.90360061640892

C	3.56624424483456	3.46983111089436	11.26171815181428
H	3.43876912972592	2.69473724610199	12.02168783632214
H	3.90384889235612	5.04662503064091	12.70678128127370
C	3.98647423020445	5.78182195209423	10.68023695319122
H	4.17613981475718	6.81578611682328	10.96931646191981
C	6.63855727192527	10.05596893370376	5.76776030800816
C	5.71090463469217	11.33107040140371	4.07620790072517
C	6.23161839800733	10.12559654713832	3.49920417888498
C	6.77796638224934	9.32024108683576	4.55675044986533
C	6.35707012304025	9.89481633259781	2.03248644264598
H	5.42935710392747	10.13511068764194	1.49540861731781
H	6.65372465820554	8.86963319336442	1.78926002286162
H	7.16945546228522	7.27065716863802	5.10815631382347
Co	4.66288695485914	9.70594227779112	4.93276409825052
N	3.56487919621651	6.86868951049996	4.81939102587008
C	3.85482870311296	7.15555718489282	6.12198884338438
N	3.73890864357763	6.18657045475956	7.01773017218668
C	4.29718275019531	8.70154745274135	7.75516441850931
N	4.22482617456827	8.43438557348169	6.42221505768873
H	5.11544514162813	11.38449996757777	9.30570971944324
C	2.04099397796300	8.54158628895394	3.08251922932336
C	1.88916124304388	9.63133139420380	2.20847613172079
C	-0.49377934808071	9.21806837632451	2.10893067300539
H	-1.48430320894590	9.48251829779102	1.73183260203993
C	-0.34549963058507	8.14199535063808	2.99071855085507
C	0.91874698121470	7.79697193434585	3.47321168026714
C	5.52252508647931	6.34803579796798	2.52975910751470
H	5.78382315262908	6.09612215988944	3.55690405077207

C	5.83526700854257	6.01004905722152	0.15288051793900
H	4.55521056697444	7.17657575580426	-1.14590083211951
H	3.38635341865652	8.30891077596350	0.70495675488113
C	3.15098769104207	5.49910471916492	4.47175112636994
H	3.52495383784670	3.88806140630649	7.87475060275932
H	3.26356540958311	2.10985335443754	9.60332835626697
C	3.82801206366716	4.79026220508544	11.64758452134816
C	5.96815970576082	11.29782392180078	5.48877625172419
C	7.25126622965963	9.64016495019721	7.05946904316602
H	6.96307245870317	8.61920488451928	7.35084239541855
H	8.34685485085235	9.62544386035550	6.92688346457353
H	7.01884516333452	10.33248004655390	7.87379643184725
C	5.79854665408369	12.42219953986811	6.45730405926468
H	5.42254465464343	13.32360846898019	5.96120374233614
H	5.11010275157710	12.16272075487335	7.26988487170729
H	6.77353177791349	12.66378751223085	6.90994464691736
C	5.25395894477695	12.50653020234565	3.28339545930965
H	4.61008669088280	13.17787640249615	3.86022816119087
H	6.14051806155956	13.08662156558844	2.97360450425689
H	4.72542628174613	12.20294883853112	2.37236603141665
H	7.13779881737635	10.57605581751958	1.65110800689229
C	7.56661408311220	8.05588883951688	4.44699665632770
H	7.59306322803973	7.67403544708052	3.42103811372657
H	8.60552811057368	8.24927810292067	4.76247875611190
B	2.24698822850125	10.70949574573068	10.34895773860119
H	4.22230500927217	10.83667954638440	11.37552903306066
H	3.78112257229502	9.19200573791342	10.95389608564641
H	-0.37439668851646	13.23748638599999	10.73815434875439

H	-0.42102130365860	10.22100356797126	11.20345738808891
H	-1.48588785488781	12.05542692316620	10.08450135661286
C	-0.42503557579405	12.21005624763480	10.35017881176157
C	-0.03562093014659	11.22257982041709	11.46677172053063
H	-0.56511384373589	11.51378707638486	12.39378981391988
C	0.42900385496410	12.09537870740203	9.07113220947737
H	0.03857347495488	11.24687077405753	8.48265560993862
H	0.25253953330033	12.98467829848236	8.43677321687510
C	1.48127872585598	11.10022657186345	11.72289217651114
C	1.94549828796065	11.89264027293032	9.29259371374220
H	1.57096403955514	12.74910282984185	13.16581096454955
C	2.11995975807619	12.39806953983453	12.27144508449795
H	3.13715919703424	12.15683280732725	12.62673702491941
C	2.64852594890196	13.15635330830742	9.84164792752404
C	2.22017903654572	13.56401592955867	11.26502308570526
H	2.48167902813809	14.01459277341557	9.16360863218290
H	1.25590874613774	14.08896841361999	11.21280692113454
H	3.73846766441962	12.98083373881109	9.83517531641824
H	2.93435417416948	14.31200751103172	11.65250917774439
H	2.53481326318462	12.29432379869631	2.25530588387344
C	1.97932869879372	12.85557483294364	3.01437505618794
H	-0.07814576765365	10.63586739736676	4.51259513181339
H	0.93530994983583	12.94119860794839	2.67375295620082
H	2.40666026169066	13.86864722199961	3.06904898312463
C	0.23169510375989	11.46852695628685	5.16191256953999
C	3.05636960246906	10.52030773806168	5.07402255157494
H	2.38742099837388	9.84519004904988	5.63968404976958
H	-0.49211220854562	12.29072960205234	5.03418020108764

Si	1.96182403642951	12.07902865870899	4.73950547968676
H	0.18397849843476	11.13140956118271	6.20892371926301
C	2.49057170225166	13.39279455341670	5.98762962461142
H	1.62407524482182	14.06372405707446	6.11633672317553
H	3.33369610445427	13.99027074704590	5.61418114013962
H	2.75014428476155	12.98247636573003	6.96997433851580
H	1.03829809898887	6.85965758414584	10.36490710253081
O	1.54817690404773	9.30869751400282	9.85950235624463
H	0.37113976521810	4.55302371988911	9.72020617506419
C	0.75578782253283	6.63057490429648	9.33648177335686
C	0.38329519873201	5.34816488011719	8.97118754501314
H	-1.15184578944085	3.26608955853916	7.87233734364322
C	1.26020859636924	8.97782029049656	8.69880074570617
C	0.80567514735815	7.65245558793549	8.36246288254584
H	1.38163675473197	9.70521910627783	7.87980900176773
C	0.04090834558334	5.04562601274327	7.63590928298984
C	-0.31848572262658	3.63881231355965	7.25448087612105
C	0.45088655417870	7.36612671675274	7.02984252517155
C	0.06258787392267	6.07679831791421	6.67965120180140
H	-0.60180204538997	3.56204355265497	6.19552371616054
H	0.48064487507845	8.16316106124184	6.28249021030726
H	-0.22665287779711	5.85049816587155	5.65087666670011
H	0.53744176678422	2.96699442404514	7.43770140099130
H	1.62365487991561	10.31745006410815	12.49371514356286
H	2.39585073487440	11.67442055810519	8.30835026233332

7.2.2. Structure ThSI2

E = -4503.47164548 a.u. G= -4502.43390954 a.u.

133

P	4.11355428601254	8.11820455222468	3.41351865052901
C	3.77866234173642	6.57169253617101	8.09550659830646
N	4.09856418504379	7.80392864462244	8.51089347414081
O	4.37262277651516	9.99192194308570	7.94784454948071
C	4.69150695324534	10.30633674517492	9.34197755364578
H	5.51632784374585	9.64239823248904	9.63448727127407
C	3.54149301552069	10.22421320857242	10.33328415479244
H	3.51637057728929	9.93454398013912	1.24037046147856
C	1.50321291941233	9.54019734663784	0.59880435478509
H	1.52979574201981	10.31919325802494	-0.16615080757958
H	-0.59345377230856	7.22423719121931	1.96921927262309
H	1.39094058193324	6.80609759776831	3.35841822183139
C	5.26636589709641	7.17726277110400	2.34971520268175
C	7.05255230404871	5.54009566643550	2.17788001196468
H	7.74074757310973	4.84299969523671	2.66155607551592
H	7.75902988217854	5.11020094873999	0.17805651931065
C	6.16798549603562	6.57637420491865	0.17780422718033
C	5.26814816814350	7.31290528739201	0.95188232434610
H	4.12673216740581	4.83299607184125	4.62550606347320
H	3.31492453328563	5.43875378792396	3.15415033044198
H	2.39439898274202	5.25978354539278	4.68310640454455
C	3.50161584373768	5.53997342889131	9.10983842221523
C	3.09484289685069	4.25217167105065	8.71534823222748
C	2.79132899146701	3.28892925751895	9.67646968786857
C	2.89063347821571	3.60114363794722	11.03817643146450

H	2.64905668414840	2.84589097795164	11.79022507061688
H	3.36666606184196	5.12646844804139	12.49943956433527
C	3.59780586493662	5.84827696880817	10.47945158239304
H	3.90291354734551	6.85104098996492	10.77936580598279
C	6.52097467911806	10.38237986797090	5.78101446007990
C	5.52890704189637	11.63744436495479	4.11419746125258
C	6.19553951700159	10.53814852071520	3.49864593415978
C	6.78858078711897	9.73844616533528	4.53885837481996
C	6.44684722315516	10.40381481680777	2.03246288618346
H	5.57905106838789	10.70886419357136	1.43210757995640
H	6.73236648588547	9.38553883793598	1.74626239912845
H	7.56567467474055	7.79628421619348	5.07724727756592
Co	4.64983369586814	9.81686973025494	4.79502529987683
N	3.71138732181857	6.91275206459703	4.59747599624004
C	3.87443026806209	7.21829870300695	5.91819459066806
N	3.63455245982426	6.25667781776519	6.80454239901978
C	4.24527665846058	8.73874479444822	7.58429259673326
N	4.24038266073943	8.49219227093948	6.24827714150734
H	5.07011753438310	11.33316063674244	9.27309444667283
C	2.61572382634421	8.31635508713664	2.37962006205591
C	2.63160781958720	9.31281323182421	1.38840901675971
C	0.34090685447989	8.79007035141596	0.80208074037985
H	-0.54515380917441	8.97593105529686	0.19061198621221
C	0.31336087225463	7.80870351179908	1.79729403050917
C	1.44462642775501	7.56703779451715	2.58176728441083
C	6.16495865304886	6.28270344543063	2.95708497127673
H	6.17116317146964	6.15641630139880	4.04026126604773
C	7.06134336063812	5.68998868753150	0.78710810800705

H	6.16199830820293	6.69189861597602	-0.90848524283168
H	4.56978526403435	7.98600975774248	0.45533658236450
C	3.36518225637626	5.52588708811191	4.24413181504997
H	3.01438410271722	4.02311662438316	7.65240836635782
H	2.47372252437071	2.29102423882377	9.36530959861250
C	3.29405277628760	4.88148896434901	11.43732626483832
C	5.70068020157908	11.52945265201642	5.54159579157457
C	7.15817892705888	9.95058723844779	7.05886843376019
H	6.89955214609841	8.91572222004574	7.33047542734717
H	8.25219729883382	9.96741603639293	6.92059086759559
H	6.91393935916194	10.61228063834922	7.89513063184179
C	5.29655756158922	12.56072757180110	6.54699674977149
H	4.28416488978313	12.38600297791881	6.93045669665546
H	5.98206954766385	12.55908975051379	7.40562341978188
H	5.31896944449023	13.56457003100330	6.09994628267050
C	5.06846170113569	12.84638374394360	3.37131897934560
H	4.25830308357400	13.38207869475648	3.87234583297843
H	5.92268479747581	13.54279941249863	3.29895022374869
H	4.75575438368945	12.60661246167225	2.34900845336834
H	7.28082514053546	11.07354198673866	1.75951782538837
C	7.77143555545995	8.62415709742395	4.38391916394207
H	7.79558578625854	8.22740740667011	3.36389190055463
H	8.78117607406114	9.00235527174319	4.62123221392432
B	2.27021075056199	11.14065701543848	10.19461767735758
H	3.98298084121617	10.45945049126807	11.32206967979488
H	3.20435164101468	9.17921421195583	10.44761746636982
H	-0.64334265089984	13.24453160805831	10.16063570122514
H	-0.41763823807770	10.25012362608388	10.71428090951063

H	-1.54336479864779	11.93263109653641	9.43157246128455
C	-0.54296221456329	12.20913733027459	9.80637233469126
C	-0.18060661540180	11.29423872701925	10.98745744258435
H	-0.83186305815422	11.53599044327796	11.84700004888268
C	0.45371669392582	12.15191909906969	8.63491782130390
H	0.24855864913008	11.23967446804883	8.05009745402793
H	0.25436275811844	12.99393580702315	7.94892217881259
C	1.30168538700165	11.34361493549440	11.41580384545963
C	1.94996803922168	12.12679203011042	9.01817290808779
H	1.10573847168378	13.01673748439726	12.82066018589173
C	1.76714955451129	12.72336828326473	11.98554116130390
H	2.76936531841899	12.58318141426169	12.42901315884505
C	2.48376010692024	13.46936241078660	9.61661618551744
C	1.84283430592615	13.87071956453432	10.95788748230135
H	2.33939931382682	14.27672982415494	8.87634076662325
H	0.83906644171841	14.27782649063650	10.77586687904234
H	3.57716521426509	13.37851340314293	9.75197521002188
H	2.42252551004352	14.70141708392777	11.39491353709782
H	2.75303043340423	12.11808544156278	2.15688962893271
C	1.84633667318692	12.50667094759337	2.63579641619692
H	-0.37646732029517	10.81257049420563	4.26947545726577
H	0.98323104750600	12.04402249985615	2.13229985038679
H	1.80494703644422	13.59385886174614	2.45908786927119
C	-0.06224541265073	11.70312423013140	4.83980126800733
C	2.68253914760223	10.47279871669786	4.78986214698846
H	2.22604754871374	9.91083154347259	3.96184110922342
H	-0.69516299512530	12.54511566720779	4.51209307200613
Si	1.74711171615879	12.13738176630165	4.48835286717504

H	-0.26467156053185	11.52726097399423	5.90635807679881
C	2.16204706257575	13.63981033678644	5.54366601043983
H	1.36203756635048	14.38064655664829	5.37452999860525
H	3.11851711399420	14.12287170481822	5.30612949057137
H	2.16128456341324	13.38715906067536	6.61218066263913
H	1.63344508039644	9.44359857033336	8.51400404575767
O	2.02149019692750	9.89229416387333	5.99343613652049
H	0.94421673564694	7.97900570615643	10.40377608843520
C	1.06384074316084	8.53521864670309	8.33489395464327
C	0.67931696305309	7.71602943054878	9.37752863261371
H	-0.73784731893793	6.18853206498586	11.14341967367475
C	1.20864328766005	8.92330327244568	5.88467378396391
C	0.73474594550901	8.18826037803047	7.00196172734849
H	0.91817540849152	8.60553558035072	4.87401085019117
C	-0.03660769398227	6.52094844544687	9.13390832291837
C	-0.38719445922401	5.61395657644189	10.27219970591680
C	-0.00781239981900	7.00855272505212	6.75254309515699
C	-0.38043995242521	6.18968864436359	7.80831482787231
H	-1.15136415056875	4.87760489845193	9.98803028686272
H	-0.26114688675508	6.73955422813827	5.72408717139725
H	-0.93949823676285	5.27199847853874	7.61363466168585
H	0.51500151465718	5.06444718540517	10.59260939051150
H	1.45970987537965	10.59695054644565	12.21356063459867
H	2.53759821052522	11.91748006950968	8.11180365794111

7.2.3. Structure ThSI3

E = -4503.38907887 a.u. G= -4502.35877202 a.u.

133

Coordinates from ORCA-job Co_carbene_S6_3_3 E -4503.389078865940

P	3.86482261294109	8.37472582946896	3.20628489661431
C	3.20177173785508	6.07084581306944	7.44304297290331
N	3.71701377395434	7.16743211691543	8.01686856453129
O	4.46462663293870	9.30901083243046	7.73421517101054
C	4.98581679036092	9.27494757119196	9.10333744515860
H	5.53554255013630	8.33094031969678	9.21489190725459
C	3.92702646465264	9.44798141454543	10.17871549088613
H	4.46075524165198	9.55328667430106	0.57931628879700
C	2.82051591697987	9.02314579367659	-0.70333271418648
H	3.25193559123402	9.53328490524930	-1.56734574482701
H	0.07233488401605	7.22601909033890	0.22690665102791
H	1.26580359765997	7.24117289026920	2.38352836500531
C	5.56477436474062	7.78340458905659	2.87822027998659
C	7.68228214694870	7.13422367103580	3.87764958490021
H	8.32338068142674	7.04861564381787	4.75713098155031
H	9.14255461196643	6.28032681698230	2.52893911249543
C	7.30684001760944	6.79875603831591	1.50913253345030
C	6.01682919664775	7.32454278118194	1.63252704105758
H	3.99476451779618	5.00804701559809	3.81627174532668
H	3.31199562016983	5.74356942220485	2.33518524255811
H	2.22063904903440	5.15316696538950	3.61962425789677
C	2.83496517777624	4.94299136953181	8.31166380516786
C	2.15392970949283	3.83173278859857	7.78155388630371
C	1.78366781520082	2.77676142409418	8.61393842089142

C	2.09985457802154	2.81535996416799	9.97796049187566
H	1.81227074274140	1.98536993547979	10.62815340613077
H	3.02586043039217	3.94729060952039	11.57489033926434
C	3.14403092870860	4.97925756736358	9.68430409529272
H	3.66636328259507	5.84616107107398	10.08961783566479
C	6.24690072732801	11.56142777344604	5.94570343523974
C	5.17342464948393	11.75456899003065	3.89819539491446
C	6.47641450667121	10.96182540371309	3.80559873579337
C	7.03355747315367	10.78955983432862	5.08396431711716
C	7.10762310821837	10.66408155956069	2.52118962592450
H	6.37045344770635	10.38600211543204	1.75593398736262
H	7.91205961904166	9.92274884160680	2.58796578366431
H	8.15332245615847	9.32233843138368	6.19105877885588
Co	3.34138299612174	9.83287736886411	4.90256418650157
N	3.21862587217401	6.96848977476477	4.03767741567661
C	3.24461006519152	7.03847051476970	5.39448802193405
N	3.03129761354502	5.95118317814899	6.11862483218401
C	3.89291966617906	8.23212817976166	7.25678156643763
N	3.55359319484050	8.25588904437464	5.93904534010268
H	5.70232087762665	10.10293914073823	9.11439752743548
C	2.96513642054057	8.37220475964038	1.63173791232788
C	3.50434810040749	9.03272098577418	0.51336073058343
C	1.59240330659408	8.36368722371239	-0.81465246305556
H	1.06248086777132	8.35196122277159	-1.76988713824297
C	1.03859960234872	7.72966082151886	0.30190652374835
C	1.71533037939774	7.73750559811517	1.52318008595930
C	6.40654004896273	7.68523288506344	4.00283568797346
H	6.06572725383587	8.02747539536684	4.98165058184112

C	8.13925385463924	6.70126358328955	2.62795273832933
H	7.65517055237149	6.45212989780671	0.53343660958728
H	5.37050844107979	7.36287354539286	0.75581925105486
C	3.18584084902060	5.63631236243079	3.41735624589451
H	1.91338962572262	3.81372929462316	6.71810193238506
H	1.24789144036870	1.91933725881036	8.20019078208294
C	2.78153566365420	3.91670740818525	10.51061614044033
C	5.05802419818658	12.16007089269099	5.19059178942733
C	6.59427806076238	11.94030347045972	7.31127061977416
H	7.31320935942078	11.25566579364740	7.77773468589512
H	7.07125892284795	12.94304005690891	7.25481457465885
H	5.70180795901016	12.08569998723259	7.93496408150171
C	4.13553191572775	13.13828598851196	5.81184344628535
H	3.36869530454023	12.60165270000380	6.40025369450718
H	4.67849644927676	13.81138066049999	6.49253564052489
H	3.61952593548567	13.74041816707417	5.05415006821182
C	4.48315575253098	12.27634225557871	2.68368366875613
H	3.57091287759167	12.82533582532823	2.94322212699089
H	5.15516253870216	12.96890285420067	2.14703258555083
H	4.21966860865603	11.47965592624339	1.97554751861155
H	7.54927799987593	11.62093649309755	2.16587249713105
C	8.32139360470557	10.11989953249982	5.45007708467393
H	8.80358748235276	9.67081087030956	4.57381275481374
H	9.02561089636501	10.84215773933669	5.89282527777623
B	3.10067564367783	10.78829912990037	10.26601445499424
H	4.45559012689263	9.36182834002533	11.14939302380255
H	3.23642183843518	8.58934836525454	10.17757217527310
H	1.34094344234706	13.89730088060962	10.78042709807776

H	0.22250051113243	11.06707172186983	10.58122735536521
H	0.06528841636960	13.29898285536034	9.74358200358345
C	1.04490966297837	13.02942187614623	10.17433817264465
C	0.86574334751617	11.80519374307073	11.09119876085346
H	0.30817419346995	12.11296960262913	11.99441891383166
C	2.05331196078855	12.82995320426681	9.02704658293128
H	1.56664693527474	12.27027812020687	8.21109910383204
H	2.30924708959012	13.81469176122770	8.59449433188940
C	2.17268620283939	11.08999879698286	11.49949850537238
C	3.34518722625462	12.07093839382719	9.39454908857679
H	2.55409047463387	12.27917408196375	13.30246945820224
C	3.11387954568025	11.94696185255422	12.40971546236349
H	3.91715625509878	11.28693180863188	12.78308861555856
C	4.31756719707971	12.87184596766331	10.31911072272480
C	3.75985398009158	13.16728929559578	11.72359844615588
H	4.59619447849031	13.81836491502607	9.82093316775996
H	3.03427723120025	13.98958667754729	11.66329665411313
H	5.25652843869149	12.29700462261270	10.42187433016684
H	4.57780886494613	13.54274866753886	12.36179637172093
H	2.14228520609081	11.37616467082865	1.48225516797438
C	1.07692371999778	11.16084380565514	1.62773429340786
H	-1.32128425217918	9.45136302895368	3.05914004100735
H	0.82423542799169	10.25796105317916	1.05875438784179
H	0.50322283861670	12.00126450833277	1.20076488663808
C	-1.21468325066565	10.44778230139220	3.51854986580437
C	1.42706954826015	9.71645267708880	4.55588492546015
H	-1.85947596924248	11.15296360033953	2.96717584243948
Si	0.58396698921410	11.02866448199826	3.43273360079285

H	-1.57419240592272	10.38876651929038	4.55825216161535
C	0.73388215736793	12.75662386331647	4.17466625076275
H	0.03372181237834	13.41651818419531	3.63516636930529
H	1.74269151729862	13.17286873607646	4.04456614952000
H	0.48568852278621	12.80313391112383	5.24497559392008
H	1.38936275664134	10.19379845885837	8.67896692301597
O	2.48016401265660	10.70036415714982	6.25120908276991
H	0.67940762943160	8.51593673317693	10.36593900136242
C	0.95595643790662	9.24701436737350	8.35854325873954
C	0.55404583036757	8.30240707376709	9.30100982356639
H	-0.71341981012797	6.50865280386913	10.87145608043591
C	1.20275400360857	10.10760096478474	6.00456611060227
C	0.81086991166664	9.01347803284527	6.98470902903366
C	-0.01173555960997	7.07933182221587	8.90406159771983
C	-0.42341851561528	6.04356670380361	9.91697093751828
C	0.23512089539088	7.80109601706491	6.58470960511415
C	-0.16256722178521	6.84968000476406	7.52924283523389
H	-1.26667650536369	5.43752104679190	9.55242532317667
H	0.08696579077539	7.58225379386010	5.52571412783495
H	-0.59956914255606	5.90761813573063	7.18708102325310
H	0.40923091276967	5.35224924054561	10.12765303306316
H	1.90953810613073	10.18455826487249	12.07365956607567
H	3.87363927955303	11.84742206748475	8.45779142456761
H	1.14593826292276	8.69484150258881	4.27719748267319
H	0.43902608432052	10.90138838265001	6.12624925522414

7.2.4. Structure ThSI4

E = -4503.50122065 a.u. G= -4502.46500492 a.u.

133

P	3.77606626250424	7.77780820571808	3.69757854843420
C	4.03691639492857	6.06931300510010	8.31367469896515
N	4.36472043097009	7.29728654174480	8.73466089164067
O	4.55031060111278	9.50727994191908	8.21834645221272
C	4.96084437342158	9.75498557199857	9.60658661154350
H	5.71512136963398	8.99858980248799	9.85497894895110
C	3.80339308155549	9.75661145848750	10.58836559454204
H	2.86490263974261	10.01816385134706	2.13294390854100
C	0.93335110218150	9.50899936808148	1.34179333586830
H	0.85815522659304	10.43182655292000	0.76454593971523
H	-0.81723194969903	6.67354593008499	2.07375791314567
H	1.20953000057727	6.21262780230080	3.38483776221603
C	4.78690927215499	6.93428572274841	2.43818953492702
C	6.61060246763822	5.42496693781141	1.90795213464906
H	7.40475966173381	4.75167041517713	2.23860746041982
H	7.01447779885365	5.13096653785762	-0.19720328289491
C	5.35860550772854	6.48476165089418	0.12515345749292
C	4.55351483726277	7.12896998030583	1.06727174804762
H	3.98402600633308	4.44428411939460	4.84742665598510
H	3.11430998792212	5.05815081295601	3.41621056035249
H	2.25017460830488	4.85909427680889	4.97765858342249
C	3.94460850006993	4.98743950387801	9.30680814427105
C	3.70328176390140	3.66444687959923	8.89128806086788
C	3.58791227829753	2.64507185325951	9.83490178290016

C	3.70665707929038	2.93688819257512	11.19970534660982
H	3.61019615737469	2.13733306447929	11.93855395159594
H	4.03590094693867	4.47777240634856	12.68453204466064
C	4.06844267425522	5.27333534050596	10.67918832993617
H	4.24769501931930	6.30156179054402	10.99476972314402
C	6.39898430439249	10.16858883897788	5.87697384430754
C	5.17155578602214	11.19663085715954	4.20574232015611
C	5.90011611854945	10.13739003616236	3.60130194507072
C	6.57356389013837	9.42071388853945	4.65854676818248
C	6.08572822788027	9.92306357279279	2.13754555958812
H	5.16459232805407	10.07645440040542	1.56075662373329
H	6.48373010428216	8.92948409288809	1.90759858495759
H	7.38454877534429	7.52351296738281	5.27054872139120
Co	4.52732508861259	9.39860710319319	5.05982016213795
N	3.55779066029394	6.51682857601380	4.86222452402224
C	3.85334253584405	6.78508000765059	6.16677911535627
N	3.74050579350198	5.79305273859129	7.03990583983051
C	4.39256007272704	8.26115345169510	7.83110249919141
N	4.24700802128540	8.05261629670977	6.49688361022633
H	5.43923030344808	10.73983122160797	9.55712651736887
C	2.19587064267512	8.07710743898686	2.84356962681307
C	2.07467550895321	9.26824865428155	2.10897122369235
C	-0.11037530840416	8.57983092282830	1.32858561584512
H	-1.00889807196611	8.77387637603897	0.73820195617947
C	-0.00274052843774	7.40138596105321	2.07446339281323
C	1.14928393590520	7.14018745501823	2.81930038674097
C	5.81558897265103	6.07138111061810	2.85458783549298
H	5.99167541173849	5.89228547442682	3.91608930672187

C	6.38885168835968	5.63646861968970	0.54231930380229
H	5.17379817614633	6.64329789192839	-0.93970728750147
H	3.74789230720530	7.78079067496729	0.72824257626389
C	3.20242513378545	5.13426865143863	4.50431503762199
H	3.60551776647306	3.45194242251362	7.82629754378722
H	3.40164023261116	1.61926399501004	9.50858718669960
C	3.94668591745772	4.25103058629556	11.61958876054101
C	5.49257918728071	11.22483612671756	5.62319939204369
C	7.18101358920789	9.88234938615192	7.11065611020282
H	7.07625366285139	8.83933952613778	7.44239594711019
H	8.24812056470462	10.02615456067971	6.86902063303779
H	6.92576911552247	10.55519766450066	7.93374206751903
C	4.97792667210364	12.24612056977545	6.57681713033501
H	5.23161039801776	13.25681250731144	6.21874556130097
H	3.88258301496494	12.18767356246499	6.64195146645781
H	5.39285180384685	12.11670962726651	7.58266157470733
C	4.35796627280162	12.22424648401721	3.50040584266275
H	3.48361097160750	12.51298546227390	4.09228012087626
H	4.96864653588989	13.13229440492779	3.35746783069930
H	4.02478615209864	11.88594603083800	2.51149292501276
H	6.81987214513107	10.66917190405415	1.78713090462450
C	7.55778262410800	8.30826960728051	4.51959077282663
H	7.53537413577959	7.85390015669864	3.52395451135850
H	8.57333604292889	8.70540708843586	4.68857159786248
B	2.82489150636258	10.98927484420791	10.61943805599359
H	4.27134938758438	9.85183138886868	11.59422754490453
H	3.30486582475156	8.77675192315381	10.60401281470803
H	0.56588298906056	13.77636561140852	10.46936221385351

H	0.25146183873523	10.98148023329689	9.29571816002934
H	0.22366036483684	13.30255489024083	8.81919006535965
C	0.81654945933614	13.02470652567339	9.70814755215002
C	0.37450262264201	11.62800908784325	10.18190317119995
H	-0.62823464848941	11.69712407131938	10.64111784787542
C	2.30984339956845	13.12756527090780	9.35869129310411
H	2.48233799319900	12.63232162801776	8.38781999984713
H	2.57812494407987	14.18822775595513	9.20230437265907
C	1.34663966389372	10.91392616371754	11.15555474536575
C	3.27674623305075	12.47602998651016	10.37365501472579
H	0.41064841814432	11.56660321467642	13.02450629180482
C	1.41865405363037	11.56122439352678	12.57179841944568
H	2.03546105368437	10.90677365507696	13.21458987758945
C	3.29650770294975	13.14446861591858	11.77974790188034
C	2.00595629100280	12.98298899740366	12.60393078862020
H	3.53558616243787	14.21817222160981	11.67306135394358
H	1.25009827879995	13.70255153139345	12.25977113121998
H	4.13516232101996	12.70257826623336	12.34809358653332
H	2.21625261699460	13.25826178347468	13.65152549461322
H	-0.10142177177016	11.95035435372502	2.38034184261074
C	0.57343137071897	12.63966238469100	2.91005120006595
H	-1.66752683454872	11.20783564827174	4.90567236419739
H	0.20070693012133	13.66610221218181	2.75433340449311
H	1.57103294084213	12.56766593063765	2.45094933287802
C	-1.16011897300314	12.05244798468968	5.40013892818829
C	1.32319560241496	10.52731358546880	5.09216728267213
H	0.93243345373518	9.75315867961080	4.42439209953340
H	-1.75166662741138	12.96388803968796	5.21264025063266

Si	0.59567139097998	12.26595087501865	4.75148632583103
H	-1.15316267060244	11.86333574483560	6.48637486492904
C	1.46967993255023	13.62515063517925	5.70773033657041
H	0.87545233802365	14.54874111195722	5.60436305869049
H	2.48264489408948	13.83874378969863	5.33753661728551
H	1.53606857818411	13.38797003076266	6.77806193289797
H	1.75147402920327	9.19849171315756	8.99091280918929
O	2.69832209875049	10.22734814224588	5.50382599725890
H	1.10496651462535	7.01592613624133	9.93350712309407
C	1.34462227986318	8.43419840840291	8.33283602567384
C	0.97358510509134	7.20016187968481	8.86412309681158
H	-0.34126039063033	4.92713769687689	9.61348650169072
C	1.60155459653265	10.05275108260863	6.46263227844295
C	1.20917582078350	8.70745017277112	6.96411911802257
H	1.67695575686044	10.80803739627761	7.25160779175407
C	0.44741317061847	6.19052391442492	8.04291432317415
C	0.09464529277993	4.84055501005509	8.60640209666596
C	0.64509456635944	7.71522273479805	6.14821560382412
C	0.28112976244047	6.47797511197843	6.67770078485330
H	-0.61487186600162	4.30532109420504	7.95840639528173
H	0.47283672482488	7.90568434741676	5.09105198682041
H	-0.15162541420015	5.72105628474090	6.01802720304178
H	1.00000624015552	4.21661154289187	8.69892040642484
H	0.99265998435264	9.87674305778067	11.28833241133755
H	4.29625591071280	12.56150373485544	9.96128017589853

7.2.5. Structure ThSI5

E= -4503.50758909 a.u. G= -4502.47362106 a.u.

133

P	4.31594845098219	7.69585892167237	3.83926155669077
C	4.56638124928942	6.21324134105426	8.53559724245689
N	4.79505999597428	7.48128529695888	8.90407613175486
O	4.84273477137409	9.67072304532265	8.26494572285054
C	5.12655600086666	10.04022044146267	9.65383088847831
H	5.95030579538433	9.39879241453782	9.99315632810420
C	3.92301030956925	9.95930734487834	10.57645868917593
H	2.85295620972643	10.04848171777716	2.97719125384462
C	1.07639921195104	9.36895971029002	1.97852170595577
H	0.72438655254270	10.38291316814026	1.77869230009485
H	0.24627925349648	6.10290659937604	1.44542016503294
H	2.36764090234595	5.75842564255569	2.64272058028514
C	5.39531880674544	6.88190496394464	2.62539086087142
C	7.29120667286659	5.45020131660326	2.14183242065203
H	8.10963910595149	4.81693531500158	2.49142289304974
H	7.73540644898438	5.14594670779923	0.04655123969288
C	6.02044794111905	6.43342261331478	0.32620754479905
C	5.17580913985370	7.05442729961345	1.24837413453198
H	4.58584494802689	4.40391113042450	5.25500518941185
H	3.96071423975635	4.89943029466175	3.66365203645108
H	2.85448770511206	4.81928973726415	5.07672027167308
C	4.49316472527723	5.18178159945648	9.58253955718065
C	4.28255101097923	3.83401232430205	9.23503723408025
C	4.18196330401402	2.86285604958055	10.22996071261315

C	4.28713090854373	3.22722336054197	11.57830385865443
H	4.20351189922935	2.46494427095672	12.35706135912172
H	4.57726632604082	4.84923355534513	12.98271181780676
C	4.60166149789911	5.54091350353722	10.93908326353828
H	4.75811423307545	6.58767933984059	11.20187229248323
C	6.76593170873926	10.23318820651862	5.88324127135786
C	5.46480342171345	11.23411425001544	4.24922812287867
C	6.22121035410489	10.19103923778454	3.63514380786476
C	6.99789462335628	9.53983557217943	4.66015863328872
C	6.32033017861094	9.98717894977900	2.16191777075246
H	5.33416282106577	9.98185005404933	1.67713293980871
H	6.85230176525519	9.06798523876071	1.89793849064659
H	7.95378266178703	7.69060790002675	5.21699443007396
Co	4.88652278533106	9.41151029024986	5.15299850039425
N	4.18653592458715	6.46358858074944	5.04903777224811
C	4.42107000702204	6.80464937761440	6.34771374402281
N	4.34558179800195	5.85280862479861	7.26750989714085
C	4.79399169200190	8.39668809001106	7.95143900988035
N	4.71358279772338	8.10970063532586	6.62776634944718
H	5.49103548811231	11.07175416067747	9.56834398920636
C	2.73875882181631	7.88375570023036	2.94850237830279
C	2.27141919558868	9.17869773750891	2.67698143851948
C	0.34133488898091	8.26449126143746	1.54538518825579
H	-0.60011782309871	8.40870224388284	1.01055254909420
C	0.81471422926840	6.96918599341794	1.79101843570770
C	2.01412927701242	6.77458479118424	2.47511522063496
C	6.45637005650598	6.07343426325891	3.06902075008465
H	6.62353261357151	5.91932286557141	4.13602067989852

C	7.07866023799259	5.63421015757909	0.77047185319761
H	5.84654159538860	6.57131976858984	-0.74326301966810
H	4.35004436982322	7.67225774209901	0.89246954189252
C	3.87287407540624	5.06020796230250	4.74219257493147
H	4.19681381722248	3.56398425362603	8.18217408940525
H	4.01770069643322	1.81803747519661	9.95639082992081
C	4.49750721701487	4.56614746108747	11.93053611326154
C	5.79700184526978	11.26825150262082	5.64982710344087
C	7.54175788814268	9.96541230566808	7.12770611740473
H	7.46593584969738	8.91458013741894	7.44523947574042
H	8.60804014130763	10.15042183757067	6.91367175248908
H	7.24440047809538	10.61568567785495	7.95554809921702
C	5.37413949531975	12.34048218445422	6.59659780254575
H	5.92341750019880	13.26702888613103	6.35831216023324
H	4.30175237224789	12.56318830110773	6.51073151290777
H	5.59057690884341	12.07565885112986	7.63614317209850
C	4.64065249456900	12.24463740826781	3.52367551117408
H	3.85029074100390	12.67157500146558	4.15662039538534
H	5.29223972527978	13.07999592447612	3.21462423891590
H	4.18496340877864	11.83070427405655	2.61375862894922
H	6.88238659230718	10.83941087480790	1.74246423093500
C	8.05902713194013	8.50370526255528	4.48430607311599
H	8.05438664867066	8.07194549708623	3.47815920294570
H	9.04625676403737	8.96769142514640	4.64944073741046
B	2.72806498146870	10.96872884879488	10.40264985237600
H	4.30347979727150	10.14147577497096	11.60219162319139
H	3.54367223162626	8.92396596692292	10.62794359465257
H	-0.03608507348304	13.25593632077431	10.25238681525082

H	-0.03255964075903	10.20353267867974	10.36715049903418
H	-0.86161039070039	12.12980057657505	9.19911877839759
C	0.07561611769523	12.27923794250940	9.76171471583812
C	0.20124116814108	11.17994021577766	10.82932204653389
H	-0.57129439303307	11.33738964646173	11.60375762415502
C	1.24730845134715	12.32832312891005	8.76538960494056
H	1.10235369826272	11.52850987039469	8.02052360703112
H	1.20075884772882	13.27246491619844	8.19246234992130
C	1.59389229868933	11.06551271408627	11.48654363575335
C	2.65695017051218	12.14901463879793	9.37021707760367
H	1.23910739218333	12.52101206318652	13.08710013866331
C	2.01611223527007	12.31127210115319	12.33003315663612
H	2.92531744218044	12.04157876072890	12.89715881484695
C	3.13829622685165	13.35053478905257	10.24632851649130
C	2.30574753233293	13.58976449831651	11.51886562596838
H	3.14782057284876	14.26740413453117	9.62975392628556
H	1.36252194168578	14.08450070495546	11.24997493248393
H	4.19056795618378	13.16911321023848	10.53353035039129
H	2.84008773143476	14.30721028157248	12.16469678346734
H	1.58338685623998	10.20051611644065	12.17226773794165
H	3.37177471092191	12.04787684592171	8.54094776144153
C	3.16150227967609	10.01217795931737	5.34790573324292
C	1.94810424123514	9.68640191593123	6.10752650978558
C	1.63679542877880	8.31407016628303	6.65978486422847
C	1.11092125684282	7.31760799098656	5.82976621474733
C	0.74770196214452	6.07780928833255	6.35707129661151
H	0.34274536313044	5.31137099027568	5.69088871727987
C	0.88818103965123	5.79855840635977	7.72659159772862

C	0.55476165437574	4.44233424854459	8.28754132011417
H	-0.12209806797074	3.88462179175507	7.62380112978578
H	0.08908836147320	4.52229838846189	9.28181007206448
H	1.47436275742268	3.84395301077186	8.40928593635604
C	1.37846528224185	6.81817840634087	8.55720118898456
H	1.48225066463056	6.64004622381705	9.63040127336068
H	0.96824576270787	7.51281996611344	4.76931975038099
C	1.74006638635639	8.05750944827132	8.03169196924321
H	2.09326360668277	8.84031503361045	8.70294755168573
O	0.84276206759506	10.34964395069274	5.58770422554289
Si	-0.84609189045829	10.29428907817745	5.87853410283763
C	-1.28184546395135	9.45787077757879	7.49858522252437
H	-0.69957312247858	9.85280319953174	8.34267255113214
H	-2.34943736821089	9.64741173046295	7.70427069734981
H	-1.12855777398168	8.36981859556166	7.45161558854522
C	-1.35399043315222	12.09788609378195	5.87418309719151
H	-0.89627880338062	12.64453924339350	6.71344203986497
H	-2.44970288768728	12.18661456155698	5.96214712352940
H	-1.04555095996936	12.58547918859456	4.93485565402182
C	-1.64095258285524	9.38726762781167	4.44424311856282
H	-2.73680124615180	9.38406915868171	4.57155074312788
H	-1.29864496777477	8.34257861159999	4.38638318537799
H	-1.40769668471169	9.87881724234324	3.48718570010951
H	2.95617017462291	10.99310544444573	4.89596045864548
H	2.38411237913567	10.28729152410544	6.97875794113620

7.2.6. Structure ThSI8

E = -4503.55335675 a.u. G= -4502.51654233 a.u.

133

P	3.79125494178041	7.54220415659745	4.16953254376683
C	5.22463031008755	6.17638894899347	8.67980367100169
N	5.33143087630526	7.47098005815088	9.01557917659739
O	4.88669608272997	9.62881070418092	8.41768748069587
C	5.21222100067088	10.05881428928830	9.77825007711447
H	6.18053628521450	9.61055028563237	10.03631123775772
C	4.12829729245223	9.71669905054130	10.78381403968025
H	2.31765491173063	9.39069336211585	2.53894832113720
C	0.47998551133861	8.42471029837904	1.97529536075051
H	0.17280329806418	9.24262324395564	1.32090471900829
H	-0.59684659681733	5.40028703492500	3.13090130029461
H	1.55534903639984	5.51132282101928	4.30910216793757
C	4.80679443038546	6.82143194127121	2.82906389646713
C	6.81387852724826	5.62883283870000	2.16346688525728
H	7.74941353776664	5.13389782672654	2.43394220616544
H	7.04189255214086	5.26503777648753	0.04252505093526
C	5.20647747257929	6.31913058303489	0.48962882354069
C	4.39937000287867	6.87252789452580	1.48633258948162
H	4.76645709413378	4.36335254618570	5.37461211668620
H	3.60183393943023	4.73376614622544	4.07547752271749
H	3.02326215810254	4.47651109749007	5.75441402084382
C	5.63798730724695	5.16155288210275	9.66231262244029
C	5.52192175082679	3.79182102733012	9.35864313568520
C	5.92035855153554	2.83346915007093	10.28912418081121
C	6.43710590562671	3.23148982516782	11.52860079510776
H	6.74913662678451	2.47825604920788	12.25646795663890
H	6.95567910133298	4.90213981320185	12.80507738818110
C	6.15539849187540	5.55495287225517	10.91119698512447
H	6.23993588289899	6.61740176404248	11.14195285736537

C	6.21965924532302	10.48462398801943	5.83759655031588
C	4.64677943677691	11.18967877594885	4.29021743458160
C	5.45567434415321	10.20378845473006	3.66062327043081
C	6.37762081808937	9.69707466163465	4.64304783828790
C	5.48462606081190	9.93800518641448	2.19427583606482
H	4.48125531947308	9.88243224356400	1.75173070665004
H	6.03646431107033	9.02737952114163	1.94077782640298
H	7.57682170744899	7.99705238289190	5.20623382787257
Co	4.43333700579494	9.34939368832678	5.34200442546958
N	3.98178619562790	6.32809988674892	5.39290119183445
C	4.47760653489150	6.71348692555129	6.60171267415275
N	4.73111726275786	5.77303666874618	7.50434342229311
C	4.99310318709860	8.35851664699407	8.09924564491267
N	4.69400675937484	8.04653892490538	6.81240590742771
H	5.33569223857556	11.14372600303102	9.67178091787601
C	2.10926478081037	7.44648295470475	3.48447653744074
C	1.69521381013341	8.50351473967604	2.65830601024736
C	-0.34549125793653	7.30912202830557	2.13871697474015
H	-1.30050836947233	7.25358163415953	1.61106436205571
C	0.04999632763953	6.26827179415863	2.98526115113834
C	1.27579458120356	6.32829812626275	3.64890389655041
C	6.01584208605741	6.18719225660876	3.16237872908155
H	6.33402411596661	6.11698801489327	4.20310270196267
C	6.41493909508020	5.70069660303091	0.82415105774331
H	4.88304944053413	6.36705377631804	-0.55268987579527
H	3.45502684743529	7.34135859189600	1.20938755445255
C	3.83217934352271	4.88657580065174	5.13420392049866
H	5.11798244370628	3.49387392414042	8.39055316395338
H	5.82891932107715	1.77142379413242	10.04978898819775
C	6.55377526375845	4.59239890950028	11.83758100986550
C	5.13093426070613	11.37138659782567	5.64797461478134
C	7.19229913273737	10.44032918938315	6.96529569896861
H	7.37023315801232	9.41777409480108	7.32765228441943

H	8.15970326805907	10.80678378826637	6.58007155637956
H	6.89819830468954	11.07865691684591	7.80362047459827
C	4.68409606144297	12.43176949568778	6.59716376933216
H	5.12745601183816	13.39575425256251	6.29460945104870
H	3.59467960957457	12.57088672290818	6.60276109860205
H	5.00505140868248	12.21743164584237	7.62225092976600
C	3.70073627906733	12.07856253046837	3.55027119809470
H	3.14633546311731	12.75178181419510	4.21522509390933
H	4.28187693392038	12.70776146513805	2.85474923425030
H	2.98415017551554	11.50761700148453	2.94525088479664
H	6.00097791612479	10.79027363448071	1.71829607114150
C	7.52301613282683	8.76786539954843	4.42346680719099
H	7.47805516915875	8.27598559770066	3.44676636089654
H	8.46232892305831	9.34499200419099	4.47113350213328
B	2.69230760454412	10.36065811801977	10.65668747473195
H	4.48916768499659	10.07816742982925	11.76863756130464
H	4.06012565815050	8.62439079309692	10.91763318604127
H	-0.55338018509865	11.91973252512968	10.77474133804994
H	0.18113391886099	8.96646712699132	10.51326678773081
H	-1.11097042838391	10.75841062604314	9.58994954726562
C	-0.22263098189926	11.06352857265935	10.16975566089669
C	0.18994881083862	9.90581843231686	11.09379751879831
H	-0.57376530998604	9.78255280585523	11.88326093579173
C	0.87496585303954	11.50565526982572	9.18509223014719
H	0.89198374204407	10.78791431571857	8.35095255329907
H	0.59359181492563	12.47617491087904	8.73765349165811
C	1.58867729863965	10.04986363032248	11.73233776636008
C	2.30776187457045	11.58866215428879	9.75698618094183
H	0.94058434690475	11.18684377152541	13.49316431472762
C	1.72722198443863	11.25408172915543	12.72009535148438
H	2.68776965199680	11.14177887964621	13.25369040929805
C	2.52111533085441	12.76052552430125	10.77271852089951
C	1.69065843612537	12.64943162279133	12.06492053556957

H	2.29727536718336	13.71804133464604	10.26884429064986
H	0.65136709599063	12.93729661023476	11.85655322365253
H	3.59406764211295	12.80119166408542	11.03590564031289
H	2.06084141648348	13.39301219374184	12.79119555850822
H	1.80288262355032	9.13371701535529	12.30957358212203
H	3.00308718502781	11.76157516887819	8.92125392747186
C	1.83424675891996	10.50294802567372	5.63960745341276
H	2.27283828065121	11.50145179204321	5.58332074389904
C	2.32770437902307	9.62313125217671	6.57890219135165
C	1.64363905881323	8.41428071775126	7.06949907949983
C	0.63311271769561	7.73692498061334	6.36157153291511
C	0.04435803905657	6.58409102018125	6.88225234793839
H	-0.72344099498454	6.07232682192849	6.29565601720069
C	0.41769914289661	6.06377163273655	8.13032098131502
C	-0.18015608389486	4.78897840813112	8.66363495427066
H	-1.11853326732738	4.53585498562625	8.14849571382460
H	-0.38035446650448	4.86156828007450	9.74412940696535
H	0.51700685281885	3.94454408688584	8.52108613131529
C	1.37888299941292	6.77456573022045	8.86975171330002
H	1.66891693738645	6.42593370229477	9.86447084169474
H	0.30772935324711	8.11207772621288	5.39757700981071
C	1.97277101330338	7.91983136904501	8.34930522810266
H	2.69108554819212	8.45679545575767	8.96393782780189
H	3.03073509436652	10.08936346287834	7.26942829880173
O	0.79802275435579	10.26960207396679	4.87722324512363
Si	-0.44744990812821	11.38222746739660	4.34126187848551
C	-0.64326053429859	12.62256776744300	5.72553784459280
H	0.28507946261147	13.19337663431688	5.89131310924798
H	-1.44035303085903	13.34055467711739	5.47030857984162
H	-0.91858701579332	12.12122298370292	6.66706050460269
C	0.10852134089220	12.19030445157991	2.75121328369810
H	0.93083122946631	12.89893322567597	2.93112046385755
H	-0.73810636757409	12.75488352717766	2.32514995973226

H	0.43475556253859	11.45195456887904	2.00380386649215
C	-1.92812618603131	10.26928482624561	4.14417863190878
H	-2.80032322889367	10.85789551749558	3.81493493418879
H	-2.17555059852302	9.79014497282413	5.10510770758158
H	-1.73937539561524	9.48154705727896	3.40001673090023

8. X-ray Structural Analysis and Refinement

Suitable single crystals of complexes **1** (CCDC 2169090), **6** (CCDC 2169092), and **7** (CCDC 2169091) were selected under a microscope in polarized light with an applied nitrogen cryo-stream at about -40 °C and covered with polyfluorinated polyether. The crystals were picked with nylon loops of suitable sizes and rapidly mounted in the nitrogen cold gas stream of the diffractometer at 100 K. A Bruker D8 Venture diffractometer with IµS3 Diamond source, INCOATEC Helios mirror optics (Mo-Kα radiation; λ=0.71073 Å), and Photon III detector was used for data collection. Final cell constants were obtained from least-squares fits of setting angles of several thousand strong reflections. Intensity data were corrected for absorption using intensities of redundant reflections using SADABS.[10] The structures were readily solved by Direct and Patterson methods and subsequent Fourier techniques. The Bruker APEX4[11] software package was used for the solution and refinement of the structures. All non-hydrogen atoms were anisotropically refined and hydrogen atoms were placed at calculated positions and refined as riding atoms with isotropic displacement parameters.

The refinement of crystal structure **1** showed that both SbF_6^- anions were slightly disordered. A split atom model was used to account for the disorder. Displacement parameters of antimony and fluorine atoms were restrained using EADP, ISOR, and SIMU. ShelXL SADI and SAME instructions were used to restrain 1,2- and 1,3-distances of the anions. A total of 504 restraints were used.

A similar disorder of the SbF_6^- anions was found in crystals of complex **6** and was treated accordingly. The asymmetric unit additionally contains three molecules of dichloromethane of which two are disordered. Split atom models using EADP, ISOR, and SAME restraints (87 restraints) were used to treat the disorder. Crystallographic details of data collection and refinement of the structures are shown in Table S5.

Table S5. Crystallographic data of complexes.

	Complex 1	Complex 6	Complex 7
Formula	$C_{51}H_{65}BCoF_{12}N_8OPSb_2$	$C_{39}H_{47}Cl_6CoF_{12}N_5OPSb_2$	$C_{16}H_{24}CoF_{12}N_3Sb_2$
M_r in g mol^{-1}	1378.32	1375.91	788.81
Color, habit	red, parallelepiped	red-brown, parallelepiped	red, parallelepiped
Crystal system	Monoclinic	Monoclinic	Monoclinic
Space group	$P2_1/n$	$P2_1/c$	$P2_1/c$
a in Å	14.1823(7)	23.7011(11)	16.9549(5)
b in Å	17.7060(8)	13.0582(6)	9.7010(3)
c in Å	23.3122(11)	17.0766(8)	15.8243(5)
β in °	102.916(2)	103.440(2)	90.5730(10)
V in Å^3	5705.9(5)	5140.4(4)	2602.64(14)
Z	4	4	4
T in K	100(2)	100(2)	100(2)
Crystal size in mm^3	0.17 × 0.14 × 0.14	0.15 × 0.15 × 0.12	0.055 × 0.045 × 0.025
ρ_c in g cm^{-3}	1.604	1.778	2.013
F(000)	2768	2712	1512
Diffractometer	Bruker-D8 Venture	Bruker-D8 Venture	Bruker-D8 Venture
$\lambda_{XK\alpha}$ in Å	X = Mo<br>0.71073	X = Mo<br>0.71073	X = Mo<br>0.71073
θ_{min} in °<br>θ_{max} in °	1.888<br>35.000	1.792<br>45.343	2.403<br>36.338
Index range	$-22 \leq h \leq 22$<br>$-28 \leq k \leq 28$<br>$-37 \leq l \leq 37$	$-47 \leq h \leq 47$<br>$-26 \leq k \leq 26$<br>$-34 \leq l \leq 34$	$-28 \leq h \leq 28$<br>$-16 \leq k \leq 16$<br>$-26 \leq l \leq 26$
μ in mm^{-1}	1.339	1.786	2.786
Abs. correction	multi-scan	multi-scan	multi-scan
Reflections collected	266788	760438	138191
Reflections unique	25125	43079	12629
R_{int}	0.0432	0.0450	0.0540
Reflections obs. [F>2σ(F)]	22072	37248	10320
Residual density in e Å^{-3}	2.02/-1.20	2.83/-1.51	2.02/-1.22
Params/restraints	748/504	676/87	315/0
GOOF	1.096	1.157	1.015
R_1 [I>2σ(I)]	0.0320	0.0395	0.0268
wR_2 (all data)	0.0387	0.0478	0.0385
CCDC	2169090	2169092	2169091

8.1. Molecular Structure of 1 (CCDC 2169090)

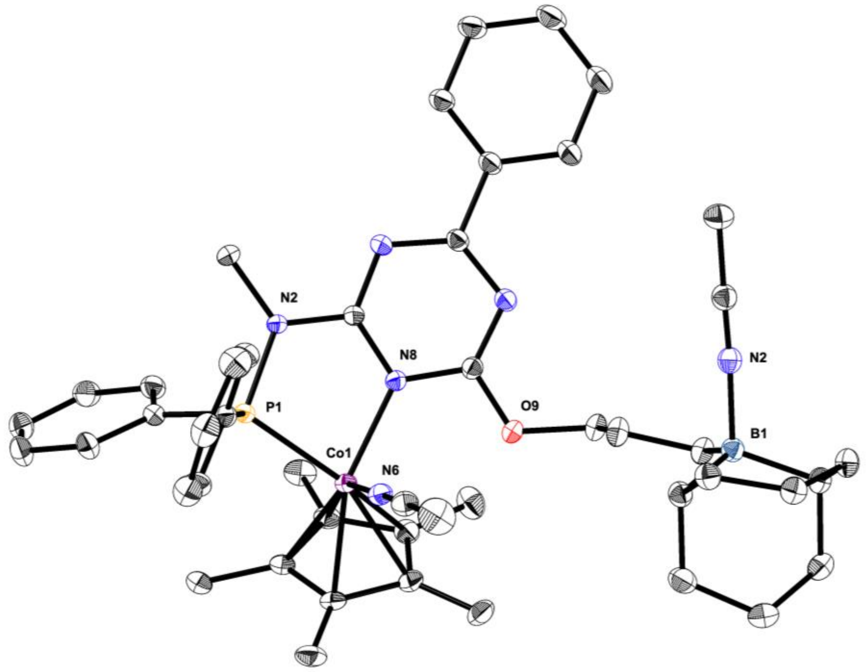

Figure S128 – Molecular structure of complex **1** in the solid-state. The SbF_6 anions, hydrogen atoms, and residual solvent molecules (MeCN) were omitted for clarity.

Molecular structure of complex **1**. Suitable single crystals materialized through layering a concentrated CH_3CN solution (0.4 mL) of complex **1** with Et_2O (4 mL). The displayed molecular structure demonstrates thermal ellipsoids at the 40% probability level for enhanced visualization. To maintain clarity, SbF_6 anions, hydrogen atoms, and residual solvent molecules (MeCN) were deliberately excluded.

8.2. Molecular Structure of 6 (CCDC 2169092)

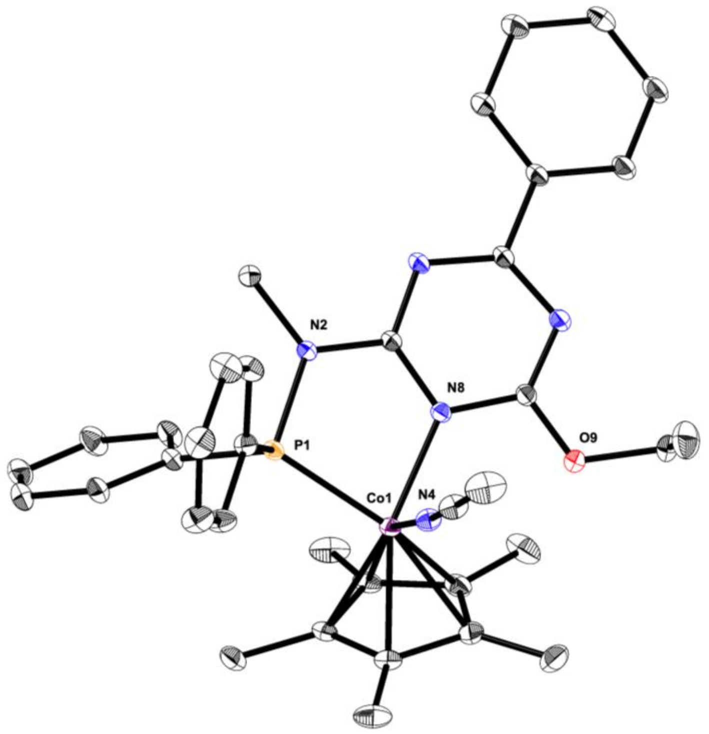

Figure S129 – Molecular structure of complex **6** in the solid-state. The SbF_6 anions, hydrogen atoms, and residual solvent molecules (CH_2Cl_2) were omitted for clarity.

Molecular structure of complex **6**. Suitable single crystals materialized through layering a concentrated CH_2Cl_2 solution (0.4 mL) of complex **6** with pentane (4 mL). The displayed molecular structure demonstrates thermal ellipsoids at the 40% probability level for enhanced visualization. To maintain clarity, SbF_6 anions, hydrogen atoms, and residual solvent molecules (CH_2Cl_2) were deliberately excluded.

8.3. Molecular Structure of 7 (CCDC 2169091)

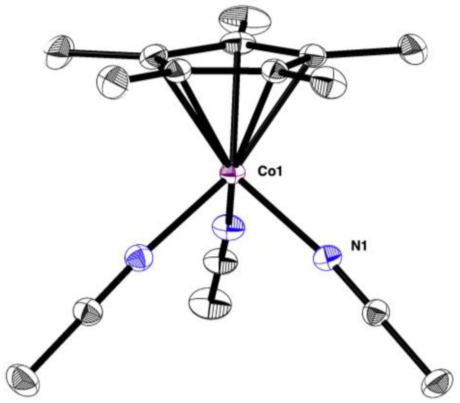

Figure S130 – Molecular structure of complex **7** in the solid-state. The SbF_6 anions, hydrogen atoms, and residual solvent molecules (CH_2Cl_2) were omitted for clarity.

Molecular structure of complex **7**. Suitable single crystals materialized through layering a concentrated CH_2Cl_2 solution (0.4 mL) of complex **7** with pentane (4 mL). The displayed molecular structure demonstrates thermal ellipsoids at the 40% probability level for enhanced visualization. To maintain clarity, SbF_6 anions, and hydrogen atoms were deliberately excluded.

9. References

[1] Yu, D. G.; Gensch, T.; de Azambuja, F.; Vasquez-Cespedes, S.; Glorius, F. Co(III)-catalyzed C-H activation/formal SN-type reactions: selective and efficient cyanation, halogenation, and allylation. *J. Am. Chem. Soc.* **2014**, *136*, 17722-17725.

[2] Chugh, V.; Chatterjee, B.; Chang, W. C.; Cramer, H. H.; Hindemith, C.; Randel, H.; Weyhermüller, T.; Farès, C.; Werlé, C. An Adaptive Rhodium Catalyst to Control the Hydrogenation Network of Nitroarenes. *Angew. Chem., Int. Ed.* **2022**, *61*, e202205515.

[3] Tobisu, M.; Takahira, T.; Morioka, T.; Chatani, N. Nickel-Catalyzed Alkylative Cross-Coupling of Anisoles with Grignard Reagents via C-O Bond Activation. *J. Am. Chem. Soc.* **2016**, *138*, 6711-6714.

[4] Dias, E. L.; Brookhart, M.; White, P. S. Rhodium(I)-catalyzed homologation of aromatic aldehydes with trimethylsilyldiazomethane. *J. Am. Chem. Soc.* **2001**, *123*, 2442-2443.

[5] Lemus, C.; Poleschak, M.; Gailly, S.; Desage-El Murr, M.; Koch, M.; Deguin, B. Lewis acid catalyzed enlargement of cyclic beta-alkoxyenals and one-pot synthesis of polyfunctional enoxysilanes derived from aucubin with trimethylsilyldiazomethane. *Chem. - Eur. J.* **2013**, *19*, 4686-4690.

[6] Gao, L.; Yang, W.; Wu, Y.; Song, Z., The Brook Rearrangement, In *Organic Reactions*, **2020**, pp 1-612.

[7] Burgos, C. H.; Canales, E.; Matos, K.; Soderquist, J. A. Asymmetric allyl- and crotylboration with the robust, versatile, and recyclable 10-TMS-9-borabicyclo[3.3.2]decanes. *J. Am. Chem. Soc.* **2005**, *127*, 8044-8049.

[8] a) Neese, F. The ORCA program system. *Wiley Interdiscip. Rev. Comput. Mol. Sci.* **2012**, *2*, 73-78; b) Neese, F.; Wennmohs, F.; Becker, U.; Riplinger, C. The ORCA quantum chemistry program package. *J. Chem. Phys.* **2020**, *152*, 224108; c) Neese, F. Software update: The ORCA program system-Version 5.0. *Wiley Interdiscip. Rev. Comput. Mol. Sci.* **2022**, *12*, e1606.

[9] a) Becke, A. D. Density-functional exchange-energy approximation with correct asymptotic behavior. *Phys. Rev. A* **1988**, *38*, 3098-3100; b) Lee, C.; Yang, W.; Parr, R. G. Development of the Colle-Salvetti correlation-energy formula into a functional of the electron density. *Phys. Rev. B* **1988**, *37*, 785-789; c) Andrae, D.; Häußermann, U.; Dolg, M.; Stoll, H.; Preuß, H. Energy-adjustedab initio pseudopotentials for the second and third row transition elements. *Theoretica chimica acta* **1990**, *77*, 123-141; d) Becke, A. D. Density-functional thermochemistry. I. The effect of the exchange-only gradient correction. *J. Chem. Phys.* **1992**, *96*, 2155-2160; e) Weigend, F.; Ahlrichs, R. Balanced basis sets of split valence, triple zeta valence and quadruple zeta valence quality for H to Rn: Design and assessment of accuracy.

Phys. Chem. Chem. Phys. **2005**, *7*, 3297-3305; f) Weigend, F. Accurate Coulomb-fitting basis sets for H to Rn. *Phys. Chem. Chem. Phys.* **2006**, *8*, 1057-1065; g) Weigend, F. Hartree–Fock exchange fitting basis sets for H to Rn. *J. Comput. Chem.* **2008**, *29*, 167-175; h) Bykov, D.; Petrenko, T.; Izsak, R.; Kossmann, S.; Becker, U.; Valeev, E.; Neese, F. Efficient implementation of the analytic second derivatives of Hartree-Fock and hybrid DFT energies: a detailed analysis of different approximations. *Mol. Phys.* **2015**, *113*, 1961-1977; i) Caldeweyher, E.; Bannwarth, C.; Grimme, S. Extension of the D3 dispersion coefficient model. *J. Chem. Phys.* **2017**, *147,* 034112; j) Caldeweyher, E.; Ehlert, S.; Hansen, A.; Neugebauer, H.; Spicher, S.; Bannwarth, C.; Grimme, S. A generally applicable atomic-charge dependent London dispersion correction. *J. Chem. Phys.* **2019**, *150,* 154122; k) Garcia-Rates, M.; Neese, F. Efficient implementation of the analytical second derivatives of hartree-fock and hybrid DFT energies within the framework of the conductor-like polarizable continuum model. *J. Comput. Chem.* **2019**, *40*, 1816-1828; l) Garcia-Rates, M.; Neese, F. Effect of the Solute Cavity on the Solvation Energy and its Derivatives within the Framework of the Gaussian Charge Scheme. *J. Comput. Chem.* **2020**, *41*, 922-939; m) Neese, F. The SHARK integral generation and digestion system. *J. Comput. Chem.* **2023**, *44*, 381-396.

[10] Krause, L.; Herbst-Irmer, R.; Sheldrick, G. M.; Stalke, D. Comparison of silver and molybdenum microfocus X-ray sources for single-crystal structure determination. *J. Appl. Crystallogr.* **2015**, *48*, 3-10.

[11] *APEX4 program package*, v2022.1-1; Bruker AXS: Karlsruhe, Germany, 2022.
